# Design, Synthesis, *In Vitro* and *In Vivo* Characterization of Selective NKCC1 Inhibitors for the Treatment of Core Symptoms in Down Syndrome

**DOI:** 10.1021/acs.jmedchem.1c00603

**Published:** 2021-06-17

**Authors:** Marco Borgogno, Annalisa Savardi, Jacopo Manigrasso, Alessandra Turci, Corinne Portioli, Giuliana Ottonello, Sine Mandrup Bertozzi, Andrea Armirotti, Andrea Contestabile, Laura Cancedda, Marco De Vivo

**Affiliations:** † Molecular Modeling and Drug Discovery Laboratory, Istituto Italiano di Tecnologia, via Morego, 30, 16163 Genoa, Italy; ‡ Brain Development and Disease Laboratory, Istituto Italiano di Tecnologia, via Morego, 30, 16163 Genoa, Italy; § Dulbecco Telethon Institute, 38123 Rome, Italy; ∥ Università degli Studi di Genova, via Balbi, 5, 16126 Genoa, Italy; ⊥ Analytical Chemistry Facility, Istituto Italiano di Tecnologia, via Morego, 30, 16163 Genoa, Italy

## Abstract

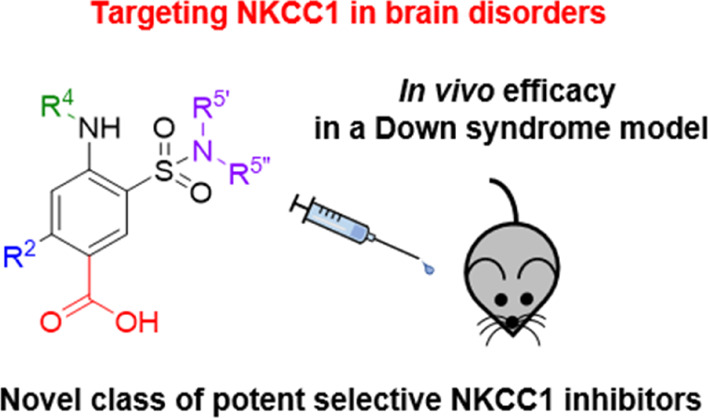

Intracellular chloride concentration [Cl^–^]_i_ is defective in several neurological disorders. In neurons, [Cl^–^]_i_ is mainly regulated by the action of the Na^+^–K^+^–Cl^–^ importer NKCC1 and the K^+^–Cl^–^ exporter KCC2. Recently, we have reported the discovery of **ARN23746** as the lead candidate of a novel class of selective inhibitors of NKCC1. Importantly, **ARN23746** is able to rescue core symptoms of Down syndrome (DS) and autism in mouse models. Here, we describe the discovery and extensive characterization of this chemical class of selective NKCC1 inhibitors, with focus on **ARN23746** and other promising derivatives. In particular, we present compound **40** (**ARN24092**) as a backup/follow-up lead with *in vivo* efficacy in a mouse model of DS. These results further strengthen the potential of this new class of compounds for the treatment of core symptoms of brain disorders characterized by the defective NKCC1/KCC2 expression ratio.

## Introduction

In recent years, a large body of constantly increasing experimental evidence has indicated modulation of intracellular chloride concentration [Cl^–^]_i_ as a valuable therapeutic strategy for a number of neurological conditions, including Down syndrome (DS).^1−3^ [Cl^–^]_i_ is mainly regulated in neurons by the sodium (Na^+^)–potassium (K^+^)–chloride (Cl^–^) importer NKCC1 and the K^+^–Cl^–^ exporter KCC2. Both in brain samples from human subjects with DS and in the most widely used mouse model of DS (the Ts65Dn mouse), expression of NKCC1 is upregulated, which leads to an augmented NKCC1/KCC2 expression ratio. Moreover, similar variations in the Cl transporters’ ratio (due either to higher expression of NKCC1 and/or to lower expression of KCC2) were observed in several other brain disorders, both in human samples and in animal models.^1,2,4^ These alterations lead to an increased [Cl^–^]_i_ in neurons, which in turn affects the neuronal function in brain disorders.^1^

**Figure 1:**
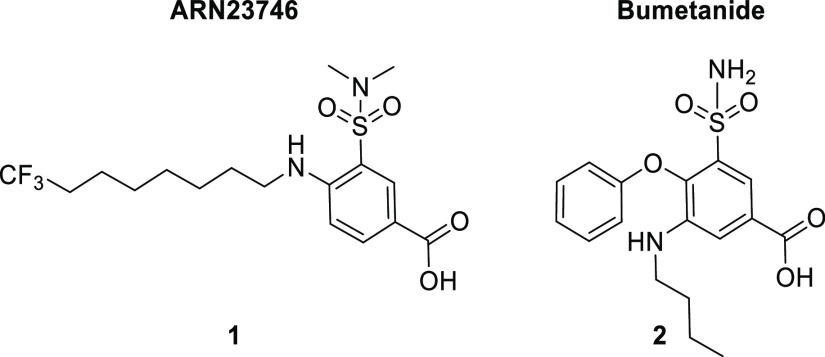
Structures of **ARN23746** and bumetanide.

In this context, we have recently reported the discovery of 3-(*N*,*N*-dimethylsulfamoyl)-4-((8,8,8-trifluorooctyl)amino)benzoic acid, compound **1** (**ARN23746** in Figure 1), as a potent and selective NKCC1 inhibitor, with *in vivo* efficacy in mouse models of DS and autism, thus potentially also in other neurodevelopmental disorders characterized by impaired [Cl^–^]_i_.^5^ This lead compound belongs to a chemical class of 4-amino-3-(alkylsulfamoyl)-benzoic acids. This chemical class markedly differs from previous unselective inhibitors such as the FDA-approved diuretic bumetanide, **2** (Figure 1). Indeed, being selective for NKCC1, this chemical class has a safer pharmacological profile for chronic use to treat brain disorders because it is devoid of unwanted diuretic effects (caused by inhibition of the isoform NKCC2 in the kidney, as in the case of bumetanide). This new class of compounds may thus provide a new, better, and safer therapeutic approach for DS and possibly a large panel of neurological diseases characterized by the NKCC1/KCC2 defective expression ratio.

Several studies have indeed indicated that bumetanide rescues [Cl^–^]_i_ and behavioral deficits in the Ts65Dn mouse model of DS,^6^ as well as in mouse models of a number of other brain disorders.^2,7^ Most notably, bumetanide treatment has shown positive outcomes also in humans during several clinical trials and case studies of neurodevelopmental disorders (autism,^8−18^ Fragile X,^19^ Asperger syndrome,^20^ 15q11.2 duplication,^21^ schizophrenia,^22,23^ and tuberous sclerosis complex^24,25^), neurodegenerative disorders (Parkinson disease^26^), and also neurological disorders (epilepsy^27−30^ and neuropathic pain^31^). Nevertheless, the strong diuretic effect of bumetanide severely endangers drug compliance, while also leading to hypokalaemia and general ionic imbalance,^10^ ototoxicity in young individuals,^32^ and potential kidney damage upon chronic treatments.^33−37^ As such, bumetanide and its close analogues and prodrugs^38−41^ have severe limitations and downsides when considered as a clinical option to treat brain disorders. Moreover, the fact that the bumetanide’s pharmacological effect is washed out after treatment interruption^6,11^ implies that a lifelong administration of this drug would be required, thus with patients subjected to bumetanide-induced excessive diuresis (and related electrolytes imbalance issues) during chronic treatments.

In this regard, our new compound **1**, as others compounds from this new chemical class, shows no increased diuresis, *in vivo*, thanks to their selective action on NKCC1. This major benefit of **1** thus may allow us to overcome the limitations and drawbacks related to bumetanide and other unselective diuretics. However, in the first disclosure of compound **1**,^5^ we reported its *in vitro* and *in vivo* efficacy and overall drug-like profile, while we only briefly described the computational and medicinal chemistry effort for its discovery and characterization. Here, we describe in detail how compound **1** (Figure 1) was designed, optimized, and developed into a lead molecule ready to enter into advanced preclinical studies. Compound **1** is the result of an exhaustive structure–activity relationship (SAR) study based on modeling and synthesis and extended characterization *in vitro* and *in vivo*. This overall neuroscience drug discovery effort is here presented with 42 new compounds. In addition to the resulting SAR, we highlight the identification and characterization *in vitro* and *in vivo* of a promising backup/follow-up molecule, that is, compound **40** (**ARN24092**).

## Results and Discussion

### Ligand-Based Library Screening

When we started our drug discovery campaign toward novel selective NKCC1 inhibitors, we could apply a ligand-based drug-design strategy by building a pharmacophoric model templated on the bumetanide’s structure. We refined the first bumetanide’s pharmacophore by superimposing it with structures of other unspecific NKCC1 inhibitors (i.e., furosemide, azosemide, piretanide, benzmetanide, bendroflumethiazide, benzthiazide, chlorothiazide, metolazone, and quinethazone—vide infra). We used this model as a search filter for the virtual screening of our institution’s chemical library and other chemical libraries from commercial vendors (∼135,000 compounds, in total). This computational effort identified a total of 253 compounds that we tested at two concentrations (10 and 100 μM) in a Cl^–^ influx assay in HEK293 cells lines transfected with NKCC1.^5^ Among these 253 compounds, we identified the two structurally related 2-amino-5-nitro-benzenesulfonamide derivatives **3** (**ARN22393**) and **4** (**ARN22394**), diversified for the substituent on the amino group (Table 1). Compound **3**, with an *n*-hexyl chain on the amino group and a methylated sulfonamide, showed NKCC1 inhibitory activities of 6.4 ± 3.4% at 10 μM and 29.4 ± 2.8% at 100 μM. Also, compound **4**, bearing a 3,3-dimethylbutyl chain on the amino group and a methylated sulfonamide, showed NKCC1 inhibitory activities of 17.7 ± 3.9% at 10 μM and 28.7 ± 4.4% at 100 μM. Despite the moderate potency in NKCC1 inhibition compared to bumetanide (58.8 ± 5.6% at 10 μM and 71.7 ± 7.0% at 100 μM), **3** and **4** were considered promising hit compounds due to their basal activity and good chemical tractability. These were thus selected as a starting point for the design and synthesis of new derivatives.

**Table 1:**
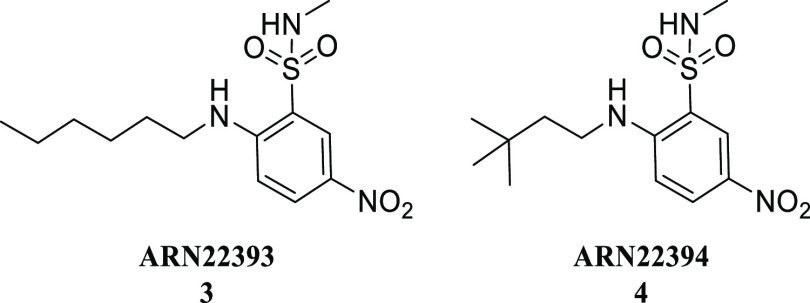
Structure and Activity of Hit Compounds **ARN22393** and **ARN22394** Tested in the Cl^–^ Influx Assay

entry	10 μM	100 μM
bumetanide	58.8 ± 5.6%	71.7 ± 7.0%
**3**	6.4 ± 3.4%	29.4 ± 2.8%
**4**	17.7 ± 3.9%	28.7 ± 4.4%

### Hit to the Lead Process toward the Discovery of Compound **1** (**ARN23746**)

**Figure 2:**
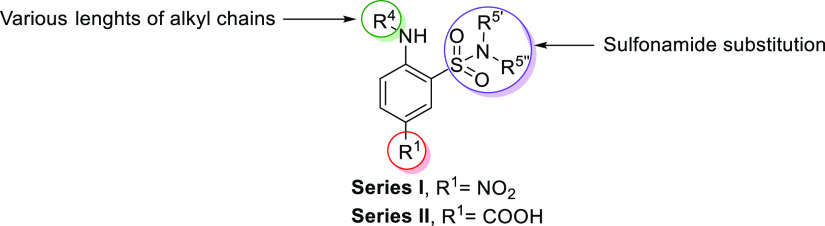
Representation of the points subject to chemical manipulation of **3** (**ARN22393**) and **4** (**ARN22394**).

We synthesized new compounds based on the chemical core of **3** and **4** (Table 1). Two series of analogues were generated based on the substituents in position R^1^ of the aromatic ring: a 2-amino-5-nitro-benzene-sulfonamide series (Series I, Figure 2) and a 4-amino-3-sulfamoyl-benzoic acid series (Series II, Figure 2). In particular, we compared the nitro group with the carboxylic acid, with the latter found to be a crucial feature for NKCC1 inhibition,^5^ although suboptimal for neuroscience drug design.^42^

For both series, in short, we explored: (i) substitutions on the amino group (R^4^, Figure 2) through the insertion of an alkyl side chain of different lengths (C4 to C8) and (ii) the sulfonamide via its methylation, as compared to dimethylation (R^5′^, R^5″^, Figure 2). Taken together, this investigation allowed us to test the influence of these specific modifications and decipher how the different dispositions of similar substituents of our new class of compounds, when compared to bumetanide,^5^ affect activity and selectivity against NKCC1.

### Series I and Series II Exploration and Development

#### Series I

First, we compared the inhibitory activity of the newly synthetized 2-amino-5-nitro-benzene-sulfonamide derivatives of Series I, with the hit compounds **3** and **4** (Table 2). For this purpose, all compounds were tested in the Cl^–^ influx assay. Notably, all 10 derivatives showed lower activity compared with the hit compounds **3** and **4**. While at 100 μM, some compounds retained moderate activity, all compounds were inactive at 10 μM. For example, shortening/reducing the bulk of the chain on the amino group, as in **5**, returned no activity against NKCC1 at 10 μM. The *n*-hexyl chain, as in **6**, also had no inhibitory activity at 10 μM. Likewise, elongation of the aminoalkyl chain to *n*-octyl combined with all the differently substituted sulfonamide on R^2^ (Table 2), as in **7**, **10**, and **13**, had no detectable inhibitory activity at both 10 and 100 μM. In addition, the overall Series I suffered from poor kinetic solubility, often below 10 μM in an aqueous medium. For instance, the poor solubility of the derivatives bearing alkylated sulfonamides (**11**, **13**, and **14**, Table 2) did not allow us to perform the Cl^–^ influx assay at high concentration (Table 2). Nevertheless, we noted that the presence of a primary sulfonamide in **5** was able to give a modest enhancement in the kinetic solubility compared to related methylated counterpart **9**. Finally, a poor profile in terms of metabolic stability *in vitro* (**3–7** and **12**, Table 2) was also found as a further drawback of Series I (Table 2).

**Table 2:**
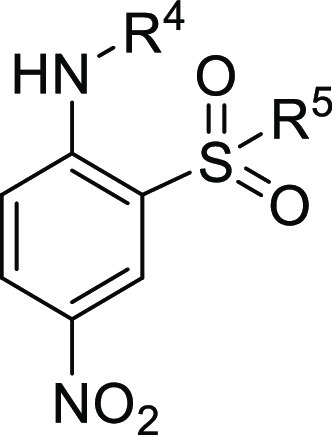

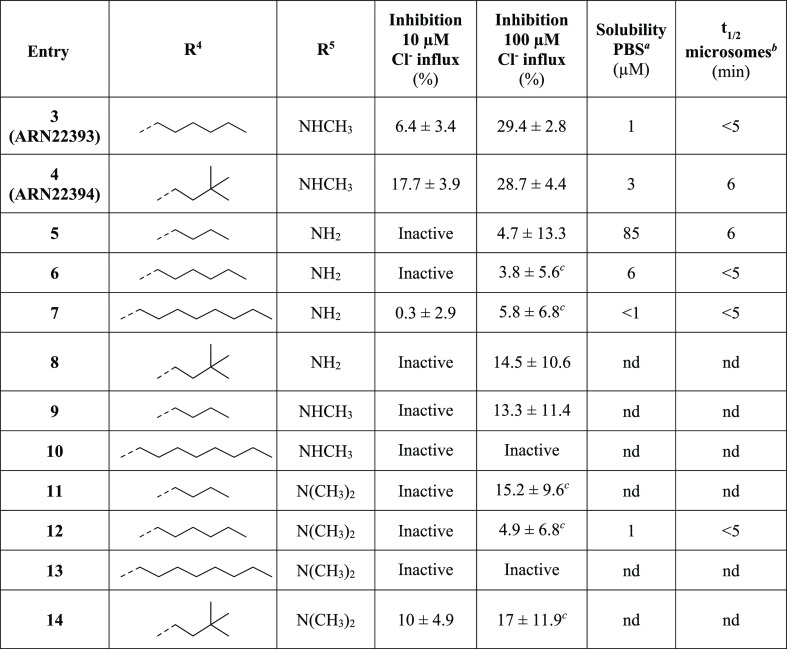
Inhibitory Activity of 2-Amino-5-nitro-benzene-sulfonamide Derivatives and *In Vitro* Chemical Stability and Solubility of the Selected Derivatives

a Aqueous kinetic solubility of compounds from a 10 mM DMSO solution in PBS at pH 7.4. Target concentration is 250 μM (final DMSO 2.5%).

b Metabolic stability in mouse liver homogenates. Compounds were incubated at 5 μM (final DMSO 0.1%).

c Precipitation observed in the assay buffer.

Overall, the manipulation of the substituents in Series I did not lead to better compounds compared to the starting hits. Consequently, we deprioritized further synthetic efforts toward the investigation of this chemical series. Our decision to deprioritize this chemical class was also based on the fact that compounds with nitro groups (especially nitro aromatic compounds) are known to possibly induce severe toxicity, *in vivo* (i.e., carcinogenicity, hepatotoxicity, mutagenicity, and bone marrow suppression).^43^

#### Series II

This series of NKCC1 inhibitors is characterized by the replacement of the nitro group (Series I) with the carboxylic acid (Table 3), confirming the importance of this acid moiety on the aromatic core for activity on NKCC1, as shown by precedent data acquired from bumetanide analogues.^5^ Also, the presence of the carboxylic acid resulted in enhanced solubility (Table 3). Moreover, metabolic stability, although suboptimal, was slightly improved with exception of derivatives bearing linear chains, especially when combined with the dimethylated sulfonamide (**21** = 12 min and **25** = 13 min). In particular, these compounds were burdened by a short half-life in mouse microsomes compared to the branched 3, 3-dimethylbutyl-substituted analogues (**26** = >60 min). This suggests the linear alkyl substituent on the amine as a privileged point of metabolism.

**Table 3:**
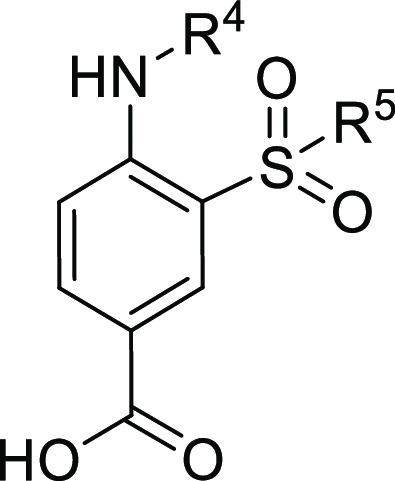

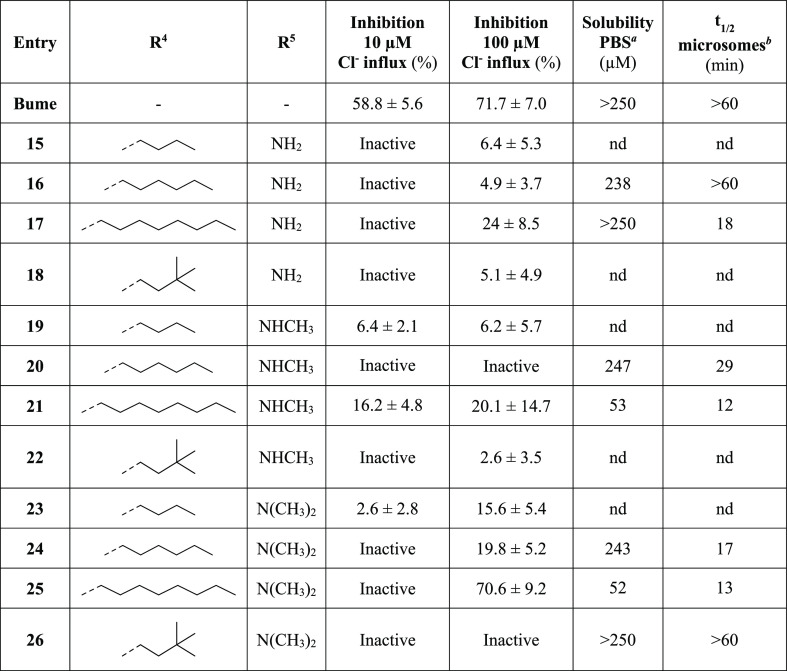
Inhibitory Activity of 4-Amino-3-sulfamoyl-benzoic Acid Derivatives and *In Vitro* Metabolic Stability and Solubility of Selected Derivatives

a Aqueous kinetic solubility of compounds from a 10 mM DMSO solution in PBS at pH 7.4. Target concentration is 250 μM (final DMSO 2.5%).

b Metabolic stability in mouse liver homogenates. Compounds were incubated at 5 μM (final DMSO 0.1%).

With respect to activity, the derivatives bearing a short *n*-butyl chain as in compounds **15**, **19**, and **23** displayed a decrease in potency at 100 μM (**15** = 6.4 ± 5.3%, **19** = 6.2 ± 5.7%, and **23** = 15.6 ± 5.4%; Table 3) when compared to the hit compounds. The combination of the dimethylated sulfonamide with elongation of the chain on the amino group to the *n*-hexyl motif resulted in comparable activity to the hit compounds at 100 μM, as for **24** (19.8 ± 5.2%). On the other hand, elongation of such a chain combined with primary and secondary sulfonamides returned very low activity (**16** = 4.9 ± 3.9%; **20** = inactive). Interestingly, insertion of the *n*-octyl chain resulted in different effects on the inhibitory activity depending on the nature of the substituent present on the sulfonamide. For example, compounds **17** and **21**, bearing, respectively, a primary or methylated sulfonamide, displayed an increase in activity at 100 μM (**17** = 24 ± 8.5% and **21** = 20.1 ± 14.7%). Notably, combination of the *n*-octyl chain with the *N*,*N*-dimethylsulfonamide, as in derivative **25**, resulted in a boost of the inhibitory effect, with an activity similar to that of bumetanide (71.7 ± 7%) at 100 μM. Surprisingly, combination of the carboxylic acid with the 3,3-dimethylbutyl alkyl chain as in compounds **18**, **22**, and **26** led to a substantial decrease (or even loss) in activity compared to the corresponding structurally related hit compound **4** (**18**, 10 μM = inactive and 100 μM = 5.1 ± 4.9%; **22**, 10 μM = inactive and 100 μM = 2.6 ± 3.5%; and **26**, 10 μM = inactive and 100 μM = inactive).

Altogether, these results indicate that insertion of a carboxylic acid on the aromatic ring is valuable in terms of inhibitory activity, which prompted us to further evaluate derivatives of this benzoic acid-based chemical scaffold. Moreover, the *n*-octyl chain gave the best results in terms of NKCC1 inhibitory activity. However, we noted that all combinations of different alkyl chains with the *N*,*N*-dimethyl sulfonamide appeared to be crucial for activity. Nevertheless, this promising class still needed to be improved, aiming at better activity and drug-like properties such as solubility and metabolic stability (Table 3).

### Alkyl Chain Substituent Optimization in Series II

In an effort to further enhance potency and tune the solubility and metabolic stability of Series II compounds, we evaluated two different substitutions in the terminal point of the alkyl chain on the amino group. Such modifications were combined with the dimethylated sulfonamide motif, which gave the best results in terms of NKCC1 inhibitory activity (Table 3). Thus, we introduced on the linear alkyl chain (C4, C6, and C8) a polar methyl ether terminal (**29–31**, Table 4) or a more lipophilic trifluoromethyl moiety (**1** and **27–28**, Table 4). These are two substituents that we hypothesized to influence the overall chemicophysical properties of our compounds by mitigating the metabolic reactivity of the alkyl chain on the amine, which appeared to be a metabolic soft spot.

**Table 4:**
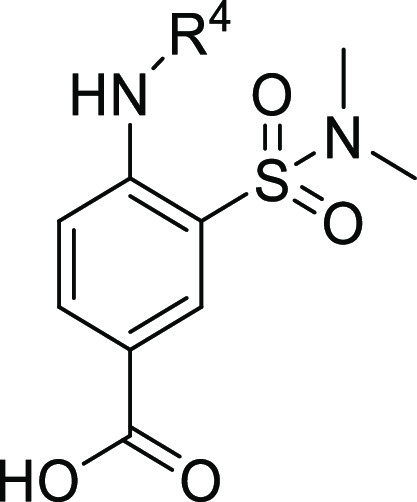

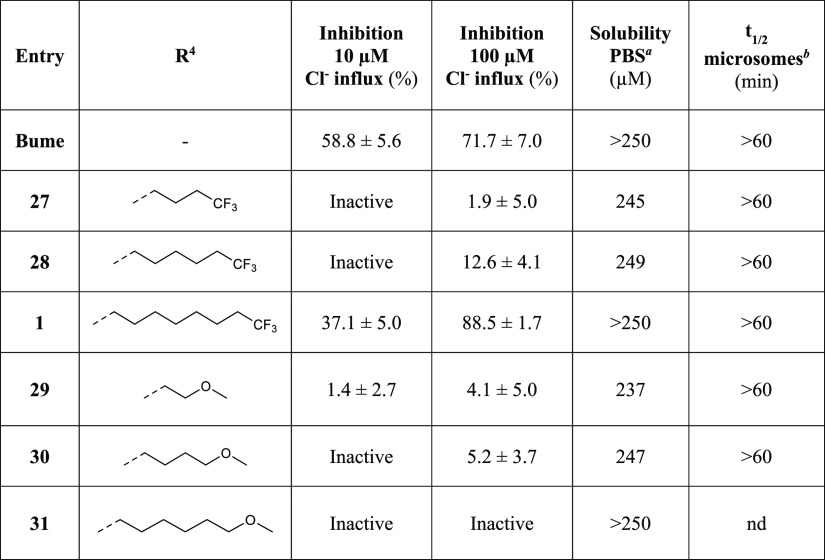
Inhibitory Activity of Terminal Alkyl-Substituted Derivatives and *In Vitro* Metabolic Stability and Solubility of Selected Derivatives

a Aqueous kinetic solubility of compounds from a 10 mM DMSO solution in PBS at pH 7.4. Target concentration is 250 μM (final DMSO 2.5%).

b Metabolic stability in mouse liver homogenates. Compounds were incubated at 5 μM (final DMSO 0.1%).

Among the synthesized derivatives, we observed that the insertion of the terminal trifluoromethyl group in **27** resulted in an inactive compound. However, **27** showed enhanced metabolic half-life, over 60 min. In analogy to what we observed with the alkyl chain derivatives **15–26** (Table 3), also here the elongation of the chain length from 6 to 8 carbon atoms (i.e., compound **28** vs **1**) resulted in an increase in activity (Table 4), whereas compound **28** displayed lower activity when compared to its nonfluorinated counterpart **24** (19.8 ± 5.2% at 100 μM). Importantly, the presence of the trifluoromethyl group substantially enhanced the activity of the *n*-octyl derivative **1**. At 100 μM, compound **1** displayed a superior NKCC1 inhibitory activity (88.5 ± 11.7%), also when compared to bumetanide (71.7 ± 7%). This was probably due to more extended lipophilic interactions of the trifluoromethyl group with the target. In addition, **1** displayed a substantially improved kinetic solubility (>250 μM) and half-life in mouse liver homogenates (>60 min).

The insertion of the terminal methyl ether led to an amelioration of drug-like properties (metabolic stability and solubility, Table 4). However, such a modification resulted in a dramatic drop of the inhibitory activity, as for the derivatives **29** and **30** (**29**, 10 μM = 1.4 ± 2.7% and 100 μM = 4.1 ± 5.0% and **30**, μM 10 = inactive and 100 μM = 5.2 ± 3.7%). Notably, also the derivative having a longer chain on the amine, **31**, did not show any NKCC1 inhibition, suggesting that the terminal methyl ether may disrupt a key interaction established by the alkyl chain with the transporter.

Thus, we considered the trifluoro *n*-octyl chain as a valuable modification for the improvement of activity and the overall drug-like profile. We decided to further characterize compound **1** for activity in neurons, which is a more relevant experimental setting for the development of compounds designed for the treatment of brain disorders.

### Selection of Lead Compound **1** (**ARN23746**)

We assessed the activity of **1** in neurons by the calcium (Ca^2+^) influx assay in immature primary cultures, which is an indirect measure of NKCC1 inhibition^5^ (Table 5). We also assessed bumetanide activity in the same neuronal assay for comparison. In line with the Cl^–^ influx assay, bumetanide displayed a reduction of Ca^2+^ uptake of 51.9 ± 2.3% at 10 μM and 54.7 ± 2.5% at 100 μM. Compound **1** showed the most potent inhibition, 45.7 ± 4.3% at 10 μM and 92.8 ± 1.9% at 100 μM, which is almost twofold better in comparison to bumetanide at 100 μM. We thus selected **1** as our best molecule for the further exploration and characterization of additional novel analogues. In particular, as the trifluoro *n*-octyl chain resulted to be pivotal for potency and metabolic stability in **1**, we conserved this structural motif throughout the design of the novel derivatives, in combination with either diverse sulfonamide substitutions in R^5^ (Table 6) or the insertion of substituents of different natures in position R^2^ (Table 7).

**Table 5:** Inhibitory Activity of Bumetanide and Compound **1** Evaluated in the Ca^2+^ Influx Assay

entry	inhibition 10 μM Ca^2+^ influx (%)	inhibition 100 μM Ca^2+^ influx (%)
bumetanide	51.9 ± 2.3	54.7 ± 2.5
**1**	45.7 ± 4.3	92.8 ± 1.9

**Table 6:**
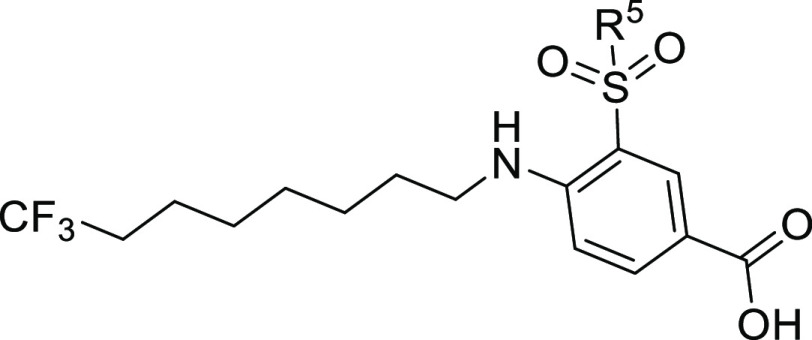

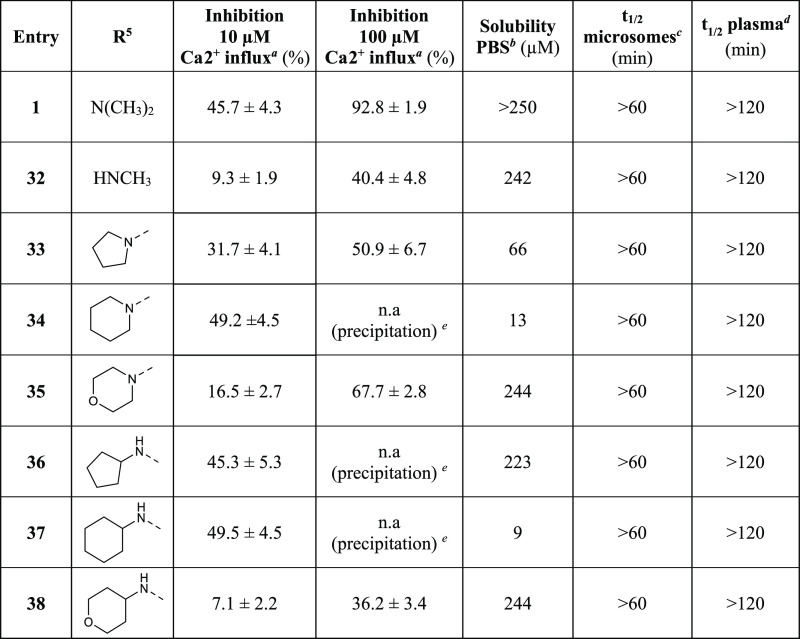
Inhibitory Activity, Metabolic and Plasmatic Stability, and Solubility of Analogues Modified at R^5^

a Inhibition percentage in Ca^2+^ influx assay in cultured neurons.

b Aqueous kinetic solubility of compounds from a 10 mM DMSO solution in PBS at pH 7.4. Target concentration is 250 μM (final DMSO 2.5%).

c Metabolic stability in mouse liver homogenates.

d Plasma stability in mouse plasma at 37 °C. Compounds were incubated at 5 μM (final DMSO 0.1%).

e Precipitation observed in the assay buffer.

**Table 7:**
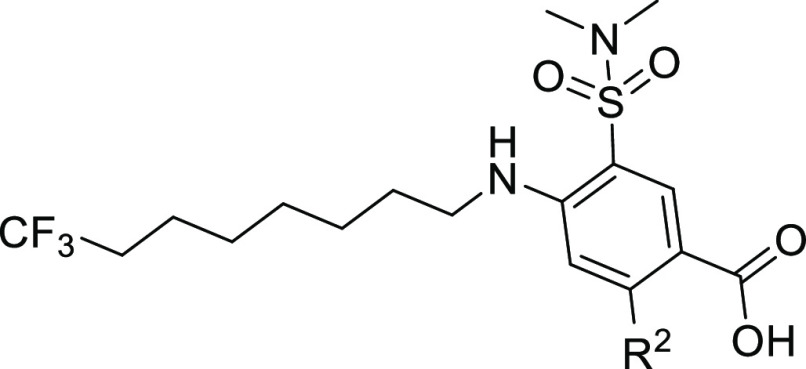

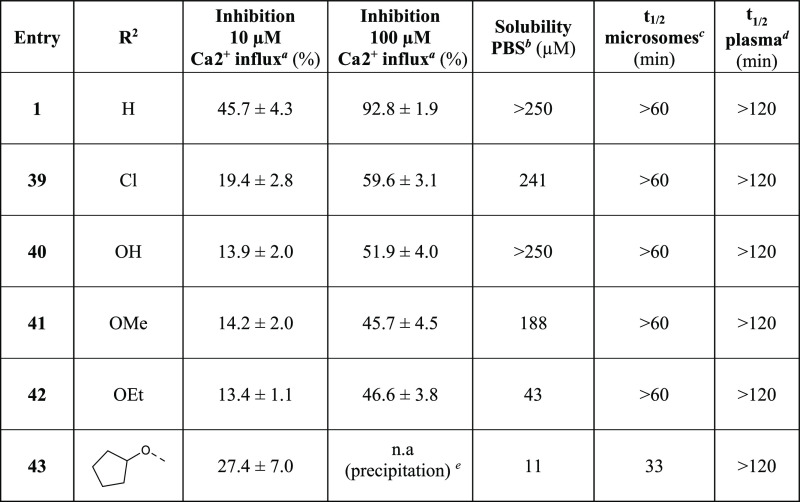
Inhibitory Activity, Metabolic and Plasmatic Stability, and Solubility of Analogues Modified at R^2^

a Inhibition percentage in Ca^2+^ influx assay in cultured neurons.

b Aqueous kinetic solubility of compounds from a 10 mM DMSO solution in PBS at pH 7.4. Target concentration is 250 μM (final DMSO 2.5%).

c Metabolic stability in mouse liver homogenates.

d Plasmatic stability in mouse plasma at 37 °C. Compounds were incubated at 5 μM (final DMSO 0.1%).

e Precipitation observed in the assay buffer.

### R^5^ Sulfonamide Evaluation

The analogue **32**, bearing an *N*-methyl sulfonamide, displayed a marked decrease in inhibitory activity (10 μM = 9.3 ± 1.9% and 100 μM = 40.4 ± 4.8%) in comparison to **1**. This was consistent with the other *N*-methyl analogues (Table 3). Interestingly, the insertion of a five-membered cyclic sulfonamide in analogue **33** was tolerated at 10 μM, although it resulted in a reduction in activity at 100 μM (50.9 ± 6.7%). Moreover, we did not observe a substantial difference in activity between the pyrrolidine **33** and the six-membered piperidine derivative **34**, at 10 μM. This suggests that the substituted sulfonamide may interact with the target in a site where the nitrogen atom forms a key H-bond acceptor interaction. This may orientate the group attached to the sulfonamide in a lipophilic pocket, with tolerance for bulkier groups compared to the dimethyl group of **1**. However, the introduction of the pyrrolidine and piperidine led to a dramatic decrease in the kinetic solubility of compounds **33** and **34** (**33** = 66 μM and **34** = 13 μM), while having no effect on the other measured properties such as metabolic and plasmatic stability (Table 6). For compound **34**, its low solubility allowed only to test it at 10 μM.

The polar six-membered morpholine derivative **35** retained activity at 10 μM (16.5 ± 2.7%), while showing good inhibitory activity at 100 μM (67.8 ± 2.8%; Table 6). In addition, the presence of the morpholine ring resulted also in a substantially higher solubility (244 μM) compared to the aliphatic piperidine **34** (13 μM). The effect of the substitutions of the two cycloalkyl derivatives cyclopentane **36** and cyclohexane **37** were comparable to one of the corresponding cyclic amines at 10 μM (**36** = 45.3 ± 5.3% and **37** = 49.5 ± 4.5%). This suggests that the nitrogen atom of the secondary sulfonamide may interact with the target in a similar manner to the one of **33** and **34**, thus likely serving as the H-bond acceptor upon binding. Strikingly, testing of the cyclopentane analogue **36** at 100 μM resulted in precipitation of the compound in the assay buffer despite good kinetic solubility in phosphate-buffered saline (PBS) (223 μM), whereas insertion of cyclohexane **37** resulted in markedly diminished kinetic solubility (9 μM), hampering their testing at high concentrations. Finally, the more polar tetrahydropyran **38** resulted in a substantial loss in activity at both the concentrations (10 μM = 7.1 ± 2.2% and 100 μM = 36.2 ± 3.4%). In summary, cyclic and cycloalkyl sulfonamides are tolerated in terms of activity but can have a drastic negative effect on solubility, which may be mitigated by heterocycles bearing polar atoms. However, an increase in polarity coincided with a loss of inhibitory activity.

### R^2^ Substituent Evaluation

To investigate the presence of an additional substituent anchored to the central aromatic core, we decided to explore the chemically accessible position 2 by testing diverse chemical moieties (Table 7). In detail, we evaluated the effect of a chlorine, a hydroxyl group, and diverse alkyl ethers. Irrespective of the nature of the substituent inserted, the R^2^-substituted analogues **39** and **40** returned lower activity when compared to **1** (Table 7). Moreover, these compounds retained a similar activity despite their substitution in R^2^. Indeed, the presence of the lipophilic chlorine atom in R^2^, as in **39**, resulted only in a slight increase in inhibitory activity at 100 μM (59.6 ± 3.1%), when compared to the hydrophilic hydroxyl group of **40** (51.8 ± 4.0%). On the other hand, compounds **39** and **40** displayed a comparable inhibitory activity at 10 μM (**39** = 19.4 ± 2.8% and **40** = 13.9 ± 2.0%). Replacement of the hydroxyl group of **40** with two short alkyl ethers as in **41** and **42** (Table 7) also resulted in a comparable inhibitory activity at 100 μM (**41** = 45.7 ± 4.7% and **42** = 46.6 ± 3.8%), although leading to a reduction in kinetic solubility (**41** = 188 μM and **42** = 43 μM), which is someway proportional to the length of the alkyl group. Moreover, when we inserted the bulk cyclopentane ether as in **43**, we observed inhibition levels similar to **40** at 10 μM (27.4 ± 7.0%). However, **43** suffered from a dramatic decrease in kinetic solubility (11 μM), which hampered its testing at 100 μM. In addition, **43** was also burdened by a remarkable drop in metabolic stability (33 min), indicating that saturated cycles in position 2 may be a metabolic soft spot. However, in terms of inhibitory activity, substituents in position R^2^ are quite tolerated, with the majority of these compounds that also displayed favorable drug-like properties.

### Modeling and Pharmacophore Hypotheses for NKCC1 Inhibition

**Figure 3:**
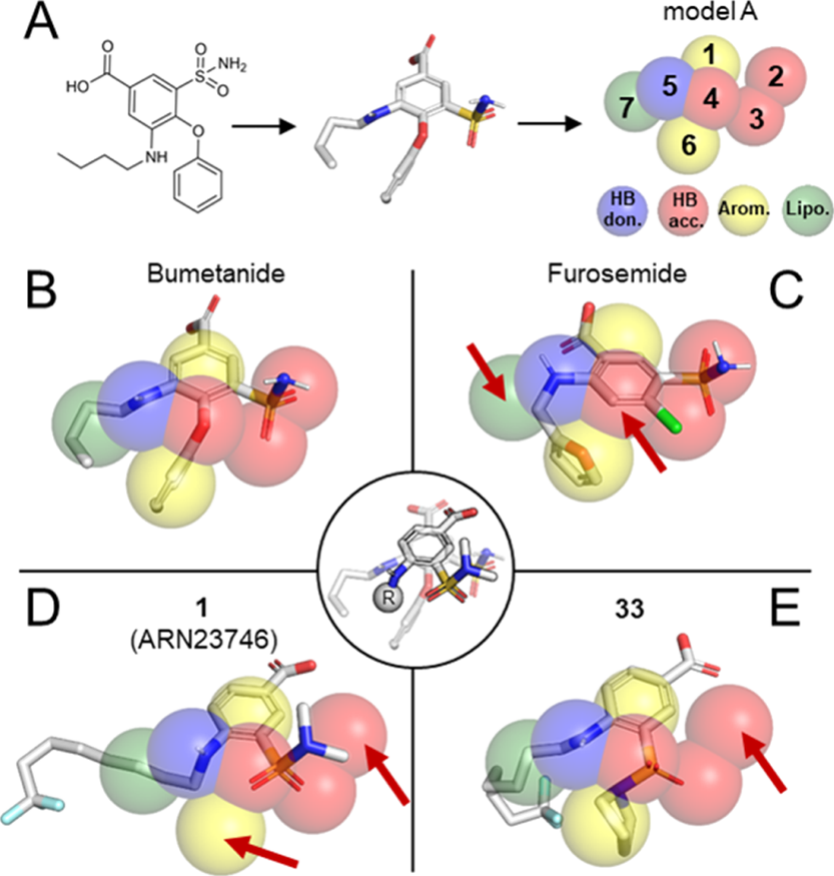
Pharmacophore model generation and fitting. (A) Identification of the low energy conformations of bumetanide (white sticks) and generation of the pharmacophore hypothesis (transparent spheres). (B–E) Overlap of bumetanide, furosemide, **1** (**ARN23746**), and **33** (containing a bulky pyrrolidine group, which is useful to appreciate spatial rotation of the sulfonamide) with the pharmacophore features. Red arrows highlight features’ mismatches between the compounds and the pharmacophore model. Furosemide is the less potent NKCC1 inhibitor, and it shows a different fit onto the model in comparison to other bumetanide derivatives. In the middle circle, the overlap of bumetanide (transparent sticks) and the main scaffold of our selective inhibitors (outlined sticks) is represented. Due to a slight rotation upon the aromatic plane, the fitting of our inhibitors differs from that of bumetanide derivatives.

In an effort to characterize the three-dimensional (3D) spatial arrangement for activity of our compounds, we sought to rationalize the SAR with a ligand-based computational approach, building upon the data provided by our experimental work.^5^ Thus, we first performed a force-field-based conformational search of the bumetanide’s structure to identify the most energetically favored conformations of its substituents, in the 3D space. We considered each substituent as a pharmacophore’s feature of bumetanide (i.e., HB donors, HB acceptors, and hydrophobic and aromatic groups). We initially built several pharmacophore hypotheses based on the bumetanide’s structure and conformers. Then, using Phase,^44,45^ we evaluated these pharmacophore models through the structural fitting of a set of NKCC1 inhibitors based on a 5-sulfamoyl benzoic acid scaffold (i.e., benzmetanide, piretanide, and furosemide). These inhibitors were ranked in each model according to the Phase score.^44,45^ As a result, we could identify a specific pharmacophore model (namely, model A; Figure 3A—see methods for details on features of model A) able to separate furosemide, the least active in our subset of NKCC1 inhibitors, from the other compounds. Specifically, benzmetanide, piretanide, and bumetanide returned phase scores of 2.22, 2.13, and 2.52, respectively. This is reflected by a good overlap with the 3D arrangement of the pharmacophore’s features of model A (Figures 3B and S1). On the other hand, we found that furosemide mismatched the 3D arrangement of the pharmacophore model A (phase score = 1.12). This was due to the poor fit of the HB acceptor in position 4 and the lipophilic group in 7 (Figure 3C). In other words, aware of the qualitative nature of our pharmacophore model built with a limited number of active compounds, we noted that our pharmacophore hypothesis was somehow able to discriminate the more potent NKCC1 inhibitors, from the least active furosemide.

Thus, we challenged this simple model with our congeneric series of novel 42 derivatives. As a result, all our compounds showed a good fit onto the pharmacophore model A (see Table S1, for scoring). Indeed, our compounds matched the HB acceptor in position 4 and the hydrophobic group in 7, suggesting that the presence of these two structural features helped increasing the affinity for NKCC1 (Figure 3D,E). In agreement with these findings, our SAR study showed that the more hydrophobic is the feature in 7, the higher is the inhibition of NKCC1, reaching its peak with the lead compound **1** (92.8 ± 1.9% inhibition at 100 μM; phase score = 1.47). However, according to this model, our compounds show a different pharmacophore fit also compared to bumetanide and its two derivatives benzmetanide and piretanide. In particular, our new scaffold does not match the HB acceptor in position 4 through a phenoxy moiety (Figure 3D,E), as most active bumetanide’s derivatives do (Figures 3B and S1). Instead, our compounds satisfy this match through a rotation of ∼18.6° of the entire compound onto its aromatic ring (Figure 3, middle circle). This rotation allows the sulfonamide’s oxygens to be aligned with both the HB acceptors in position 4 and 3 (Figure 3D,E). Importantly, such rotation did not alter the positioning of the carboxylic moiety. Position 2 of the pharmacophore model remained unmatched in our new scaffold (Figure 3D,E). We note that the presence of a HB acceptor in position 2 is conserved in all the unselective bumetanide derivatives, while it is absent in our selective NKCC1 inhibitors, suggesting that such a feature could be specific for NKCC2 binding and inhibition. Moreover, position 6 is unmatched in our scaffold in the presence of small sulfonamide’s substituents (Figure 3D). However, introducing bulky substituents on the sulfonamide leads to a rotation of this group, which reverses its orientation, matching the position 6 of the pharmacophore model, as shown by the fit of **33** (Figure 3E). Such a structural flexibility of the sulfonamide group may justify the tolerance for bulky sulfonamide’s substituents observed in our SAR study.

Taken together, these multiple structural alignments over our pharmacophore Phase models distinguished fairly well, although only qualitatively, more potent versus less potent NKCC1 inhibitors, providing also structural hints for NKCC1 selectivity.

### As a Valuable Backup/Follow-Up of 1, Compound 40 Is Able to Rescue Memory Deficits in a DS Mouse Model, While Having No Diuretic Effect

**Figure 4:**
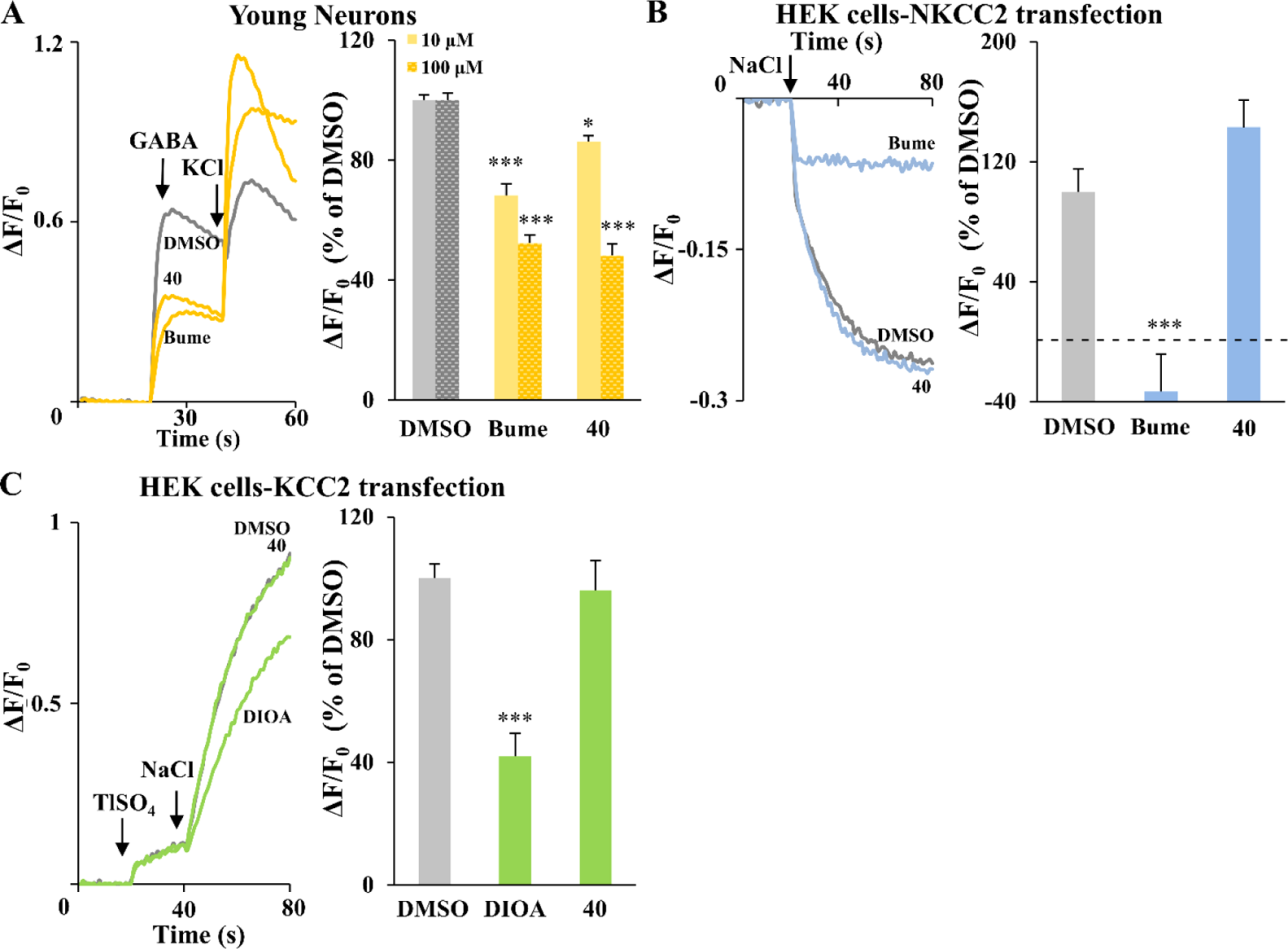
*In vitro* activity and selectivity of compound **40** in cell-based assays. (A) Left, example traces obtained in the Ca^2+^ influx assay on 3 days *in vitro* (DIV) neuronal cultures for each compound (100 μM). The arrows indicate the addition of GABA (100 μM) and KCl (90 mM). Right, quantification of the effect of the indicated compounds (10 and 100 μM) in experiments as those on the left. Data are represented as a percentage of the respective control DMSO. Data represent mean ± SEM from three independent experiments. 10 μM: Kruskal–Wallis one-way ANOVA on ranks, *H* = 24.747, DF = 2, *P* < 0.001, Dunn’s post hoc test, *P < 0.05, ***P < 0.001; 100 μM: Kruskal–Wallis one-way ANOVA on ranks, *H* = 23.646, DF = 2, *P* < 0.001, Dunn’s post hoc test, ***P < 0.001. (B) Left, example traces obtained in the Cl^–^ influx assay on NKCC2-transfected HEK293 cells for each compound (10 μM). The arrow indicates the addition of NaCl (74 mM) to initiate the NKCC1-mediated Cl^–^ influx. Right, quantification of the NKCC2 inhibitory activity in experiments as those on the left. Data are presented as a percentage of the respective control DMSO. Data represent mean ± SEM from four independent experiments (one-way ANOVA, *F*(2, 48) = 21.161, *P* < 0.001, Dunnett’s post hoc test, ***P < 0.001). (C) Left, example traces obtained in the Tl influx assay on KCC2-transfected HEK293 cells for each compound (10 μM). The arrows indicate the addition of Tl_2_SO_4_ (2 mM) and NaCl (74 mM). Right, quantification of the KCC2 inhibitory activity in experiments as those on the left. Data are presented as a percentage of the respective control DMSO. Data represent mean ± SEM from three independent experiments (one-way ANOVA, *F*(2, 37) = 19.194, *P* < 0.001, Dunnett’s post hoc test, ***P < 0.001).

We have previously reported **1** (**ARN23746**) as a lead compound with *in vivo* efficacy in rescuing cognitive impairment in a DS mouse model and social deficits and repetitive behaviors in an autism mouse model.^5^ Here, we report on the characterization of another highly promising compound that may serve as a valuable backup/follow-up of the lead **1**. Compound **40** (**ARN24092**) showed significant dose-dependent inhibition of NKCC1 in the Ca^2+^ influx assay (Figures 4A and S2).^5^ Moreover, as for compound **1**,^5^ and in contrast to bumetanide or DIOA, at 10 μM, compound **40** did not show any significant inhibition of NKCC2 in the Cl^–^ influx assay (Figure 4B) nor inhibition of KCC2 in the thallium (Tl) influx assay (Figure 4C).^5^ Finally, compound **40** showed great drug-like properties in terms of solubility and metabolic stability (Table 7).

**Figure 5:**
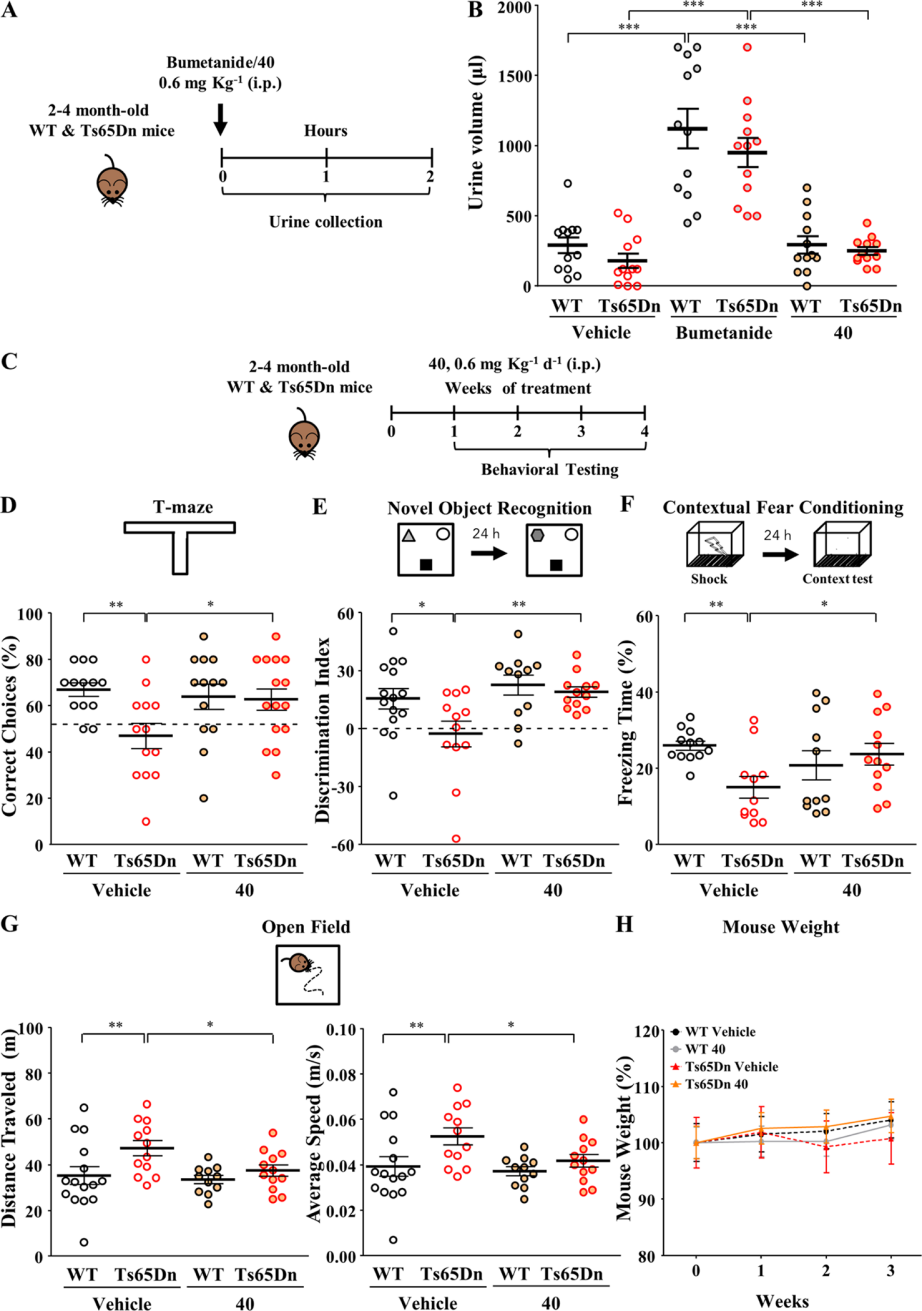
Assessment of *in vivo* diuresis and efficacy of compound **40** in Ts65Dn mice. (A) Schematic representation the experimental protocol for the treatment of adult WT and Ts65Dn mice and assessment of the diuretic effect. (B) Quantification of the mean ± SEM and single animal cases of the urine volume collected for 2 h after mice were treated with the indicated drugs (two-way ANOVA on Ranks, *F*_treatment_(2, 66) = 54.315, *P* < 0.001, Tukey’s post hoc test, ***P < 0.001). (C) Schematic representing the experimental protocol for the treatment of adult WT and Ts65Dn mice with **40** for *in vivo* efficacy assessment of memory and hyperactivity in DS mice. (D) Top, schematic representation of the T-maze test. Bottom, quantification of the mean ± SEM and single animal cases of correct choices in mice treated with the indicated drugs (two-way ANOVA, *F*_interaction_(1, 50) = 4.036, *P* = 0.050, Tukey’s post hoc test, *P < 0.05, **P < 0.01). (E) Top, schematic representation of the novel-object recognition test. Bottom, quantification of the mean ± SEM and single animal cases of the discrimination index in mice treated with the indicated drugs (two-way ANOVA, *F*_treatment_(1, 46) = 7.640, *P* = 0.008, Tukey’s post hoc test, *P < 0.05, **P < 0.01). (F) Top, schematic representation of the CFC test. Bottom, quantification of the mean ± SEM and single animal cases of the freezing response in mice treated with the indicated drugs (two-way ANOVA, *F*_interaction_(1, 42) = 6.209, *P* = 0.017, Tukey’s post hoc test, *P < 0.05, **P < 0.01). (G) Top, schematic representation of the open field test. Bottom left, quantification of the mean ± SEM and single animal cases of the distance traveled during the test in mice treated with the indicated drugs (two-way ANOVA, *F*_genotype_(1, 46) = 6.206, *P* = 0.016, Tukey’s post hoc test, *P < 0.05, **P < 0.01). Bottom right, quantification of the mean ± SEM and single animal cases of the average walking speed in mice treated with the indicated drugs (two-way ANOVA, *F*_genotype_(1, 46) = 6.206, *P* = 0.016, Tukey’s post hoc test, *P < 0.05, **P < 0.01). (H) Quantification of the body weight of WT and Ts65Dn mice across the 3 weeks of treatment with the indicated drugs.

Thus, we selected **40** for *in vivo* efficacy studies. Based on our recently published work on **1**,^5^ we decided to assess *in vivo* efficacy of **40** in a mouse model of DS. DS is a neurodevelopmental disorder caused by the triplication of human chromosome 21, and it is the leading cause of genetic intellectual disability. First, we assessed the diuretic effect of **40** in adult (2–4 months old) Ts65Dn mice and their WT littermates, using bumetanide as the positive control, as previously performed.^5^ Compound **40** and bumetanide were administered via intraperitoneal (ip) injection at a dosage of 0.6 mg kg^–1^. We selected an *in vivo* dosage threefold higher than the one used for **1** (0.2 mg kg^–1^)^5^ because **40** showed an *in vitro* NKCC1 inhibition in Ca^2+^ influx assay approximately threefold lower than **ARN23746** at 10 μM (compare data of **1** and **40** in Table 7). After the treatment, mice were placed in metabolic cages where urine was collected for the following 2 h (Figure 5A). As expected, bumetanide administration significantly increased the urine volume in both WT and Ts65Dn mice, when compared with vehicle-treated mice (Figure 5B). Conversely, treatment with compound **40** did not induce any significant diuresis both in WT and in Ts65Dn mice, when compared to the vehicle-treated mice (Figure 5B).

We then evaluated the efficacy of **40** in rescuing memory impairment in Ts65Dn mice, as previously performed with **1**.^5^ In particular, we investigated both the short-term hippocampus-dependent working memory and the long-term hippocampus-dependent memory after a chronic (7–21 days) systemic treatment with **40** (ip, 0.6 mg kg^–1^, daily, Figure 5C). We found that **40** fully restored the short-term working memory of Ts65Dn mice in the T-maze test, as assessed by the rescue of the number of correct choices (Figure 5D). Moreover, in the novel object recognition (NOR) task, **40** completely rescued the poor novel-discrimination ability of Ts65Dn mice (Figure 5E). The effect of **40** in the NOR tests was not due to object preference (Table S2) or to alterations in total object exploration (Table S2). Finally, treatment with **40** completely restored associative memory in Ts65Dn mice in the contextual fear-conditioning (CFC) test, as assessed by the rescue of the freezing response induced after re-exposure to the training context 24 h after conditioning (Figure 5F). The rescue of the poor freezing response was not due to altered sensitivity to shock or by changes in nonassociative freezing (Table S2). Interestingly, we found that **40** rescued also the hyperactivity of Ts65Dn mice, expressed as the distance traveled and average walking speed during the open-field free exploration of a squared arena (corresponding to the first day of habituation to the arena used for the NOR test). Notably, 3 weeks of daily treatment with **40** did not affect the general health of the mice, as evaluated by daily hands-on examination and did not affect the animal body weight measures (Figure 5H).

## Chemistry

### Synthesis of Series I and Series II Compounds

**Scheme 1:**
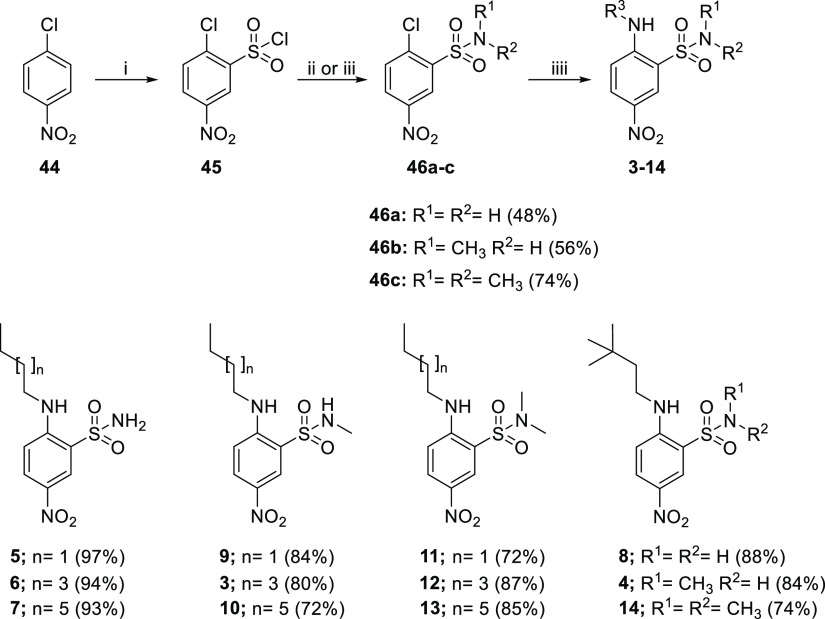
Reagents and conditions: (i) HSO_3_Cl, 120 °C, 46%; (ii) NH_4_OH, THF, 0 °C to rt, 48%; (iii) amine hydrochloride, TEA, DCM, 0 °C to rt, 58–74%; and (iii) amine, toluene, 100 °C, 72–97%.

The synthesis of 2-amino-5-nitro-benzene-sulfonamide derivatives of Series I was initially undertaken by regioselective electrophilic aromatic substitution of commercial 1-chloro-4-nitro-benzene **44** with chlorosulfonic acid (Scheme 1), which afforded intermediate **45** in a 46% yield. Then, substitution of the chlorosulfonyl group of **45** was approached via two different methodologies to access the target sulfonamides **46a–e**. The use of aqueous ammonia afforded the primary sulfonamide **46a** in good yield (48%). Alternatively, alkylated sulfonamides **46b–c** were obtained in good yields (56–74%) using methylamine or dimethylamine hydrochloride in the presence of two equivalents of triethylamine. Finally, nucleophilic aromatic substitution with the proper alkyl amines occurred efficiently to afford the target compounds **3–14**.

**Scheme 2:**
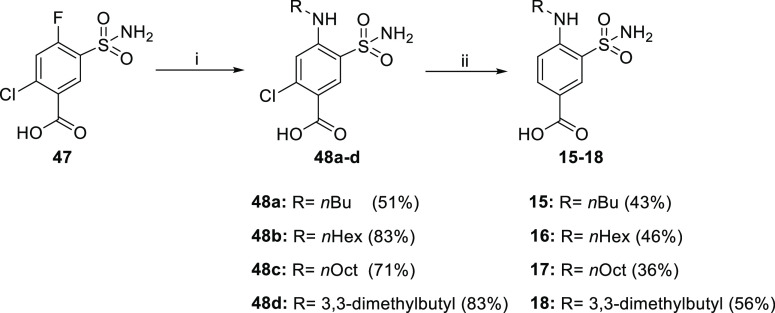
Reagents and conditions: (i) amine, 80–100 °C and (ii) HCOONH_4_, Pd(OH)_2_, MeOH, Ar, 80 °C.

Synthesis of 4-amino-3-sulfamoyl-benzoic acid compounds of Series II (Scheme 2) was achieved with the first step of nucleophilic aromatic substitution with the proper amines of commercial 2-chloro-4-fluoro-5-sulfamoylbenzoic acid **47**. Substitutions were run in neat amine, affording compounds **48a–d** in good yields (51–83%). Subsequently, dehalogenation of **48a–d** was performed via palladium-catalysed reduction with ammonium formate as the hydrogen source to afford target compounds **15–18**.

**Scheme 3:**
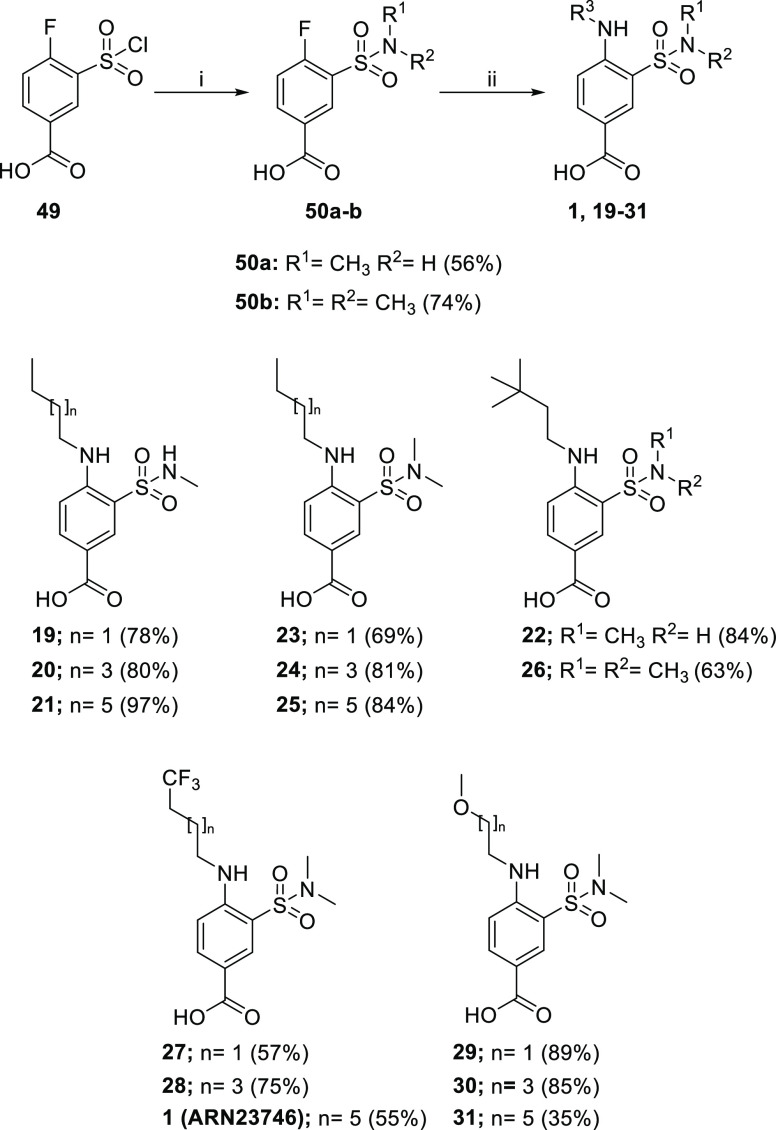
Reagents and conditions: (i) methylamine or dimethylamine (1 M in THF), THF 0 °C to rt and (ii) amine, TEA, 1,4-dioxane, 100 °C.

Derivatives **1** and **19–31** (Scheme 3) were obtained by the first substitution of 3-(chlorosulfonyl)-4-fluorobenzoic acid **49** with an excess of methylamine or dimethylamine to access compounds **50a–b** in good yields (56–74%). Finally, these intermediates were refluxed in 1,4-dioxane with the proper alkyl amines to afford the target compounds in high yields.

**Scheme 4:**
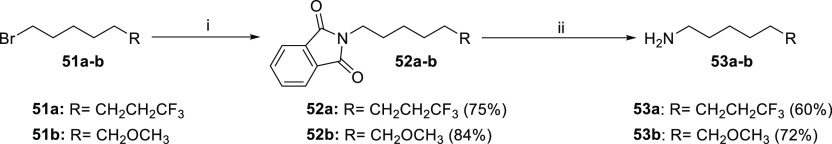
Reagents and conditions: (i) potassium phtalimide, DMF, rt and (ii) hydrazine hydrate, EtOH, reflux.

Noncommercial substituted *n*-octyl amines **53a–b** for the synthesis of **1** and **31** were, respectively, prepared via a two-step procedure starting from Gabriel reaction on bromides **51a–b** (Scheme 4). *N*-alkyl phthalimides **52a-b** were then cleaved with hydrazine hydrate to afford the pure amines.

**Scheme 5:**
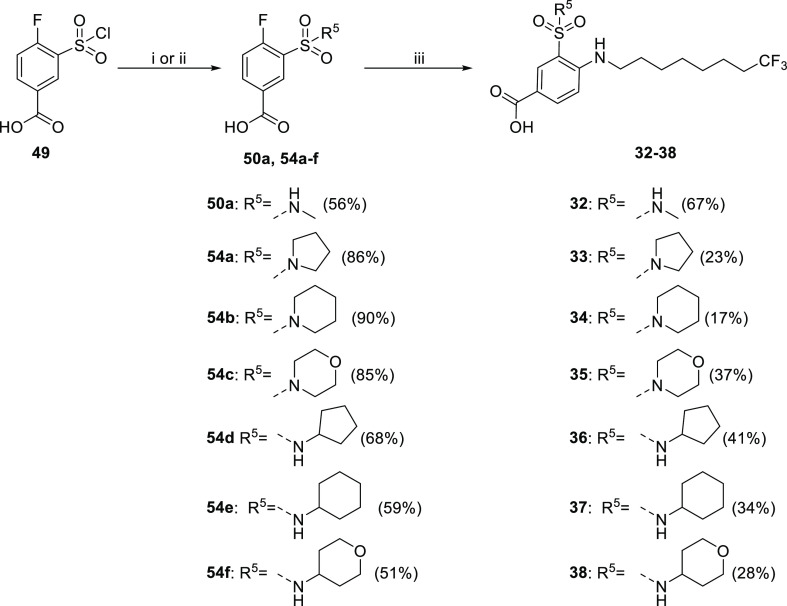
Reagents and conditions: (i) amine, THF, 0 °C to rt, (ii) amine hydrochloride, DIPEA, THF, 0 °C to rt; and (iii) amine **53a**, TEA, 1,4-dioxane, 100 °C.

R^5^-substituted sulfonamide derivatives **32–38** were prepared via substitution of chlorosulfonyl of **49**, applying two different methodologies depending on the amine used (Scheme 5). Commercially available amines have been used as a free base without the addition of any base, while hydrochloride amines were reacted with **49** in the presence of DIPEA. The substitution reaction afforded the selected N-substituted sulfonamide **50a** and **54a–f** building blocks in good yields (51–90%). Interestingly, cyclic secondary amines gave substantially higher yields when compared to the primary cycloalkyl amines. Finally, nucleophilic aromatic substitution with amine **53a** yielded the target compounds **32–38**.

**Scheme 6:**
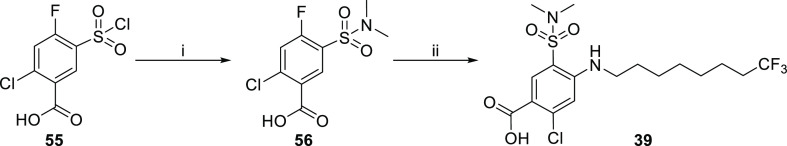
Reagents and conditions: (i) dimethylamine, DIPEA, THF, 0 °C to rt, 41% and (ii) amine **53a**, TEA, 1,4-dioxane, 100 °C, 88%.

R^2^-substituted 2-chloro derivative **39** (Scheme 6) was prepared by reaction of commercial 2-chloro-5-chlorosulfonyl-4-fluoro-benzoic acid **55** with dimethylamine in the presence of DIPEA, affording intermediate **56** was isolated in a 41% yield after chromatographic purification. Consequently, the highly activated position 4 reacted efficiently in the nucleophilic aromatic substitution step with amine **53a** to afford compound **39** in high yield.

**Scheme 7:**
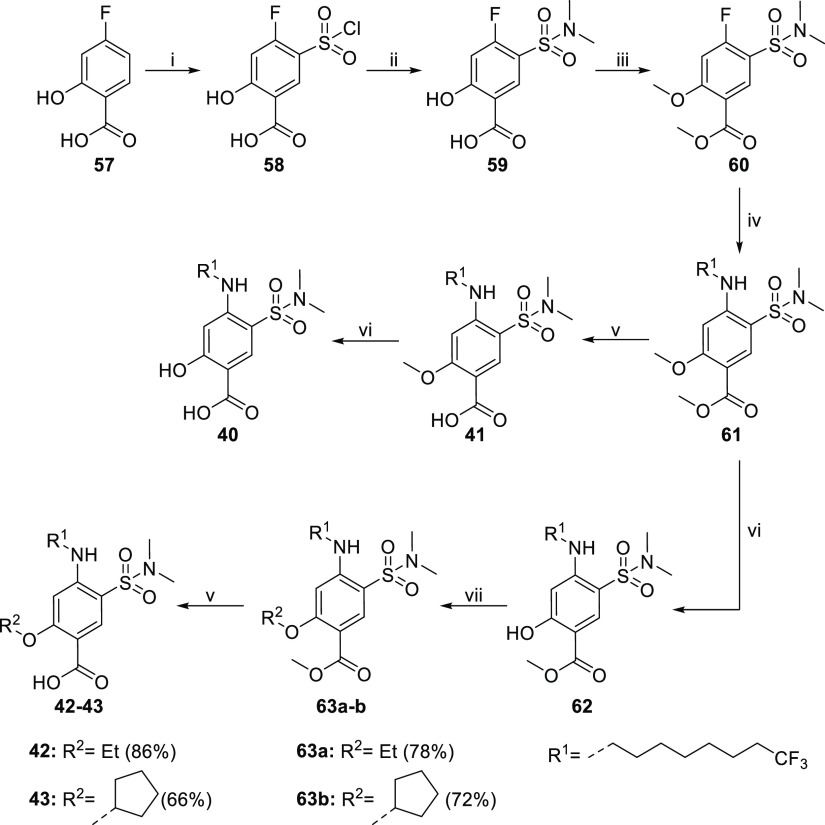
Reagents and conditions: (i) HOS_3_Cl, 0 to 120 °C, 35%; (ii) dimethylamine, DIPEA, THF, 0 °C, 70%; (iii) TMS-CHN_2_, DCM/MeOH 80:20, 0 °C to rt, 92%; (iv) amine **52a**, TEA, 1,4-dioxane, 100 °C, 84%; (v) LiOH aq, THF, rt, 72–86%; (vi) BBr_3_, DCM, 0 °C to rt, 73–83%; and (vii) alkyl halide, acetonitrile, K_2_CO_3_, 80 °C, 72–78%.

As depicted in Scheme 7, synthesis of R^2^ hydroxyl and ether derivatives was initiated by performing electrophilic aromatic substitution on commercial 4-fluorosalicylic acid **57** with chlorosulfonic acid, which yielded 5-chlorosulfonyl derivate **58** in a regioselective fashion. Subsequent reaction of **58** with dimethylamine in the presence of DIPEA afforded the 5-dimethyl sulfonamide intermediate **59** in a nice 70% yield. Both phenol and carboxylic acid groups of **59** were protected by methylation with an excess of TMS-CHN_2_, affording dimethylated product **60** in an excellent 92% yield. Nucleophilic aromatic substitution of **60** with amine **53a** efficiently yielded compound **61**. The two methyl groups were then removed by orthogonal deprotection to access different products. Target compounds **40** and **41** were obtained in the first route by generation of the 2-methoxy analogue **41** via methyl ester hydrolysis with lithium hydroxide. Then, demethylation of the phenol with boron tribromide afforded the 2-hydroxy analogue **40** in a high yield. Conversely, intermediate 2-hydroxy methyl ester **62** was generated by the first demethylation of the methyl ether group of **61** with boron tribromide, unmasking the phenol group that was then alkylated to generate intermediates **63a–b**. Final ester hydrolysis of direct precursors **63a–b** with aqueous lithium hydroxide afforded target compounds **42** and **43** in a high yield.

## Concluding Remarks and Future Work

Here, we have summarized our drug discovery effort for the discovery of **1** and the expansion of this new chemical class of selective NKCC1 inhibitors. Based on the hit compounds emerged from a virtual screening campaign, we synthesized and tested 12 new 2-amino-5-nitro-benzenesulfonamides (Series I). None of them exhibited enhanced potency in NKCC1 inhibition in comparison to the hit compounds (**3** and **4**). Conversely, the 4-amino-3-sulfamoyl-benzoic acid derivatives (Series II) emerged as the best class in terms of potency, when compared to nitrobenzenes. In particular, we performed numerous manipulations on this series, also inspired by the investigation of the specific effect of bumetanide substituents, reported in our previous work.^5^ The benzoic acid derivatives bearing the *n*-octyl chain showed enhanced potency when compared to the shorter chain derivatives, particularly when combined with dimethylation of the sulfonamide (as for compound **25**). Insertion of the trifluoromethyl group in compound **1** (**ARN23746**) at the terminal carbon of the *n*-octyl chain strongly increased the potency, which reached levels comparable to those of bumetanide. Then, we also confirmed potency of **1** in cultured immature neurons. Next, we further characterized the chemical class represented by our lead compound **1**. We defined the effect of the trifluoro *n*-octyl chain combined with modifications of the other substituents on the aromatic ring. Manipulations of the sulfonamide in position R^5^ preserved the compound activity, although the introduction of cyclic and cycloalkyl sulfonamides had a detrimental effect on solubility. Our data also suggest the further exploitability of the chemically accessible position 2 on the aromatic ring for the design of additional active analogues. In this regard, the recent resolution of the NKCC1^46,47^ and KCC1, KCC2, KCC3, and KCC4^48−51^ cryo-EM structures has marked an important milestone in the understanding of the structure and mechanism of action of these cation-chloride cotransporter proteins.^52^ The data reported in these recent studies open now the way to structure-based drug design, toward the development of additional specific inhibitors. In particular, we (as well as other researchers) are currently performing molecular simulations of such challenging protein systems to better define their exact mechanism for ion transportation and possible ways for their modulation by small molecules. This may thus lead to further optimization of this chemical class and its overall profile to target the central nervous system (CNS).

Importantly, we also report here the compound **40** (**ARN24092**), which we demonstrate to be able to rescue cognitive impairment in four diverse tasks *in vivo*, while not exerting any diuretic effect in the treated animals. This compound is therefore an optimal first backup/follow-up compound of our lead **1** (**ARN23746**), as others will certainly follow and be reported in due course. Along these lines, further investigations on the mechanism of action *in vivo* will be performed (e.g., electrophysiology, NKCC1 knockdown neurons, and chloride imaging).

In conclusion, our data present a new chemical class of selective NKCC1 inhibitors as a solid basis for advanced preclinical studies and drug development toward unprecedented sustainable therapeutic treatments in DS and possibly several other neurological conditions characterized by defective chloride homeostasis.

## Experimental Section

### Chemistry

#### General Considerations

All the commercially available reagents and solvents were used as purchased from vendors without further purification. Dry solvents were purchased from Sigma-Aldrich. Automated column chromatography purifications were carried out using a Teledyne ISCO apparatus (CombiFlash Rf) with prepacked silica gel or basic alumina columns of different sizes (from 4 g up to 40 g) and mixtures of increasing polarity of cyclohexane and ethyl acetate (EtOAc), cyclohexane or dicloromethane (DCM), and methanol (MeOH). NMR experiments were run on a Bruker AVANCE III 400 system (400.13 MHz for ^1^H and 100.62 MHz for 13C), equipped with a BBI probe and *Z*-gradients, and Bruker FT NMR AVANCE III 600 MHz spectrometer equipped with a 5 mm CryoProbe QCI ^1^H/^19^F–^13^C/^15^N–D quadruple resonance, a shielded *Z*-gradient coil and the automatic sample changer SampleJet NMR system (600 MHz for 1H, 151 MHz for ^13^C, and 565 MHz for ^19^F). Spectra were acquired at 300 K, using deuterated dimethylsulfoxide (DMSO-*d*_6_) or deuterated chloroform (CDCl_3_) as solvents. For ^1^H NMR, data are reported as follows: chemical shift, multiplicity (s = singlet, d = doublet, dd = double of doublets, t = triplet, q = quartet, h = sextet, and m = multiplet), coupling constants (Hz), and integration. UPLC/MS analyses were run on a Waters ACQUITY UPLC/MS system consisting of a SQD (single quadrupole detector) mass spectrometer equipped with an electrospray ionization interface and a photodiode array detector. The PDA range was 210–400 nm. Analyses were performed on an ACQUITY UPLC BEH C18 column (100 × 2.1 mmID, particle size 1.7 μm) with a VanGuard BEH C18 precolumn (5 × 2.1 mmID, particle size 1.7 μm). The mobile phase was 10 mM NH_4_OAc in H_2_O at pH 5 adjusted with CH_3_COOH (A) and 10 mM NH_4_OAc in CH_3_CN–H_2_O (95:5) at pH 5.0. Two types of gradients were applied depending on the analysis, gradient 1 (5 to 95% mobile phase B in 3 min) or gradient 2 (50 to 100% mobile phase B in 3 min). Electrospray ionization in positive and negative modes was applied. High-resolution mass spectrometry (HRMS) was carried out on a Waters Synapt G2 quadrupole-Tof instrument equipped with an ESI ion source. The analyses were carried out on an ACQUITY UPLC BEH C18 column (50 × 2.1 mmID, particle size 1.7 μm), using H_2_O + 0.1% formic acid (A) and MeCN +0.1% formic acid as the mobile phase. ESI was applied in positive and negative modes. All final compounds displayed ≥95% purity by UPLC/MS analysis with the exception of compounds **15** (92%) and **16** (90%).

#### General Procedure 1: Synthesis of Sulfonamides 46b-C

To an ice-cold solution of the proper amine hydrochloride (1.0 mmol) and triethylamine (2 mmol) in DCM (1.0 mL) was added intermediate **45** (1 mmol) dissolved in DCM (1.5 mL), and the reaction mixture was stirred at room temperature for 1 h. At reaction completion, the reaction crude was diluted with DCM (20 mL) and washed with an NH_4_Cl saturated solution (20 mL) and the aqueous layer was extracted twice with DCM (2 × 20 mL). The combined organic layers were dried over Na_2_SO_4_ and concentrated to dryness at low pressure. Purification by silica gel flash chromatography afforded the pure titled compounds.

#### General Procedure 2: General Nucleophilic Aromatic Substitution Procedure A

A suspension of intermediates **46a–c** (1 mmol) and the appropriate amine (5 mmol) was stirred in dry toluene (0.7 mL) under an argon atmosphere at 100 °C for 1 h. After reaction completion, the mixture was evaporated to dryness at low pressure. The dry residue was treated with water (10 mL) and extracted with EtOAc (10 mL). The organic layer was dried over Na_2_SO_4_ and concentrated to dryness at low pressure. Purification by silica gel flash chromatography afforded the pure titled compounds.

#### General Procedure 3: General Nucleophilic Aromatic Substitution Procedure B

A suspension of commercial 2-chloro-4-fluoro-5-sulfamoyl-benzoic acid **47** (1 mmol) and the appropriate amine (5 mmol) in dry toluene (0.7 mL) was stirred under an argon atmosphere at 100 °C for 1 h. After reaction completion, the mixture was evaporated to dryness at low pressure and the residue was treated with a saturated NH_4_Cl aqueous solution (15 mL) and extracted twice with EtOAc (2 × 15 mL). The combined organic layers were dried over Na_2_SO_4_ and concentrated to dryness at low pressure. Trituration in cyclohexane afforded finally the pure titled compounds.

#### General Procedure 4: General Dehalogenation Procedure

Under an argon atmosphere, to a suspension of the proper 4-amino-2-chloro-5-sulfamoyl-benzoic acid **48a–d** (1 mmol) and palladium hydroxide on carbon (20 wt %) in dry methanol (20 mL) was added ammonium formate (4 mmol), and the reaction mixture was stirred at reflux temperature for 1 h. After reaction completion, the crude was filtered through a Celite coarse patch and the filtrate concentrated to dryness at low pressure. The dry residue was diluted in EtOAc (10 mL) and washed with a saturated NH_4_Cl solution (10 mL). The organic layer was dried over Na_2_SO_4_ and concentrated to dryness at low pressure. Trituration in cyclohexane afforded finally the pure titled compounds.

#### General Procedure 5: Synthesis of Sulfonamides **50a–b**

4-Fluoro-3-chlorosulfonyl-benzoic acid **49** (1 mmol) dissolved in 1.5 mL of tetrahydrofuran (THF) was added dropwise to 8 mL of an ice-cold solution of the proper amine (2 mmol) in THF and stirred for 30 min at rt. At reaction completion, the reaction mixture was evaporated to dryness. The dry residue was dissolved in water and treated with 2 N HCl until it reached pH 3. The resulting precipitated product was filtered and rinsed with water to afford the pure titled compounds.

#### General Procedure 6: Nucleophilic Aromatic Substitution Procedure C for the Synthesis of Compounds **1**, **19–31**, **32–39**, and **61**

A suspension of intermediates **50a–b**, **54a–f**, **56**, or **60** (1 mmol) and the appropriate amine (2 mmol) in dry 1,4-dioxane (3 mL) was stirred under an argon atmosphere at 100 °C for 16 h. After reaction completion, the mixture was evaporated to dryness at low pressure and the residue was treated with a saturated NH_4_Cl aqueous solution (15 mL) and extracted twice with EtOAc (2 × 15 mL). The combined organic layers were dried over Na_2_SO_4_ and concentrated to dryness at low pressure. Purification by silica gel flash chromatography with CH_2_Cl_2_/MeOH followed by trituration with a suitable solvent (cyclohexane or diethyl ether) and then afforded the pure title compounds.

#### General Procedure 7: Synthesis of Intermediates **52a–b**

A suspension of potassium phthalimide (1 mmol) and the appropriate alkyl bromide **51a–b** (1.2 mmol) in dry *N*,*N*-dimethylformamide (DMF, 3.5 mL) was stirred at room temperature for 16 h. After reaction completion, the mixture was diluted with water (35 mL) and extracted with EtOAc (35 mL). The organic layer was dried over Na_2_SO_4_ and concentrated to dryness at low pressure. Purification by silica gel flash chromatography finally afforded the pure titled compounds.

#### General Procedure 8: Synthesis of Amines **53a–b**

The corresponding intermediate **52a–b** (1 mmol) was refluxed in absolute ethanol (4 mL) with hydrazine hydrate (1.5 mmol) for 4 h. At reaction completion, the reaction mixture was cooled at room temperature and the resulting precipitated solid was filtered. The solid was washed with ethanol, and the filtrate concentrated to dryness at low pressure. Purification by basic alumina flash chromatography finally afforded the pure titled amines.

#### General Procedure 9: Synthesis of Sulfonamides **54a–f**

4-Fluoro-3-chlorosulfonyl-benzoic acid **49** (1 mmol) dissolved in 2 mL of THF was added dropwise to 8 mL of an ice-cold solution of the proper amine (3 mmol) in THF and stirred for 1 h at rt. At reaction completion, the reaction mixture was evaporated to dryness and the residue was treated with water and HCl. The precipitated product was filtered and rinsed with water to afford the pure titled compounds.

##### 2-Chloro-5-nitro-benzenesulfonyl Chloride (**45**)

1-Chloro-4-nitrobenzene **44** (500 mg, 3.14 mmol) was stirred in chlorosulfonic acid (1.05 mL, 15.71 mmol) at 120 °C for 16 h. At reaction completion, the mixture was slowly poured onto ice-cold water (30 mL) and extracted twice with DCM (2 × 30 mL). The combined organic layers were dried over Na_2_SO_4_ and concentrated to dryness at low pressure to afford **44** as a brownish solid (374.1 mg, 46% yield). UPLC/MS: *R*_*t*_ = 2.14 min (gradient 1); MS (ESI) *m*/*z*: 253.7 [M – H]^−^, C_6_H_2_Cl_2_NO_4_S [M – H]^−^ calcd: 253.9. ^1^H NMR (400 MHz, DMSO-*d*_6_): δ 8.61 (d, *J* = 2.9 Hz, 1H), 8.16 (dd, *J* = 8.7, 2.9 Hz, 1H), 7.70 (d, *J* = 8.6 Hz, 1H).

##### 2-Chloro-5-nitro-benzenesulfonamide (**46a**)

To an ice-cold solution of 5 mL of tetrahydrofuran and 4 mL of 20% aqueous NH_4_OH was added compound **45** (374.1 mg, 1.47 mmol) dissolved in THF, and the reaction mixture was stirred at room temperature for 1 h. The reaction crude was then evaporated to dryness at low pressure, and the residue was suspended in water (20 mL) and extracted twice with EtOAc (2 × 20 mL). The combined organic layers were dried over Na_2_SO_4_ and concentrated to dryness at low pressure. Purification by silica gel flash chromatography (cyclohexane/EtOAc from 90:10 to 70:30) afforded the pure **46a** (166.2 mg, 48% yield) as a brown solid. UPLC/MS: *R*_*t*_ = 1.42 min (gradient 1); MS (ESI) *m*/*z*: 235.3 [M – H]^−^, C_6_H_4_ClN_2_O_4_S [M – H]^−^ calcd: 235. 1. ^1^H NMR (400 MHz, DMSO-*d*_6_): δ 8.68 (d, *J* = 2.7 Hz, 1H), 8.42 (dd, *J* = 8.7, 2.8 Hz, 1H), 7.98 (s, 2H), 7.96 (d, *J* = 8.7 Hz, 1H).

##### 2-Chloro-*N*-methyl-5-nitro-benzenesulfonamide (**46b**)

The titled compound was synthesized according to general procedure 1 using intermediate **45** (347 mg, 1.46 mmol) and methylamine hydrochloride (100.7 mg, 1.46 mmol). Purification by silica gel flash chromatography (cyclohexane/TBME 95:05) afforded the pure **46b** (204.9 mg, 56% yield) as a brown solid. UPLC/MS: *R*_*t*_ = 1.62 min (gradient 1); MS (ESI) *m*/*z*: 249.3 [M – H]^−^. C_7_H_6_ClN_2_O_4_S [M – H]^−^ calcd: 249.1. ^1^H NMR (400 MHz, DMSO-*d*_6_): δ 8.61 (d, *J* = 2.7 Hz, 1H), 8.45 (dd, *J* = 8.7, 2.8 Hz, 1H), 8.11 (q, *J* = 4.9 Hz, 1H), 7.98 (d, *J* = 8.7 Hz, 1H), 2.53 (d, *J* = 4.7 Hz, 3H).

##### 2-Chloro-*N*,*N*-dimethyl-5-nitro-benzenesulfonamide (**46c**)

The titled compound was synthesized according to general procedure 1 using intermediate **45** (190.3 mg, 0.8 mmol) and dimethylamine hydrochloride (163.7 mg, 1.60 mmol). Purification by silica gel flash chromatography (cyclohexane/EtOAc 80:20) afforded the pure **46c** (156.32 mg, 74% yield) as a brownish solid. UPLC/MS: *R*_*t*_ = 1.98 min (gradient 1); MS (ESI) *m*/*z*: 265.3 [M + H]^+^. C_8_H_10_ClN_2_O_4_S [M + H]^+^ calcd: 265.0. ^1^H NMR (400 MHz, DMSO-*d*_6_): δ 8.59 (d, *J* = 2.7 Hz, 1H), 8.46 (dd, *J* = 8.7, 2.8 Hz, 1H), 8.01 (d, *J* = 8.7 Hz, 1H), 2.87 (s, 6H).

##### 2-(Butylamino)-5-nitro-benzenesulfonamide (**5**)

Compound **5** was synthesized according to the general procedure 2 using intermediate **46a** (50 mg, 0.21 mmol) and butylamine (0.1 mL, 1.05 mmol). The compound was obtained as a pure yellow solid without silica gel purification (55.96 mg, 97% yield). UPLC/MS: *R*_*t*_ = 2.03 min (gradient 1); MS (ESI) *m*/*z*: 274.4 [M – H]^+^. C_10_H_16_N_3_O_4_S [M + H]^+^ calcd: 274.1; ^1^H NMR (400 MHz, DMSO-*d*_6_): δ 8.48 (d, *J* = 2.7 Hz, 1H), 8.19 (dd, *J* = 9.4, 2.7 Hz, 1H), 6.95 (d, *J* = 9.4 Hz, 1H), 3.35 (m, 2H), 1.65–1.55 (m, 2H), 1.44–1.32 (m, 2H), 0.92 (t, *J* = 7.3 Hz, 3H). ^13^C NMR (100 MHz, DMSO-*d*_6_): δ 149.29 (Cq), 134.51 (Cq), 128.89 (CH), 125.35 (CH), 124.08 (Cq), 111.69 (CH), 42.54 (CH_2_), 30.14 (CH_2_), 19.49 (CH_2_), 13.64 (CH_3_). HRMS (AP-ESI) *m*/*z*: calcd for C_10_H_16_N_3_O_4_S [M + H]^+^, 274.0862; found, 274.0858.

##### 2-(Hexylamino)-5-nitro-benzenesulfonamide (**6**)

Compound **6** was synthesized according to the general procedure 2 using intermediate **46a** (50 mg, 0.21 mmol) and hexylamine (0.14 mL, 1.05 mmol). Purification by silica gel flash chromatography (cyclohexane/EtOAc from 90:10 to 70:30) afforded the pure **6** (59.81 mg, 94% yield) as a yellow solid. UPLC/MS: *R*_*t*_ = 2.34 min (gradient 1); MS (ESI) *m*/*z*: 302.5 [M + H]^+^. C_12_H_20_N_3_O_4_S [M + H]^+^ calcd: 302.1; ^1^H NMR (400 MHz, DMSO-*d*_6_): δ 8.49 (d, *J* = 2.7 Hz, 1H), 8.19 (ddd, *J* = 9.4, 2.8, 0.5 Hz, 1H), 7.72 (s, 2H), 6.95 (d, *J* = 9.4 Hz, 1H), 6.85 (t, *J* = 5.6 Hz, 1H), 3.37–3.28 (m, 2H), 1.66–1.56 (m, 2H), 1.41–1.25 (m, 6H), 0.90–0.83 (m, 3H). ^13^C NMR (100 MHz, DMSO-*d*_6_): δ 149.27 (Cq), 134.50 (Cq), 128.89 (CH), 125.35 (CH), 124.08 (Cq), 111.68 (CH), 42.83 (CH_2_), 30.88 (CH_2_), 28.00 (CH_2_), 25.91 (CH_2_), 22.02 (CH_2_), 13.86 (CH_3_). HRMS (AP-ESI) *m*/*z*: calcd for C_12_H_20_N_3_O_4_S [M + H]^+^, 302.1175; found, 302.1171.

##### 5-Nitro-2-(octylamino)benzenesulfonamide (**7**)

Compound **7** was synthesized according to the general procedure 2 using intermediate **46a** (50 mg, 0.21 mmol) and *n*-octylamine (0.175 mL, 1.05 mmol). Purification by silica gel flash chromatography (cyclohexane/EtOAc 80:20) afforded the pure **7** (64.27 mg, 93% yield) as a yellow solid. UPLC/MS: *R*_*t*_ = 2.61 min (gradient 1); MS (ESI) *m*/*z*: 330.5 [M + H]^+^. C_14_H_24_N_3_O_4_S [M + H]^+^ calcd: 330.1; ^1^H NMR (400 MHz, DMSO-*d*_6_): δ 8.49 (d, *J* = 2.8 Hz, 1H), 8.20 (dd, *J* = 9.4, 2.8 Hz, 1H), 7.73 (s, 2H), 6.95 (d, *J* = 9.4 Hz, 1H), 6.86 (s, 1H), 3.34–3.29 (m, 2H), 1.62 (p, *J* = 7.2 Hz, 2H), 1.41–1.20 (m, 10H), 0.90–0.81 (m, 3H). ^13^C NMR (101 MHz, DMSO-*d*_6_): δ 149.30 (Cq), 134.51 (Cq), 128.93 (CH), 125.38 (CH), 124.09 (Cq), 111.72 (CH), 42.85 (CH_2_), 31.23 (CH_2_), 28.68 (CH_2_), 28.65 (CH_2_), 28.05 (CH_2_), 26.28 (CH_2_), 22.10 (CH_2_), 13.98 (CH_3_). HRMS (AP-ESI) *m*/*z*: calcd for C_14_H_24_N_3_O_4_S [M + H]^+^, 330.1488; found, 330.1477.

##### 2-(3,3-Dimethylbutylamino)-5-nitro-benzenesulfonamide (**8**)

Compound **8** was synthesized according to the general procedure 2 using intermediate **46a** (50 mg, 0.21 mmol) and 3,3-dimethylbutan-1-amine (0.148 mL, 1.05 mmol). Purification by silica gel flash chromatography (cyclohexane/EtOAc from 95:05 to 75:25) afforded the pure **8** (55.6 mg, 88% yield) as a yellow solid. UPLC/MS: *R*_*t*_ = 2.29 min (gradient 1); MS (ESI) *m*/*z*: 302.3 [M + H]^+^. C_12_H_20_N_3_O_4_S [M + H]^+^ calcd: 302.1; ^1^H NMR (400 MHz, DMSO-*d*_6_): δ 8.48 (d, *J* = 2.7 Hz, 1H), 8.21 (dd, *J* = 9.4, 2.8 Hz, 1H), 7.70 (s, 2H), 6.93 (d, *J* = 9.4 Hz, 1H), 6.78 (t, *J* = 4.7 Hz, 1H), 3.38–3.30 (m, 2H), 1.59–1.51 (m, 2H), 0.96 (s, 9H). ^13^C NMR (101 MHz, DMSO-*d*_6_): δ 149.19 (Cq), 134.50 (Cq), 128.94 (CH), 125.32 (CH), 124.14 (Cq), 111.60 (CH), 41.69 (CH_2_), 39.47 (CH_2_, extrapolated from HSQC), 29.68 (Cq), 29.18 (CH_3_, 3C). HRMS (AP-ESI) *m*/*z*: calcd for C_12_H_20_N_3_O_4_S [M + H]^+^, 302.1175; found, 302.1167.

##### 2-(Butylamino)-*N*-methyl-5-nitro-benzenesulfonamide (**9**)

Compound **9** was synthesized according to the general procedure 2 using intermediate **46b** (40 mg, 0.16 mmol) and butylamine (0.8 mL, 0.79 mmol). Purification by silica gel flash chromatography (cyclohexane/EtOAc 80:20) afforded the pure **9** (38.65 mg, 84% yield) as a yellow solid. UPLC/MS: *R*_*t*_ = 2.27 min (gradient 1); MS (ESI) *m*/*z*: 288.4 [M + H]^+^. C_11_H_18_N_3_O_4_S [M + H]^+^ calcd: 288.1; ^1^H NMR (400 MHz, DMSO-*d*_6_): δ 8.40 (d, *J* = 2.8 Hz, 1H), 8.21 (dd, *J* = 9.4, 2.7 Hz, 1H), 7.89 (s, 1H), 6.98 (d, *J* = 9.4 Hz, 1H), 6.88 (t, *J* = 5.6 Hz, 1H), 3.38–3.33 (m, 2H), 2.44 (s, 3H), 1.66–1.54 (m, 2H), 1.43–1.32 (m, 2H), 0.92 (t, *J* = 7.4 Hz, 3H). ^13^C NMR (101 MHz, DMSO-*d*_6_): δ 149.85 (Cq), 134.75 (Cq), 129.37 (CH), 126.72 (CH), 118.72 (Cq), 112.01 (CH), 42.50 (CH_2_), 30.08 (CH_2_), 28.18 (CH_3_), 19.47 (CH_2_), 13.62 (CH_3_). HRMS (AP-ESI) *m*/*z*: calcd for C_11_H_18_N_3_O_4_S [M + H]^+^, 288.1018; found, 288.1009.

##### 2-(Hexylamino)-*N*-methyl-5-nitro-benzenesulfonamide (**3**)

The title compound was synthesized according to the general procedure 2 using intermediate **46b** (40 mg, 0.16 mmol) and hexylamine (0.1 mL, 0.79 mmol). Purification by silica gel flash chromatography (cyclohexane/EtOAc 80:20) afforded the pure **3** (40.38 mg, 80% yield) as a yellow solid. UPLC/MS: *R*_*t*_ = 2.56 min (gradient 1); MS (ESI) *m*/*z*: 316.4 [M + H]^+^. C_13_H_22_N_3_O_4_S [M + H]^+^ calcd: 316.1; ^1^H NMR (400 MHz, DMSO-*d*_6_): δ 8.40 (d, *J* = 2.8 Hz, 1H), 8.21 (dd, *J* = 9.4, 2.8 Hz, 1H), 7.88 (s, 1H), 6.97 (d, *J* = 9.5 Hz, 1H), 6.92 (t, *J* = 5.6 Hz, 1H), 3.38–3.27 (m, 2H), 2.44 (s, 3H), 1.66–1.54 (m, 2H), 1.40–1.24 (m, 6H), 0.90–0.82 (m, 3H). ^13^C NMR (101 MHz, DMSO-*d*_6_): δ 149.89 (Cq), 134.71 (Cq), 129.29 (CH), 126.67 (CH), 112.94 (Cq), 111.90 (CH), 42.77 (CH_2_), 30.85 (CH_2_), 28.31 (CH_3_), 27.94 (CH_2_), 25.89 (CH_2_), 22.02 (CH_2_), 13.85 (CH_3_). HRMS (AP-ESI) *m*/*z*: calcd for C_13_H_22_N_3_O_4_S [M + H]^+^, 316.1331; found, 316.1334.

##### *N*-Methyl-5-nitro-2-(octylamino)benzenesulfonamide (**10**)

The title compound was synthesized according to the general procedure 2 using intermediate **46b** (40 mg, 0.16 mmol) and octylamine (0.13 mL, 0.79 mmol). Purification by silica gel flash chromatography (cyclohexane/EtOAc 80:20) afforded the pure **10** (39.56 mg, 72% yield). UPLC/MS: *R*_*t*_ = 1.99 min (gradient 1); MS (ESI) *m*/*z*: 344.4 [M + H]^+^. C_15_H_26_N_3_O_4_S [M + H]^+^ calcd: 344.1; ^1^H NMR (400 MHz, DMSO-*d*_6_): δ 8.41 (d, *J* = 2.8 Hz, 1H), 8.22 (dd, *J* = 9.4, 2.8 Hz, 1H), 7.89 (s, 1H), 6.98 (d, *J* = 9.4 Hz, 1H), 6.89 (t, *J* = 5.5 Hz, 1H), 3.36–3.30 (m, 2H), 2.45 (s, 3H), 1.65–1.56 (m, 2H), 1.40–1.20 (m, 10H), 0.89–0.82 (m, 3H). ^13^C NMR (101 MHz, DMSO-*d*_6_): δ 149.85 (Cq), 134.73 (Cq), 129.36 (CH), 126.72 (CH), 118.72 (Cq), 112.00 (CH), 42.78 (CH_2_), 31.17 (CH_2_), 28.61 (CH_2_, 2C), 28.17 (CH_2_), 27.96 (CH_3_), 26.22 (CH_2_), 22.05 (CH_2_), 13.92 (CH_3_). HRMS (AP-ESI) *m*/*z*: calcd for C_15_H_26_N_3_O_4_S [M + H]^+^, 364.1644; found, 364.1643.

##### 2-(3,3-Dimethylbutylamino)-*N*-methyl-5-nitro-benzenesulfonamide (**4**)

Compound **4** was synthesized according to the general procedure 2 using intermediate **46b** (40 mg, 0.16 mmol) and 3,3-dimethylbutan-1-amine (0.11 mL, 0.79 mmol). Purification by silica gel flash chromatography (cyclohexane/EtOAc 80:20) afforded the pure **4** (42.26 mg, 84% yield) as a yellow solid. UPLC/MS: *R*_*t*_ = 2.15 min (gradient 1); MS (ESI) *m*/*z*: 316.4 [M – H]^+^. C_13_H_22_N_3_O_4_S [M + H]^+^ calcd: 316.1; ^1^H NMR (400 MHz, DMSO-*d*_6_): δ 8.40 (d, *J* = 2.7 Hz, 1H), 8.23 (dd, *J* = 9.3, 2.8 Hz, 1H), 7.90–7.84 (m, 2H) 6.96 (d, *J* = 9.4 Hz, 1H), 6.81 (t, *J* = 5.4 Hz, 1H), 3.36–3.30 (m, 2H), 2.43 (s, 3H), 1.57–1.51 (m, 2H), 0.96 (s, 9H). ^13^C NMR (101 MHz, DMSO-*d*_6_): δ 149.73 (Cq), 134.72 (Cq), 129.41 (CH), 126.69 (CH), 118.74 (Cq), 111.89 (CH), 41.59 (CH_2_), 39.57 (CH_2_), 29.68 (Cq), 29.17 (CH_3_, 3C), 28.19 (CH_3_). HRMS (AP-ESI) *m*/*z*: calcd for C_13_H_22_N_3_O_4_S [M + H]^+^, 316.1331; found, 316.1329.

##### 2-(Butylamino)-*N*,*N*-dimethyl-5-nitro-benzenesulfonamide (**11**)

The title compound was synthesized according to the general procedure 2 using intermediate **46c** (50 mg, 0.19 mmol) and butylamine (93 μL, 0.94 mmol). Purification by silica gel flash chromatography (cyclohexane/EtOAc 75:25) afforded the pure **11** (41.45 mg, 72% yield) as a yellow solid. UPLC/MS: *R*_*t*_ = 2.47 min (gradient 1); MS (ESI) *m*/*z*: 302.4 [M + H]^+^. C_12_H_20_N_3_O_4_S [M + H]^+^ calcd: 302.1; ^1^H NMR (400 MHz, DMSO-*d*_6_): δ 8.29 (d, *J* = 2.8 Hz, 1H), 8.25 (ddd, *J* = 9.4, 2.7, 0.6 Hz, 1H), 7.21 (t, *J* = 5.6 Hz, 1H), 7.03 (d, *J* = 9.5 Hz, 1H), 3.38–3.32 (m, 2H), 2.72 (s, 6H), 1.63–1.53 (m, 2H), 1.42–1.32 (m, 2H), 0.93 (t, *J* = 7.3 Hz, 3H). ^13^C NMR (101 MHz, DMSO-*d*_6_): δ 150.77 (Cq), 134.83 (Cq), 129.79 (CH), 127.18 (CH), 115.37 (Cq), 112.54 (CH), 42.33 (CH_2_), 37.27 (CH_3_, 2C), 30.03 (CH_2_), 19.49 (CH_2_), 13.58 (CH_3_). HRMS (AP-ESI) *m*/*z*: calcd for C_12_H_20_N_3_O_4_S [M + H]^+^, 302.1175; found, 302.1174.

##### 2-(Hexylamino)-*N*,*N*-dimethyl-5-nitro-benzenesulfonamide (**12**)

Compound **12** was synthesized according to the general procedure 2 using intermediate **46c** (65 mg, 0.24 mmol) and hexylamine (0.16 mL, 1.21 mmol). Purification by silica gel flash chromatography (cyclohexane/EtOAc 80:20) afforded the pure **12** (68.42 mg, 87% yield) as a yellow solid. UPLC/MS: *R*_*t*_ = 1.80 min (gradient 1); MS (ESI) *m*/*z*: 328.5 [M – H]^−^. C_14_H_22_N_3_O_4_S [M – H]^−^ calcd: 328.1; ^1^H NMR (400 MHz, DMSO-*d*_6_): δ 8.28 (d, *J* = 2.7 Hz, 1H), 8.24 (ddd, *J* = 9.4, 2.8, 0.6 Hz, 1H), 7.21 (t, *J* = 5.6 Hz, 1H), 7.01 (d, *J* = 9.4 Hz, 1H), 3.36–3.30 (m, 2H), 2.71 (s, 6H), 1.62–1.53 (m, 2H), 1.38–1.24 (m, 6H), 0.90–0.82 (m, 3H). ^13^C NMR (101 MHz, DMSO-*d*_6_): δ 150.76 (Cq), 134.83 (Cq), 129.79 (CH), 127.19 (CH), 115.35 (Cq), 112.54 (CH), 42.60 (CH_2_), 37.27 (CH_3_, 2C), 30.81 (CH_2_), 27.88 (CH_2_), 25.90 (CH_2_), 22.01 (CH_2_), 13.83 (CH_3_). HRMS (AP-ESI) *m*/*z*: calcd for C_14_H_24_N_3_O_4_S [M + H]^+^, 330.1488; found, 330.1478.

##### *N*,*N*-Dimethyl-5-nitro-2-(octylamino)benzenesulfonamide (**13**)

Compound **13** was synthesized according to the general procedure 2 using intermediate **46c** (50 mg, 0.19 mmol) and octylamine (0.15 mL, 0.94 mmol). Purification by silica gel flash chromatography (cyclohexane/EtOAc 85:15) afforded the pure **13** (57.52 mg, 85% yield) as a yellow solid. UPLC/MS: *R*_*t*_ = 2.30 min (gradient 1); MS (ESI) *m*/*z*: 358.4 [M + H]^+^. C_16_H_28_N_3_O_4_S [M + H]^+^ calcd: 358.2; ^1^H NMR (400 MHz, DMSO-*d*_6_): δ 8.28 (d, *J* = 2.8 Hz, 1H), 8.23 (ddd, *J* = 9.4, 2.8, 0.6 Hz, 1H), 7.20 (t, *J* = 5.6 Hz, 1H), 7.01 (d, *J* = 9.5 Hz, 1H), 3.38–3.31 (m, 2H), 2.71 (s, 6H), 1.62–1.53 (m, 2H), 1.37–1.20 (m, 10H), 0.87–0.82 (m, 3H). ^13^C NMR (101 MHz, DMSO-*d*_6_): δ 150.76 (Cq), 134.83 (Cq), 129.79 (CH), 127.18 (CH), 115.35 (Cq), 112.54 (CH), 42.59 (CH_2_), 37.26 (CH_3_, 2C), 31.16 (CH_2_), 28.60 (CH_2_), 28.56 (CH_2_), 27.91 (CH_2_), 26.23 (CH_2_), 22.04 (CH_2_), 13.92 (CH_3_). HRMS (AP-ESI) *m*/*z*: calcd for C_16_H_28_N_3_O_4_S [M + H]^+^, 358.1801; found, 358.1807.

##### 2-(3,3-Dimethylbutylamino)-*N*,*N*-dimethyl-5-nitro-benzenesulfonamide (**14**)

Compound **14** was synthesized according to the general procedure 2 using intermediate **46c** (50 mg, 0.19 mmol) and 3,3-dimethylbutan-1-amine (0.13 mL, 0.94 mmol). Purification by silica gel flash chromatography (cyclohexane/EtOAc 85:15) afforded the pure **14** (51.11 mg, 82% yield). UPLC/MS: *R*_*t*_ = 2.70 min (gradient 1); MS (ESI) *m*/*z*: 330.4 [M + H]^+^. C_14_H_24_N_3_O_4_S [M + H]^+^ calcd: 330.1; ^1^H NMR (400 MHz, DMSO-*d*_6_): δ 8.28 (d, *J* = 2.7 Hz, 1H), 8.25 (ddd, *J* = 9.3, 2.8, 0.6 Hz, 1H), 7.16 (t, *J* = 5.6 Hz, 1H), 6.98 (d, *J* = 9.3 Hz, 1H), 3.38–3.32 (m, 2H), 2.71 (s, 6H), 1.52–1.47 (m, 2H), 0.95 (s, 9H). ^13^C NMR (101 MHz, DMSO-*d*_6_): δ 150.66 (Cq), 134.78 (Cq), 129.85 (CH), 127.18 (CH), 115.44 (Cq), 112.41 (CH), 41.49 (CH_2_), 39.41 (CH_2_), 37.31 (CH_3_, 2C), 29.70 (Cq), 29.15 (CH_3_, 3C). HRMS (AP-ESI) *m*/*z*: calcd for C_14_H_24_N_3_O_4_S [M + H]^+^, 330.1488; found, 330.1483.

##### 4-(Butylamino)-2-chloro-5-sulfamoyl-benzoic Acid (**48a**)

Compound **48a** was synthesized according to the general procedure 3 using intermediate **47** (70 mg, 0.26 mmol) and butylamine (0.13 mL, 1.32 mmol). Final trituration with cyclohexane (1 mL) afforded the pure **48a** (40.84 mg, 51% yield) as a white solid. UPLC/MS: *R*_*t*_ = 1.52 min (gradient 1); MS (ESI) *m*/*z*: 305.3 [M – H]^−^. C_11_H_14_ClN_2_O_4_S [M – H]^−^ calcd: 305.04; ^1^H NMR (400 MHz, DMSO-*d*_6_): δ 12.80 (bs, 1H), 8.26 (s, 1H), 7.57 (s, 2H), 6.84 (s, 1H), 6.39 (t, *J* = 5.3 Hz, 1H), 3.31–3.21 (m, 2H), 1.64–1.53 (m, 2H), 1.44–1.33 (m, 2H), 0.93 (t, *J* = 7.3 Hz, 3H).

##### 2-Chloro-4-(hexylamino)-5-sulfamoyl-benzoic Acid (**48b**)

Compound **48b** was synthesized according to the general procedure 3 using intermediate **47** (50 mg, 0.19 mmol) and hexylamine (0.12 mL, 0.95 mmol). Trituration with cyclohexane (1 mL) afforded the pure **48b** (52.82 mg, 83% yield) as a white solid. UPLC/MS: *R*_*t*_ = 1.78 min (gradient 1); MS (ESI) *m*/*z*: 333.4 [M – H]^−^. C_13_H_18_ClN_2_O_4_S [M – H]^−^ calcd: 333.1. ^1^H NMR (400 MHz, DMSO-*d*_6_): δ 12.77 (bs, 1H), 8.25 (s, 1H), 7.55 (s, 2H), 6.83 (s, 1H), 6.39 (t, *J* = 5.4 Hz, 1H), 3.27–3.20 (m, 2H), 1.59 (p, *J* = 7.1 Hz, 2H), 1.41–1.24 (m, 6H), 0.90–0.84 (m, 3H).

##### 2-Chloro-4-(octylamino)-5-sulfamoyl-benzoic Acid (**48c**)

Compound **48c** was synthesized according to the general procedure 3 using intermediate **47** (50 mg, 0.19 mmol) and octylamine (0.16 mL, 0.95 mmol). Trituration with cyclohexane (1 mL) afforded the pure **48c** (48.89 mg, 71% yield) as a white solid. UPLC/MS: *R*_*t*_ = 2.01 min (gradient 1); MS (ESI) *m*/*z*: 361.4 [M – H]^−^. C_15_H_22_ClN_2_O_4_S [M – H]^−^ calcd: 361.1. ^1^H NMR (400 MHz, DMSO-*d*_6_): δ 12.78 (bs, 1H), 8.26 (s, 1H), 7.56 (s, 2H), 6.84 (s, 1H), 6.40 (t, *J* = 5.3 Hz, 1H), 3.28–3.21 (m, 2H), 1.65–1.55 (m, 2H), 1.41–1.20 (m, 10H), 0.90–0.83 (m, 3H).

##### 2-Chloro-4-(3,3-dimethylbutylamino)-5-sulfamoyl-benzoic Acid (**48d**)

Compound **48d** was synthesized according to the general procedure 3 using intermediate **47** (50 mg, 0.19 mmol) and 3,3-dimethylbutan-1-amine (0.13 mL, 0.95 mmol). Trituration with cyclohexane (1 mL) afforded the pure **48d** (52.82 mg, 83% yield) as a white solid. UPLC/MS: *R*_*t*_ = 1.66 min (gradient 1); MS (ESI) *m*/*z*: 333.4 [M – H]^−^. C_13_H_18_ClN_2_O_4_S [M – H]^−^ calcd: 333.1; ^1^H NMR (400 MHz, DMSO-*d*_6_): δ 8.25 (s, 1H), 7.54 (s, 2H), 6.83 (s, 1H), 6.29 (t, *J* = 5.1 Hz, 1H), 3.27–3.20 (m, 2H), 1.56–1.50 (m, 2H), 0.96 (s, 9H).

##### 4-(Butylamino)-3-sulfamoyl-benzoic Acid (**15**)

Compound **15** was synthesized according to the general procedure 4 using intermediate **48a** (30 mg, 0.1 mmol). Final trituration with cyclohexane (1 mL) afforded the pure **15** (11.71 mg, 43% yield) as a white solid. UPLC/MS: *R*_*t*_ = 1.53 min (gradient 1); MS (ESI) *m*/*z*: 273.4 [M + H]^+^. C_11_H_17_N_2_O_4_S [M + H]^+^ calcd: 273.1; ^1^H NMR (400 MHz, DMSO-*d*_6_): δ 8.23 (d, *J* = 2.1 Hz, 1H), 7.87 (dd, *J* = 8.8, 2.2 Hz, 1H), 7.46 (s, 2H), 6.83 (d, *J* = 8.9 Hz, 1H), 6.37 (t, *J* = 5.4 Hz, 1H), 3.28–3.21 (m, 2H), 1.64–1.55 (m, 2H), 1.44–1.34 (m, 2H), 0.92 (t, *J* = 7.3 Hz, 3H). ^13^C NMR (101 MHz, DMSO-*d*_6_): δ 166.65 (CO), 147.89 (Cq), 134.39 (CH), 130.61 (CH), 124.14 (Cq), 116.25 (Cq), 111.13 (CH), 42.25 (CH_2_), 30.32 (CH_2_), 19.57 (CH_2_), 13.70 (CH_3_). HRMS (AP-ESI) *m*/*z*: calcd for C_11_H_17_N_2_O_4_S [M + H]^+^, 273.0909; found, 272.0903.

##### 4-(Hexylamino)-3-sulfamoyl-benzoic Acid (**16**)

Compound **16** was synthesized according to the general procedure 4 using intermediate **48b** (30.7 mg, 0.09 mmol). Final trituration with cyclohexane (1 mL) afforded the pure **16** (11.71 mg, 43% yield) as a white solid. UPLC/MS: *R*_*t*_ = 1.81 min; MS (ESI) *m*/*z*: 301.4 [M + H]^+^. C_13_H_21_N_2_O_4_S [M + H]^+^ calcd: 301.1; ^1^H NMR (400 MHz, DMSO-*d*_6_): δ 12.45 (bs, 1H), 8.23 (d, *J* = 2.1 Hz, 1H), 7.87 (dd, *J* = 8.8, 2.2 Hz, 1H), 7.46 (s, 2H), 6.82 (d, *J* = 8.9 Hz, 1H), 6.38 (t, *J* = 5.4 Hz, 1H), 3.27–3.20 (m, 2H), 1.60 (p, *J* = 7.1 Hz, 2H), 1.42–1.25 (m, 6H), 0.92–0.80 (m, 3H). ^13^C NMR (101 MHz, DMSO): δ 166.59 (CO), 147.89 (Cq), 134.38 (CH), 130.61 (CH), 124.14 (Cq), 116.15 (Cq), 111.14 (CH), 42.56 (CH_2_), 30.94 (CH_2_), 28.16 (CH_2_), 26.02 (CH_2_), 22.04 (CH_2_), 13.88 (CH_3_). HRMS (AP-ESI) *m*/*z*: calcd for C_13_H_21_N_2_O_4_S [M + H]^+^, 301.1222; found, 301.1219.

##### 4-(Octylamino)-3-sulfamoyl-benzoic Acid (**17**)

Compound **17** was synthesized according to the general procedure 4 using intermediate **48c** (35.7 mg, 0.1 mmol). Final trituration with cyclohexane (1 mL) afforded the pure **17** (9.68 mg, 36% yield) as a white solid. UPLC/MS: *R*_*t*_ = 2.16 min (gradient 1); MS (ESI) *m*/*z*: 329.4 [M + H]^+^. C_15_H_25_N_2_O_4_S [M + H]^+^ calcd: 329.1; ^1^H NMR (400 MHz, DMSO-*d*_6_): δ 12.43 (bs, 1H), 8.23 (d, *J* = 2.1 Hz, 1H), 7.86 (dd, *J* = 8.7, 2.1 Hz, 1H), 7.46 (s, 2H), 6.82 (d, *J* = 8.9 Hz, 1H), 6.38 (t, *J* = 5.3 Hz, 1H), 3.27–3.19 (m, 2H), 1.65–1.56 (m, 2H), 1.42–1.15 (m, 12H), 0.92–0.80 (m, 3H). ^13^C NMR (101 MHz, DMSO-*d*_6_): δ 166.61 (CO), 147.90 (Cq), 134.38 (CH), 130.61 (CH), 124.15 (Cq), 116.17 (Cq), 111.14 (CH), 42.57 (CH_2_), 31.23 (CH_2_), 28.72 (CH_2_), 28.65 (CH_2_), 28.21 (CH_2_), 26.37 (CH_2_), 22.09 (CH_2_), 13.95 (CH_3_). HRMS (AP-ESI) *m*/*z*: calcd for C_15_H_25_N_2_O_4_S [M + H]^+^, 329.1535; found, 329.1527.

##### 4-(3,3-Dimethylbutylamino)-3-sulfamoyl-benzoic Acid (**18**)

Compound **18** was synthesized according to the general procedure **4** using intermediate **48d** (29.6 mg, 0.09 mmol). Final trituration with cyclohexane (1 mL) afforded the pure **18** (15.13 mg, 56% yield) as a white solid. UPLC/MS: *R*_*t*_ = 1.80 min (gradient 1); MS (ESI) *m*/*z*: 301.4 [M + H]^+^. C_13_H_21_N_2_O_4_S [M + H]^+^ calcd: 301.1; ^1^H NMR (400 MHz, DMSO-*d*_6_): δ 12.48 (bs, 1H), 8.24 (d, *J* = 2.1 Hz, 1H), 7.89 (dd, *J* = 8.8, 2.1 Hz, 1H), 7.46 (s, 2H), 6.83 (d, *J* = 8.9 Hz, 1H), 3.28–3.21 (m, 2H), 1.59–1.52 (m, 2H), 0.97 (s, 9H). ^13^C NMR (101 MHz, DMSO): δ 166.62 (CO), 147.86 (Cq), 134.44 (CH), 130.60 (CH), 124.21 (Cq), 116.15 (Cq), 111.10 (CH), 41.98 (CH_2_), 39.23 (CH_2_), 29.68 (Cq), 29.27 (CH_3_, 3C). HRMS (AP-ESI) *m*/*z*: calcd for C_13_H_21_N_2_O_4_S [M + H]^+^, 301.1222; found, 301.1216.

##### 4-Fluoro-3-(methylsulfamoyl)benzoic Acid (**50a**)

Compound **50a** was obtained according to the procedure described by Savardi et al.^5^ (313.8 mg, 64% yield). NMR and UPLC/MS characterizations were in agreement with the ones previously reported.

##### 3-(Dimethylsulfamoyl)-4-Fluoro-benzoic Acid (**50b**)

Compound **50b** was obtained according to the procedure described by Savardi et al.^5^ (749 mg, 73% yield). NMR and UPLC/MS characterizations were in agreement with the ones previously reported.

##### 4-(Butylamino)-3-(-*N*-methylsulfamoyl)benzoic Acid (**19**)

Compound **19** was synthesized according to the general procedure 6 using intermediate **50a** (50 mg, 0.21 mmol) and butylamine (42 μL, 0.42 mmol) in dry 1,4-dioxane (0.7 mL). Trituration with cyclohexane (1 mL) afforded the pure **19** (47.10 mg, 78% yield) as a white solid. UPLC/MS: *R*_*t*_ = 1.66 min (gradient 1); MS (ESI) *m*/*z*: 285.4 [M – H]^−^. C_12_H_17_N_2_O_4_S [M – H]^−^ calcd: 285.1. ^1^H NMR (400 MHz, DMSO-*d*_6_): δ 8.15 (d, *J* = 2.1 Hz, 1H), 7.90 (dd, *J* = 8.8, 2.1 Hz, 1H), 7.66 (s, 1H), 6.86 (d, *J* = 8.9 Hz, 1H), 6.44 (t, *J* = 5.4 Hz, 1H), 3.24 (q, *J* = 6.6 Hz, 2H), 2.39 (s, 3H), 1.58 (p, *J* = 7.2 Hz, 2H), 1.43–1.32 (m, 2H), 0.92 (t, *J* = 7.3 Hz, 3H). ^13^C NMR (101 MHz, DMSO-*d*_6_): δ 166.50 (CO), 148.58 (Cq), 134.92 (CH), 131.97 (CH), 118.66 (Cq), 116.39 (Cq), 111.48 (CH), 42.19 (CH_2_), 30.25 (CH_2_), 28.18 (CH_3_), 19.55 (CH_2_), 13.67 (CH_3_). HRMS (AP-ESI) *m*/*z*: calcd for C_12_H_19_N_2_O_4_S [M + H]^+^, 287.1066; found, 287.1064.

##### 4-(Hexylamino)-3-(methylsulfamoyl)benzoic Acid (**20**)

Compound **30ab** was synthesized according to the general procedure 6 using intermediate **50a** (50 mg, 0.21 mmol) and hexylamine (57 μL, 0.42 mmol) in dry 1,4-dioxane (0.7 mL). Trituration with cyclohexane (1 mL) afforded the pure **20** (51.69 mg, 78% yield) as a white solid. UPLC/MS: *R*_*t*_ = 2.00 min (gradient 1); MS (ESI) *m*/*z*: 313.4 [M – H]^−^. C_14_H_21_N_2_O_4_S [M – H]^−^ calcd: 313.1. ^1^H NMR (400 MHz, DMSO-*d*_6_): δ 12.53 (bs, 1H), 8.15 (d, *J* = 2.1 Hz, 1H), 7.90 (dd, *J* = 8.8, 2.1 Hz, 1H), 7.63 (q, *J* = 5.0 Hz, 1H), 6.86 (d, *J* = 8.9 Hz, 1H), 6.44 (t, *J* = 5.3 Hz, 1H), 3.23 (q, *J* = 6.6 Hz, 2H), 2.39 (d, *J* = 4.9 Hz, 3H), 1.60 (p, *J* = 7.1 Hz, 2H), 1.40–1.25 (m, 6H), 0.90–0.83 (m, 3H). ^13^C NMR (101 MHz, DMSO-*d*_6_): δ 166.52 (CO), 148.57 (Cq), 134.92 (CH), 131.9 (CH), 118.65 (Cq), 116.46 (Cq), 111.48 (CH), 42.49 (CH_2_), 30.91 (CH_2_), 28.19 (CH_2_), 28.09 (CH_3_), 25.99 (CH_2_), 22.04 (CH_2_), 13.86 (CH_3_). HRMS (AP-ESI) *m*/*z*: calcd for C_14_H_23_N_2_O_4_S [M + H]^+^, 315.1379; found, 315.1387.

##### 3-(-*N*-Methylsulfamoyl)-4-(octylamino)benzoic Acid (**21**)

Compound **21** was obtained according to the procedure described by Savardi et al.^5^ (69.5 mg, 97% yield). NMR and UPLC/MS characterizations were in agreement with the ones previously reported. HRMS (AP-ESI) *m*/*z*: calcd for C_16_H_27_N_2_O_4_S [M + H]^+^, 343.1692; found, 343.1686.

##### 4-(-*N*-3,3-Dimethylbutylamino)-3-(methylsulfamoyl)benzoic Acid (**22**)

Compound **22** was synthesized according to the general procedure 6 using intermediate **50a** (50 mg, 0.21 mmol) and 3,3-dimethylbutan-1-amine (60 μL, 0.42 mmol) in dry 1,4-dioxane (0.7 mL). Trituration with cyclohexane (1 mL) afforded the pure **22** (50.56 mg, 84% yield) as a white solid. UPLC/MS: *R*_*t*_ = 1.93 min (gradient 1); MS (ESI) *m*/*z*: 313.4 [M – H]^−^. C_14_H_21_N_2_O_4_S [M – H]^−^ calcd: 313.1. ^1^H NMR (400 MHz, DMSO-*d*_6_): δ 12.52 (s, 1H), 8.15 (d, *J* = 2.1 Hz, 1H), 7.91 (dd, *J* = 8.8, 2.1 Hz, 1H), 7.62 (q, *J* = 5.0 Hz, 1H), 6.86 (d, *J* = 8.9 Hz, 1H), 6.35 (t, *J* = 5.2 Hz, 1H), 3.27–3.20 (m, 2H), 2.38 (d, *J* = 5.0 Hz, 3H), 1.57–1.50 (m, 2H), 0.96 (s, 9H). ^13^C NMR (101 MHz, DMSO-*d*_6_): δ 166.54 (CO), 148.48 (Cq), 134.96 (CH), 131.96 (CH), 118.66 (Cq), 116.38 (Cq), 111.41 (CH), 41.89 (CH_2_), 39.19 (CH_2_), 29.68 (Cq), 29.25 (CH_3_, 3C), 28.21 (CH_3_). HRMS (AP-ESI) *m*/*z*: calcd for C_14_H_23_N_2_O_4_S [M + H]^+^, 315.1379; found, 315.1370.

##### 4-(Butylamino)-3-(-*N*,*N*-dimethylsulfamoyl)benzoic Acid (**23**)

Compound **23** was synthesized according to the general procedure 6 using intermediate **50b** (50 mg, 0.20 mmol) and butylamine (40 μL, 0.40 mmol) in dry 1,4-dioxane (0.7 mL). Trituration with cyclohexane (1 mL) afforded the pure **23** (41.45 mg, 69% yield) as a white solid. UPLC/MS: *R*_*t*_ = 1.90 min (gradient 1); MS (ESI) *m*/*z*: 299.4 [M – H]^−^. C_13_H_19_N_2_O_4_S [M – H]^−^ calcd: 299.1. ^1^H NMR (400 MHz, DMSO-*d*_6_): δ 12.62 (s, 1H), 8.05 (d, *J* = 2.1 Hz, 1H), 7.93 (dd, *J* = 8.9, 2.1 Hz, 1H), 6.91 (d, *J* = 9.0 Hz, 1H), 6.74 (t, *J* = 5.4 Hz, 1H), 3.29–3.19 (m, 2H), 2.66 (s, 6H), 1.61–1.52 (m, 2H), 1.42–1.31 (m, 2H), 0.92 (t, *J* = 7.3 Hz, 3H). ^13^C NMR (101 MHz, DMSO-*d*_6_): δ 166.34 (CO), 149.54 (Cq), 135.42 (CH), 132.43 (CH), 116.62 (Cq), 115.18 (Cq), 112.05 (CH), 42.03 (CH_2_), 37.31 (CH_3_, 2C), 30.22 (CH_2_), 19.57 (CH_2_), 13.63 (CH_3_). HRMS (AP-ESI) *m*/*z*: calcd for C_13_H_21_N_2_O_4_S [M + H]^+^, 301.1222; found, 301.1215.

##### 3-(-*N*,*N*-Dimethylsulfamoyl)-4-(hexylamino)benzoic Acid (**24**)

The titled compound was synthesized according to the general procedure 6 using intermediate **50b** (50 mg, 0.20 mmol) and hexylamine (53 μL, 0.40 mmol) in dry 1,4-dioxane (0.7 mL). Trituration with cyclohexane (1 mL) afforded the pure **24** (53.20 mg, 81% yield) as a white solid. UPLC/MS: *R*_*t*_ = 2.17 min (gradient 1); MS (ESI) *m*/*z*: 327.4 [M – H]^−^. C_15_H_23_N_2_O_4_S [M – H]^−^ calcd: 327.1. ^1^H NMR (400 MHz, DMSO-*d*_6_): δ 12.63 (s, 1H), 8.04 (d, *J* = 2.1 Hz, 1H), 7.93 (dd, *J* = 8.8, 2.1 Hz, 1H), 6.90 (d, *J* = 9.0 Hz, 1H), 6.74 (t, *J* = 5.4 Hz, 1H), 3.28–3.18 (m, 2H), 2.65 (s, 6H), 1.57 (p, *J* = 7.0 Hz, 2H), 1.39–1.24 (m, 6H), 0.89–0.84 (m, 3H). ^13^C NMR (101 MHz, DMSO-*d*_6_): δ 166.37 (CO), 149.53 (Cq), 135.44 (CH), 132.45 (CH), 116.71 (Cq), 115.17 (Cq), 112.04 (CH), 42.31 (CH_2_), 37.31 (CH_3_, 2C), 30.86 (CH_2_), 28.07 (CH_2_), 26.01 (CH_2_), 22.05 (CH_2_), 13.86 (CH_3_). HRMS (AP-ESI) *m*/*z*: calcd for C_15_H_25_N_2_O_4_S [M + H]^+^, 329.1535; found, 329.1530.

##### 3-(-*N*,*N*-Dimethylsulfamoyl)-4-(octylamino)benzoic Acid (**25**)

Compound **25** was obtained according to the procedure described by Savardi et al.^5^ (59.9 mg, 84% yield). NMR and UPLC/MS characterizations were in agreement with the ones previously reported. HRMS (AP-ESI) *m*/*z*: calcd for C_17_H_29_N_2_O_4_S [M + H]^+^, 357.1848; found, 357.1848.

##### 4-(-*N*-3,3-Dimethylbutylamino)-3-(dimethylsulfamoyl)benzoic Acid (**26**)

Compound **30bd** was synthesized according to the general procedure 6 using intermediate **50b** (50 mg, 0.20 mmol) and 3,3-dimethylbutan-1-amine (57 μL, 0.40 mmol) in dry 1,4-dioxane (0.7 mL). Trituration with cyclohexane (1 mL) afforded the pure **26** (42 mg, 63% yield) as a white solid. UPLC/MS: *R*_*t*_ = 2.13 min (gradient 1); MS (ESI) *m*/*z*: 327.4 [M – H]^−^. C_15_H_23_N_2_O_4_S [M – H]^−^ calcd: 327.1. ^1^H NMR (400 MHz, DMSO-*d*_6_): δ 12.63 (s, 1H), 8.05 (d, *J* = 2.0 Hz, 1H), 7.95 (dd, *J* = 8.9, 2.1 Hz, 1H), 6.90 (d, *J* = 8.9 Hz, 1H), 6.69 (t, *J* = 5.3 Hz, 1H), 3.29–3.22 (m, 2H), 2.66 (s, 6H), 1.54–1.46 (m, 2H), 0.96 (s, 9H). ^13^C NMR (101 MHz, DMSO-*d*_6_): δ 166.37 (CO), 149.48 (Cq), 135.47 (CH), 132.45 (CH), 116.59 (Cq), 115.27 (Cq), 111.98 (CH), 41.85 (CH_2_), 38.87 (CH_2_, extrapolated from HSQC), 37.35 (CH_3_, 2C), 29.71 (Cq), 29.24 (CH_3_, 3C). HRMS (AP-ESI) *m*/*z*: calcd for C_15_H_25_N_2_O_4_S [M + H]^+^, 329.1535; found, 329.1544.

##### 3-(-*N*,*N*-Dimethylsulfamoyl)-4-(4,4,4-trifluorobutylamino)benzoic Acid (**27**)

Compound **27** was synthesized according to the general procedure 6 using intermediate **50b** (50 mg, 0.20 mmol) and commercial 4,4,4-trifluorobutylamine (48 μL, 0.40 mmol) in dry 1,4-dioxane (0.7 mL). Trituration with cyclohexane (1 mL) afforded the pure **27** (40.13 mg, 57% yield) as a white solid. UPLC/MS: *R*_*t*_ = 1.78 min (gradient 1); MS (ESI) *m*/*z*: 353.4 [M – H]^−^. C_13_H_16_F_3_N_2_O_4_S [M – H]^−^ calcd: 353.1. ^1^H NMR (400 MHz, DMSO-*d*_6_): δ 12.64 (bs, 1H), 8.07 (d, *J* = 2.1 Hz, 1H), 7.95 (dd, *J* = 8.8, 2.1 Hz, 1H), 6.98 (d, *J* = 9.0 Hz, 1H), 6.88 (t, *J* = 5.9 Hz, 1H), 3.38 (q, *J* = 6.8 Hz, 2H), 2.67 (s, 6H), 2.40–2.25 (m, 2H), 1.83–1.73 (m, 2H). ^13^C NMR (101 MHz, DMSO-*d*_6_): δ 166.28 (CO), 149.27 (Cq), 135.41 (CH), 131.66 (CH), 127.45 (CF_3_, q, ^1^*J*_CF_ = 277.1 Hz), 116.97 (Cq), 115.56 (Cq), 112.01 (CH), 40.95 (CH_2_), 37.28 (CH_3_, 2C), 30.09 (CH_2_, q, ^2^*J*_CF_ = 28.1 Hz), 20.90 (CH_2_). ^19^F NMR (565 MHz, DMSO-*d*_6_): δ −63.68 (t, *J* = 11.7 Hz). HRMS (AP-ESI) *m*/*z*: calcd for C_13_H_18_F_3_N_2_O_4_S [M + H]^+^, 355.0939; found, 355.0942.

##### 3-(-*N*,*N*-Dimethylsulfamoyl)-4-(6,6,6-trifluorohexylamino)benzoic Acid (**28**)

Compound **28** was synthesized according to the general procedure 6 using intermediate **50b** (50 mg, 0.20 mmol) and commercial 6,6,6-trifluorohexylamine (60 μL, 0.40 mmol) in dry 1,4-dioxane (0.7 mL). Trituration with cyclohexane (1 mL) afforded the pure **28** (57.32 mg, 75% yield). UPLC/MS: *R*_*t*_ = 2.02 min (gradient 1); MS (ESI) *m*/*z*: 381.4 [M – H]^−^. C_15_H_20_F_3_N_2_O_4_S [M – H]^−^ calcd: 381.1. ^1^H NMR (400 MHz, DMSO-*d*_6_): δ 12.64 (bs, 1H), 8.05 (d, *J* = 2.1 Hz, 1H), 7.94 (dd, *J* = 8.8, 2.1 Hz, 1H), 6.93 (d, *J* = 9.0 Hz, 1H), 6.77 (t, *J* = 5.4 Hz, 1H), 3.26 (q, *J* = 6.8 Hz, 2H), 2.66 (s, 6H), 2.32–2.18 (m, 2H), 1.62 (p, *J* = 7.4 Hz, 2H), 1.58–1.48 (m, 2H), 1.47–1.37 (m, 2H). ^13^C NMR (101 MHz, DMSO-*d*_6_): δ 166.31 (CO), 149.49 (Cq), 135.38 (CH), 132.43 (CH), 127.49 (CF_3_, q, ^1^*J*_CF_ = 277.1 Hz), 116.62 (Cq), 115.21 (Cq), 112.05 (CH), 42.09 (CH_2_), 37.30 (CH_3_, 2C), 32.31 (CH_2_, q, ^2^*J*_CF_ = 27.3 Hz), 27.66 (CH_2_), 25.32 (CH_2_), 21.12 (CH_2_). ^19^F NMR (565 MHz, DMSO-*d*_6_): δ −63.71 (t, *J* = 11.7 Hz). HRMS (AP-ESI) *m*/*z*: calcd for C_15_H_22_F_3_N_2_O_4_S [M + H]^+^, 383.1252; found, 383.1250.

##### 3-(-*N*,*N*-Dimethylsulfamoyl)-4-(8,8,8-trifluorooctylamino)benzoic Acid (**1**)

Compound **1** was synthesized according to the general procedure 6 using intermediate **50b** (50 mg, 0.20 mmol) and amine **53a** (89 mg, 0.40 mmol) in dry 1,4-dioxane (0.7 mL). Purification by silica gel flash chromatography (CH_2_Cl_2_/MeOH from 100:0 to 98:02) followed by trituration with cyclohexane (1 mL) afforded the pure compound **1** (44.3 mg, 55% yield) as a white solid. UPLC/MS: *R*_*t*_ = 2.28 min (gradient 1); MS (ESI) *m*/*z*: 409.4 [M – H]^−^. C_17_H_24_F_3_N_2_O_4_S [M – H]^−^ calcd: 409.1. ^1^H NMR (400 MHz, DMSO-*d*_6_): δ 12.62 (s, 1H), 8.05 (d, *J* = 2.1 Hz, 1H), 7.93 (dd, *J* = 8.8, 2.1 Hz, 1H), 6.91 (d, *J* = 9.0 Hz, 1H), 6.75 (t, *J* = 5.4 Hz, 1H), 3.24 (q, *J* = 6.6 Hz, 2H), 2.66 (s, 6H), 2.29–2.14 (m, 2H), 1.64–1.52 (m, 2H), 1.52–1.39 (m, 2H), 1.40–1.25 (m, 6H). ^13^C NMR (101 MHz, DMSO-*d*_6_): δ 166.31 (CO), 149.50 (Cq), 135.38 (CH), 132.42 (CH), 127.68 (CF_3_, q, ^1^*J*_CF_ = 276.6 Hz), 116.62 (Cq), 115.18 (Cq), 112.01 (CH), 42.22 (CH_2_), 37.27 (CH_3_, 2C), 32.34 (CH_2_, q, ^2^*J*_CF_ = 26.9 Hz), 28.11 (CH_2_), 27.97 (CH_2_), 27.79 (CH_2_), 26.06 (CH_2_), 21.31 (CH_2_). ^19^F NMR (565 MHz, DMSO-*d*_6_): δ −63.77 (t, *J* = 11.7 Hz). HRMS (AP-ESI) *m*/*z*: calcd for C_17_H_26_F_3_N_2_O_4_S [M + H]^+^, 411.1565; found, 411.1565.

##### 3-(-*N*,*N*-Dimethylsulfamoyl)-4-(2-methoxyethylamino)benzoic Acid (**29**)

Compound **29** was synthesized according to the general procedure 6 using intermediate **50b** (50 mg, 0.20 mmol) and commercial 2-methoxyethylamine (36 μL, 0.40 mmol) in dry 1,4-dioxane (0.7 mL). Trituration with cyclohexane (1 mL) afforded the pure **29** (53.96 mg, 89% yield). UPLC/MS: *R*_*t*_ = 1.40 min (gradient 1); MS (ESI) *m*/*z*: 301.4 [M – H]^−^. C_12_H_17_N_2_O_5_S [M – H]^−^ calcd: 301.1. ^1^H NMR (400 MHz, DMSO-*d*_6_): δ 8.05 (d, *J* = 2.1 Hz, 1H), 7.93 (dd, *J* = 8.8, 2.1 Hz, 1H), 6.95 (d, *J* = 9.0 Hz, 1H), 6.89 (t, *J* = 5.3 Hz, 1H), 3.55 (t, *J* = 5.2 Hz, 2H), 3.40 (q, *J* = 5.3 Hz, 2H), 3.29 (s, 3H), 2.65 (s, 6H). ^13^C NMR (101 MHz, DMSO): δ 166.31 (CO), 149.44 (Cq), 135.37 (CH), 132.36 (CH), 116.93 (Cq), 115.51 (Cq), 112.21 (CH), 69.73 (CH_2_), 58.02 (OCH_3_), 41.89 (CH_2_), 37.28 (CH_3_, 2C). HRMS (AP-ESI) *m*/*z*: calcd for C_12_H_19_N_2_O_5_S [M + H]^+^, 303.1015; found, 303.1014.

##### 3-(-*N*,*N*-Dimethylsulfamoyl)-4-(4-methoxybutylamino)benzoic Acid (**30**)

Compound **30** was synthesized according to the general procedure 6 using intermediate **50b** (50 mg, 0.20 mmol) and commercial 4-methoxybutan-1-amine (51 μL, 0.40 mmol) in dry 1,4-dioxane (0.7 mL). Trituration with cyclohexane (1 mL) afforded the pure **30** (56.08 mg, 85% yield) as a white solid. UPLC/MS: *R*_*t*_ = 1.59 min (gradient 1); MS (ESI) *m*/*z*: 329.4 [M – H]^−^. C_14_H_21_N_2_O_5_S [M – H]^−^ calcd: 339.1. ^1^H NMR (400 MHz, DMSO-*d*_6_): δ 12.63 (s, 1H), 8.05 (d, *J* = 2.1 Hz, 1H), 7.93 (dd, *J* = 8.8, 2.1 Hz, 1H), 6.91 (d, *J* = 8.9 Hz, 1H), 6.77 (t, *J* = 5.5 Hz, 1H), 3.38–3.32 (m, 2H), 3.26 (q, *J* = 6.5 Hz, 2H), 3.22 (s, 3H), 2.65 (s, 6H), 1.65–1.51 (m, 4H). ^13^C NMR (101 MHz, DMSO-*d*_6_): δ 166.30 (CO), 149.48 (Cq), 135.38 (CH), 132.44 (CH), 116.59 (Cq), 115.22 (Cq), 112.02 (CH), 71.42 (CH_2_), 57.80 (OCH_3_), 42.11 (CH_2_), 37.29 (CH_3_, 2C), 26.37 (CH_2_), 25.01 (CH_2_). HRMS (AP-ESI) *m*/*z*: calcd for C_14_H_23_N_2_O_5_S [M + H]^+^, 331.1328; found, 331.1328.

##### 3-(-*N*,*N*-Dimethylsulfamoyl)-4-(6-methoxyhexylamino)benzoic Acid (**31**)

Compound **31** was synthesized according to the general procedure 6 using intermediate **50b** (50 mg, 0.20 mmol) and amine **53b** (53.1 mg, 0.40 mmol) in dry 1,4-dioxane (0.7 mL). Trituration with cyclohexane (1 mL) afforded the pure **31** (23.14 mg, 32% yield) as a white solid. UPLC/MS: *R*_*t*_ = 1.84 min (gradient 1); MS (ESI) *m*/*z*: 357.5 [M – H]^−^. C_16_H_25_N_2_O_5_S [M – H]^−^ calcd: 357.2. ^1^H NMR (400 MHz, DMSO-*d*_6_): δ 12.63 (s, 1H), 8.05 (d, *J* = 2.1 Hz, 1H), 7.93 (dd, *J* = 8.8, 2.1 Hz, 1H), 6.91 (d, *J* = 8.9 Hz, 1H), 6.77 (t, *J* = 5.5 Hz, 1H), 3.38–3.32 (m, 2H), 3.26 (q, *J* = 6.5 Hz, 2H), 3.22 (s, 3H), 2.65 (s, 6H), 1.65–1.51 (m, 4H). ^13^C NMR (101 MHz, DMSO-*d*_6_): δ 166.77 (CO), 149.73 (Cq), 135.75 (CH), 132.67 (CH), 117.19 (Cq), 115.32 (Cq), 112.31 (CH), 72.02 (CH_2_), 58.03 (OCH_3_), 42.48 (CH_2_), 37.54 (CH_3_, 2C), 29.14 (CH_2_), 26.37 (CH_2_), 25.53 (CH_2_). HRMS (AP-ESI) *m*/*z*: calcd for C_16_H_27_N_2_O_5_S [M + H]^+^, 359.1641; found, 359.1641.

##### 2-(8,8,8-Trifluorooctyl)isoindoline-1,3-dione (**52a**)

Compound **52a** was synthesized according to the general procedure 7 using potassium phthalimide (300 mg, 1.60 mmol) and commercial 8-bromo-1,1,1-trifluorooctane **51a** (0.4 mL, 2.08 mmol) in dry DMF (5.5 mL). Purification by silica gel flash chromatography (cyclohexane/EtOAc 85:15) afforded the pure **52a** (392.63 mg, 75% yield) as colorless oil. UPLC/MS: *R*_*t*_ = 1.76 min (gradient 2); MS (ESI) *m*/*z*: 314.4 [M + H]^+^. C_16_H_19_F_3_NO_2_ [M + H]^+^ calcd: 314.1. ^1^H NMR (400 MHz, chloroform-*d*): δ 7.86–7.81 (m, 2H), 7.73–7.67 (m, 2H), 3.70–3.65 (m, 2H), 2.11–1.97 (m, 2H), 1.68 (p, *J* = 7.2 Hz, 2H), 1.58–1.47 (m, 2H), 1.39–1.30 (m, 6H).

##### 2-(6-Methoxyhexyl)isoindoline-1,3-dione (**52b**)

Compound **52b** was synthesized according to the general procedure 7 using potassium phthalimide (300 mg, 1.60 mmol) and commercial 1-bromo-6-methoxyhexane **51b** (0.36 mL, 2.08 mmol) in dry DMF (5.5 mL). Purification by silica gel flash chromatography (cyclohexane/EtOAc 70:30) afforded the pure **52b** (355.72 mg, 84% yield) as colorless oil. UPLC/MS: *R*_*t*_ = 2.23 min (gradient 2); MS (ESI) *m*/*z*: 262.5 [M + H]^+^. C_15_H_20_NO_3_ [M + H]^+^ calcd: 262.1. ^1^H NMR (400 MHz, chloroform-*d*): δ 7.86–7.79 (m, 2H), 7.73–7.66 (m, 2H), 3.67 (t, *J* = 7.4 Hz, 2H), 3.34 (t, *J* = 6.5 Hz, 2H), 3.30 (s, 3H), 1.68 (p, *J* = 6.1, 5.6 Hz, 2H), 1.56 (p, *J* = 6.6 Hz, 2H), 1.43–1.31 (m, 4H).

##### 8,8,8-Trifluorooctan-1-amine (**53a**)

Compound **53a** was synthesized according to the general procedure 8 using intermediate **52a** (393 mg, 1.24 mmol) and hydrazine hydrate (0.14 mL, 1.86 mmol) in absolute ethanol (5.5 mL). Purification by basic alumina flash chromatography (dichloromethane/methanol 95:5) afforded the pure **53a** (136.31 mg, yield 60%) as colorless oil. UPLC/MS: *R*_*t*_ = 1.59 min (gradient 1); MS (ESI) *m*/*z*: 184.4 [M + H]^+^. C_8_H_17_F_3_N [M + H]^+^ calcd: 184.1. ^1^H NMR (400 MHz, DMSO-*d*_6_): δ 2.78–2.68 (m, 2H), 2.30–2.15 (m, 2H), 1.61–1.41 (m, 4H), 1.38–1.21 (m, 6H).

##### 6-Methoxyhexan-1-amine (**53b**)

Compound **53b** was synthesized according to the general procedure 8 using intermediate **52b** (356 mg, 1.35 mmol) and hydrazine hydrate (0.15 mL, 2.02 mmol) in absolute ethanol (5.5 mL). Purification by basic alumina flash chromatography (dichloromethane/methanol 90:10) afforded the pure **53b** (127.55 mg, 72% yield) as colorless oil. UPLC/MS: *R*_*t*_ = 1.00 min (gradient 1); MS (ESI) *m*/*z*: 132.4 [M + H]^+^. C_7_H_18_NO [M + H]^+^ calcd: 132.1. ^1^H NMR (400 MHz, DMSO-*d*_6_): δ 3.29 (t, *J* = 6.5 Hz, 2H), 3.20 (s, 3H), 1.51–1.43 (m, 2H), 2.68 (p, *J* = 6.2 Hz, 2H), 1.37–1.21 (m, 6H).

##### 3-(-*N*,*N*-Methylsulfamoyl)-4-(8,8,8-trifluorooctylamino)benzoic Acid (**32**)

Compound **32** was synthesized following the general procedure 6 previously described using intermediate **50a** (100 mg, 0.42 mmol) and amine **53a** (86.4 mg, 0.47 mmol) in dry 1,4-dioxane (1.4 mL). Purification by silica gel flash chromatography (CH_2_Cl_2_/MeOH from 100:0 to 98:02) followed by trituration with cyclohexane (2 mL) afforded the pure **32** (111.5 mg, 67% yield) as a white solid. UPLC-MS: *R*_*t*_ = 2.11 min (gradient 1); MS (ESI) *m*/*z*: 395.2 [M – H]^−^. C_16_H_22_F_3_N_2_O_4_S [M – H]^−^ calcd: 395.1. ^1^H NMR (400 MHz, DMSO-*d*_6_): δ 8.15 (d, *J* = 2.1 Hz, 1H), 7.90 (dd, *J* = 8.8, 2.1 Hz, 1H), 7.63 (q, *J* = 5.0 Hz, 1H), 6.86 (d, *J* = 8.9 Hz, 1H), 6.44 (t, *J* = 5.4 Hz, 1H), 3.24 (q, *J* = 6.7 Hz, 2H), 2.39 (d, *J* = 4.8 Hz, 3H), 2.28–2.15 (m, 2H), 1.64–1.55 (m, 2H), 1.51–1.42 (m, 2H), 1.39–1.30 (m, 6H). ^13^C NMR (101 MHz, DMSO-*d*_6_): δ 166.96 (CO), 149.03 (Cq), 135.37 (CH), 132.44 (CH), 128.22 (CF_3_, q, ^1^*J*_CF_ = 276.5 Hz), 119.13 (Cq), 116.87 (Cq), 111.96 (CH), 42.88 (CH_2_), 32.84 (CH_2_, q, ^2^*J*_CF_ = 27.17 Hz), 28.67 (CH_2_), 28.65 (CH_2_) 28.49 (CH_2_), 28.31 (CH_3_), 26.55 (CH_2_), 21.82 (CH_2_). ^19^F NMR (565 MHz, DMSO-*d*_6_): δ −63.76 (t, *J* = 11.7 Hz). HRMS (AP-ESI) *m*/*z*: calcd for C_16_H_24_F_3_N_2_O_4_S [M + H]^+^, 397.1409; found, 397.1403.

##### 4-Fluoro-3-pyrrolidin-1-yl-sulfonyl-benzoic Acid (**54a**)

Compound **54a** was synthesized following the general procedure 8 previously described using intermediate **49** (250 mg, 1.04 mmol) and pyrrolidine (0.26 mL, 3.11 mmol) in THF (8 mL). The described workup afforded pure **54a** (261.4 mg, 88% yield) as a white solid. UPLC/MS: *R*_*t*_ = 1.17 min (gradient 1); MS (ESI) *m*/*z*: 272.4 [M – H]^−^. C_11_H_11_FNO_4_S [M – H]^−^ calcd: 272.05. ^1^H NMR (400 MHz, DMSO-*d*_6_): δ 8.30 (dd, *J* = 6.8, 2.3 Hz, 1H), 8.25 (ddd, *J* = 8.6, 4.8, 2.3 Hz, 1H), 7.62 (dd, *J* = 10.1, 8.6 Hz, 1H), 3.28–3.21 (m, 4H), 1.81–1.73 (m, 4H).

##### 4-Fluoro-3-(1-piperidylsulfonyl)benzoic Acid (**54b**)

Compound **54b** was synthesized following the general procedure 8 previously described using intermediate **49** (250 mg, 1.04 mmol) and piperidine (0.31 mL, 3.11 mmol) in THF (8 mL). The described workup afforded pure **54b** (261.4 mg, 88% yield) as a white solid. UPLC/MS: *R*_*t*_ = 1.34 min (gradient 1); MS (ESI) *m*/*z*: 286.4 [M – H]^−^. C_12_H_13_FNO_4_S [M – H]^−^ calcd: 286.06. ^1^H NMR (400 MHz, DMSO-*d*_6_): δ 8.28–8.23 (m, 2H), 7.65–7.58 (m, 1H), 3.08 (t, *J* = 5.4 Hz, 4H), 1.58–1.49 (m, 4H), 1.46–1.39 (m, 2H).

##### 4-Fluoro-3-morpholinosulfonyl-benzoic Acid (**54c**)

Compound **54c** was synthesized following the general procedure 8 previously described using intermediate **49** (250 mg, 1.04 mmol) and morpholine (0.27 mL, 3.11 mmol) in THF (8 mL). The described workup afforded pure **54c** (248.1 mg, 83% yield) as a white solid. UPLC/MS: *R*_*t*_ = 1.03 min (gradient 1); MS (ESI) *m*/*z*: 288.4 [M – H]^−^. C_11_H_11_FNO_5_S [M – H]^−^ calcd: 288.04. ^1^H NMR (400 MHz, DMSO-*d*_6_): δ 8.32–8.24 (m, 2H), 7.64 (dd, *J* = 10.1, 8.5 Hz, 1H), 3.67–3.60 (m, 4H), 3.10–3.04 (m, 4H).

##### 3-(-*N*-Cyclopentylsulfamoyl)-4-fluoro-benzoic Acid (**54d**)

Compound **54d** was synthesized following the general procedure 8 previously described using intermediate **49** (250 mg, 1.04 mmol) and cyclopentane amine (0.21 mL, 2.07 mmol) in THF (8.5 mL). The described workup afforded the pure **54d** (203.7 mg, 68% yield) as a white solid. UPLC/MS: *R*_*t*_ = 1.25 min (gradient 1); MS (ESI) *m*/*z*: 286.4 [M – H]^−^. C_12_H_13_FNO_4_S [M – H]^−^ calcd: 286.06. ^1^H NMR (400 MHz, DMSO-*d*_6_): δ 8.33 (dd, *J* = 7.1, 2.3 Hz, 1H), 8.21 (ddd, *J* = 8.6, 4.7, 2.3 Hz, 1H), 8.12 (d, *J* = 7.6 Hz, 1H), 7.56 (dd, *J* = 10.0, 8.6 Hz, 1H), 3.58–3.48 (m, 1H), 1.68–1.48 (m, 4H), 1.45–1.28 (m, 4H).

##### 3-(-*N*-Cyclohexylsulfamoyl)-4-fluoro-benzoic Acid (**54e**)

Compound **54e** was synthesized following the general procedure 8 previously described using intermediate **49** (250 mg, 1.04 mmol) and cyclohexane amine (0.24 mL, 2.07 mmol) in THF (8.5 mL). The described workup and trituration with a cyclohexane/ethyl acetate 9:1 mixture (2 mL) afforded pure **54e** (185.6 mg, 59% yield) as a white solid. UPLC/MS: *R*_*t*_ = 1.37 min (gradient 1); MS (ESI) *m*/*z*: 286.4 [M – H]^−^. C_13_H_15_FNO_4_S [M – H]^−^ calcd: 286.06. ^1^H NMR (400 MHz, DMSO-*d*_6_): δ 8.33 (dd, *J* = 7.1, 2.3 Hz, 1H), 8.21 (ddd, *J* = 8.6, 4.7, 2.3 Hz, 1H), 8.12 (d, *J* = 7.6 Hz, 1H), 7.56 (dd, *J* = 10.0, 8.6 Hz, 1H), 3.58–3.48 (m, 1H), 1.68–1.48 (m, 4H) 1.53–1.42 (m, 2H), 1.45–1.28 (m, 4H).

##### 4-Fluoro-3-(*N*-(tetrahydro-2*H*-pyran-4-yl)sulfamoyl)benzoic Acid (**54f**)

The title compound was synthesized following the general procedure 8 previously described using intermediate **49** (250 mg, 1.04 mmol) and tetrahydro-2*H*-pyran-4-amine (0.32 mL, 2.07 mmol) in THF (8.5 mL). The described workup afforded pure **54f** (160.9 mg, 51% yield) as a white solid. UPLC/MS: *R*_*t*_ = 0.93 min (gradient 1); MS (ESI) *m*/*z*: 302.1 [M – H]^−^. C_12_H_13_FNO_5_S [M – H]^−^ calcd: 302.06. ^1^H NMR (400 MHz, DMSO-*d*_6_): δ 8.34 (dd, *J* = 7.1, 2.3 Hz, 1H), 8.27 (d, *J* = 7.8 Hz, 1H), 8.24–8.18 (m, 1H), 7.57 (t, *J* = 9.3 Hz, 1H), 3.77–3.68 (m, 2H), 3.27–3.19 (m, 3H), 1.58–1.49 (m, 2H), 1.49–1.37 (m, 2H).

##### 3-Pyrrolidin-1-ylsulfonyl-4-(8,8,8-trifluorooctylamino)benzoic Acid (**33**)

Compound **42b** was synthesized following the general procedure 6 previously described using intermediate **54a** (50 mg, 0.17 mmol) and amine **53a** (34.8 mg, 0.19 mmol) in dry 1,4-dioxane (0.55 mL). Purification by silica gel flash chromatography (CH_2_Cl_2_/MeOH from 100:0 to 99:01) followed by trituration with diethyl ether (1 mL) afforded the pure **33** (17.3 mg, 23% yield) as a white solid. UPLC/MS: *R*_*t*_ = 2.30 min (gradient 1); MS (ESI) *m*/*z*: 435.5 [M – H]^−^. C_19_H_26_F_3_N_2_O_4_S [M – H]^−^ calcd: 435.2. ^1^H NMR (400 MHz, DMSO-*d*_6_): δ 8.11 (d, *J* = 2.1 Hz, 1H), 7.92 (dd, *J* = 8.8, 2.1 Hz, 1H), 6.89 (d, *J* = 8.9 Hz, 1H), 6.74 (t, *J* = 5.3 Hz, 1H), 3.24 (q, *J* = 6.7 Hz, 2H), 3.18–3.11 (m, 4H), 2.29–2.14 (m, 2H), 1.79–1.68 (m, 4H), 1.57 (m, 2H), 1.46 (m, 2H), 1.33 (s, 6H). ^13^C NMR (150 MHz, DMSO-*d*_6_): δ 166.91 (CO), 149.92 (Cq), 135.83 (CH), 132.46 (CH), 128.14 (CF_3_, q, ^1^*J*_CF_ = 277.3 Hz), 117.34 (Cq), 116.85 (Cq), 112.50 (CH), 47.94 (CH_2_, 2C), 42.58 (CH_2_), 32.73 (CH_2_, q, ^2^*J*_CF_ = 27.2 Hz), 28.49 (CH_2_), 28.34 (CH_2_), 28.17 (CH_2_), 26.39 (CH_2_), 25.06 (CH_2_, 2C), 21.68 (CH_2_). ^19^F NMR (565 MHz, DMSO-*d*_6_): δ −63.73 (t, *J* = 11.7 Hz). HRMS (AP-ESI) *m*/*z*: calcd for C_19_H_28_F_3_N_2_O_4_S [M + H]^+^, 437.1722; found, 437.1728.

##### 3-(1-Piperidylsulfonyl)-4-(8,8,8-trifluorooctylamino)benzoic Acid (**34**)

Compound **34** was synthesized following the general procedure 6 previously described using intermediate **54b** (50 mg, 0.17 mmol) and amine **53a** (34.8 mg, 0.19 mmol) in dry 1,4-dioxane (0.55 mL). Purification by silica gel flash chromatography (CH_2_Cl_2_/MeOH from 100:0 to 99:01) followed by trituration with diethyl ether (1 mL) afforded the pure **34** (13 mg, 17% yield) as a white solid. UPLC/MS: *R*_*t*_ = 2.40 min (gradient 1); MS (ESI) *m*/*z*: 449.5 [M – H]^−^. C_20_H_28_F_3_N_2_O_4_S [M – H]^−^ calcd: 449.2. ^1^H NMR (400 MHz, DMSO-*d*_6_): δ 8.04 (d, *J* = 2.1 Hz, 1H), 7.92 (dd, *J* = 8.8, 2.1 Hz, 1H), 6.89 (d, *J* = 9.0 Hz, 1H), 6.69 (t, *J* = 5.4 Hz, 1H), 3.24 (q, *J* = 6.7 Hz, 2H), 2.98 (t, *J* = 5.4 Hz, 4H), 2.29–2.15 (m, 2H), 1.62–1.55 (m, 2H), 1.55–1.43 (m, 6H), 1.42–1.37 (m, 2H), 1.37–1.30 (m, 6H). ^13^C (101 MHz, chloroform-*d*): δ 170.93 (CO), 150.62 (Cq), 136.12 (CH), 133.97 (CH), 127.34 (CF_3_, q, ^1^*J*_CF_ = 276.6 Hz), 117.55 (Cq), 115.75 (Cq), 111.48 (CH), 46.85 (CH_2_, 2C), 43.30 (CH_2_), 33.82 (CH_2_), (q, ^2^*J*_CF_ = 28.16 Hz), 29.04 (CH_2_), 28.88 (CH_2_), 28.74 (CH_2_), 26.96 (CH_2_), 25.33 (CH_2_, 2C), 23.64 (CH_2_), 21.95 (CH_2_). ^19^F NMR (565 MHz, DMSO-*d*_6_): δ −63.69 (t, *J* = 11.7 Hz). HRMS (AP-ESI) *m*/*z*: calcd for C_20_H_30_F_3_N_2_O_4_S [M + H]^+^, 451.1878; found, 451.1879.

##### 3-Morpholinosulfonyl-4-(8,8,8-trifluorooctylamino)benzoic Acid (**35**)

Compound **35** was synthesized following the general procedure 6 previously described using intermediate **54c** (50 mg, 0.17 mmol) and amine **53a** (34.8 mg, 0.19 mmol) in dry 1,4-dioxane (0.55 mL). Purification by silica gel flash chromatography (CH_2_Cl_2_/MeOH from 100:0 to 98:02) followed by trituration with diethyl ether (1 mL) afforded the pure **42d** (28.4 mg, 37% yield) as a white solid. UPLC/MS: *R*_*t*_ = 2.21 min (gradient 1); MS (ESI) *m*/*z*: 451.2 [M – H]^−^. C_19_H_26_F_3_N_2_O_5_S [M – H]^−^ calcd: 451.2. ^1^H NMR (400 MHz, chloroform-*d*): δ 8.33 (d, *J* = 2.1 Hz, 1H), 8.07 (dd, *J* = 8.9, 2.1 Hz, 1H), 6.87 (t, *J* = 5.0 Hz, 1H), 6.74 (d, *J* = 9.0 Hz, 1H), 3.77–3.70 (m, 4H), 3.21 (q, *J* = 7.0 Hz, 2H), 3.12–3.06 (m, 4H), 2.14–1.99 (m, 2H), 1.73–1.63 (m, 2H), 1.61–1.50 (m, 2H), 1.48–1.32 (m, 6H). ^13^C NMR (101 MHz, DMSO-*d*_6_): δ 166.25 (CO), 149.50 (Cq), 135.64 (CH), 132.61 (CH), 127.73 (CF_3_, q, ^1^*J*_CF_ = 278.6 Hz), 116.65 (Cq), 114.65 (Cq), 112.13 (CH), 65.27 (CH_2_, 2C), 45.53 (CH_2_, 2C), 42.26 (CH_2_), 32.35 (CH_2_, q, ^2^*J*_CF_ = 27.24 Hz), 28.14 (CH_2_), 27.96 (CH_2_), 27.83 (CH_2_), 26.11 (CH_2_), 21.33 (CH_2_). ^19^F NMR (565 MHz, DMSO-*d*_6_): δ −63.76 (t, *J* = 11.7 Hz). HRMS (AP-ESI) *m*/*z*: calcd for C_19_H_28_F_3_N_2_O_5_S [M + H]^+^, 453.1671; found, 453.1675.

##### 3-(-*N*-Cyclopentylsulfamoyl)-4-(8,8,8-trifluorooctylamino)benzoic Acid (**36**)

Compound **36** was synthesized following the general procedure 6 previously described using intermediate **54d** (50 mg, 0.17 mmol) and amine **53a** (35.1 mg, 0.19 mmol) in dry 1,4-dioxane (0.6 mL). Purification by silica gel flash chromatography (CH_2_Cl_2_/MeOH from 100:0 to 98:02) followed by trituration with diethyl ether (1 mL) afforded the pure **36** (31.7 mg, 41% yield) as a white solid. UPLC/MS: *R*_*t*_ = 2.33 min (gradient 1); MS (ESI) *m*/*z*: 449.5 [M – H]^−^. C_20_H_28_F_3_N_2_O_4_S [M – H]^−^ calcd: 449.2. ^1^H NMR (400 MHz, chloroform-*d*): δ 8.49 (d, *J* = 2.1 Hz, 1H), 8.08 (dd, *J* = 8.8, 2.1 Hz, 1H), 6.75 (d, *J* = 8.9 Hz, 1H), 6.53 (s, 1H), 4.63–4.51 (m, 1H), 3.63–3.53 (m, 1H), 3.25 (t, *J* = 7.1 Hz, 2H), 2.14–2.00 (m, 2H), 1.85–1.75 (m, 2H), 1.74–1.65 (m, 2H), 1.65–1.54 (m, 4H), 1.53–1.47 (m, 2H), 1.46–1.36 (m, 6H), 1.36–1.27 (m, 2H). ^13^C NMR (101 MHz, DMSO-*d*_6_): δ 166.51 (CO), 148.28 (Cq), 134.75 (CH), 131.79 (CH), 127.71 (CF_3_, q, ^1^*J*_CF_ = 268.1 Hz), 120.88 (Cq), 116.25 (Cq), 111.26 (CH), 54.06 (CH), 42.40 (CH_2_), 32.36 (CH_2_, q, ^2^*J*_CF_ = 26.86 Hz) 32.32 (CH_2_, 2C), 28.24 (CH_2_), 28.10 (CH_2_), 27.86 (CH_2_), 26.12 (CH_2_), 22.75 (CH_2_, 2C), 21.34 (CH_2_). ^19^F NMR (565 MHz, DMSO-*d*_6_): δ −63.75 (t, *J* = 11.7 Hz). HRMS (AP-ESI) *m*/*z*: calcd for C_20_H_30_F_3_N_2_O_4_S [M + H]^+^, 451.1878; found, 451.1891.

##### 3-(-*N*-Cyclohexylsulfamoyl)-4-(8,8,8-trifluorooctylamino)benzoic Acid (**37**)

Compound **37** was synthesized following the general procedure 6 previously described using intermediate **54e** (50 mg, 0.16 mmol) and amine **53a** (33.4 mg, 0.18 mmol) in dry 1,4-dioxane (0.55 mL). Purification by silica gel flash chromatography (CH_2_Cl_2_/MeOH from 100:0 to 98:02) followed by trituration with diethyl ether (1 mL) afforded the pure **37** (25.3 mg, 34% yield). UPLC/MS: *R*_*t*_ = 2.40 min (gradient 1); MS (ESI) *m*/*z*: 463.5 [M – H]^−^. C_21_H_30_F_3_N_2_O_4_S [M – H]^−^ calcd: 463.2. ^1^H NMR (400 MHz, chloroform-*d*): δ 8.49 (d, *J* = 2.1 Hz, 1H), 8.07 (dd, *J* = 8.8, 2.1 Hz, 1H), 6.74 (d, *J* = 8.9 Hz, 1H), 6.50 (s, 1H), 4.49 (d, *J* = 7.9 Hz, 1H), 3.25 (t, *J* = 7.1 Hz, 2H), 3.18–3.07 (m, 1H), 2.14–2.00 (m, 2H), 1.79–1.66 (m, 4H), 1.66–1.49 (m, 6H), 1.48–1.34 (m, 6H), 1.30–1.19 (m, 3H), 1.18–1.07 (m, 2H). ^13^C NMR (101 MHz, DMSO-*d*_6_): δ 166.53 (CO), 148.17 (Cq), 134.69 (CH), 131.51 (CH), 127.62 (CF_3_, q, ^1^*J*_CF_ = 278.3 Hz), 121.48 (Cq), 116.18 (Cq), 111.25 (CH), 51.60 (CH), 42.40 (CH_2_), 33.06 (CH_2_, 2C), 32.36 (CF_3_, q, ^2^*J*_CF_ = 27.14 Hz), 28.26 (CH_2_), 28.14 (CH_2_), 27.87 (CH_2_), 26.14 (CH_2_), 24.80 (CH_2_), 24.13 (CH_2_, 2C), 21.36 (CH_2_). ^19^F NMR (565 MHz, DMSO-*d*_6_): δ −63.75 (t, *J* = 11.7 Hz). HRMS (AP-ESI) *m*/*z*: calcd for C_21_H_32_F_3_N_2_O_4_S [M + H]^+^, 465.2035; found, 465.2046.

##### 3-(*N*-(Tetrahydro-2*H*-pyran-4-yl)sulfamoyl)-4-((8,8,8-trifluorooctyl)amino)benzoic Acid (**38**)

Compound **38** was synthesized following the general procedure 6 previously described using intermediate **54f** (50 mg, 0.16 mmol) and amine **53a** (33.4 mg, 0.18 mmol) in dry 1,4-dioxane (0.55 mL). Purification by silica gel flash chromatography (CH_2_Cl_2_/MeOH from 100:0 to 98:02) followed by trituration with diethyl ether (1 mL) afforded the pure **38** (20.9 mg, 28% yield) as a white solid. UPLC/MS: *R*_*t*_ = 2.40 min (gradient 1); MS (ESI) *m*/*z*: 463.5 [M – H]^−^. C_20_H_28_F_3_N_2_O_5_S [M – H]^−^ calcd: 463.2. ^1^H NMR (400 MHz, DMSO-*d*_6_): δ 8.21 (d, *J* = 2.1 Hz, 1H), 7.96 (d, *J* = 7.6 Hz, 1H), 7.88 (dd, *J* = 8.8, 2.1 Hz, 1H), 6.85 (d, *J* = 8.9 Hz, 1H), 6.35 (t, *J* = 5.6 Hz, 1H), 3.74–3.65 (m, 2H), 3.30–3.17 (m, 4H), 3.17–3.06 (m, 1H), 2.29–2.13 (m, 2H), 1.64–1.56 (m, 2H), 1.53–1.42 (m, 4H), 1.40–1.29 (m, 8H). ^13^C NMR (101 MHz, DMSO-*d*_6_): δ 166.66 (CO), 148.20 (Cq), 134.95 (CH), 131.63 (CH), 127.82 (CF_3_, q, ^1^*J*_CF_ = 277.1 Hz), 121.32 (Cq), 116.45 (Cq), 111.44 (CH), 65.45 (CH), 48.86 (CH_2_, 2C), 42.49 (CH_2_), 33.24 (CH_2_, 2C), 32.42 (CH_2_, q, ^2^*J*_CF_ = 26.29 Hz), 28.35 (CH_2_), 28.19 (CH_2_), 27.96 (CH_2_), 26.24 (CH_2_), 21.46 (CH_2_). ^19^F NMR (565 MHz, DMSO-*d*_6_): δ −63.75 (t, *J* = 11.7 Hz). HRMS (AP-ESI) *m*/*z*: calcd for C_20_H_30_F_3_N_2_O_5_S [M + H]^+^, 467.1828; found, 467.1835.

##### 2-Chloro-5-(*N*,*N*-dimethylsulfamoyl)-4-fluorobenzoic Acid (**56**)

2-Chloro-5-(chlorosulfonyl)-4-fluorobenzoic acid **55** (250 mg, 0.91 mmol) dissolved in 1.5 mL of THF was added dropwise to 8 mL of an ice-cold solution of dimethylamine (0.45 mL, 0.91 mmol) in THF and DIPEA (0.38 mL, 2.72 mmol) and stirred for 30 h. At reaction completion, the reaction mixture was evaporated to dryness. The dry residue was dissolved in water and treated with 2 N HCl until reaching pH 3. The resulting precipitated solid was filtered and rinsed with water. Final purification by silica gel flash chromatography (CH_2_Cl_2_/MeOH from 100:0 to 98:02) afforded the pure **56** (105.1 mg, 41% yield) as a white solid. UPLC/MS: *R*_*t*_ = 1.04 min (gradient 1); MS (ESI) *m*/*z*: 280.0 [M – H]^−^. C_9_H_8_ClFNO_4_S [M – H]^−^ calcd: 280.1. ^1^H NMR (400 MHz, DMSO-*d*_6_): δ 8.17 (d, *J* = 7.5 Hz, 1H), 7.91 (d, *J* = 10.1 Hz, 1H), 2.76 (d, *J* = 1.8 Hz, 6H).

##### 2-Chloro-5-(*N*,*N*-dimethylsulfamoyl)-4-((8,8,8-trifluorooctyl)amino)benzoic Acid (**39**)

Compound **39** was synthesized following the general procedure 6 previously described using intermediate **56** (60 mg, 0.21 mmol) and amine **53a** (46.8 mg, 0.21 mmol) in dry 1,4-dioxane (0.8 mL). Purification by silica gel flash chromatography (CH_2_Cl_2_/MeOH from 100:0 to 97:03) followed by trituration with diethyl ether (1 mL) afforded the pure **39** (82.2 mg, 88% yield) as a white solid. UPLC/MS: *R*_*t*_ = 2.14 min (gradient 1); MS (ESI) *m*/*z*: 443.1 [M – H]^−^. C_17_H_23_ClF_3_N_2_O_4_S [M – H]^−^ calcd: 444.2. ^1^H NMR (400 MHz, chloroform-*d*): δ 8.37 (s, 1H), 6.88 (t, *J* = 5.0 Hz, 1H), 6.75 (s, 1H), 3.21–3.16 (m, 2H), 2.77 (s, 6H), 2.14–2.00 (m, 2H), 1.73–1.63 (m, 2H), 1.61–1.51 (m, 2H), 1.48–1.34 (m, 6H). ^13^C NMR (150 MHz, DMSO-*d*_6_): δ 164.85 (CO), 148.78 (Cq), 139.82 (Cq), 135.04 (CH), 127.76 (CF_3_, q, ^1^*J*_CF_ = 276.4 Hz), 114.29 (Cq), 113.80 (CH), 42.24 (CH_2_), 37.29 (CH_2_, 2C), 32.37 (CH_2_, q, ^2^*J*_CF_ = 27.89 Hz), 28.10 (CH_2_), 27.85 (CH_2_), 27.82 (CH_2_), 26.03 (CH_2_), 21.35 (CH_2_). ^19^F NMR (565 MHz, DMSO-*d*_6_): δ −63.77 (t, *J* = 11.7 Hz). HRMS (AP-ESI) *m*/*z*: calcd for C_17_H_25_C_l_F_3_N_2_O_4_S [M + H]^+^, 445.1176; found, 445.1189.

##### 5-(Chlorosulfonyl)-4-fluoro-2-hydroxybenzoic Acid (**58**)

4-Fluoro-2-hydroxy-benzoic acid **57** (2 g, 12.81 mmol) was stirred in chlorosulfonic acid (4.30 mL, 64.06 mmol) at 120 °C for 4 h. At reaction completion, the mixture was slowly poured onto ice-cold water (50 mL) and the resulting precipitated solid was collected by filtration to afford **58** (1.141 g, 35% yield) as a brownish solid. UPLC/MS: *R*_*t*_ = 1.42 min (gradient 1); MS (ESI) *m*/*z*: 253.2 [M – H]^−^. C_7_H_4_ClFO_5_S [M – H]^−^ calcd: 253.0. ^1^H NMR (400 MHz, DMSO-*d*_6_): δ 8.15 (d, *J* = 8.2 Hz, 1H), 7.13–7.03 (m, 1H).

##### 5-(*N*,*N*-Dimethylsulfamoyl)-4-fluoro-2-hydroxybenzoic Acid (**59**)

Intermediate **58** (1.141 g, 4.44 mmol) was dissolved in 10 mL of THF and added dropwise to an ice-cold solution of 2 M dimethylamine in THF (2.22 mL, 4.44 mmol) and DIPEA (2.34 mL, 13.31 mmol) in 35 mL of THF. The reaction mixture was stirred at 0 °C for 8 h. At reaction completion, the mixture was evaporated to dryness at low pressure and the residue was treated with a saturated NH_4_Cl aqueous solution (50 mL) and extracted twice with EtOAc (2 × 50 mL). The combined organic layers were dried over Na_2_SO_4_ and concentrated to dryness at low pressure to afford the pure **59** (823.9 mg, 70% yield) as a white solid. UPLC/MS: *R*_*t*_ = 1.19 min (gradient 1); MS (ESI) *m*/*z*: 262.0 [M – H]^−^. C_9_H_9_FNO_5_S [M – H]^−^ calcd: 262.0. ^1^H NMR (400 MHz, DMSO-*d*_6_): δ 8.15 (d, *J* = 8.2 Hz, 1H), 7.13–7.03 (m, 1H), 2.71 (d, *J* = 1.7 Hz, 6H).

##### Methyl 5-(*N*,*N*-Dimethylsulfamoyl)-4-fluoro-2-methoxybenzoate (**60**)

To an ice-cold solution of intermediate **59** (200 mg, 0.75 mmol) in DCM/MeOH 8:2 (9 mL) was carefully added trimethylsilyldiazomethane (2 M in hexanes, 1.13 mL, 2.26 mmol), and the reaction mixture was stirred at room temperature for 2 h. At reaction completion, the reaction mixture was quenched with 2 mL of a 1 M acetic solution in methanol and evaporated to dryness. The dry residue was suspended in a saturated NaHCO_3_ (15 mL) aqueous solution and extracted twice with EtOAc (2 × 15 mL). Purification by silica gel flash chromatography (cyclohexane/EtOAc from 85:15 to 70:30) afforded the pure **60** (201 mg, 92% yield) as a white solid. UPLC/MS: *R*_*t*_ = 1.75 min (gradient 1); MS (ESI) *m*/*z*: 292.1 [M + H]^+^. C_11_H_15_FNO_5_S [M + H]^+^ calcd: 292.0. ^1^H NMR (600 MHz, chloroform-*d*): δ 8.35 (d, *J* = 5.0 Hz, 1H), 6.94 (d, *J* = 8.0 Hz, 1H), 3.85 (s, 3H), 3.79 (s, 3H), 2.72 (s, 6H).

##### Methyl 5-(*N*,*N*-Dimethylsulfamoyl)-2-methoxy-4-((8,8,8-trifluorooctyl)amino)benzoate (**61**)

Compound **58** was synthesized following the general procedure 6 previously described using intermediate **58** (50 mg, 0.17 mmol) and amine **53a** (75.4 mg, 0.34 mmol) in dry 1,4-dioxane (0.85 mL). Purification by silica gel flash chromatography (cyclohexane/EtOAc from 80:15 to 75:25) afforded the pure **61** (64.9 mg, 84% yield) as a white solid. UPLC/MS: *R*_*t*_ = 2.65 min (gradient 1); MS (ESI) *m*/*z*: 455.3 [M + H]^+^. C_19_H_30_F_3_N_2_O_5_S [M + H]^+^ calcd: 455.2. ^1^H NMR (400 MHz, chloroform-*d*): δ 8.23 (s, 1H), 6.77 (t, *J* = 4.8 Hz, 1H), 6.10 (s, 1H), 3.97 (s, 3H), 3.84 (s, 3H), 3.22–3.16 (m, 2H), 2.75 (s, 6H), 2.14–2.04 (m, 2H), 1.72 (p, *J* = 7.1 Hz, 2H), 1.60–1.55 (m, 4H), 1.45 (dd, *J* = 5.0, 2.0 Hz, 2H), 1.41 (dd, *J* = 3.9, 2.6 Hz, 4H).

##### 5-(*N*,*N*-Dimethylsulfamoyl)-2-methoxy-4-((8,8,8-trifluorooctyl)amino)benzoic Acid (**41**)

To a solution of intermediate **61** (59 mg, 0.13 mmol) dissolved in tetrahydrofuran (1.3 mL) was added a 1 M LiOH aqueous solution (0.26 mL, 0.26 mmol), and the mixture was stirred at room temperature for 16 h. At reaction completion, the crude was portioned between EtOAc (10 mL) and an NH_4_Cl saturated solution (10 mL) and the layers were separated. The organic layer was dried over Na_2_SO_4_ and concentrated to dryness at low pressure. Trituration with cyclohexane afforded the pure **41** (41.2 mg, 72% yield) as a white solid. UPLC/MS: *R*_*t*_ = 1.16 min (gradient 1); MS (ESI) *m*/*z*: 439.5 [M – H]^−^. C_18_H_26_F_3_N_2_O_5_S [M – H]^−^ calcd: 439.2. ^1^H NMR (400 MHz, DMSO-*d*_6_): δ 7.98 (s, 1H), 6.65 (t, *J* = 5.2 Hz, 1H), 6.26 (s, 1H), 3.88 (s, 3H), 3.29–3.22 (m, 2H), 2.61 (s, 6H), 1.65–1.55 (m, 2H), 1.52–1.42 (m, 4H), 1.39–1.29 (m, 6H). ^13^C NMR (100 MHz, DMSO-*d*_6_): δ 165.30 (CO), 164.05 (Cq), 150.89 (Cq), 136.09 (CH), 127.71 (CF_3_, q, ^1^*J*_CF_ = 276.51 Hz), 107.42 (Cq), 106.69 (Cq), 94.30 (CH), 55.88 (OCH_3_), 42.19 (CH_2_), 37.27 (CH_2_, 2C), 32.34 (CH_2_, q, ^2^*J*_CF_ = 27.47 Hz), 28.11 (CH_2_), 27.90 (CH_2_), 27.79 (CH_2_), 26.13 (CH_2_), 21.32 (CH_2_). ^19^F NMR (565 MHz, DMSO-*d*_6_): δ −63.77 (t, *J* = 11.7 Hz). HRMS (AP-ESI) *m*/*z*: calcd for C_18_H_28_F_3_N_2_O_5_S [M + H]^+^, 441.1671; found, 441.441.1683.

##### 5-(*N*,*N*-Dimethylsulfamoyl)-2-hydroxy-4-((8,8,8-trifluorooctyl)amino)benzoic Acid (**40**)

Under an argon atmosphere, to an ice-cold solution of compound 41 (50 mg, 0.12 mmol) dissolved in DCM (1.2 mL) was added dropwise BBr_3_ (1 M in DCM, 0.59 mL, 0.59 mmol), and the mixture was stirred at room temperature for 16 h. At reaction completion, the mixture was cooled to 0 °C, quenched with 2 mL of methanol, and evaporated to dryness. The dry residue crude was then portioned between EtOAc (10 mL) and an NH_4_Cl saturated solution (10 mL), and the layers were separated. The organic layer was dried over Na_2_SO_4_ and concentrated to dryness at low pressure. Trituration with cyclohexane afforded the pure **40** (39.9 mg, 78% yield) as a white solid. UPLC/MS: *R*_*t*_ = 1.81 min (gradient 1); MS (ESI) *m*/*z*: 425.4 [M – H]^−^. C_17_H_24_F_3_N_2_O_5_S [M – H]^−^ calcd: 425.1. ^1^H NMR (400 MHz, chloroform-*d*): δ 10.87, (broad s, 1H) 8.22 (s, 1H), 6.84 (s, 1H), 6.15 (s, 1H), 3.15 (q, *J* = 6.6 Hz, 2H), 2.74 (s, 6H), 2.15–1.97 (m, 2H), 1.72–1.61 (m, 2H), 1.60–1.50 (m, 2H), 1.38 (s, 6H). ^13^C NMR (101 MHz, chloroform-*d*): δ 173.37 (Cq), 166.54 (CO), 152.94 (Cq), 136.11 (CH), 127.34 (CF_3_, q, ^1^*J*_CF_ = 277.43 Hz), 110.72 (Cq), 100.11 (Cq), 97.69 (CH), 43.37 (CH_2_), 37.85 (CH_2_, 2C), 33.81 (CH_2_, q, ^2^*J*_CF_ = 27.95 Hz), 29.00 (CH_2_), 28.71 (CH_2_), 28.64 (CH_2_), 26.92 (CH_2_), 21.94 (CH_2_). ^19^F NMR (565 MHz, DMSO-*d*_6_): δ −63.75 (t, *J* = 11.7 Hz). HRMS (AP-ESI) *m*/*z*: calcd for C_17_H_26_F_3_N_2_O_5_S [M + H]^+^, 427.1515; found, 427.1514.

##### Methyl 5-(*N*,*N*-Dimethylsulfamoyl)-2-hydroxy-4-((8,8,8-trifluorooctyl)amino)benzoate (**62**)

Under an argon atmosphere, to an ice-cold solution of intermediate **61** (50 mg, 0.11 mmol) dissolved in DCM (1.2 mL) was added dropwise BBr_3_ (1 M in DCM, 0.55 mL, 0.55 mmol), and the mixture was stirred at room temperature for 16 h. At reaction completion, the reaction mixture was cooled to 0 °C, quenched with 2 mL of methanol, and evaporated to dryness. The dry residue crude was then portioned between EtOAc (10 mL) and an NH_4_Cl saturated solution (10 mL), and the layers were separated. The organic layer was dried over Na_2_SO_4_ and concentrated to dryness at low pressure. Purification by silica gel flash chromatography (cyclohexane/EtOAc 95:05) afforded the pure **62** (40.2 mg, 83% yield) as a white solid. UPLC/MS: *R*_*t*_ = 2.10 min (gradient 1); MS (ESI) *m*/*z*: 441.3 [M – H]^+^. C_18_H_28_F_3_N_2_O_5_S [M + H]^+^ calcd: 441.1. ^1^H NMR (400 MHz, chloroform-*d*): δ 11.26 (s, 1H), 8.17 (s, 1H), 6.73 (t, *J* = 4.6 Hz, 1H), 6.16 (s, 1H), 3.92 (s, 3H), 3.16 (q, *J* = 7.1, 5.0 Hz, 2H), 2.75 (s, 6H), 2.15–1.99 (m, 2H), 1.74–1.63 (m, 2H), 1.62–1.54 (m, 2H), 1.48–1.35 (m, 6H).

##### Methyl 5-(*N*,*N*-Dimethylsulfamoyl)-2-ethoxy-4-((8,8,8-trifluorooctyl)amino)benzoate (**63a**)

To a solution of intermediate **62** (31.8 mg, 0.07 mmol) in acetonitrile (0.7 mL) were added ethyl iodide (10 μL, 0.11 mmol) and potassium carbonate (15 mg, 0.11 mmol), and the reaction mixture was stirred at 80 °C temperature for 4 h. At reaction completion, the crude was portioned between EtOAc (10 mL) and water (10 mL) and the layers were separated. The organic layer was dried over Na_2_SO_4_ and concentrated to dryness at low pressure. Purification by silica gel flash chromatography (cyclohexane/EtOAc from 100:00 to 80:20) afforded the pure **63a** (25.6 mg, 78% yield) as a white solid. UPLC/MS: *R*_*t*_ = 1.85 min (gradient 1); MS (ESI) *m*/*z*: 469.3 [M + H]^+^. C_20_H_32_F_3_N_2_O_5_S [M + H]^+^ calcd: 469.2. ^1^H NMR (400 MHz, chloroform-*d*): δ 8.20 (s, 1H), 6.71 (t, *J* = 4.8 Hz, 1H), 6.07 (s, 1H), 4.14 (q, *J* = 7.0 Hz, 2H), 3.82 (s, 3H), 3.18–3.11 (m, 2H), 2.72 (s, 6H), 2.13–1.99 (m, 2H), 1.73–1.64 (m, 2H), 1.61–1.53 (m, 2H), 1.51 (t, *J* = 6.9 Hz, 3H), 1.48–1.35 (m, 6H).

##### 5-(*N*,*N*-Dimethylsulfamoyl)-2-ethoxy-4-((8,8,8-trifluorooctyl)amino)benzoic Acid (**42**)

To a solution of compound **63a** (25.6 mg, 0.05 mmol) in tetrahydrofuran (0.5 mL) was added a 1 M LiOH aqueous solution (0.27 mL, 0.27 mmol), and the reaction mixture was stirred at room temperature for 16 h. At reaction completion, the crude was portioned between EtOAc (10 mL) and an NH_4_Cl saturated solution (10 mL) and the layers were separated. The organic layer was dried over Na_2_SO_4_ and concentrated to dryness at low pressure. Trituration with cyclohexane afforded the pure **42** (19.54 mg, 86% yield) as a white solid. UPLC/MS: *R*_*t*_ = 1.32 min (gradient 1); MS (ESI) *m*/*z*: 453.3 [M – H]^−^. C_19_H_28_F_3_N_2_O_5_S [M – H]^−^ calcd: 453.2. ^1^H NMR (400 MHz, DMSO-*d*_6_): δ 7.95 (s, 1H), 6.62 (t, *J* = 5.2 Hz, 1H), 6.23 (s, 1H), 4.15 (q, *J* = 6.9 Hz, 2H), 3.23 (q, *J* = 6.5 Hz, 2H), 2.60 (s, 6H), 2.29–2.14 (m, 2H), 1.63–1.52 (m, 2H), 1.51–1.42 (m, 2H), 1.40–1.25 (m, 9H). ^13^C NMR (101 MHz, DMSO-*d*_6_): δ 166.08 (CO), 163.69 (Cq), 151.13 (Cq), 136.45 (CH), 128.31 (CF_3_, q, ^1^*J*_CF_ = 277.07 Hz), 107.92 (Cq), 107.57 (Cq), 95.35 (CH), 64.59 (OCH_2_), 42.61 (CH_2_), 37.77 (CH_3_, 2C), 32.80 (CH_2_, q, ^2^*J*_CF_ = 26.87 Hz), 28.61 (CH_2_), 28.37 (CH_2_), 28.27 (CH_2_), 26.59 (CH_2_), 21.80 (CH_2_), 14.83 (CH_3_). ^19^F NMR (565 MHz, DMSO-*d*_6_): δ −63.77 (t, *J* = 11.7 Hz). HRMS (AP-ESI) *m*/*z*: calcd for C_19_H_30_F_3_N_2_O_5_S [M + H]^+^, 455.1828; found, 455.1852.

##### Methyl 2-(Cyclopentyloxy)-5-(*N*,*N*-dimethylsulfamoyl)-4-((8,8,8-trifluorooctyl)amino)benzoate (**63b**)

To a solution of intermediate **12.4** (30.0 mg, 0.07 mmol) in acetonitrile (0.7 mL) were added cyclopentyl bromide (15 μL, 0.13 mmol) and potassium carbonate (28.3 mg, 0.20 mmol), and the reaction mixture was stirred at 80 °C for 4 h. At reaction completion, the crude was portioned between EtOAc (10 mL) and water (10 mL) and the layers were separated. The organic layer was dried over Na_2_SO_4_ and concentrated to dryness at low pressure. Purification by silica gel flash chromatography (cyclohexane/EtOAc from 100:00 to 90:10) afforded the pure titled compound (25.6 mg, 72% yield) as a white solid. UPLC/MS: *R*_*t*_ = 2.30 min (gradient 2); MS (ESI) *m*/*z*: 509.2 [M + H]^+^. C_23_H_36_F_3_N_2_O_5_S [M + H]^+^ calcd: 509.6. ^1^H NMR (400 MHz, chloroform-*d*): δ 8.19 (s, 1H), 6.69 (t, *J* = 4.8 Hz, 1H), 6.07 (s, 1H), 4.88–4.81 (m, 1H), 3.80 (s, 3H), 3.19–3.10 (m, 2H), 2.72 (s, 6H), 2.13–1.99 (m, 2H), 1.99–1.92 (m, 4H), 1.91–1.81 (m, 2H), 1.73–1.62 (m, 2H), 1.61–1.51 (m, 2H), 1.49–1.34 (m, 6H).

##### 2-(Cyclopentyloxy)-5-(*N*,*N*-dimethylsulfamoyl)-4-((8,8,8-trifluorooctyl)amino)benzoic Acid (**43**)

To a solution of intermediate **63b** (25.6 mg, 0.05 mmol) dissolved in tetrahydrofuran (0.25 mL) was added a 1 M LiOH aqueous solution (0.5 mL, 0.25 mmol), and the mixture was stirred at room temperature for 16 h. At reaction completion, the crude was portioned between EtOAc (10 mL) and an NH_4_Cl saturated solution (10 mL) and the layers were separated. The organic layer was dried over Na_2_SO_4_ and concentrated to dryness at low pressure. Trituration with cyclohexane afforded the pure **43** (16.3 mg, 66% yield) as a white solid. UPLC/MS: *R*_*t*_ = 1.80 min (gradient 1); MS (ESI) *m*/*z*: 493.3 [M – H]^−^. C_22_H_32_F_3_N_2_O_5_S [M – H]^−^ calcd: 493.2. ^1^H NMR (400 MHz, chloroform-*d*): δ 8.40 (s, 1H), 6.94 (s, 1H), 6.12 (s, 1H), 5.09–5.03 (m, 1H), 3.20–3.13 (m, 2H), 2.75 (s, 6H), 2.14–1.97 (m, 5H), 1.93–1.81 (m, 2H), 1.81–1.65 (m, 4H), 1.61–1.51 (m, 4H), 1.50–1.33 (m, 6H). ^13^C NMR (101 MHz, DMSO-*d*_6_): δ 165.50 (CO), 162.32 (Cq), 150.52 (Cq), 136.17 (CH), 127.71 (CF_3_, q, ^1^*J*_CF_ = 277.34 Hz), 107.51(Cq), 107.35 (Cq), 95.96 (CH), 79.90 (CH), 42.12 (CH_2_), 37.27 (CH_3_, 2C), 32.34 (CH_2_, q, ^2^*J*_CF_ = 27.66 Hz), 32.27 (CH_2_, 2C), 28.16 (CH_2_), 27.91 (CH_2_), 27.79 (CH_2_), 26.09 (CH_2_), 23.64 (CH_2_, 2C), 21.31 (CH_2_). ^19^F NMR (565 MHz, DMSO-*d*_6_): δ −63.78 (t, *J* = 11.7 Hz). HRMS (AP-ESI) *m*/*z*: calcd for C_22_H_34_F_3_N_2_O_5_S [M + H]^+^, 495.2141; found, 495.2151.

### Pharmacophore Generation and Screening

Unselective NKCC1 inhibitors, namely, bumetanide, benzmetanide, furosemide, and piretanide, and all our selective NKCC1 inhibitors were designed and prepared within the Schrodinger suite 2019-4 in order to retrieve the most favorable tautomerization and protonation states at physiological pH. Provided that the binding mode of such inhibitors is unknown, we performed an extensive conformational search using MacroModel utility to identify the most stable conformation of bumetanide in water. Multiple pharmacophore hypotheses were generated building upon the 3D structural arrangements of bumetanide substituents via Phase,^44,45^ such that each hypothesis comprised a unique set of features (e.g., HB donors, HB acceptors, and hydrophobic and aromatic groups). Then, we screened the other unselective inhibitors into each pharmacophore model and ranked the compounds according to their Phase score. One pharmacophore model (i.e., model A) was selected based on its ability to discriminate between more and less potent NKCC1 unselective inhibitors. Finally, our selective NKCC1 inhibitors were fitted into the model A via Phase, ranked by their Phase score and visually inspected to analyze the overlap with the pharmacophore features.

## Biology

### HEK Cell Culture and Transfection

HEK293 cells were cultured in Dulbecco’s modified Eagle’s medium (DMEM) supplemented with 10% fetal bovine serum, 1% l-glutamine, 100 U/mL penicillin, and 100 μg/mL streptomycin and maintained at 37 °C in a 5% CO_2_ humidified atmosphere. To assess NKCC1 and NKCC2 activity (Cl^–^ influx assay), 3 million HEK cells were plated in a 10 cm cell-culture dish and transfected with a transfection mixture comprising 5 mL of DMEM, 4 mL of Opti-MEM, 8 μg of DNA plasmid coding for NKCC1 (PRK-NKCC1 obtained from Medical Research Council and the University of Dundee), NKCC2 (OriGene plasmid #RC216145) subcloned in PRK5 plasmid, or mock control (empty vector), together with 8 μg of a plasmid coding for the Cl^–^-sensitive variant of the membrane-targeted fluorescent protein YFP, mbYFPQS (Addgene plasmid #80742),^53^ and 32 μL of Lipofectamin 2000. To assess KCC2 activity (Tl influx assay), cells were transfected with 8 μg of KCC2^54^ subcloned in the PRK5 plasmid or mock control (empty vector) and 16 μL of Lipofectamin 2000. After 4 h, the cells were collected and plated in 96-well black-walled, clear-bottomed plates at a density of 2.5 × 10^4^. After 48 h, cells were used for the Cl^–^ or Tl influx assays. All reagents were purchased from Life Technologies, unless otherwise specified.

### Cl^–^ Influx Assay in HEK Cells

Transfected cells were treated with 10 μM or 100 μM bumetanide (as positive controls), DMSO (as the negative control), or with each of our compounds in 100 μL/well of a Cl^–^-free hypotonic solution (67.5 mM Na^+^ gluconate, 2.5 mM K^+^ gluconate, 15 mM HEPES pH 7.4, 50 mM glucose, 1 mM Na_2_HPO_4_, 1 mM NaH_2_PO_4_, 1 mM MgSO_4_, and 1 mM CaSO_4_). After 30 min of incubation, plates were loaded into a Victor 3V (PerkinElmer) multiplate reader equipped with an automatic liquid injector system, and fluorescence of Cl^–^-sensitive mbYFPQS was recorded with excitation at 485 nm and emission at 535 nm. For each well, fluorescence was first recorded for 20 s of the baseline and for 60 s after delivery of a NaCl concentrated solution (74 mM final concentration in the assay well). Fluorescence of Cl^–^-sensitive mbYFPQS is inversely correlated to the intracellular Cl^–^ concentration;^53^ therefore, chloride influx into the cells determined a decrease in mbYFPQS fluorescence. To represent the fluorescent traces in time, we normalized the fluorescence value for each time point to the average of the fluorescence value of the first 20 s of the baseline (Δ*F*/*F*_0_). To quantify the average effects as represented by the bar plots, we expressed the decrease in fluorescence upon NaCl application as the average of the last 10 s of Δ*F*/*F*_0_ normalized traces. Moreover, for each experiment, to account for the contribution of Cl^–^ changes that were dependent on transporters/exchangers other than NKCC1 or NKCC2, we subtracted the value of the last 10 s of Δ*F*/*F*_0_ normalized traces obtained from mock-transfected cells (either control or treated) from the respective Δ*F*/*F*_0_ value obtained from the cells transfected with NKCC1 or NKCC2. We then presented in the figures all the data as a percentage of the fluorescence decrease versus the value of the control DMSO.

### Tl Influx Assay in HEK Cells

The thallium influx assay (FluxOR Potassium Ion Channel Assay, Life Technologies) was modified from previously published protocols.^55^ In this assay, the amount of Tl ions (as a substitute for K^+^) entering the cells by KCC2 transport is detected with a Tl-sensitive fluorogenic dye that increases fluorescence upon Tl binding. Cell were loaded with a 1:1000 dilution of Tl-sensitive fluorogenic dye (component A) and 1:100 probenecid (component D) in 100 μL/well Cl^–^ free-hypotonic solution (as above). After 1 h, cells were washed twice with 100 μL/well of hypotonic solution and treated with DIOA (Sigma, positive control), DMSO (as negative control), and **40** diluted in 200 μL/well hypotonic solution and in the presence of 100 μM ouabain to inhibit transport by the Na^+^/K^+^-ATPase pump. The plates were loaded onto a Spark (Tecan) multiplate reader equipped with a double automatic liquid injector system. Fluorescence of Tl-sensitive dye was recorded with excitation at 485 nm and emission at 535 nm. For each well, fluorescence was first recorded for 20 s of the baseline, for another 20 s after Tl_2_SO_4_ delivery (2 mM final concentration in the assay well), and for 40 s after delivery of a NaCl concentrated solution (74 mM final concentration in the assay well). To represent the fluorescent traces in time, we normalized the fluorescence value for each time point to the average of the fluorescence value of the first 20 s of the baseline (Δ*F*/*F*_0_). To quantify the average effects as represented by the bar plots, we expressed the increase in fluorescence upon NaCl application as the average of the last 10 s of Δ*F*/*F*_0_ normalized traces after subtracting the average of the last 10 s of Δ*F*/*F*_0_ normalized traces after Tl_2_SO_4_ injection. Moreover, for each experiment, to account for the contribution of Tl influx that was dependent on transporters/exchangers other than KCC2, we subtracted the value of the last 10 s of Δ*F*/*F*_0_ normalized traces obtained from mock-transfected cells (either control or treated) from the respective Δ*F*/*F*_0_ value obtained from the cells transfected with KCC2. We then presented in the figures all the data as a percentage of the fluorescence increase versus the value of the control DMSO.

### Neuron Cultures

To perform the Ca^2+^-influx assay, primary neuronal cultures of dissociated hippocampal neurons were prepared from E18 C57BL/6J mice (Charles River) as previously described^56^ and plated in 96-well black-walled, clear-bottomed plates coated with poly-l-lysine (Sigma; 0.1 mg/mL in 100 mM borate buffer, pH 8.5) at a density of 30,000 cells per well. Neurons were maintained in neurobasal medium supplemented with 2% B-27 supplement, 0.5 mM glutamine, 50 U/mL of penicillin, and 50 μg/mL of streptomycin (all from Gibco). The cells were incubated at 37 °C and 5% CO_2_ until DIV 3 for the Ca^2+^ influx assay.

### Ca^2+^ Influx Assay in Neuronal Cultures

At 3 DIVs, neurons were loaded with 2.5 μM of the Ca^**2+**^-sensitive day Fluo4-AM (Invitrogen) in extracellular solution (145 mM NaCl, 5 mM KCl, 10 mM HEPES, 5.55 mM glucose, 1 mM MgCl_2_, and 2 mM CaCl_2_, pH 7.4). After 15 min, cells were washed twice with extracellular solutions and treated with bumetanide (as a positive control), DMSO (as a negative control), or each of our compounds at 10 μM or 100 μM, in extracellular solution for 15 min. Plates were then loaded onto a Spark (Tecan) multiplate reader equipped with an automatic liquid injector system, and Fluo4 fluorescence was recorded with excitation at 485 nm and emission at 535 nm. For each well, fluorescence was first recorded for 20 s of the baseline and for 20 s after delivery of GABA (100 μM). To evaluate neuronal viability, fluorescence was recorded for an additional 20 s after delivery of a depolarizing KCl stimulus (90 mM final concentration in wells). To represent the fluorescent traces in time, we normalized the fluorescence value for each time point to the average of the fluorescence value for the first 20 s of the baseline (Δ*F*/*F*_0_). To quantify the average effects as represented by the bar plots, we measured the maximum peak increase in Δ*F*/*F*_0_ fluorescence upon GABA application normalized over the maximum peak increase in Δ*F*/*F*_0_ fluorescence upon KCl application. We then presented in the figures all the data as a percentage of the fluorescence increase versus the value of the control DMSO.

### Animals

All animal procedures were approved by IIT licensing in compliance with the Italian Ministry of Health (D.Lgs 26/2014) and EU guidelines (Directive 2010/63/EU). A veterinarian was employed to maintain the health and comfort of the animals. Mice were housed in filtered cages in a temperature-controlled room with a 12:12 h dark/light cycle and with ad libitum access to water and food. All efforts were made to minimize animal suffering and use the lowest possible number of animals required to produce statistical relevant results, according to the “3Rs concept”. In this study, we used Ts65Dn mice maintained in their original genetic background^57^ by crossing (more than 40 times) Ts65Dn female to C57BL/6JEi × C_3_SnHeSnJ (B6EiC3) F1 males (Jackson Laboratories). Ts65Dn mice were genotyped by PCR as previously described.^58^ Only males were used for behavioral experiments. Ts65Dn and controls were randomly assigned to vehicle groups (2% DMSO in saline), bumetanide (Sigma, 0.6 mg kg^–1^ body weight), or **40** (0.6 mg kg^–1^ body weight) and treated daily for ∼21 days by intraperitoneal injection (i.p.). On the day of behavioral testing, injection was performed 1 h before the task began.

### Compound Preparation for *In Vivo* Experiments

For the *in vivo* experiments, bumetanide and **40** were dissolved in DMSO in a stock solution of 3 mg/mL. On the day of the injection, the stock solution was dissolved in PBS at a concentration of 0.06 mg/mL and injected in a volume of 10 μL g^–1^ to have a final concentration of 0.6 mg kg^–1^.

### Diuresis Analysis

Diuresis analysis was performed using mouse metabolic cages (Tecniplast, 3600m021) equipped with a grid over a funnel and a plastic cone for the separate collection of urine and feces. Immediately after i.p. treatment with vehicle, bumetanide, or **40** at 0.6 mg kg^–1^, animals were placed inside the metabolic cages (one animal per cage) where food and water were available ad libitum. After 2 h, mice were returned to their home cages, and the urine volume was measured.

### Behavioral Testing

Ts65Dn male mice (8–16 weeks old) were tested after 1 week of treatment with vehicle or **40** (0.6 mg kg^–1^ i.p.). The battery of tests was run over a total period of ∼14 days (four behavioral tests with the following order Open Field, NOR, T-maze, and CFC). During the days of behavioral testing, animals were treated daily with the drug, with tests beginning 1 h after injection. The tasks were video-recorded and then analyzed manually by a blind operator. After each trial or experiment, the diverse apparatus and objects were cleaned with 70% ethanol. *T-maze*. The T-maze is a black opaque plastic apparatus with a starting arm and two perpendicular goal arms, each equipped with a sliding door and evenly illuminated by overhead red lighting (12–14 lux). The T-maze test (spontaneous alteration protocol, 11 trials) evaluates short-term memory by analyzing the correct choice of the unexplored arm. The test was performed in a similar way to that previously conducted on Ts65Dn mice.^59^ In each trial, a mouse was first placed in the starting chamber for 20 s. Then, the sliding door was removed and the animal was free to explore the apparatus. When the mouse entered (with all four limbs) one of the two goal arms, the opposite arm was closed with the sliding door. When the mouse (free to explore the remaining part of the apparatus) returned to the starting area, the previously closed goal arm was opened. The trial was repeated 11 times. Entry into a goal arm opposite the one previously chosen was considered a correct choice, while entry into the previously explored arm was considered an incorrect choice. The alternation score was calculated as the percentage of correct choices (i.e., left–right or right–left) over the total number of the 10 possible alternations. *Open field test*. The test evaluates the locomotor activity of mice by measuring the total distance traveled and the average walking speed during the free exploration. The test was performed in a gray acrylic arena (44 × 44 cm), evenly illuminated by overhead red lighting (12–14 lux) and consists in the day 1 of the NOR test. Mice were allowed to freely explore the arena for 15 min. Animal tracking and extrapolation of locomotor activity parameters were performed using Any-Maze software (Stoelting Co.). *NOR test*. The test evaluates long-term object recognition memory by measuring the ability of mice to recognize a new object with respect to familiar objects. The test was performed in a gray acrylic arena (44 × 44 cm), evenly illuminated by overhead red lighting (12–14 lux). On day 1, mice were habituated to the arena by freely exploring the chamber for 15 min. On day 2, during the acquisition phase, mice were free to explore three different objects (different in color, size, shape, and material) for 15 min. After 24 h, one object from the acquisition phase was replaced with a novel object, and the mice were tested for 15 min for their ability to recognize the new object. The time spent for exploring each object was defined as the number of seconds during which mice showed investigative behavior (i.e., head orientation, sniffing occurring within <1.0 cm) or clear contact between the object and the nose. The time spent for exploring each object, expressed as a percentage of the total exploration time, was measured for each trial. The discrimination index was calculated as the difference between the percentages of time spent for investigating the novel object and investigating the familiar objects: discrimination index = (novel object exploration time/total exploration time * 100) – (familiar object exploration time/total exploration time * 100). As a control, we monitored object preference during the acquisition phase and exploration time in the acquisition phase and trial phase. Of note, although there was a significant preference for the object B in the WT vehicle-treated group (vs both object A and object C) and in the Ts65Dn 40-treated group (only vs object A) (Table S2), the objects were randomly replaced with the new one for the test phase, thus not affecting the final result. *Contextual fear conditioning test (CFC)*. The test evaluates the long-term associative memory by measuring the freezing time of the animals placed in a location where they had received an adverse stimulus (electric shock) 24 h earlier. The experiments were performed in a fear-conditioning system (TSE), which is a transparent acrylic conditioning chamber (23 × 23 cm) equipped with a stainless-steel grid floor. Mice were placed outside the experimental room in their home cages before the test and individually transported to the TSE apparatus in standard cages. Mice were placed in the conditioning chamber, and they received one electric shock (2 s, 0.75 mA constant electric current) through the floor grid 3 min later. Mice were removed 15 s after the shock. After 24 h, mice were placed in the same chamber for 3 min. After 2 h, they were moved to a new context (black chamber with plastic gray floor and vanilla odor). The time spent for freezing was scored and expressed as the percentage of the total time analyzed. Of note, although we observed a significant higher freezing response in Ts65Dn mice compared to WT mice during the new context phase, the freezing seconds are significantly below the threshold for the animal exclusion (i.e., 30 s), thus indicating that there is no high nonassociative freezing affecting the test results. For behavioral experiments, we adopted the following exclusion criteria independent of genotype or treatment (before blind code was broken). In the T-maze test, we excluded mice that did not conclude the 10 trials within 20 min of the test. In the CFC test, we excluded mice showing very high nonassociative freezing in the new context. This was defined as more than 30 s freezing during the 3 min test. In the NOR test, we excluded animals showing very low explorative behavior. This was defined as less than 20 s of direct object exploration during the 15 min test. Following these criteria, a total of 11 mice among the NOR, CFC, and T-maze tests were excluded.

### Statistical Analysis

The results are presented as the means ± SEM. The statistical analysis was performed using SigmaPlot 13.0 (Systat) software. Where appropriate, the statistical significance was assessed using the following parametric test: Student’s *t*-test, one-way ANOVA followed by the Dunnet post hoc test, two-way ANOVA followed by the all pairwise Tukey post hoc test. Where normal distribution or equal variance assumptions were not valid, statistical significance was evaluated using the Mann–Whitney rank sum test, Kruskal–Wallis one-way ANOVA with Dunn’s post hoc test, or two-way ANOVA on ranks followed by the all pairwise Dunn’s post hoc test. *P* values <0.05 were considered significant. Outliers were excluded only from the final pool of data by a Grubb’s test run iteratively until no outliers were found.

## Supporting Information Available

Procedures for aqueous kinetic solubility, *in vitro* metabolic stability, and *in vitro* plasmatic stability; pharmacophore fitting of unselective bumetanide derivatives; dose-response curve; ranking of NKCC1 inhibitors according to the phase score; control parameters in the NOR and CFC tasks of WT and Ts65Dn mice; ^1^H NMR, ^13^C NMR, and ^19^F NMR spectra; and chromatography analysis of final compounds (PDF)
Molecular formula strings (CSV)

## Abbreviations

Bume: bumetanide
[Cl^–^]_i_: intracellular chloride concentration
CFC: contextual fear conditioning
CNS: central nervous system
DCM: dichloromethane
DMF: *N*,*N*-dimethylformamide
DMSO: dimethyl sulfoxide
DIPEA: *N*,*N*-diisopropylethylamine
DS: Down syndrome
EtOH: ethanol
MeOH: methanol
NOR: novel object recognition
TEA: triethylamine
TMS-CHN_2_: trimethylsilyldiazomethane
SAR: structure–activity relationship
WT: wild type
