# Supplementary material for: Design, Synthesis, In Vitro and In Vivo Characterization of Selective NKCC1 Inhibitors for the Treatment of Core Symptoms in Down Syndrome
Source: J Med Chem. 2021 Jun 17;64(14):10203–29. doi: 10.1021/acs.jmedchem.1c00603 (PMC8311653; doi:10.1021/acs.jmedchem.1c00603)
Supplement: Supplementary file 1 — jm1c00603_si_001.pdf [file jm1c00603_si_001.pdf]

SUPPORTING INFORMATION

Design, synthesis, *in vitro* and *in vivo* characterization of selective NKCC1 inhibitors for the treatment of core symptoms in Down syndrome and other brain disorders.

Marco Borgogno[1,#], Annalisa Savardi[2,3#], Jacopo Manigrasso[1], Alessandra Turci[2,4], Corinne Portioli[1,2], Giuliana Ottonello,[5] Sine Mandrup Bertozzi[5], Andrea Armirotti[5], Andrea Contestabile[2], Laura Cancedda[2,3,*] and Marco De Vivo[1,*]

1: Molecular Modeling and Drug Discovery Laboratory, Istituto Italiano di Tecnologia, via Morego, 30, 16163 Genoa, Italy.

2: Brain Development and Disease Laboratory, Istituto Italiano di Tecnologia, via Morego, 30, 16163 Genoa, Italy.

3: Dulbecco Telethon Institute, Italy.

4: Università degli Studi di Genova, Via Balbi, 5, 16126 Genoa, Italy

5: Analytical Chemistry Facility, Istituto Italiano di Tecnologia, via Morego, 30, 16163 Genoa, Italy.

*Correspondence should be addressed to:

Dr. Marco De Vivo, email: marco.devivo@iit.it;

Dr. Laura Cancedda, email: laura.cancedda@iit.it

Table of contents:

- 1. Aqueous kinetic solubility S3
- 2. *In vitro* mouse plasma stability S3
- 3. *In vitro* mouse microsomial stability S4
- 4. Figure S-1 S5
- 5. Figure S-2 S6
- 6. Table S-1 S7
- 7. Table S-2 S8
- 8. ^{1}H, ^{13}C and ^{19}F NMR of final compounds S9
- 9. Chromatographic analysis of final compounds S61

1. Aqueous kinetic solubility.

30 μL aliquots of 10 mM DMSO stock solution of the test compound were incubated in Phosphate Buffered Saline (PBS) at pH 7.4 at 25°C for 24h. After centrifugation the dissolved compound in the supernatant was quantified by LC-MS/MS. Target concentration was 250 μM resulting in a final concentration of 2.5% DMSO. Aqueous kinetic solubility was determined by UV quantification at 215 nm. Kinetic solubility was then calculated by dividing the peak area of the supernatant by the peak area of the reference and multiply by the reference concentration (μM) and dilution factor (1.25).

2. *In vitro* mouse plasma stability.

Compounds were diluted in preheated (37 °C) mouse plasma (Rockland Immunochemicals Inc.) with 5% DMSO to favor solubilization. The final compound concentration was 2.0 μM. At time points (0, 5, 15, 30, 60, 120 min), a 30 μL aliquot of the incubation solution was diluted in 200 μL of cold acetonitrile spiked with 200 nM Warfarin, as internal standard. After vortexing for 30 s, the solution was centrifuged at 3500g for 15 min at 4 °C, and the supernatant transferred for LC-MS/MS analysis on a Waters Acquity UPLC/MS TQD system. Compound stability was evaluated on the basis of the corresponding peak areas plotted *vs* time. The compounds' half-lives were calculated using a one-phase fitting decay of the peak area *vs* time profiles.

3. *In vitro* mouse microsomial stability.

10mM DMSO stock solution of test compound was pre-incubated at 37 ˚C for 15 min with liver microsomes (Sekisui Xenotech, LCC), 0.1M Tris-HCl buffer (pH 7.4), and 10% DMSO. The final concentration was 4.6 μM. After pre-incubation, the cofactors (NADPH, G6P, G6PDH, $MgCl_2$ pre-dissolved in 0.1M Tris-HCl) were added to the incubation mixture and the incubation was continued at 37 ˚C for 1h. At each time point (0, 5, 15, 30, 60 min), 30μL of incubation mixture was diluted with 200 μL cold acetonitile spiked with 200 nM of warfarin as internal standard, followed by centrifugation at 3500 g for 30 min. The supernatant was further diluted with H2O (1:1) for analysis.

An aliquot of 200 µl of the supernatant was removed, and the concentration of the test compound was quantified by LC-MS/MS. The percentage of the test compound remaining at each time point relative to t=0 was calculated. The half-lives ($t^{1/2}$) were determined by a one-phase decay equation using a non-linear regression of compound concentration *vs* time.

4. Figure S-1

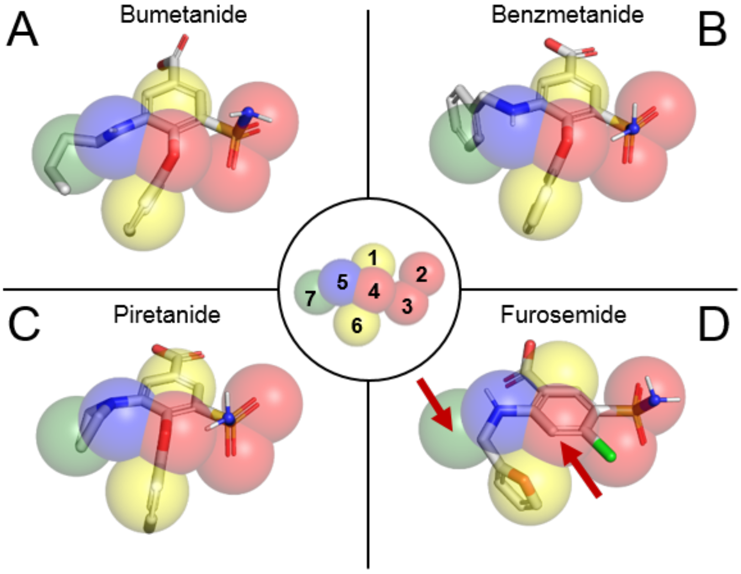

Figure S-1. Pharmacophore fitting of unselective bumetanide derivatives. A-D) Overlap of bumetanide, benzmetanide, piretanide and furosemide (white sticks) onto the pharmacophore model (transparent spheres). Red arrows indicate features' mismatch, while in the middle circle is highlighted the pharmacophore hypothesis. Furosemide has the higher IC_{50} toward NKCC1 within the series of derivatives and shows a different fit into the model, lacking both the HB-acceptor in position 4 and the hydrophobic group in position 7.

5. Figure S-2

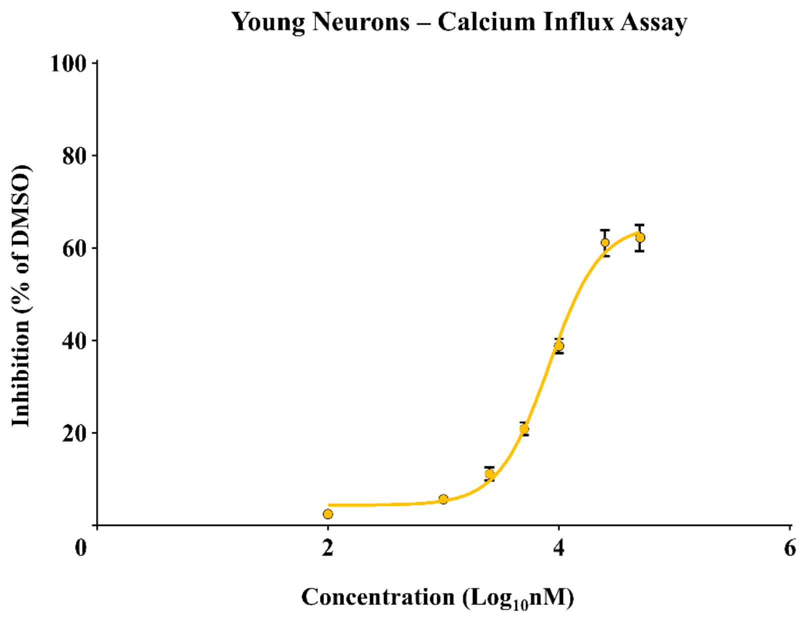

Figure S-2. Dose-response curve of 40 in young neurons with calcium influx assay.

6. Table S-1

compound	phase score		
27	1.62	4	1.50
7	1.56	22	1.50
5	1.56	20	1.50
11	1.56	25	1.49
15	1.56	3	1.49
23	1.56	42	1.49
9	1.54	40	1.49
30	1.54	37	1.47
19	1.54	1	1.47
16	1.53	21	1.46
10	1.52	28	1.46
6	1.51	43	1.46
17	1.51	32	1.45
31	1.51	34	1.45
8	1.51	33	1.44
18	1.51	41	1.44
13	1.51	44	1.44
12	1.51	36	1.42
14	1.51	38	1.41
29	1.51	39	1.13
26	1.51	35	1.07
24	1.50		

Table S-1. Ranking of NKCC1 inhibitors according to the phase score.

7. Table S-2.

% Object preference NOR

Objects	WT Vehicle	WT 40	Ts65Dn Vehicle	Ts65Dn 40	F and P (among groups)
**A**	27.58 ± 1.73	32.81 ± 2.60	30.95 ± 2.49	28.41 ± 2.91	Two-way ANOVA $F_{interaction\ (1,46)} = 2.590$, P=0.114
**B**	39.48 ± 2.19	34.35 ± 3.00	31.72 ± 1.83	38.58 ± 2.14	Two-way ANOVA $F_{interaction\ (1,46)} = 6.699$, P=0.013 Tukey *post hoc* test Within Vehicle WT vs Ts P=0.018 Within Ts Vehicle vs 40 P=0.045
**C**	32.94 ± 1.68	32.84 ± 2.67	37.33 ± 3.21	33.01 ± 2.06	Two-way ANOVA $F_{interaction\ (1,46)} = 0.764$, P=0.387
**F and P (among objects)**	One Way ANOVA $F_{interaction\ (2,42)} =$ 10.047, P<0.001 B vs A P<0,001 B vs C P=0.047	One Way ANOVA $F_{interaction\ (2,30)} =$ 0.101, P=0.904	One Way ANOVA $F_{interaction\ (2,33)} =$ 1.831, P=0.176	One Way ANOVA $F_{interaction\ (2,33)} =$ 4.496, P=0.019 B vs A P=0.014	

Total Exploration Time (sec) NOR

	WT Vehicle	WT 40	Ts65Dn Vehicle	Ts65Dn 40	F and P
**Acquisition**	107.30 ± 7.46	104.90 ± 7.89	85.96 ± 7.94	120.20 ± 14.5	Two-way ANOVA $F_{interaction\ (1,46)} = 3.477$, P=0.069
**Trial**	74.85 ± 5.79	76.64 ± 10.17	68.54 ± 5.67	76.14 ± 7.30	Two-way ANOVA $F_{interaction\ (1,46)} = 0.161$, P=0.690

Freezing Time (%) CFC

	WT Vehicle	WT 40	Ts65Dn Vehicle	Ts65Dn 40	F and P
**Pre Shock**	3.38 ± 0.42	2.34 ± 0.34	2.98 ± 0.65	2.75 ± 0.38	Two-way ANOVA $F_{interaction\ (1,44)} = 0.736$, P=0.396
**Post Shock**	53.75 ± 5.33	53.44 ± 6.88	59.28 ± 2.99	62.18 ± 4.18	Two-way ANOVA on Ranks $F_{interaction\ (1,44)} = 0.0693$, P=0.794
**New Context**	5.35 ± 0.96	3.33 ± 1.20	8.65 ± 1.10	7.34 ± 1.32	Two-way ANOVA $F_{genotype\ (1,44)} = 10.143$, P=0.003 Within Vehicle WT vs Ts P=0.044 Within 40 WT vs Ts P=0.019

Table S-2. Control parameters in the NOR and CFC tasks of WT and Ts65Dn mice.

8. ^{1}H, ^{13}C and ^{19}F Spectra of final compounds.

NMR experiments were run on a Bruker AVANCE III 400 system (400.13 MHz for ^{1}H and 100.62 MHz for 13C), equipped with a BBI probe and Z-gradients, and Bruker FT NMR AVANCE III 600 MHz spectrometer equipped with a 5mmCryoProbe QCI ^{1}H/^{19}F−^{13}C/^{15}N−D quadruple resonance, a shielded Z-gradient coil and the automatic sample changer SampleJet NMR system (600 MHz for 1H, 151 MHz for ^{13}C, and 565 MHz for ^{19}F). Spectra were acquired at 300 K, using deuterated dimethylsulfoxide (DMSO–d_6) or deuterated chloroform ($CDCl_3$) as solvents.

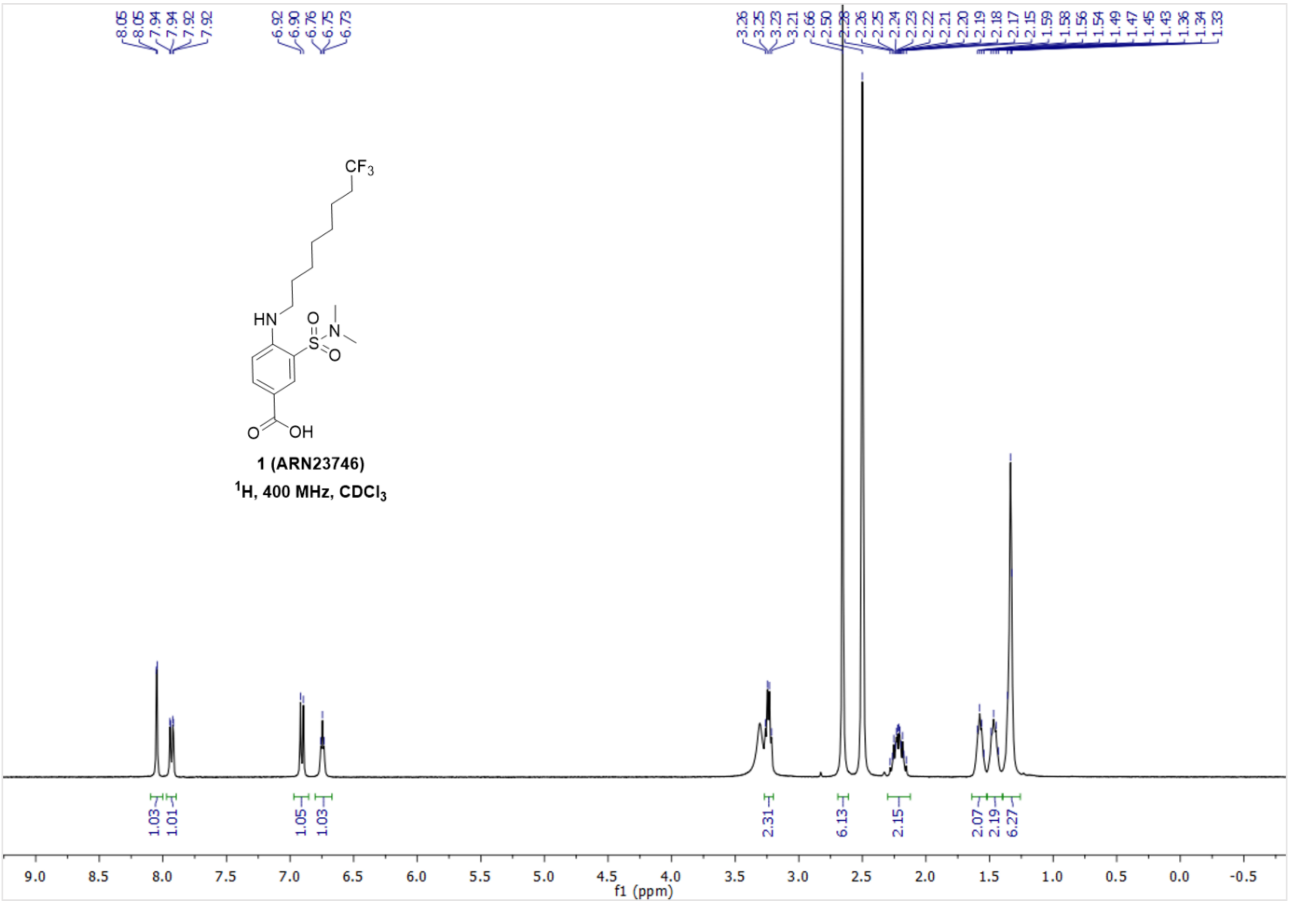

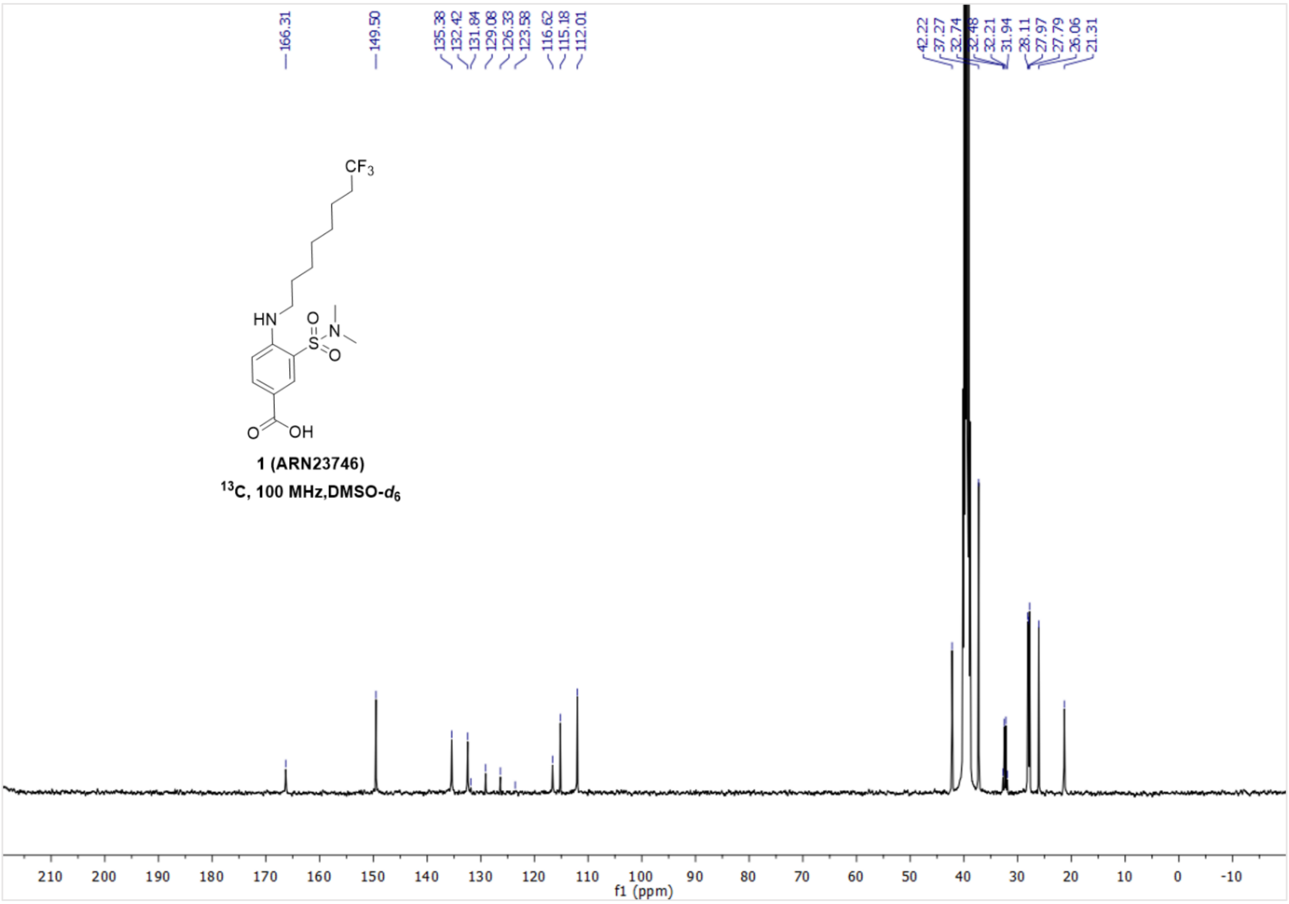

166.31
149.50
135.38
132.42
131.84
129.08
126.33
123.58
116.62
115.18
112.01
42.22
37.27
32.74
32.48
32.21
31.94
28.11
27.97
27.79
26.06
21.31
CF3
HN
O
S
N
O
O
OH
1 (ARN23746)
13C, 100 MHz,DMSO-d6
210 200 190 180 170 160 150 140 130 120 110 100 90 80 70 60 50 40 30 20 10 0 -10
f1 (ppm)

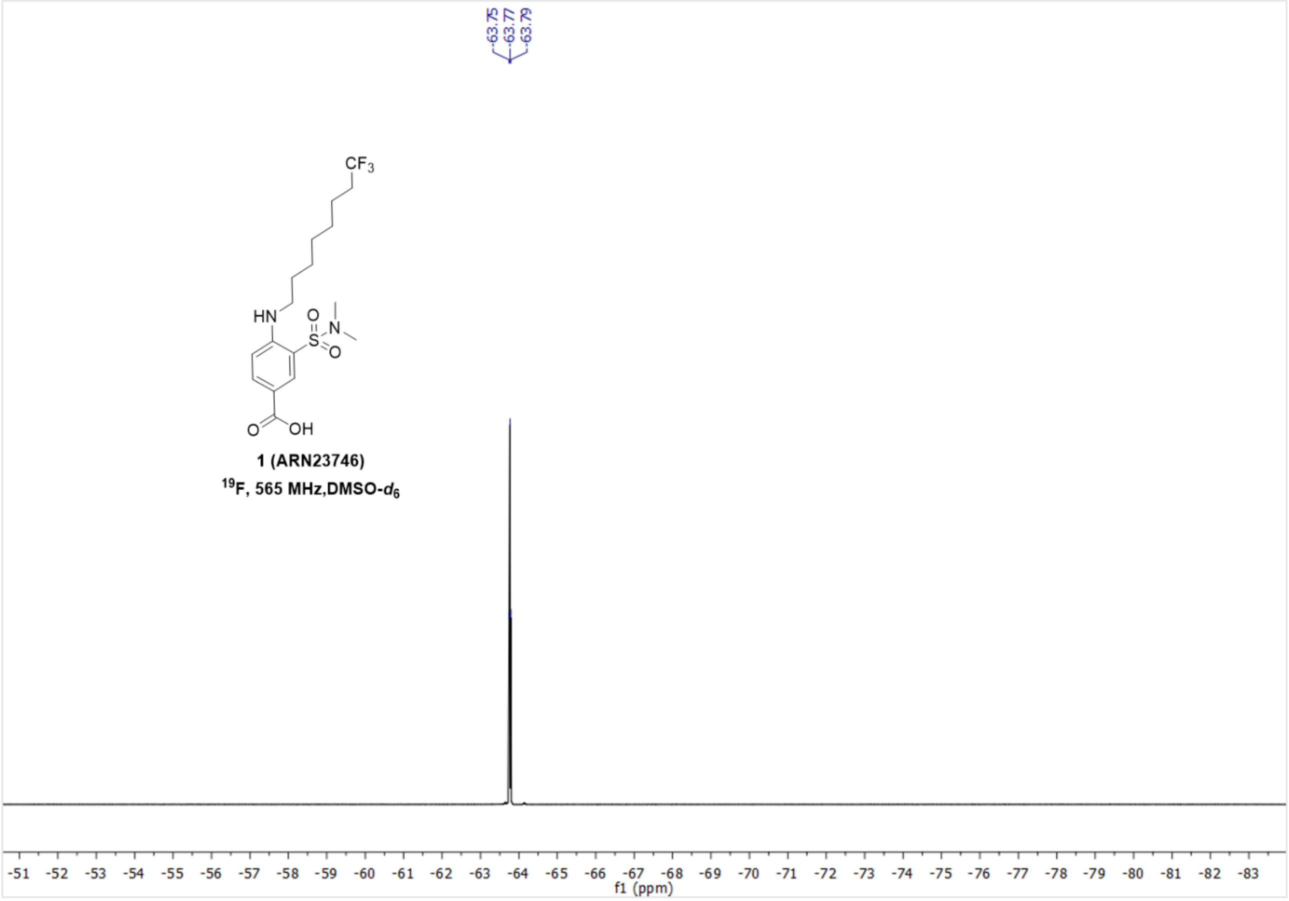

-63.75
-63.77
-63.79
CF3
HN
O
S
N
O
O
OH
1 (ARN23746)
19F, 565 MHz,DMSO-d6
-51 -52 -53 -54 -55 -56 -57 -58 -59 -60 -61 -62 -63 -64 -65 -66 -67 -68 -69 -70 -71 -72 -73 -74 -75 -76 -77 -78 -79 -80 -81 -82 -83
f1 (ppm)

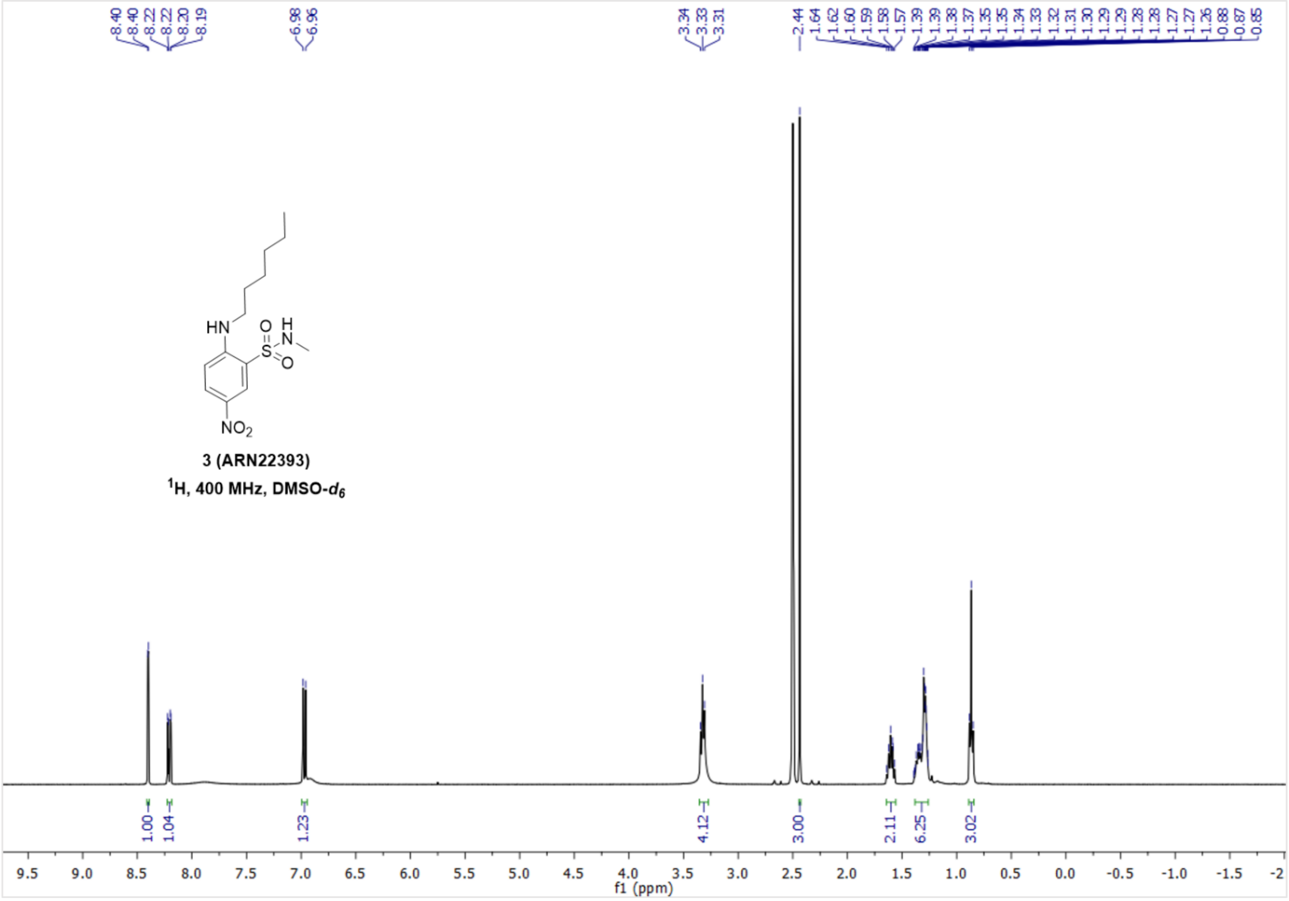
8.40
8.40
8.22
8.22
8.20
8.19
6.98
6.96
3.34
3.33
3.31
2.44
1.64
1.62
1.60
1.59
1.58
1.57
1.39
1.39
1.38
1.37
1.35
1.35
1.34
1.33
1.32
1.31
1.30
1.29
1.29
1.28
1.28
1.28
1.27
1.27
1.26
0.88
0.87
0.85
HN
O
H
S
N
O
NO_2
3 (ARN22393)
^{1}H, 400 MHz, DMSO-d_6
1.00
1.04
1.23
4.12
3.00
2.11
6.25
3.02
f1 (ppm)

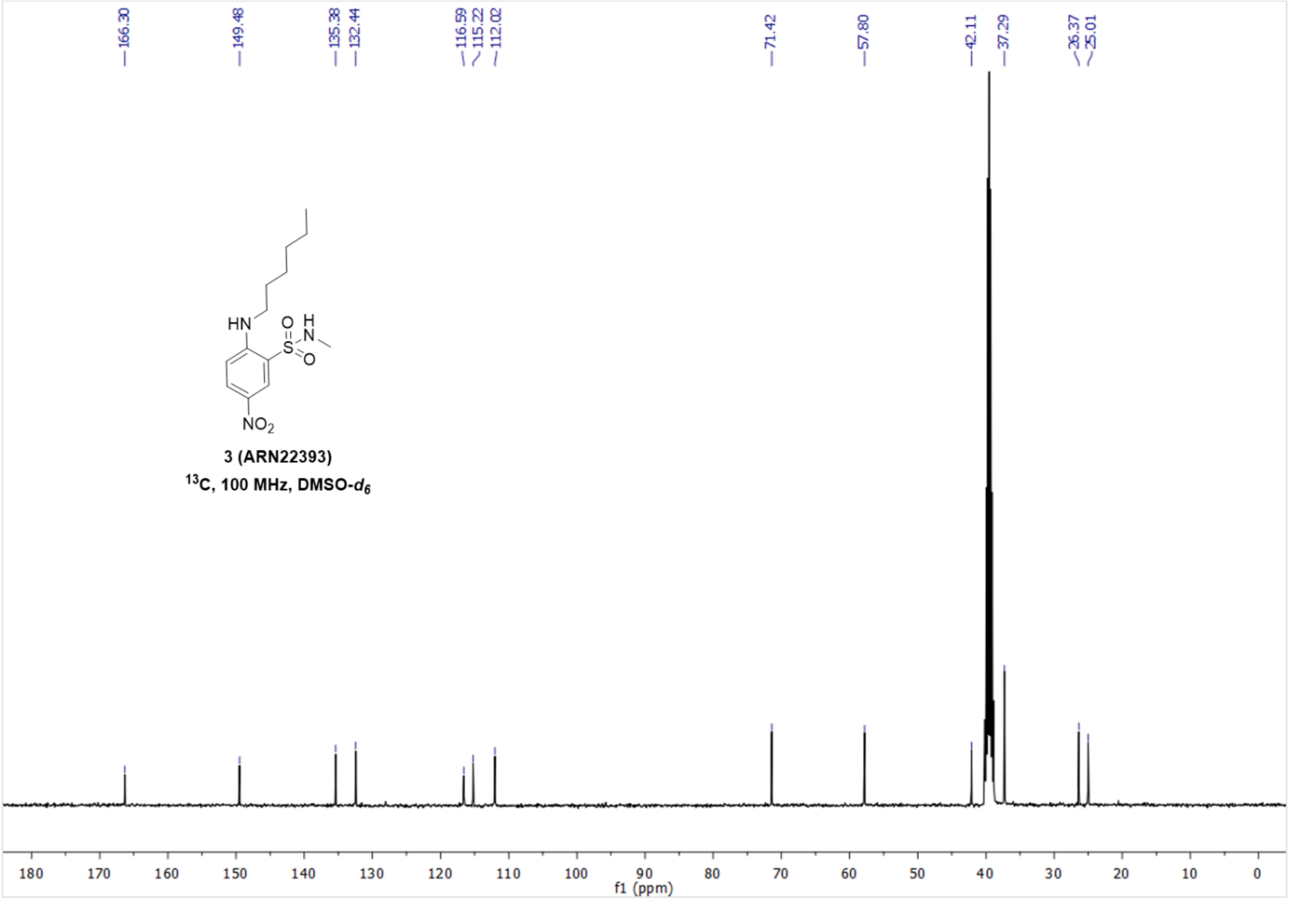
166.30
149.48
135.38
132.44
116.59
115.22
112.02
71.42
57.80
42.11
37.29
26.37
25.01
HN
O
H
S
N
O
NO_2
3 (ARN22393)
^{13}C, 100 MHz, DMSO-d_6
f1 (ppm)

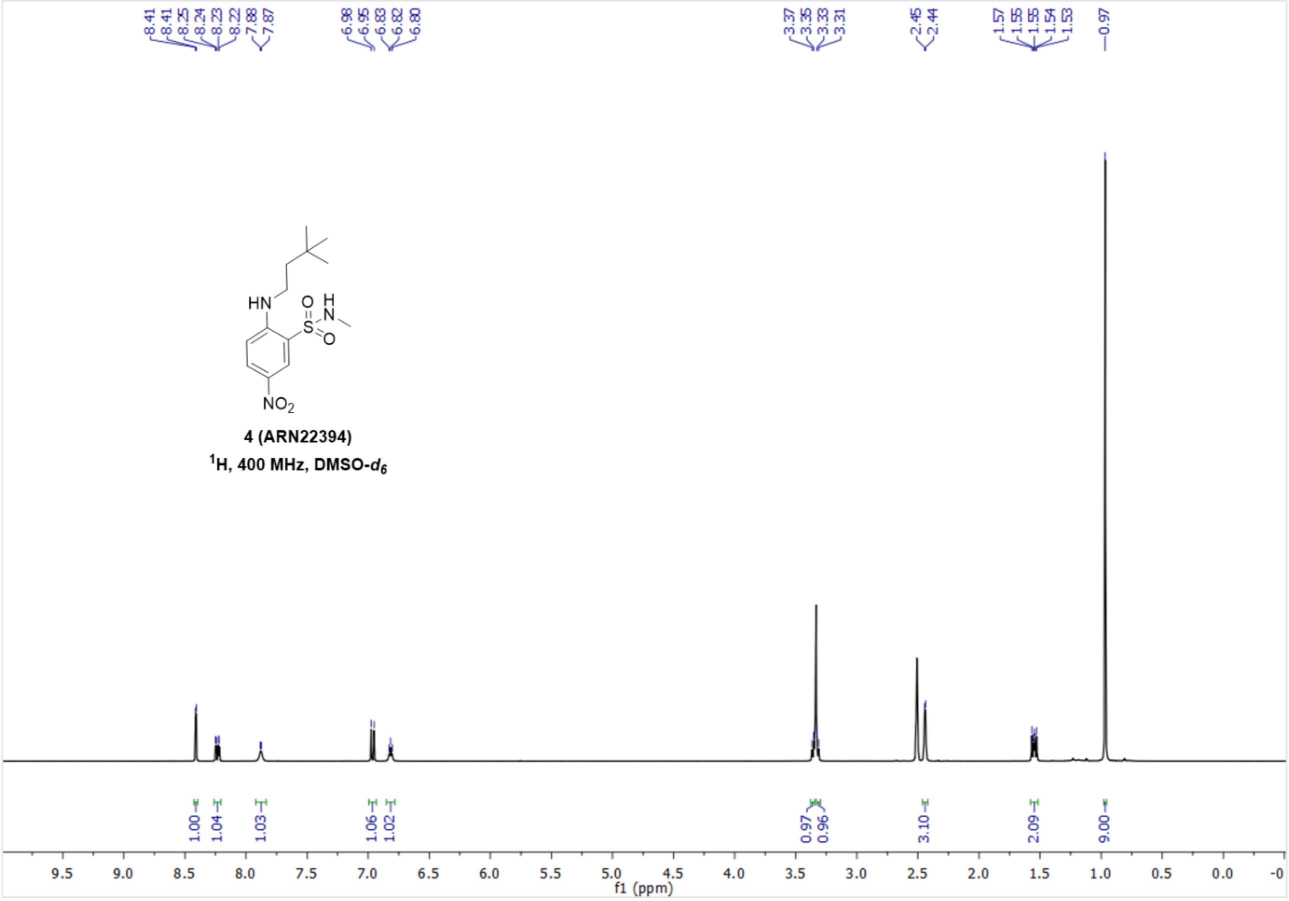

8.41
8.41
8.25
8.24
8.23
8.22
7.88
7.87
6.98
6.95
6.83
6.82
6.80
3.37
3.35
3.33
3.31
2.45
2.44
1.57
1.55
1.55
1.54
1.53
0.97
HN
O
H
N
S
O
NO2
4 (ARN22394)
1H, 400 MHz, DMSO-d6
1.00
1.04
1.03
1.06
1.02
0.97
0.96
3.10
2.09
9.00
f1 (ppm)

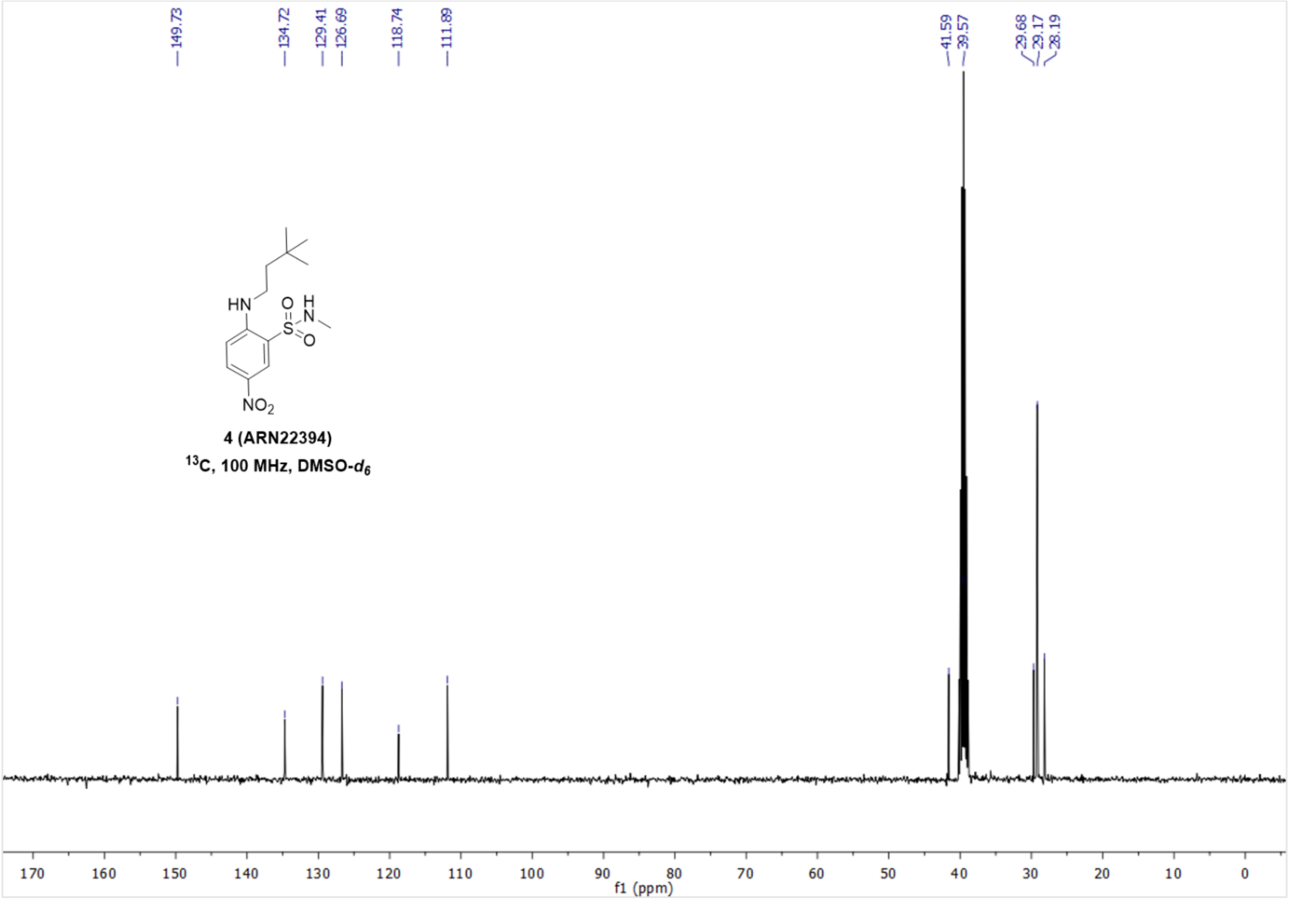

149.73
134.72
129.41
126.69
118.74
111.89
41.59
39.57
29.68
29.17
28.19
HN
O
H
N
S
O
NO2
4 (ARN22394)
13C, 100 MHz, DMSO-d6
f1 (ppm)

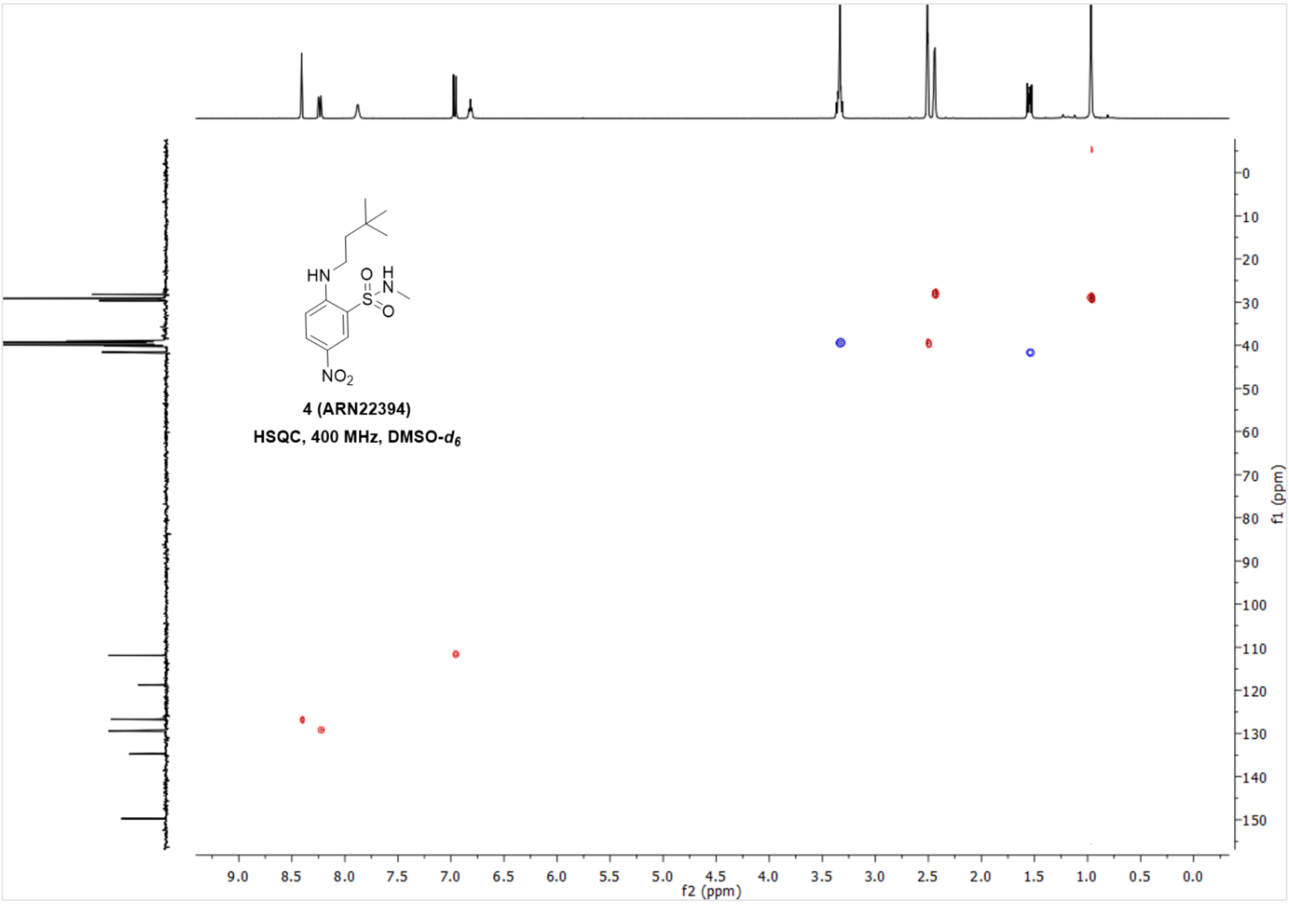

4 (ARN22394)
HSQC, 400 MHz, DMSO-d_6
f2 (ppm)
f1 (ppm)

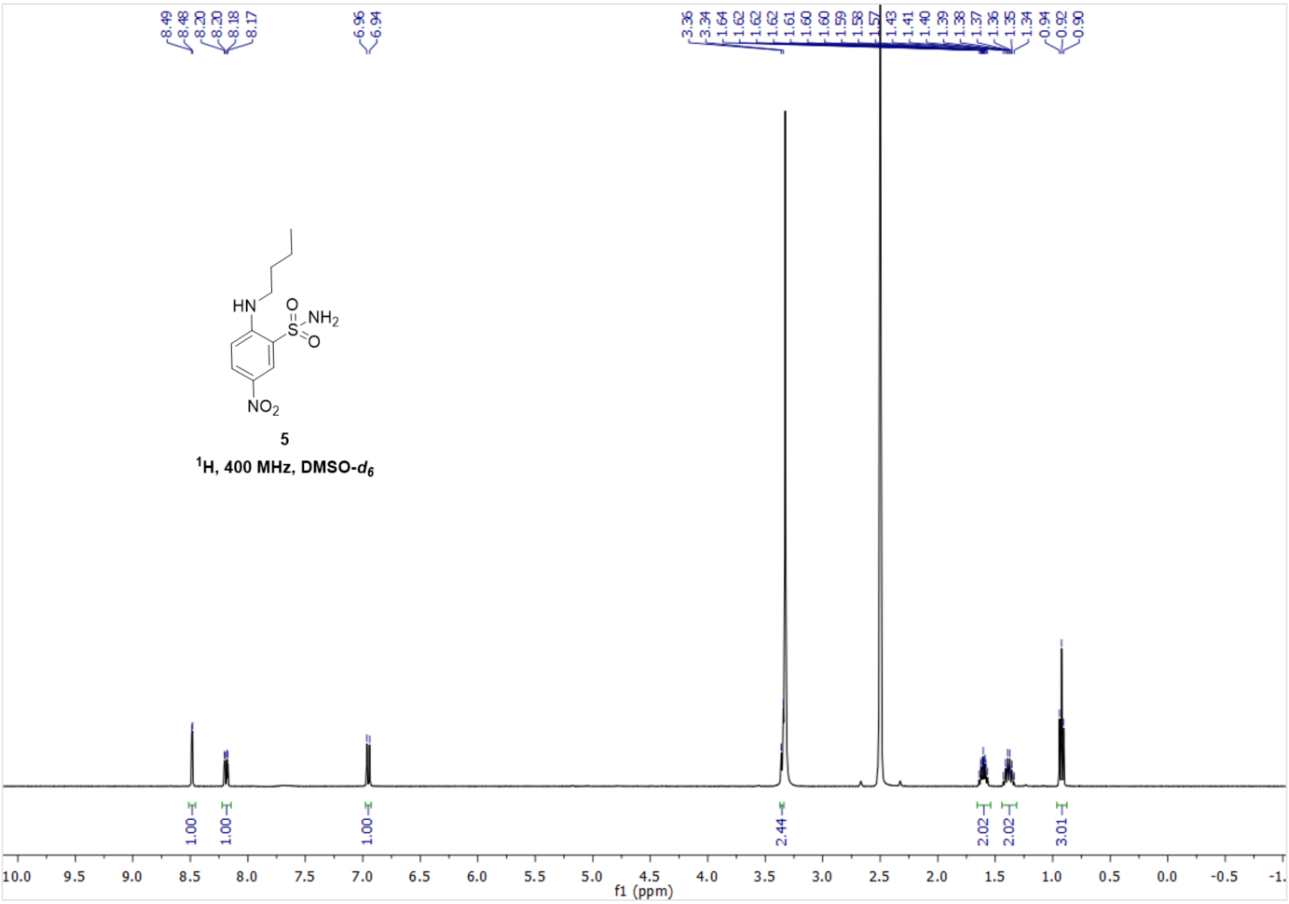

5
^{1}H, 400 MHz, DMSO-d_6
f1 (ppm)

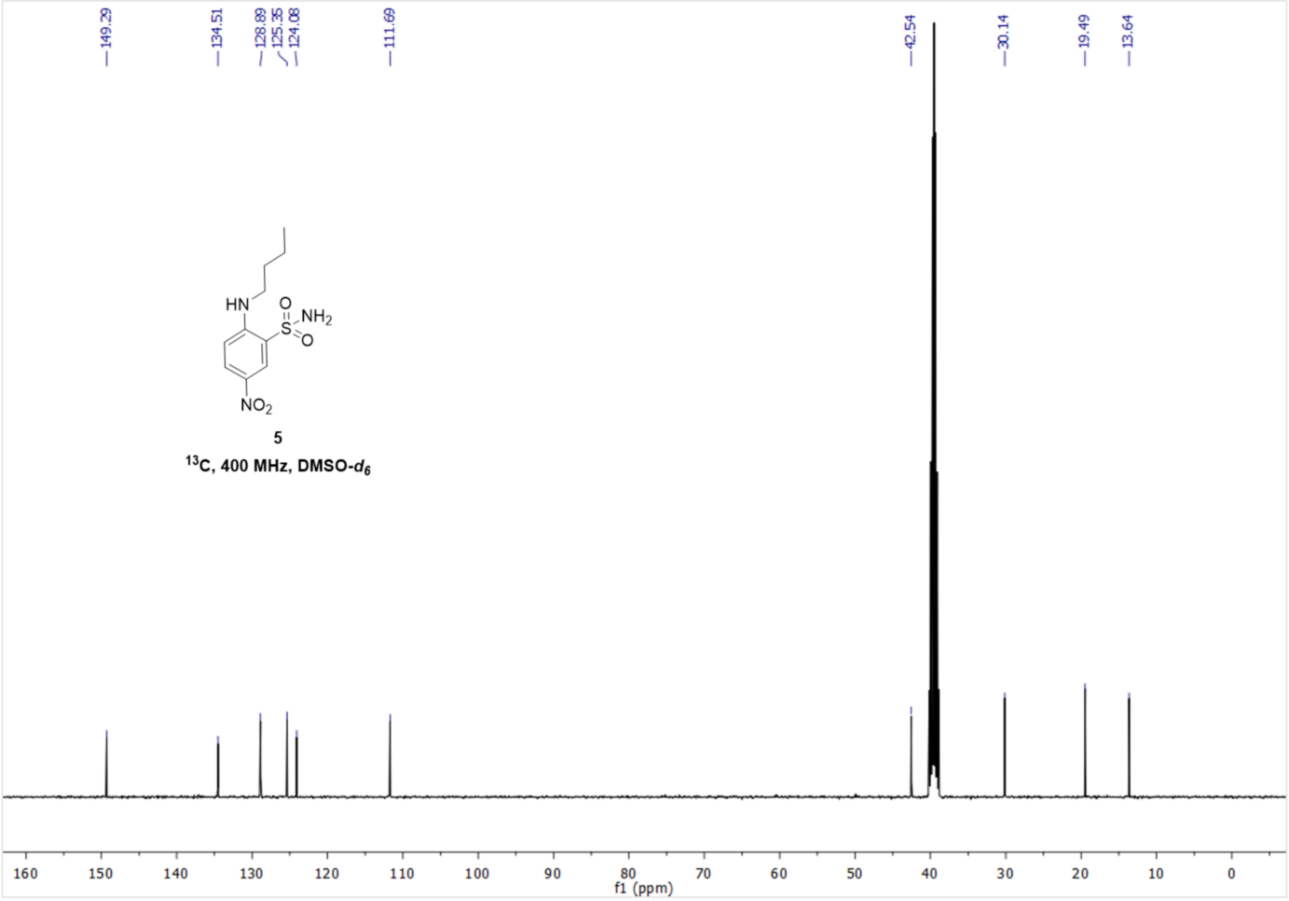

149.29
134.51
128.89
125.35
124.08
111.69
42.54
30.14
19.49
13.64
HN
NH2
NO2
5
13C, 400 MHz, DMSO-d6
f1 (ppm)

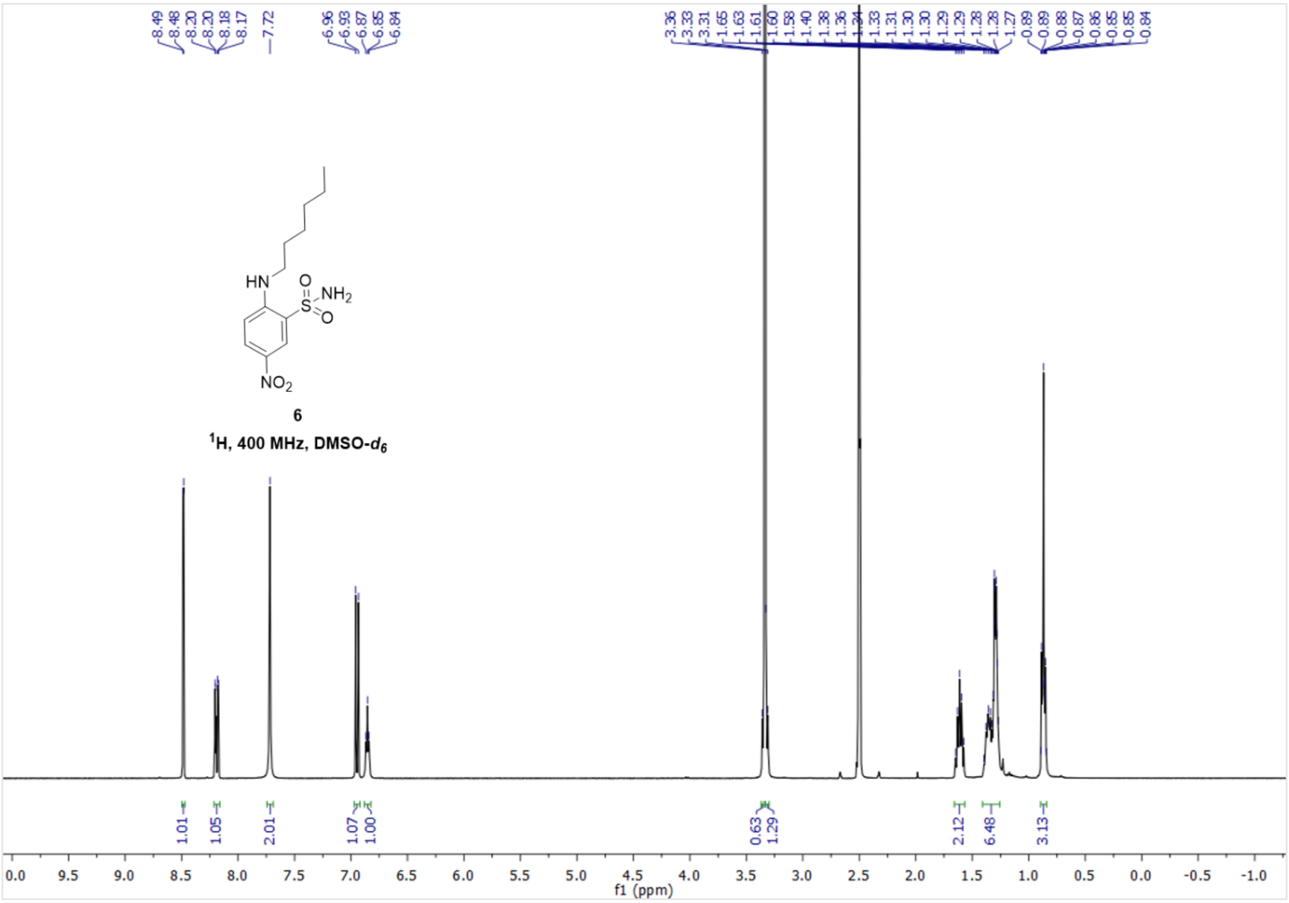

HN
NH2
NO2
6
1H, 400 MHz, DMSO-d6
1.01
1.05
2.01
1.07
1.00
0.63
1.29
2.12
6.48
3.13
f1 (ppm)

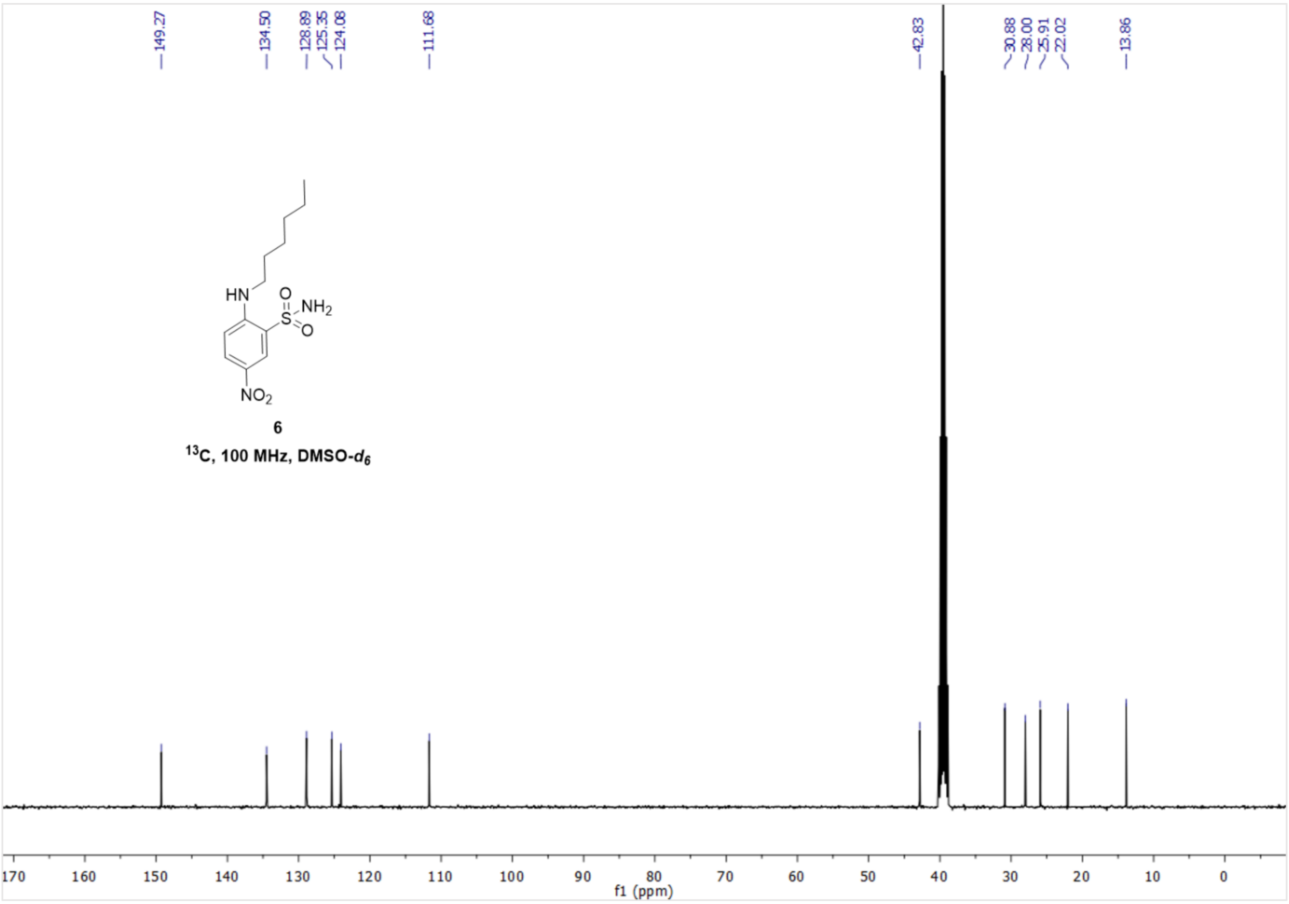
149.27
134.50
128.89
125.35
124.08
111.68
42.83
30.88
28.00
25.91
22.02
13.86
HN
NH2
NO2
6
13C, 100 MHz, DMSO-d6
f1 (ppm)

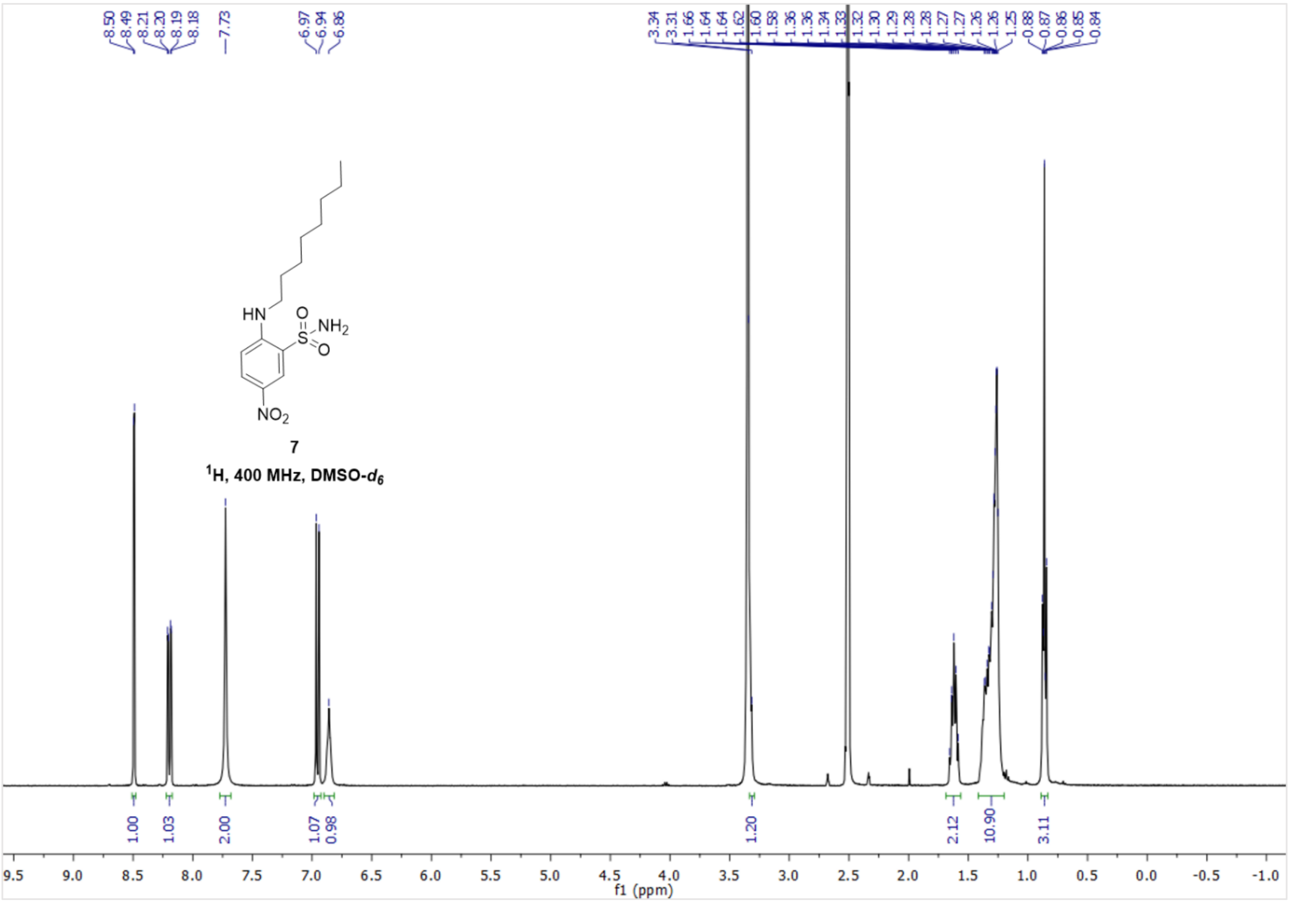
8.50
8.49
8.21
8.20
8.19
8.18
7.73
6.97
6.94
6.86
3.34
3.31
1.66
1.64
1.64
1.62
1.60
1.58
1.36
1.36
1.34
1.33
1.32
1.30
1.29
1.28
1.28
1.27
1.27
1.26
1.26
1.25
0.88
0.87
0.86
0.85
0.84
HN
NH2
NO2
7
1H, 400 MHz, DMSO-d6
1.00
1.03
2.00
1.07
0.98
1.20
2.12
10.90
3.11
f1 (ppm)

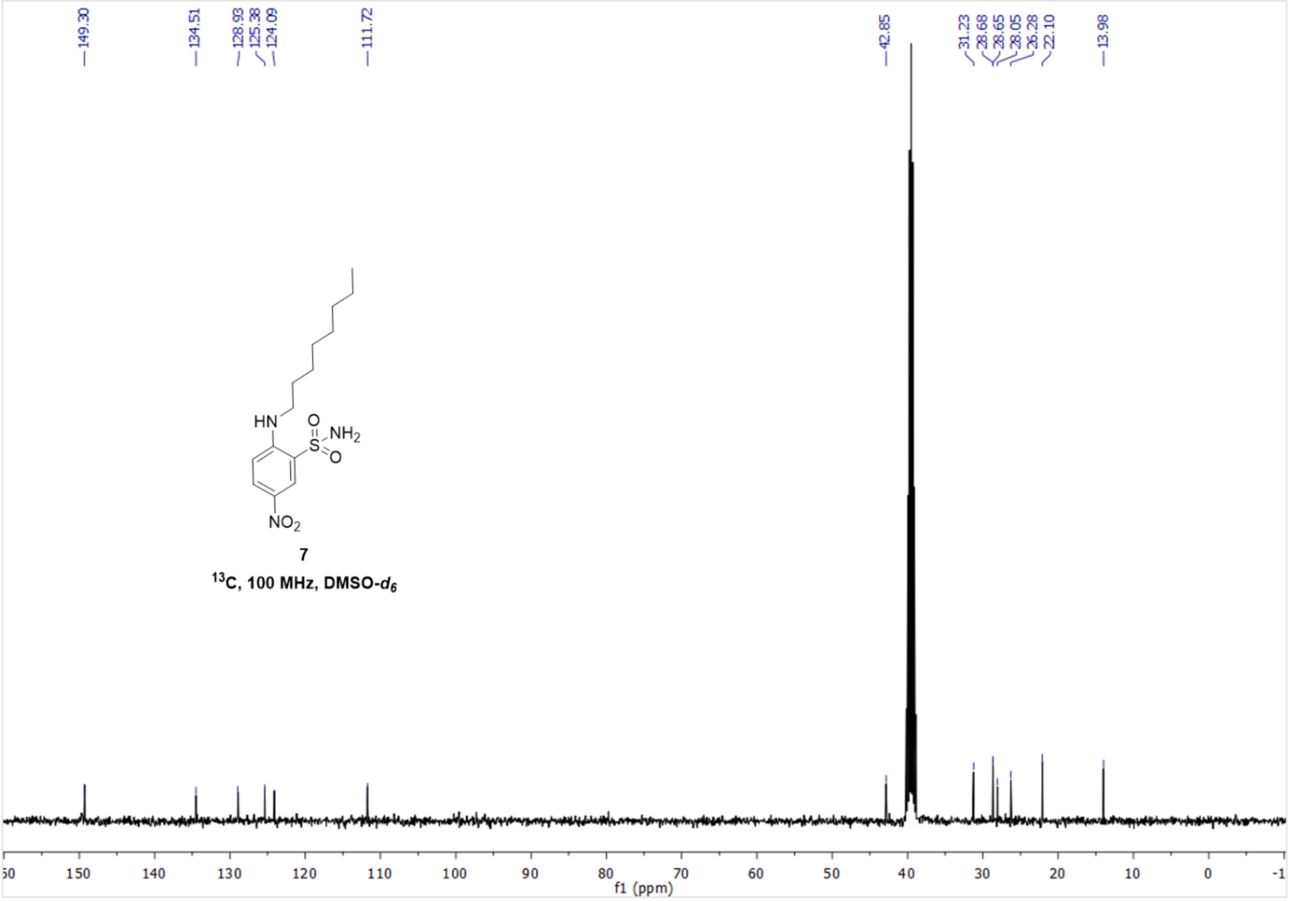
149.30
134.51
128.93
125.38
124.09
111.72
42.85
31.23
28.68
28.65
28.05
26.28
22.10
13.98
HN
NH2
NO2
7
13C, 100 MHz, DMSO-d6
f1 (ppm)

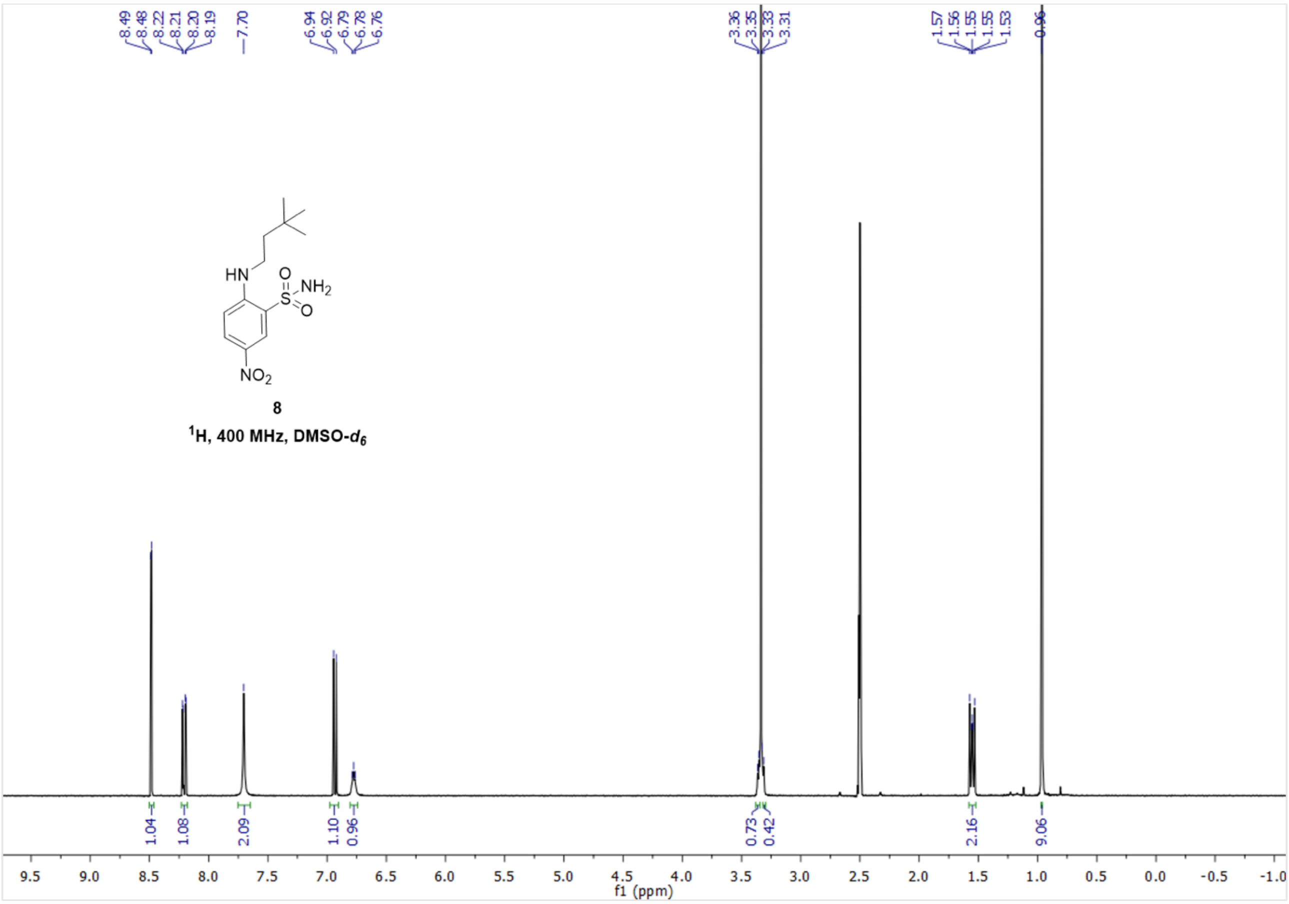
8.49
8.48
8.22
8.21
8.20
8.19
7.70
6.94
6.92
6.79
6.78
6.76
3.36
3.35
3.33
3.31
1.57
1.56
1.55
1.55
1.53
0.96
HN
NH2
NO2
8
1H, 400 MHz, DMSO-d6
1.04
1.08
2.09
1.10
0.96
0.73
0.42
2.16
9.06
f1 (ppm)

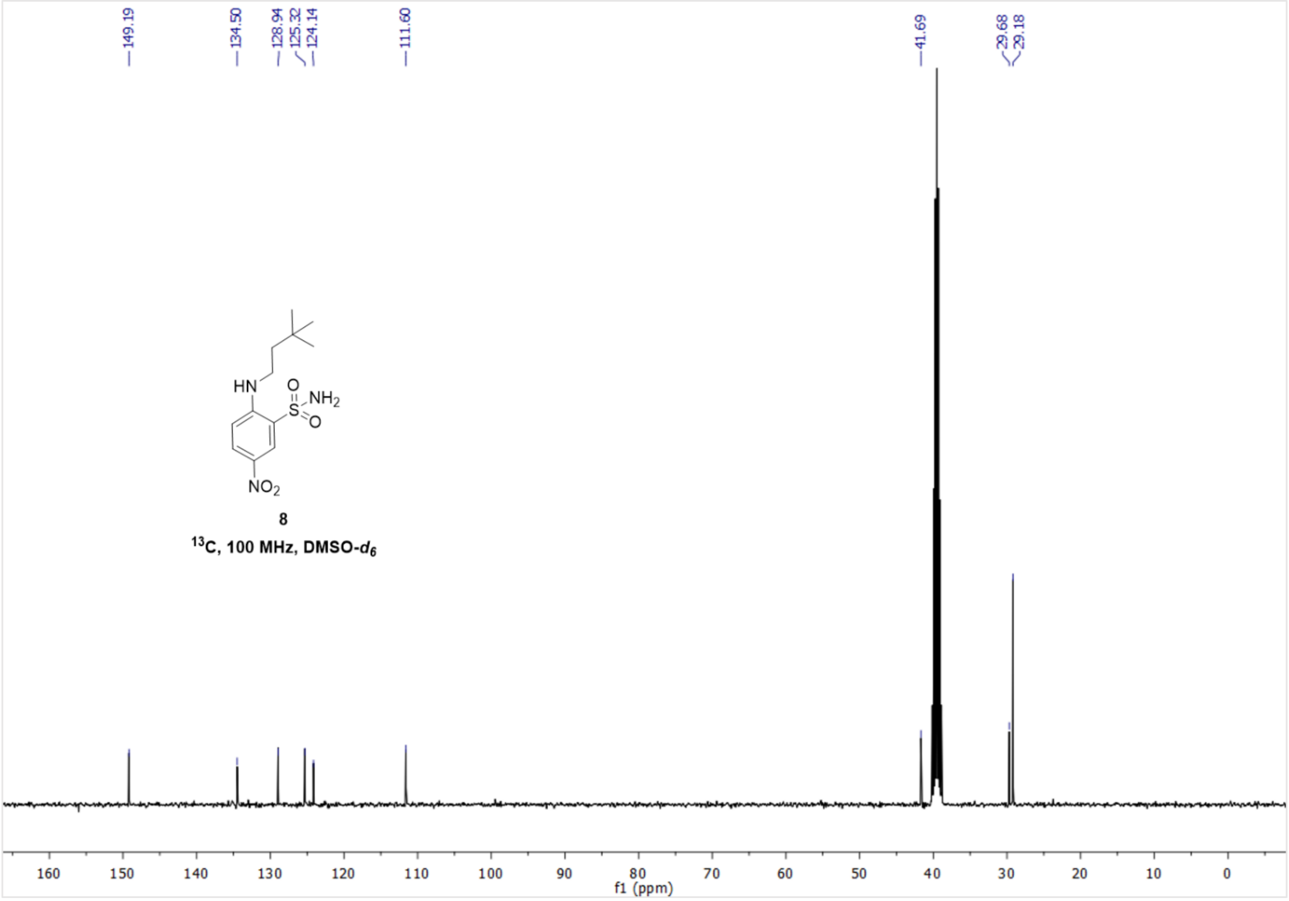
149.19
134.50
128.94
125.32
124.14
111.60
41.69
29.68
29.18
13C, 100 MHz, DMSO-d6
f1 (ppm)

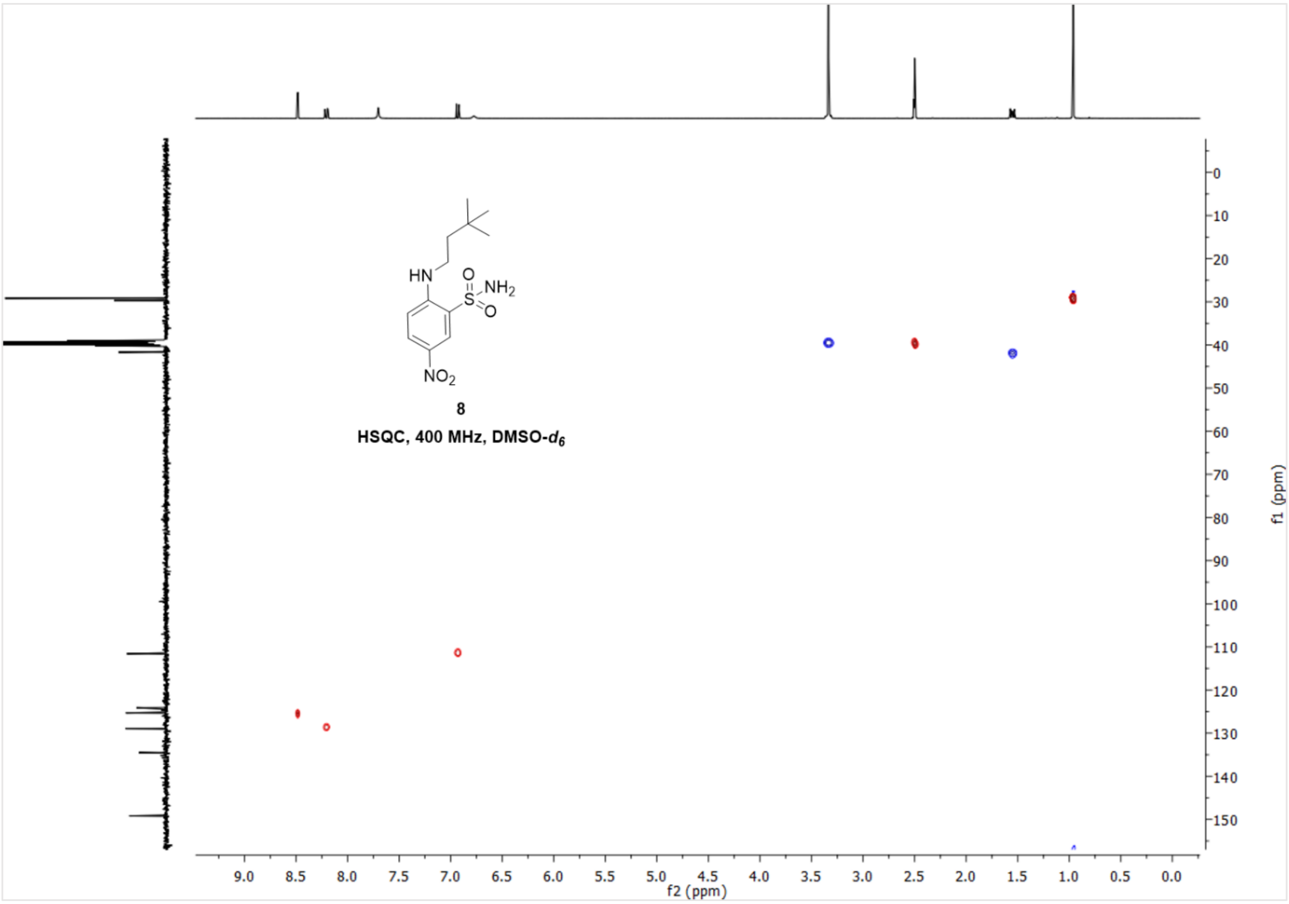
HSQC, 400 MHz, DMSO-d6
f2 (ppm)
f1 (ppm)

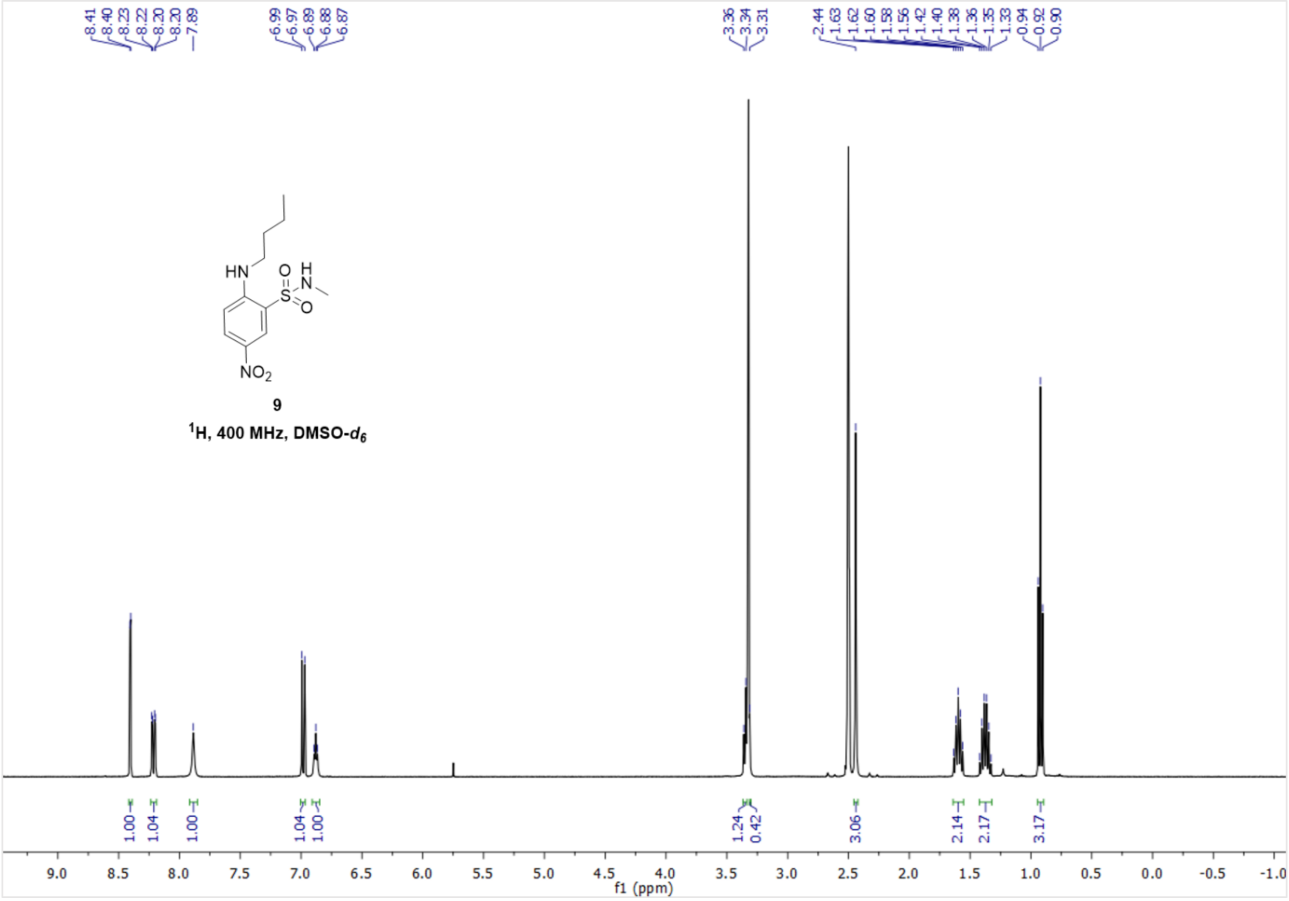
9
¹H, 400 MHz, DMSO-d6
f1 (ppm)

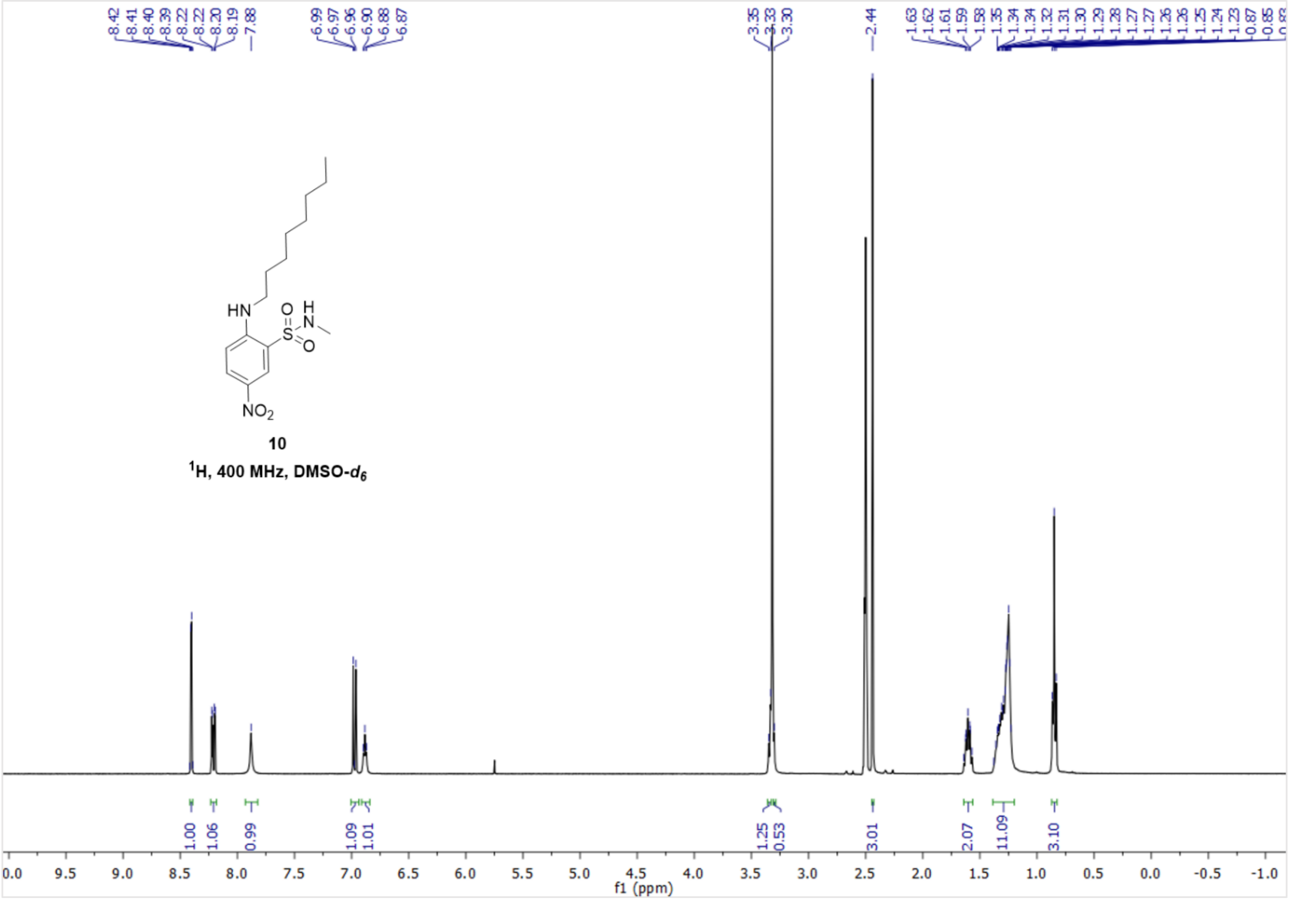
10
¹H, 400 MHz, DMSO-d6
f1 (ppm)

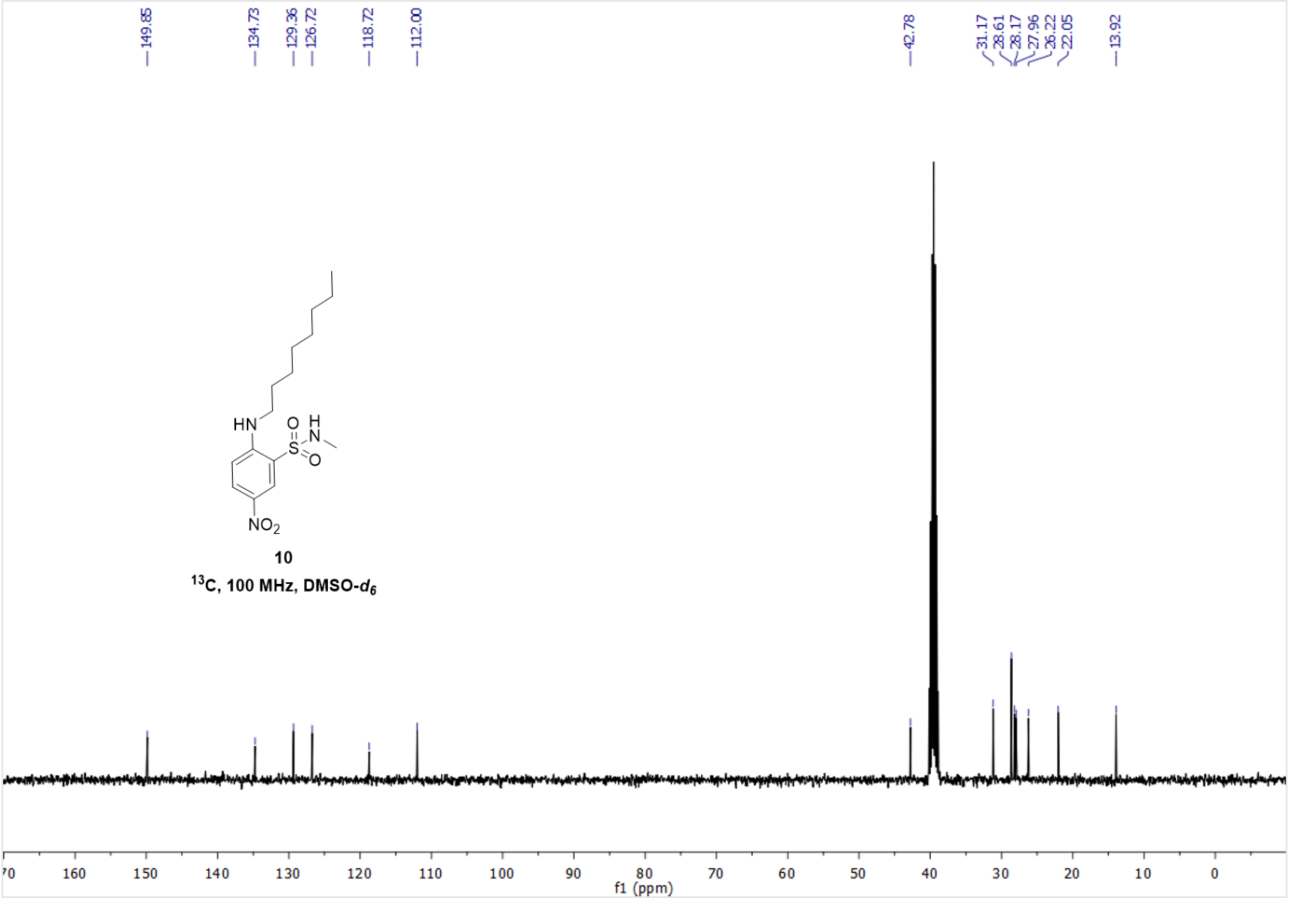

10

^{13}C, 100 MHz, DMSO-d_6

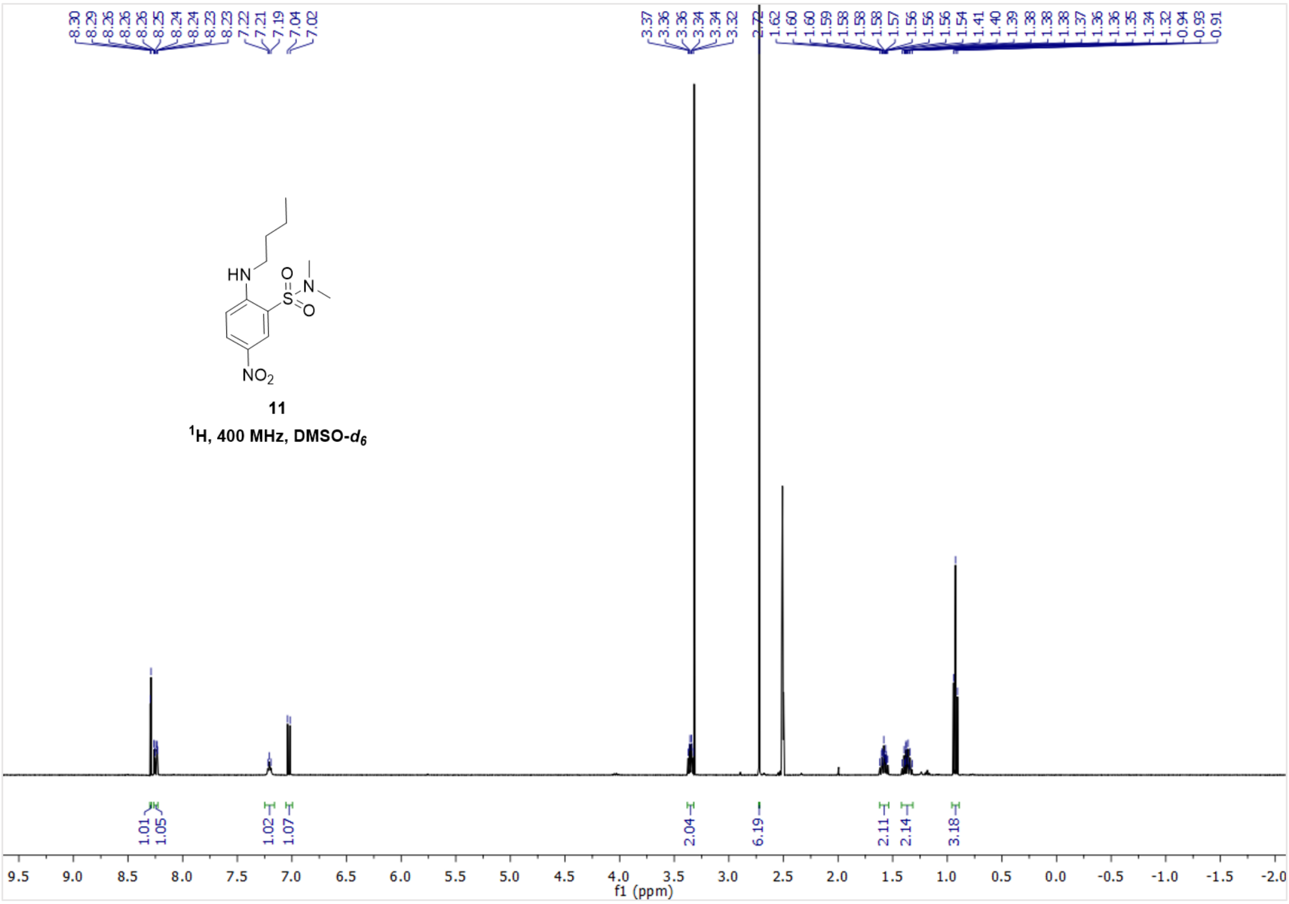

11

^{1}H, 400 MHz, DMSO-d_6

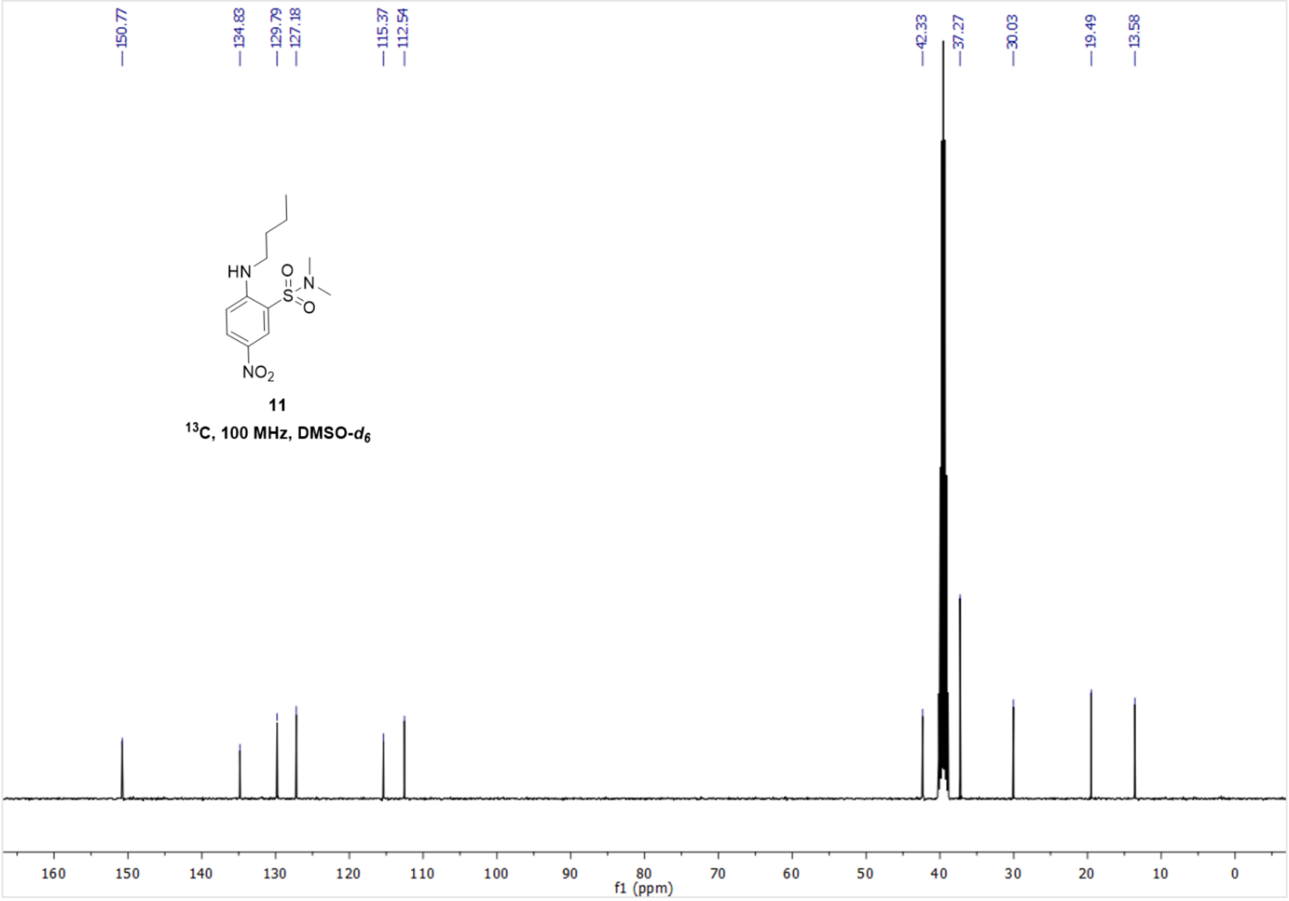

150.77
134.83
129.79
127.18
115.37
112.54
42.33
37.27
30.03
19.49
13.58
HN
O
S
N
O
NO2
11
13C, 100 MHz, DMSO-d6
f1 (ppm)

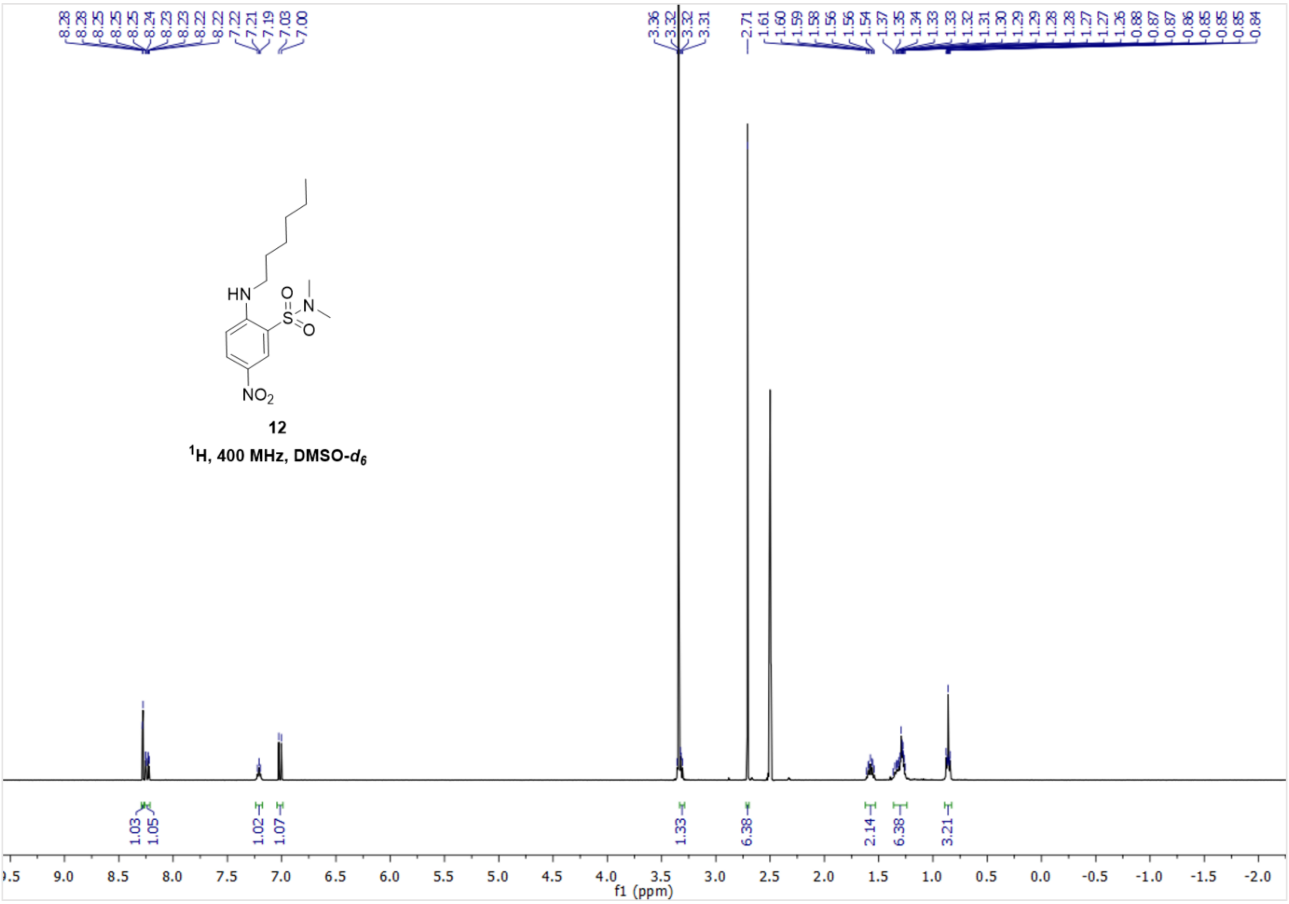

HN
O
S
N
O
NO2
12
1H, 400 MHz, DMSO-d6
1.03
1.05
1.02
1.07
1.33
6.38
2.14
6.38
3.21
f1 (ppm)

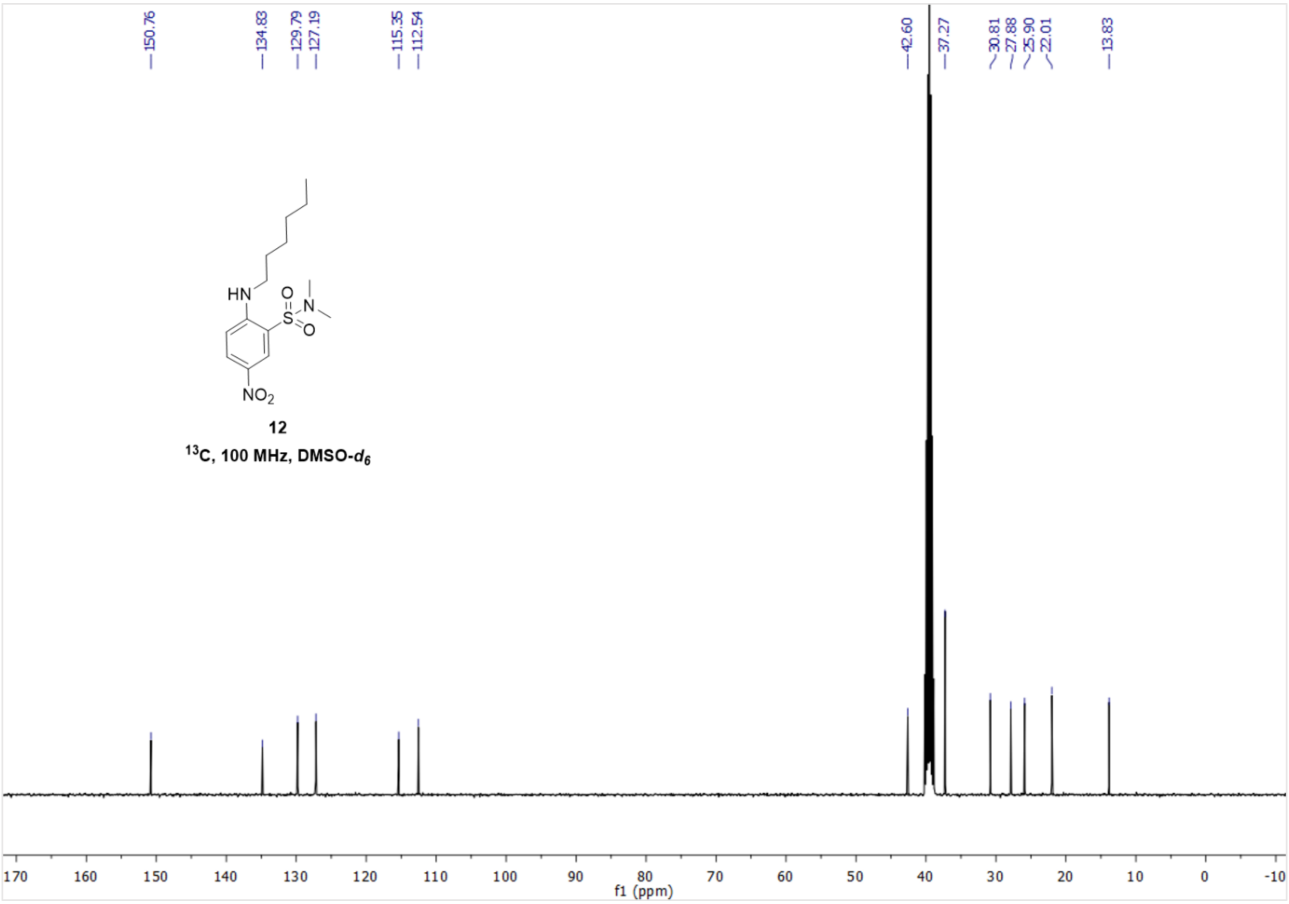
150.76
134.83
129.79
127.19
115.35
112.54
42.60
37.27
30.81
27.88
25.90
22.01
13.83
HN
O
S
N
O
NO_2
12
^{13}C, 100 MHz, DMSO-d_6
f1 (ppm)

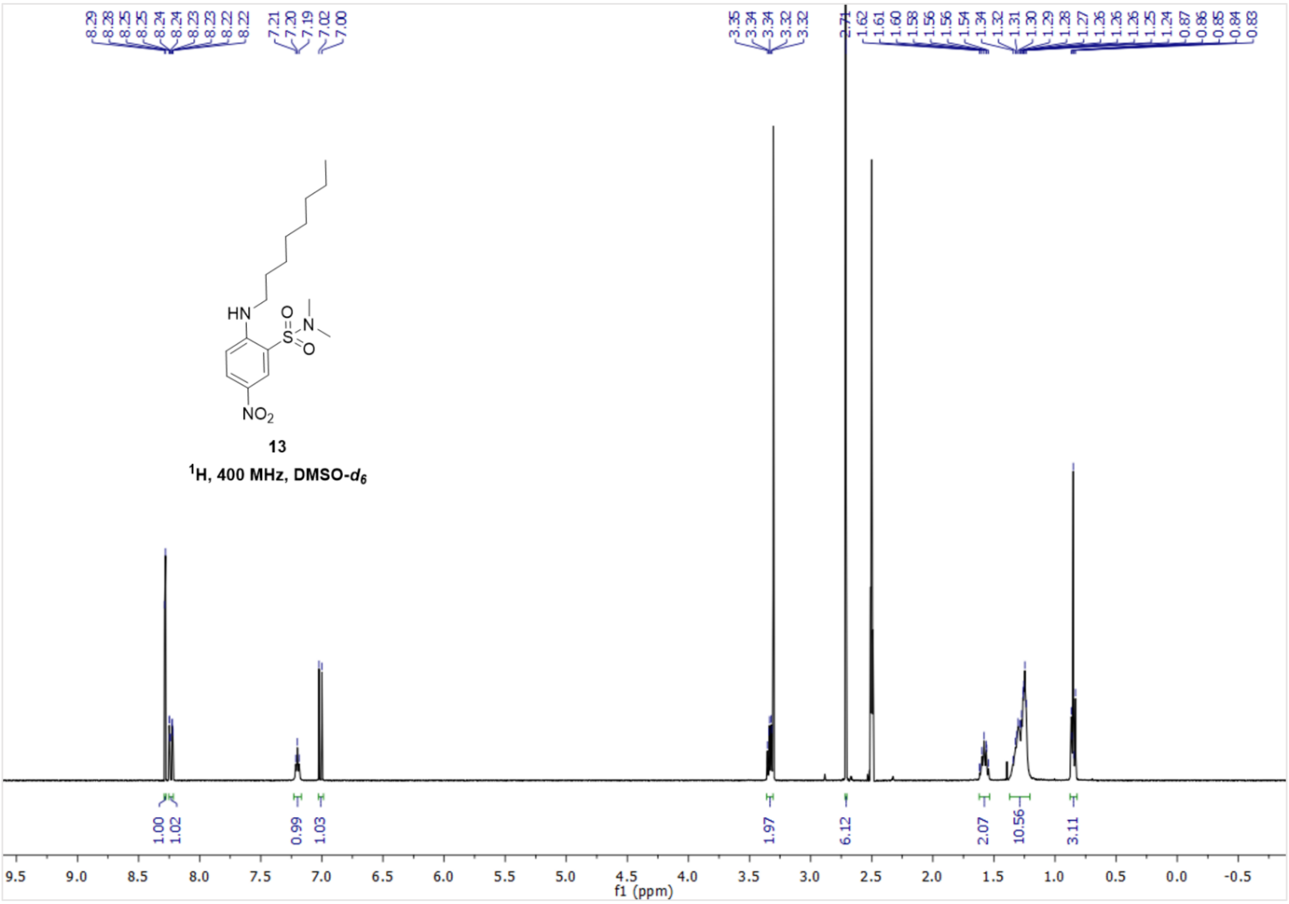
8.29
8.28
8.25
8.25
8.24
8.24
8.23
8.23
8.22
8.22
7.21
7.20
7.19
7.02
7.00
3.35
3.34
3.34
3.32
3.32
2.71
1.62
1.61
1.60
1.58
1.56
1.56
1.54
1.34
1.32
1.31
1.30
1.29
1.28
1.27
1.26
1.26
1.26
1.25
1.24
0.87
0.86
0.85
0.84
0.83
HN
O
S
N
O
NO_2
13
^{1}H, 400 MHz, DMSO-d_6
1.00
1.02
0.99
1.03
1.97
6.12
2.07
10.56
3.11
f1 (ppm)

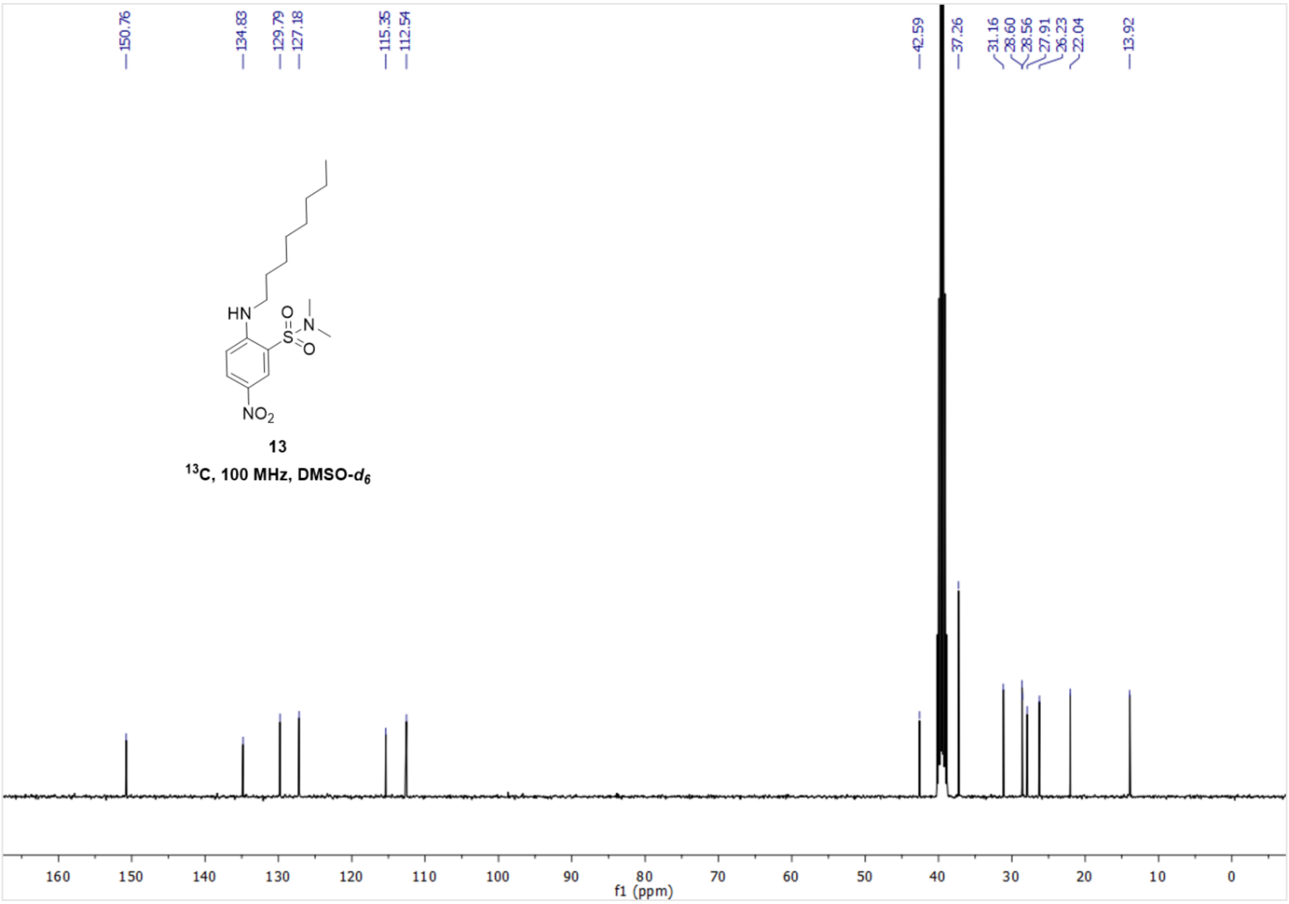

13

^{13}C, 100 MHz, DMSO-d_6

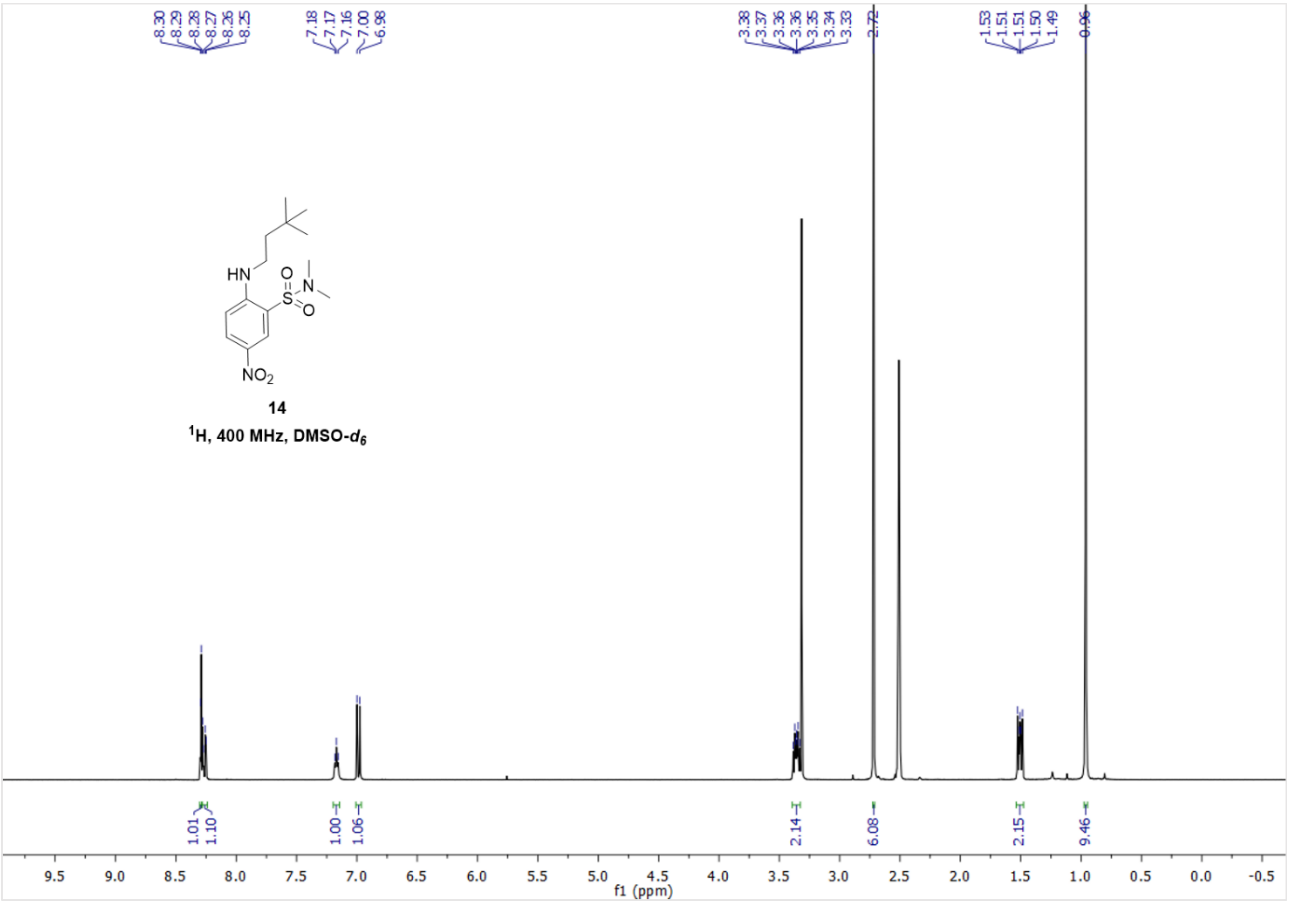

14

^{1}H, 400 MHz, DMSO-d_6

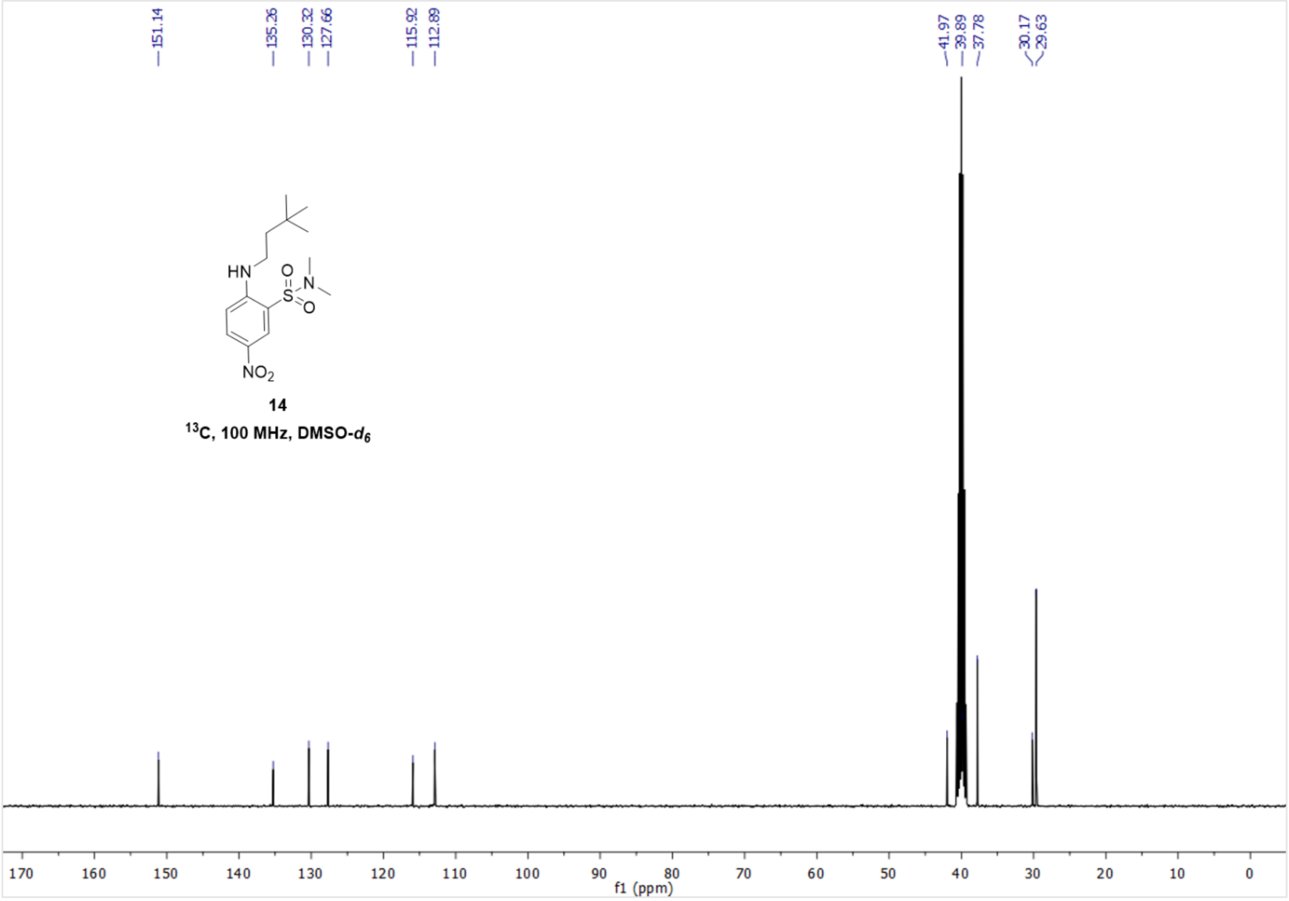

151.14
135.36
130.32
127.66
115.92
112.89
41.97
39.89
37.78
30.17
29.63
HN
O
S
N
O
NO2
14
13C, 100 MHz, DMSO-d6
170 160 150 140 130 120 110 100 90 80 70 60 50 40 30 20 10 0
f1 (ppm)

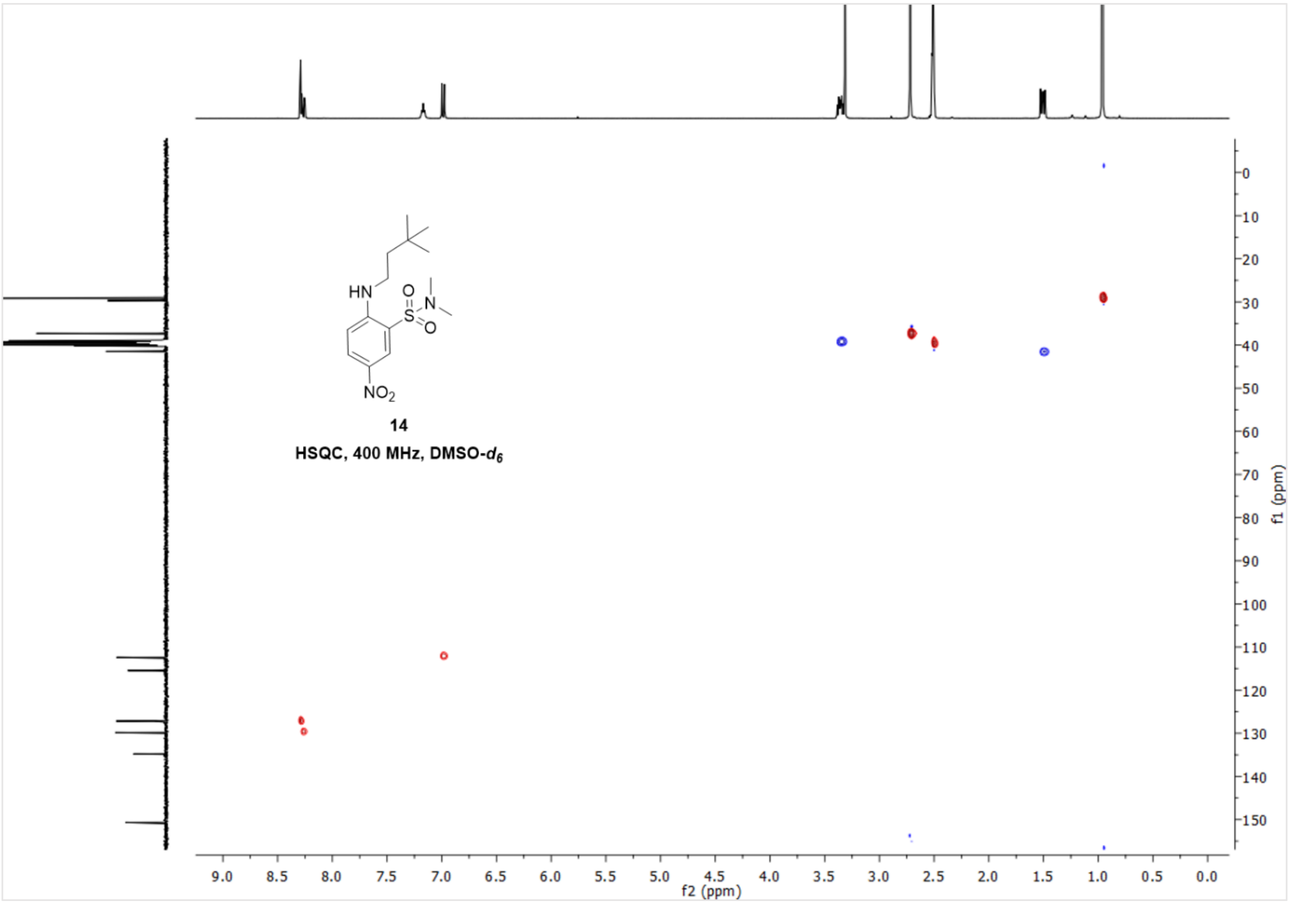

HN
O
S
N
O
NO2
14
HSQC, 400 MHz, DMSO-d6
0 10 20 30 40 50 60 70 80 90 100 110 120 130 140 150
f1 (ppm)
9.0 8.5 8.0 7.5 7.0 6.5 6.0 5.5 5.0 4.5 4.0 3.5 3.0 2.5 2.0 1.5 1.0 0.5 0.0
f2 (ppm)

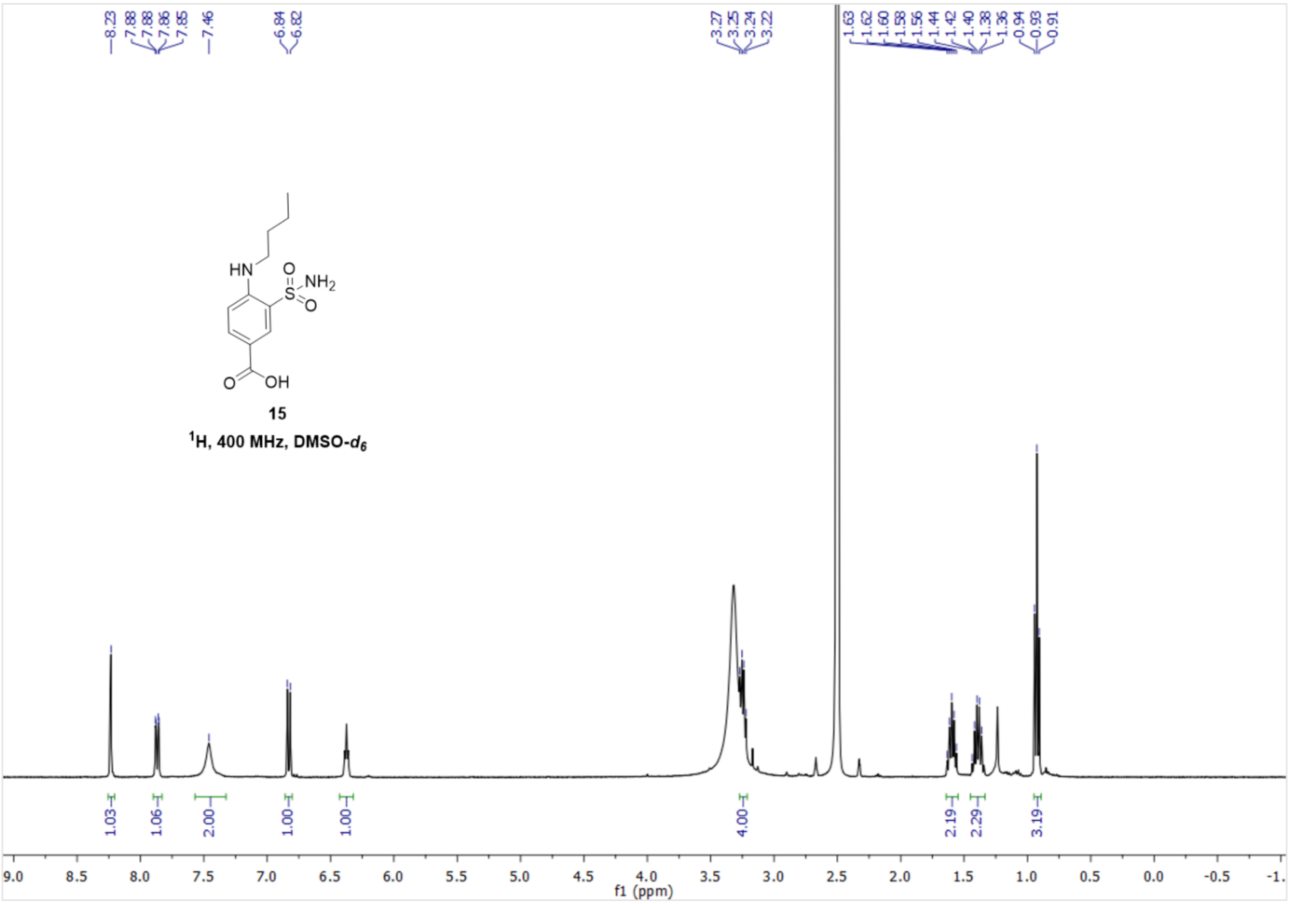

8.23
7.88
7.88
7.86
7.85
7.46
6.84
6.82
3.27
3.25
3.24
3.22
1.63
1.62
1.60
1.58
1.56
1.44
1.42
1.40
1.38
1.36
0.94
0.93
0.91
HN
O
S
NH2
O
O
OH
15
1H, 400 MHz, DMSO-d6
1.03
1.06
2.00
1.00
1.00
4.00
2.19
2.29
3.19
f1 (ppm)

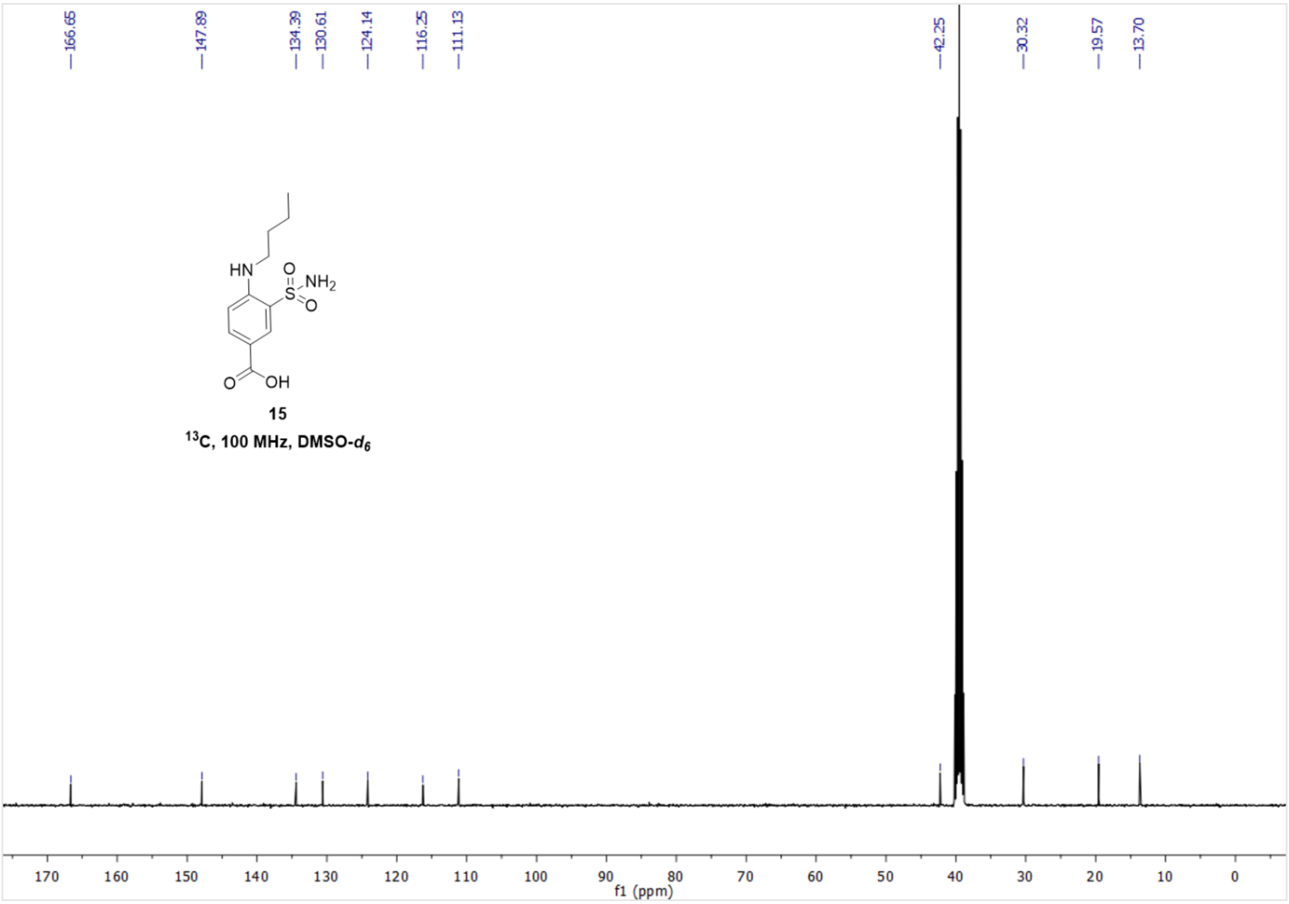

166.65
147.89
134.39
130.61
124.14
116.25
111.13
42.25
30.32
19.57
13.70
HN
O
S
NH2
O
O
OH
15
13C, 100 MHz, DMSO-d6
f1 (ppm)

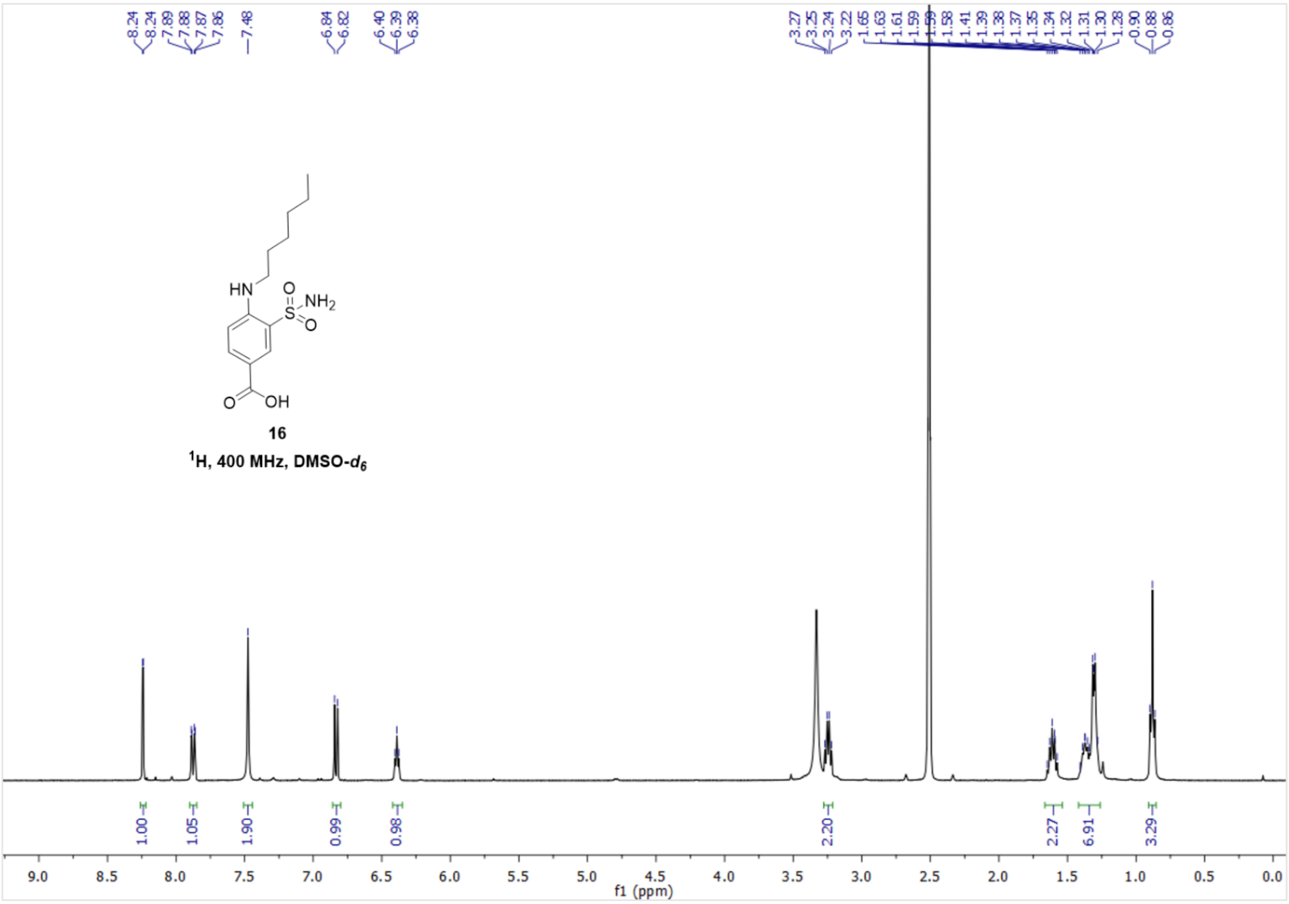
8.24
8.24
7.89
7.88
7.87
7.86
7.48
6.84
6.82
6.40
6.39
6.38
3.27
3.25
3.24
3.22
1.65
1.63
1.61
1.59
1.59
1.58
1.41
1.39
1.38
1.37
1.35
1.34
1.32
1.31
1.30
1.28
0.90
0.88
0.86
HN
O
S
NH2
O
O
OH
16
1H, 400 MHz, DMSO-d6
1.00
1.05
1.90
0.99
0.98
2.20
2.27
6.91
3.29
9.0
8.5
8.0
7.5
7.0
6.5
6.0
5.5
5.0
4.5
4.0
3.5
3.0
2.5
2.0
1.5
1.0
0.5
0.0
f1 (ppm)

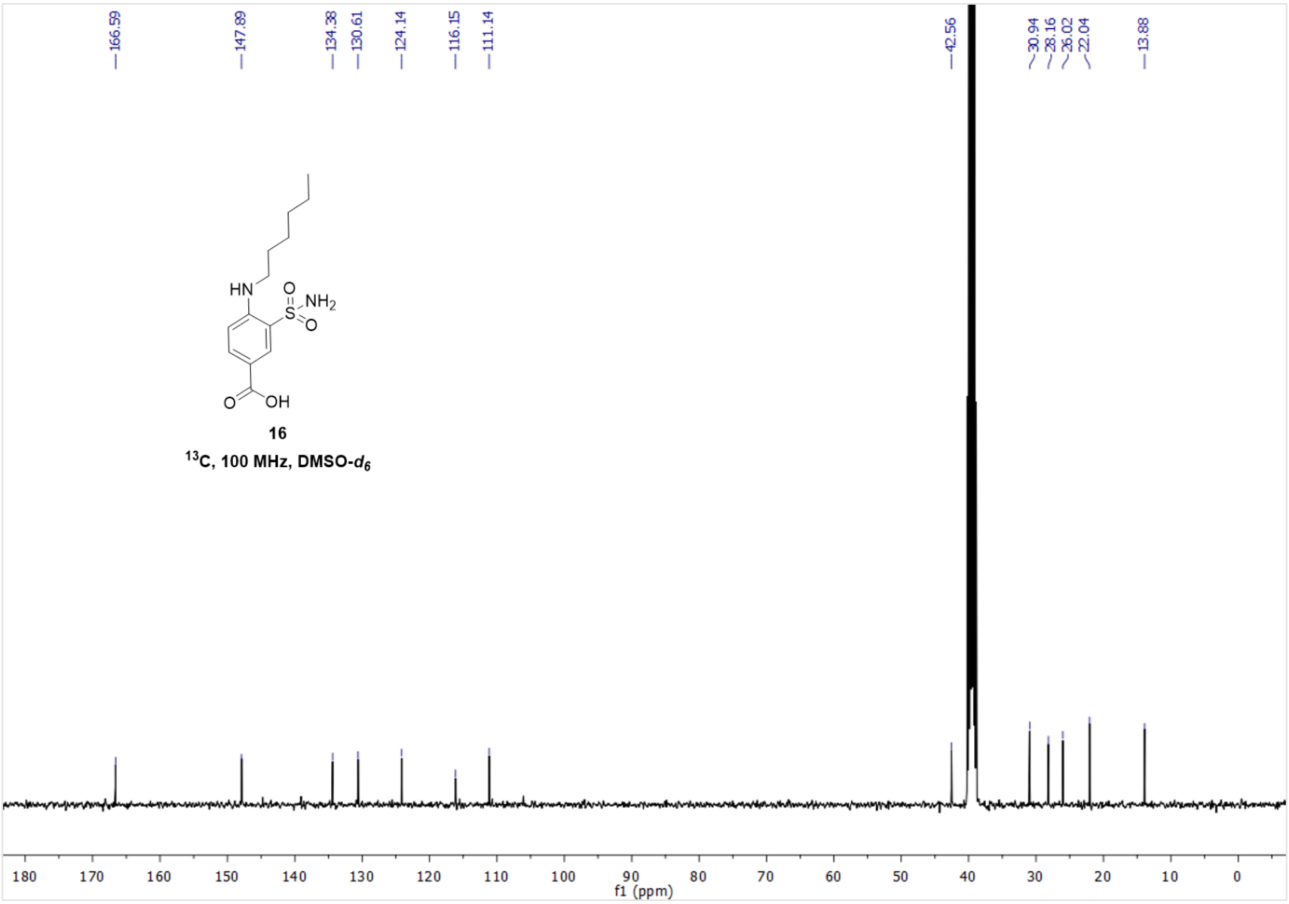
166.59
147.89
134.38
130.61
124.14
116.15
111.14
42.56
30.94
28.16
26.02
22.04
13.88
HN
O
S
NH2
O
O
OH
16
13C, 100 MHz, DMSO-d6
180
170
160
150
140
130
120
110
100
90
80
70
60
50
40
30
20
10
0
f1 (ppm)

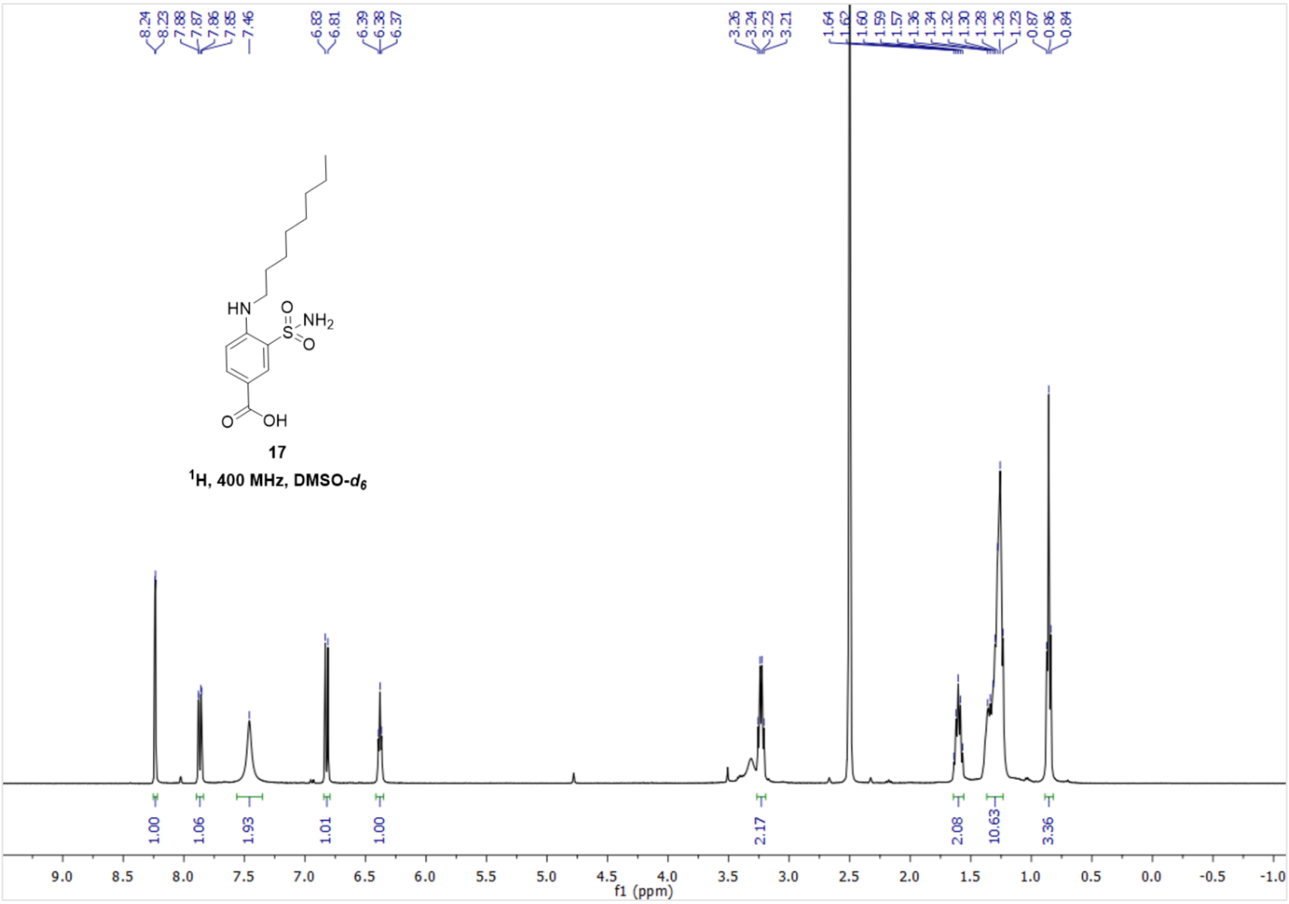

HN
NH2
OH
17
1H, 400 MHz, DMSO-d6
f1 (ppm)

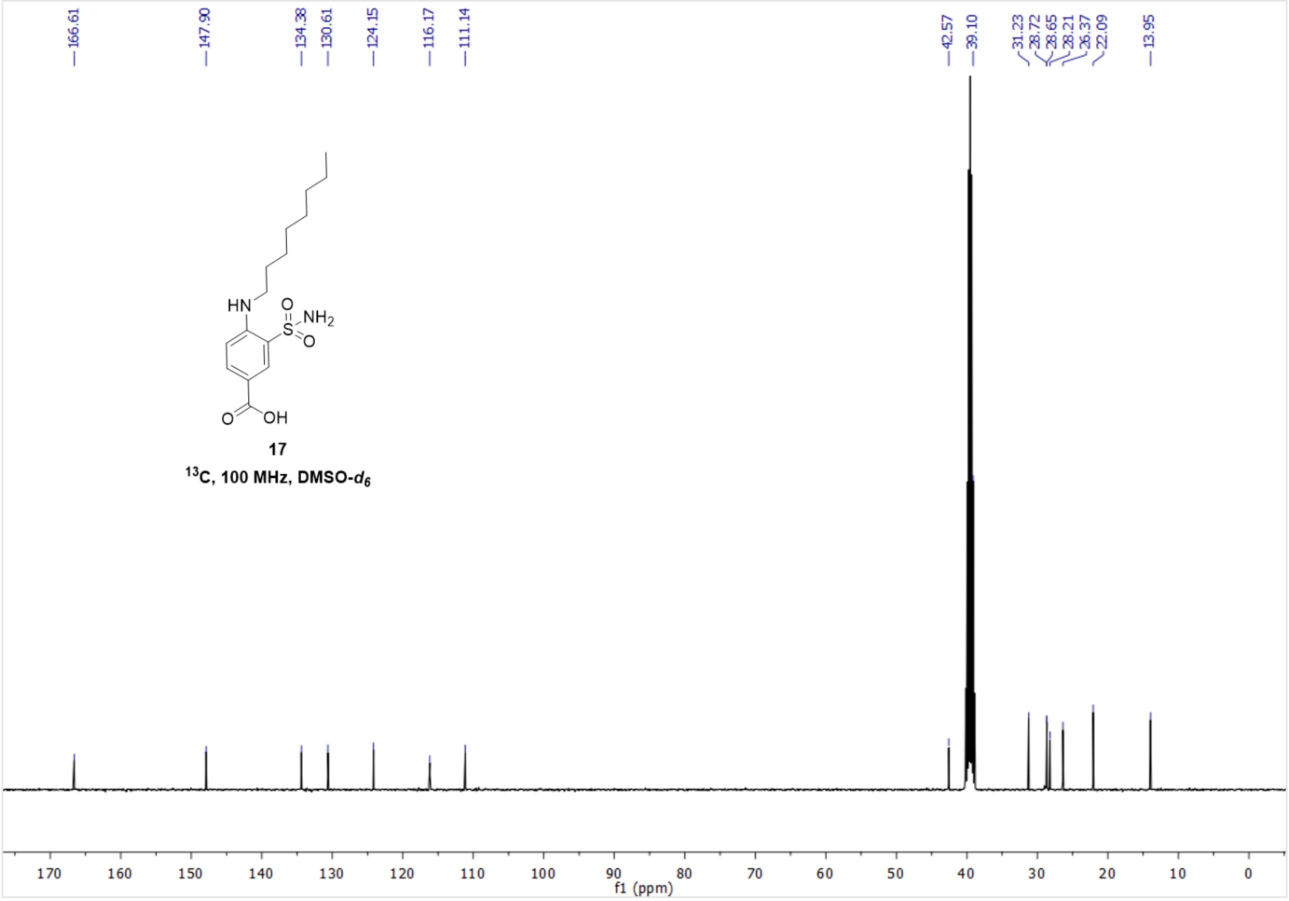

HN
NH2
OH
17
13C, 100 MHz, DMSO-d6
f1 (ppm)

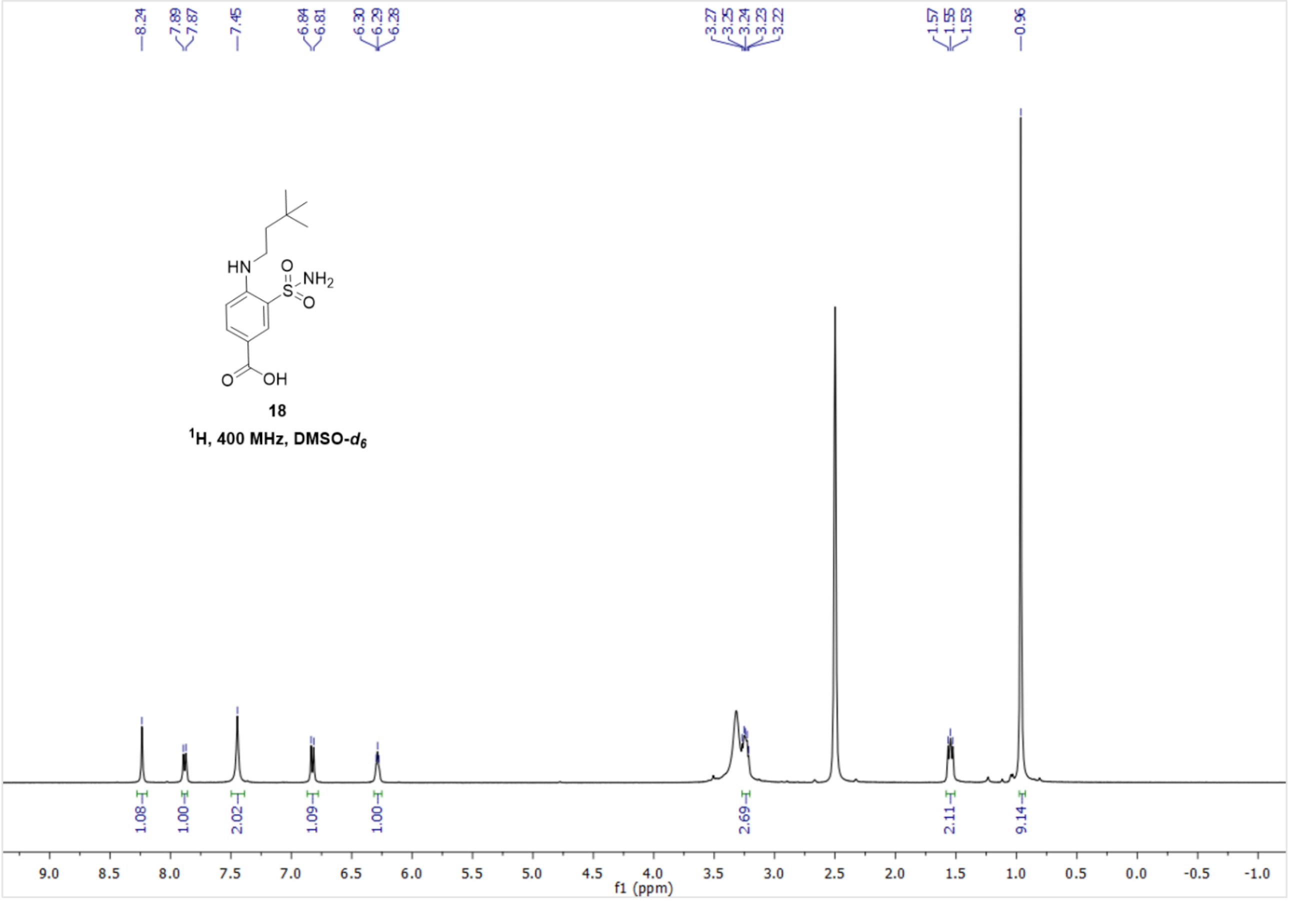
8.24
7.89
7.87
7.45
6.84
6.81
6.30
6.29
6.28
3.27
3.25
3.24
3.23
3.22
1.57
1.55
1.53
0.96
HN
O
S
NH2
O
O
OH
18
1H, 400 MHz, DMSO-d6
1.08
1.00
2.02
1.09
1.00
2.69
2.11
9.14
f1 (ppm)

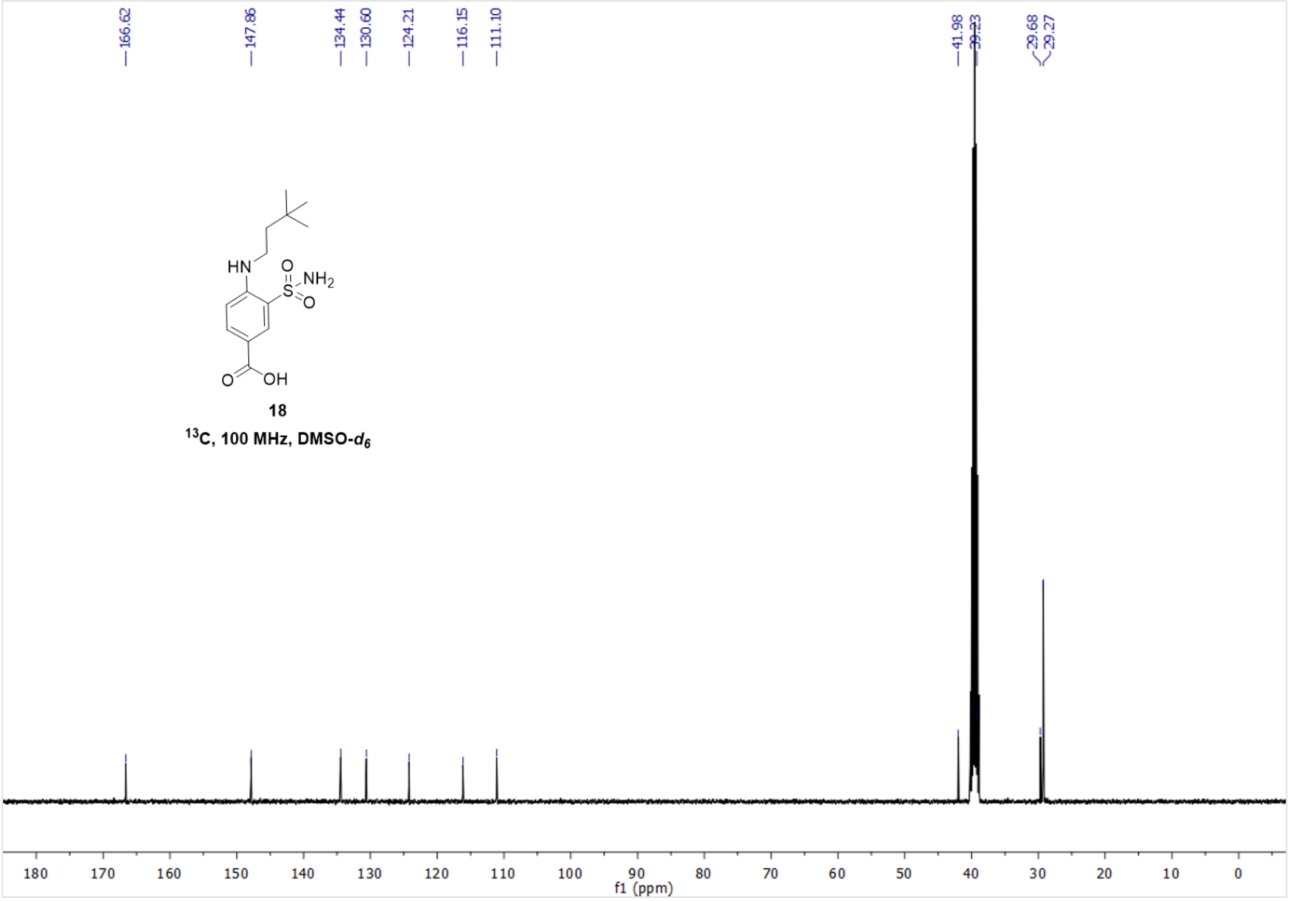
166.62
147.86
134.44
130.60
124.21
116.15
111.10
41.98
39.23
29.68
29.27
HN
O
S
NH2
O
O
OH
18
13C, 100 MHz, DMSO-d6
f1 (ppm)

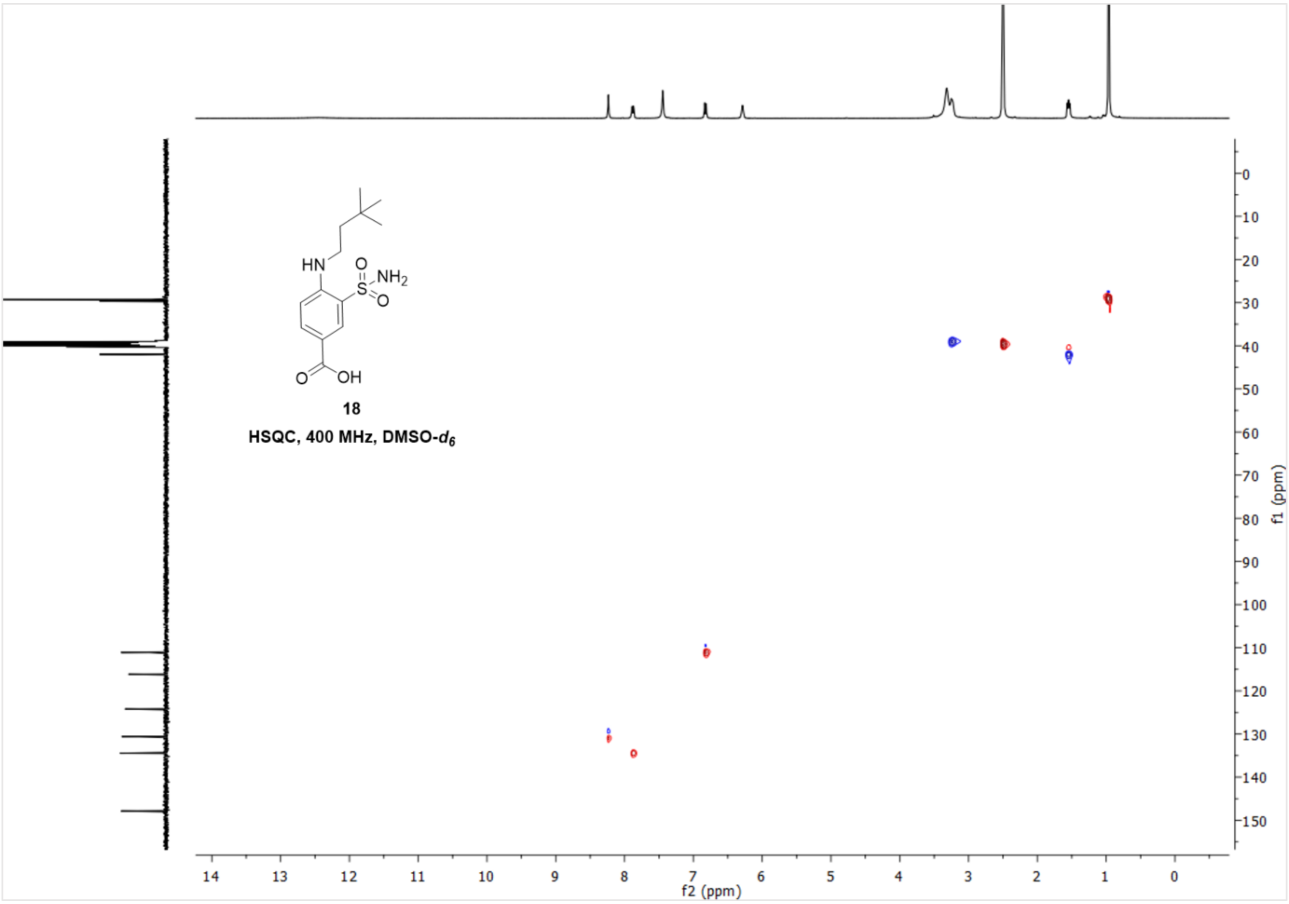

18
HSQC, 400 MHz, DMSO-d_6
f2 (ppm)
f1 (ppm)

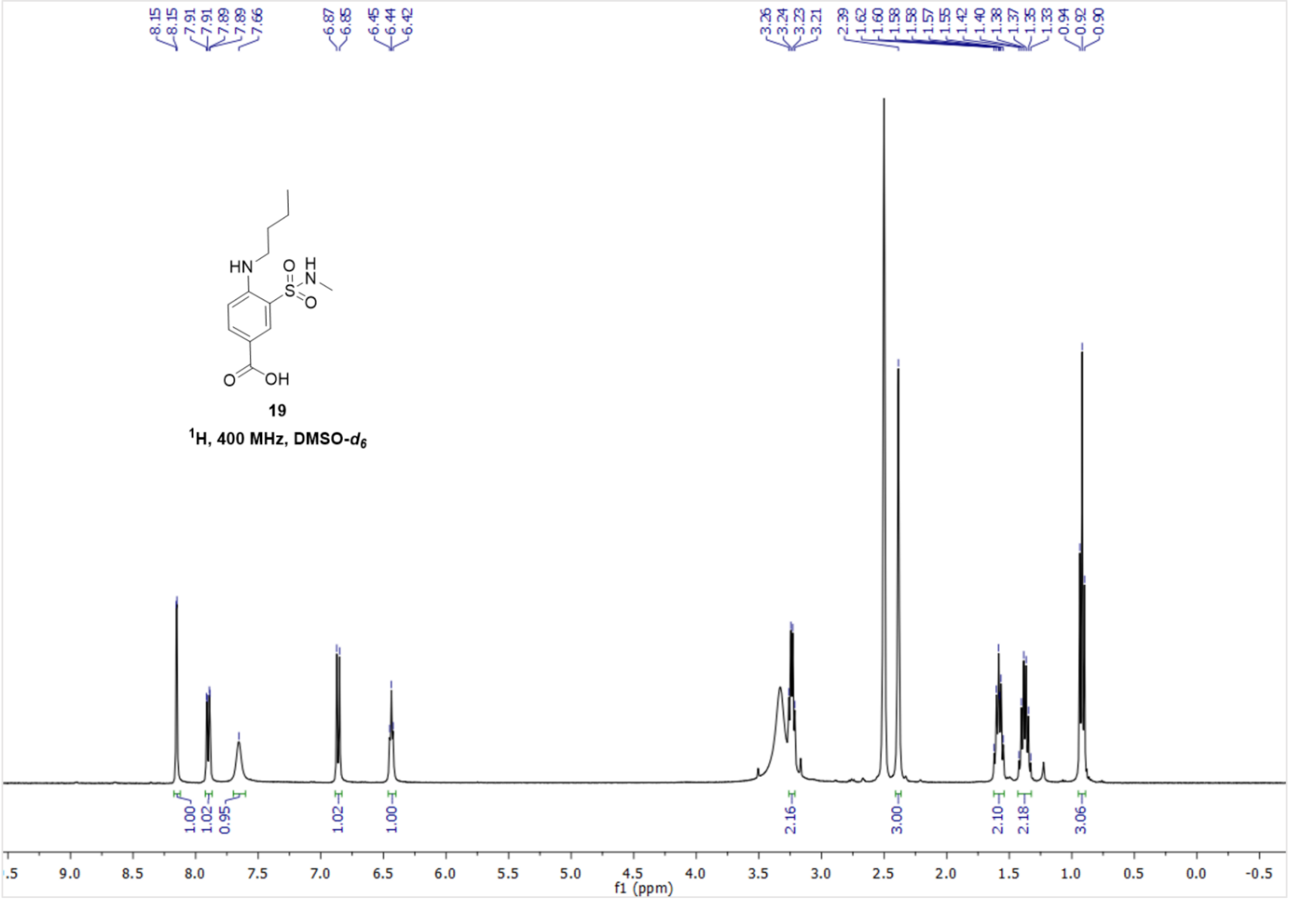

19
^{1}H, 400 MHz, DMSO-d_6
f1 (ppm)

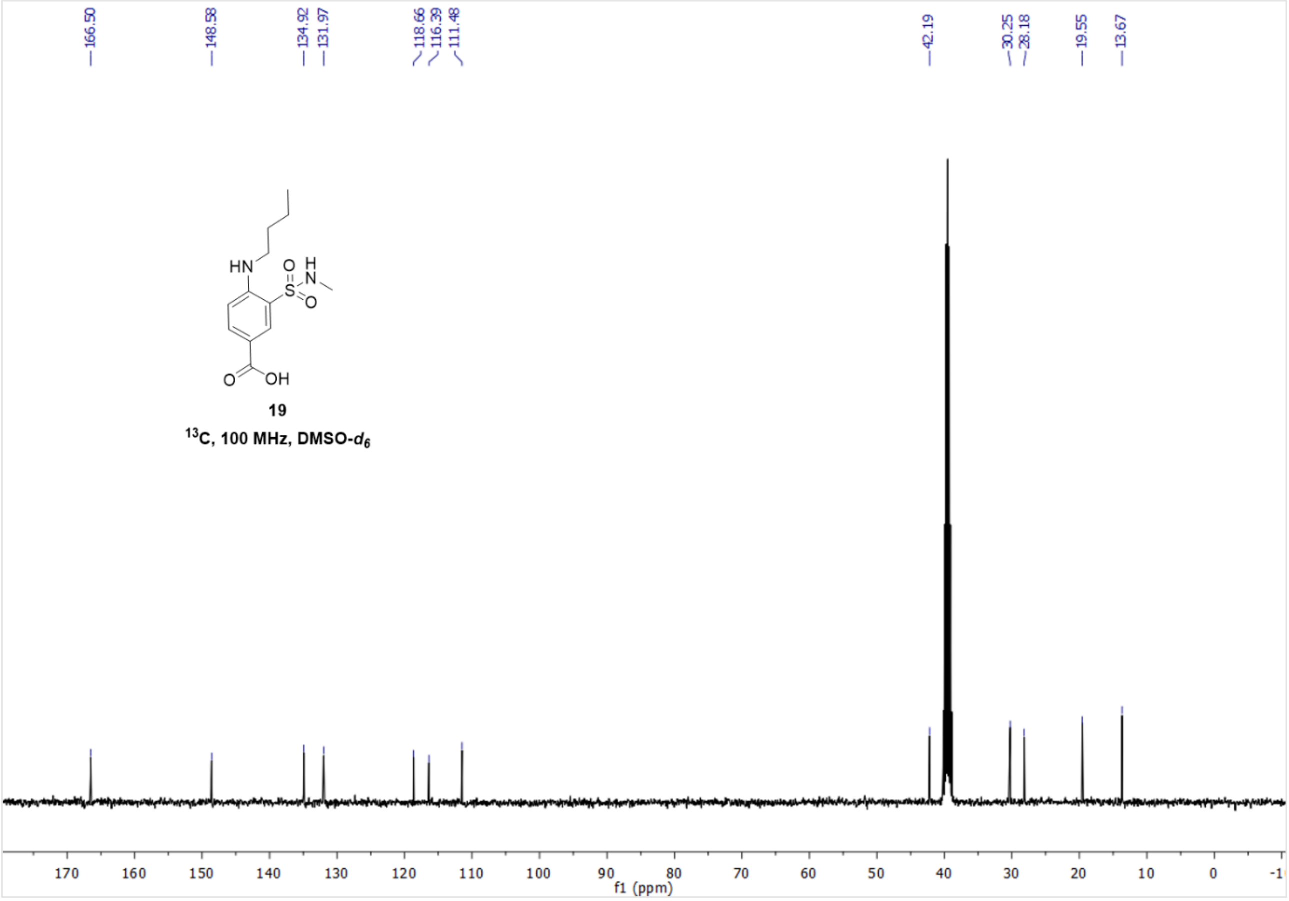

166.50
148.58
134.92
131.97
118.66
116.39
111.48
42.19
30.25
28.18
19.55
13.67
HN
O
H
S
N
O
O
OH
19
^{13}C, 100 MHz, DMSO-d_6
170 160 150 140 130 120 110 100 90 80 70 60 50 40 30 20 10 0 -1
f1 (ppm)

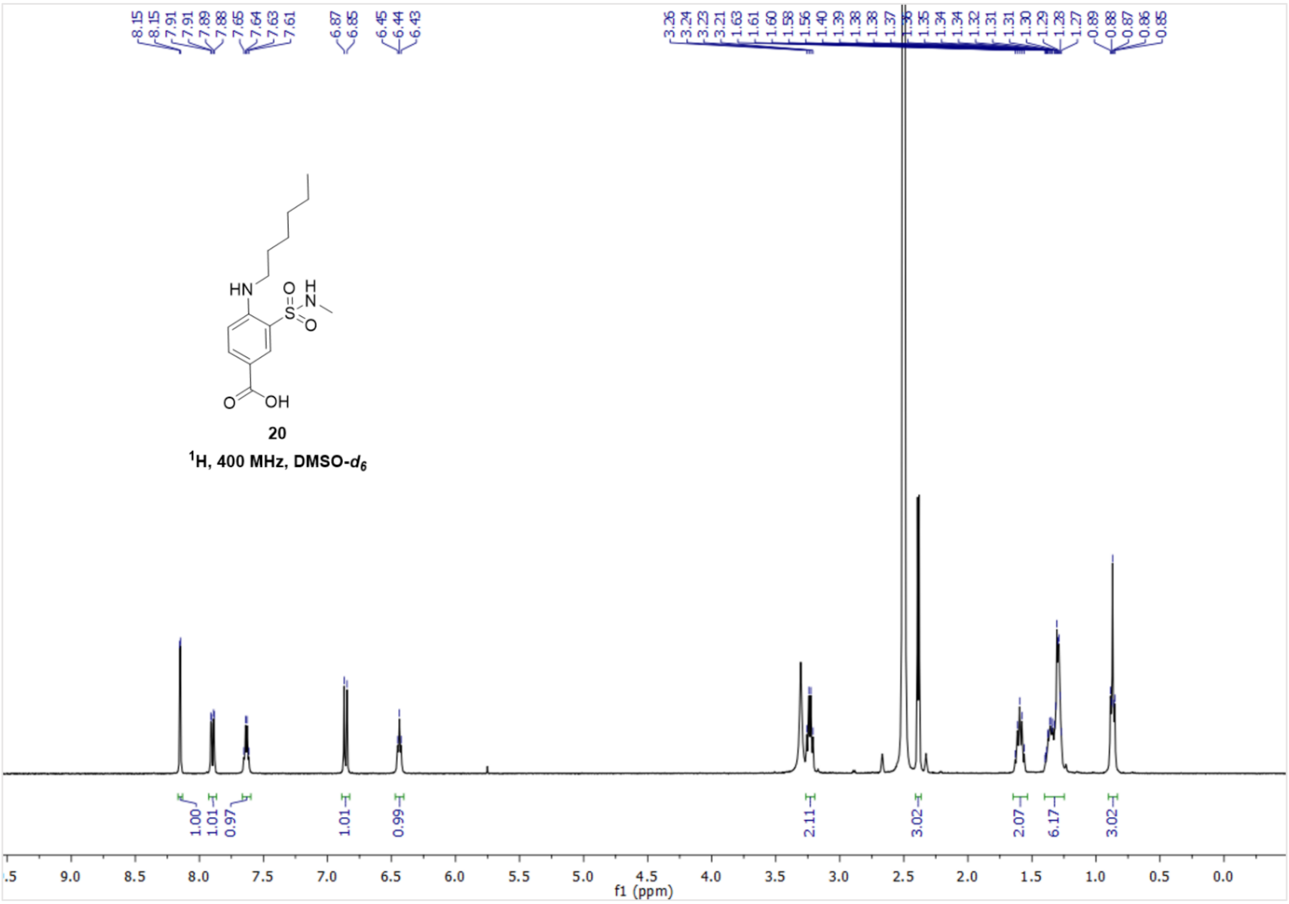

8.15
8.15
7.91
7.89
7.88
7.65
7.64
7.63
7.61
6.87
6.85
6.45
6.44
6.43
3.26
3.24
3.23
3.21
1.63
1.61
1.60
1.58
1.56
1.40
1.39
1.38
1.38
1.37
1.36
1.35
1.34
1.34
1.32
1.31
1.31
1.30
1.29
1.28
1.27
0.89
0.88
0.87
0.86
0.85
HN
O
H
S
N
O
O
OH
20
^{1}H, 400 MHz, DMSO-d_6
1.00
1.01
0.97
1.01
0.99
2.11
3.02
2.07
6.17
3.02
.5 9.0 8.5 8.0 7.5 7.0 6.5 6.0 5.5 5.0 4.5 4.0 3.5 3.0 2.5 2.0 1.5 1.0 0.5 0.0
f1 (ppm)

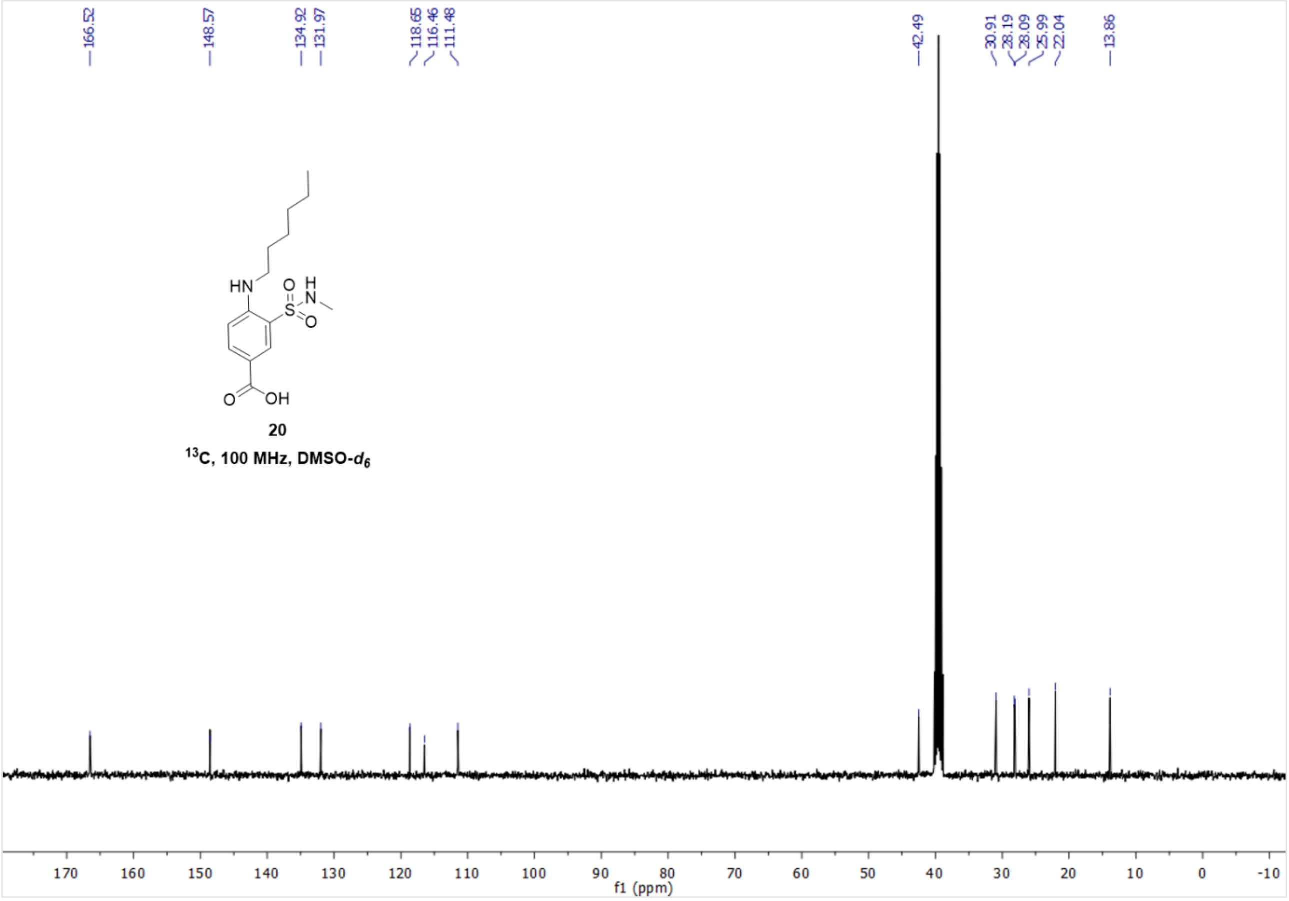

20

^{13}C, 100 MHz, DMSO-d_6

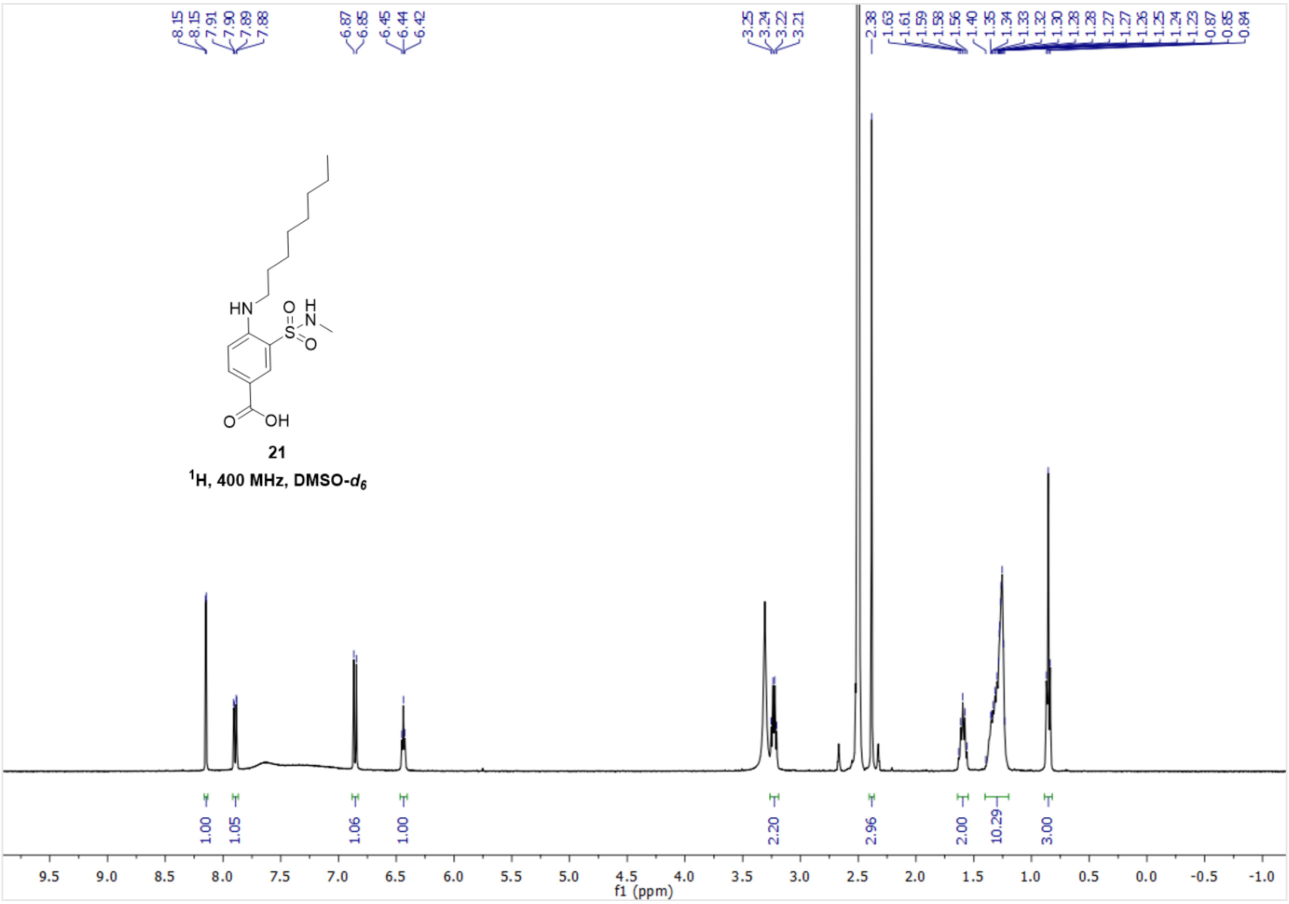

21

^{1}H, 400 MHz, DMSO-d_6

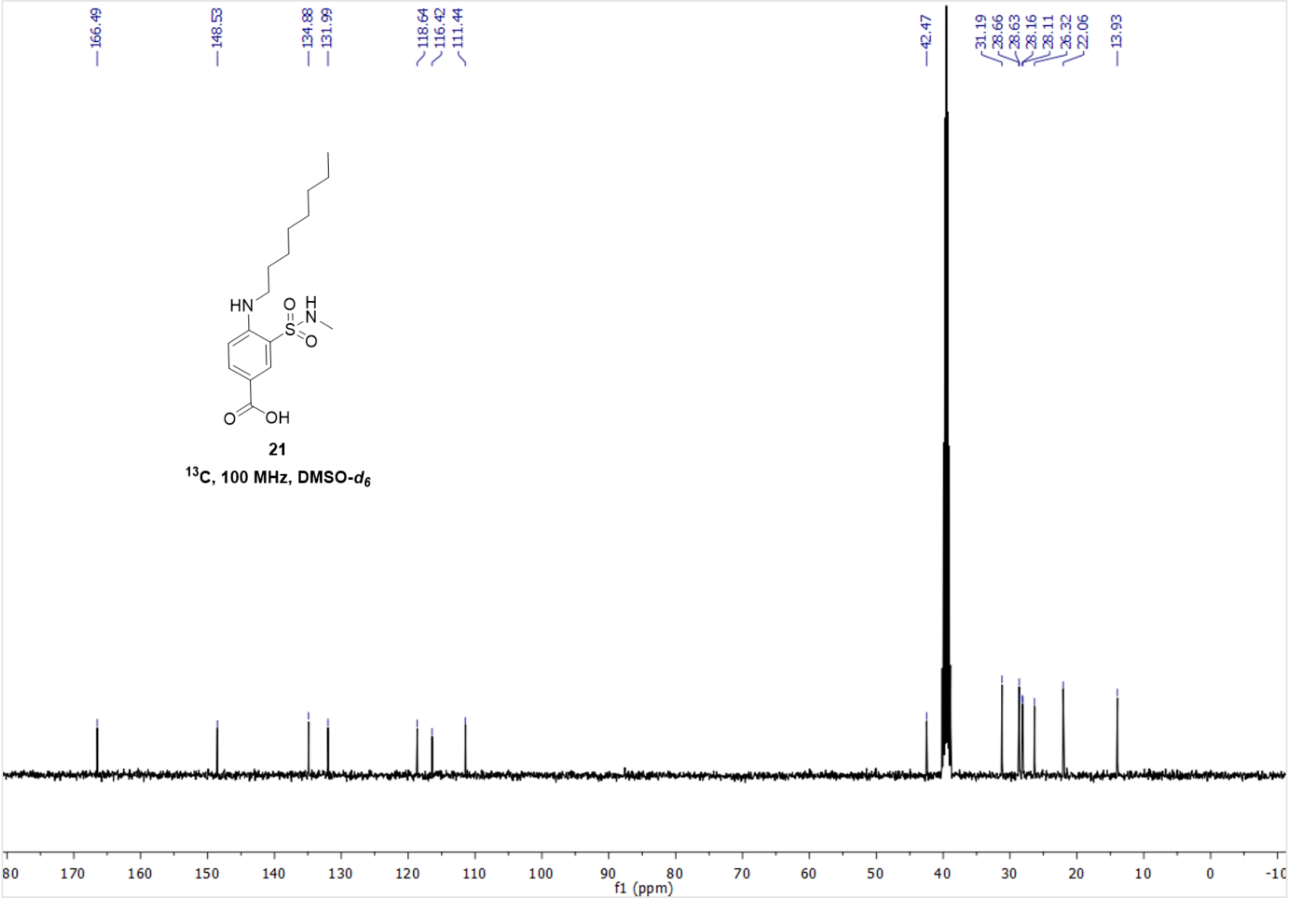

21

^{13}C, 100 MHz, DMSO-d_6

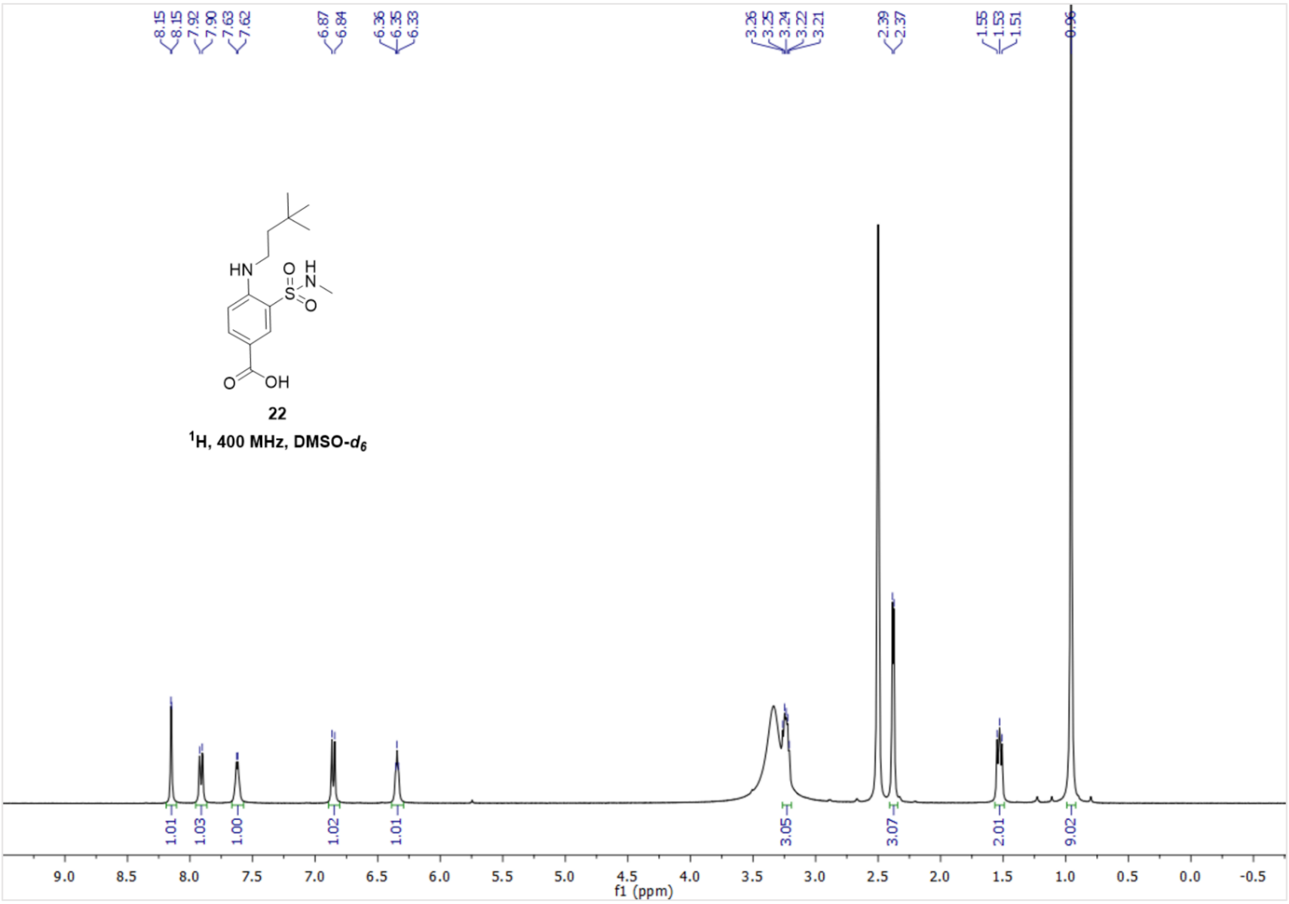

22

^{1}H, 400 MHz, DMSO-d_6

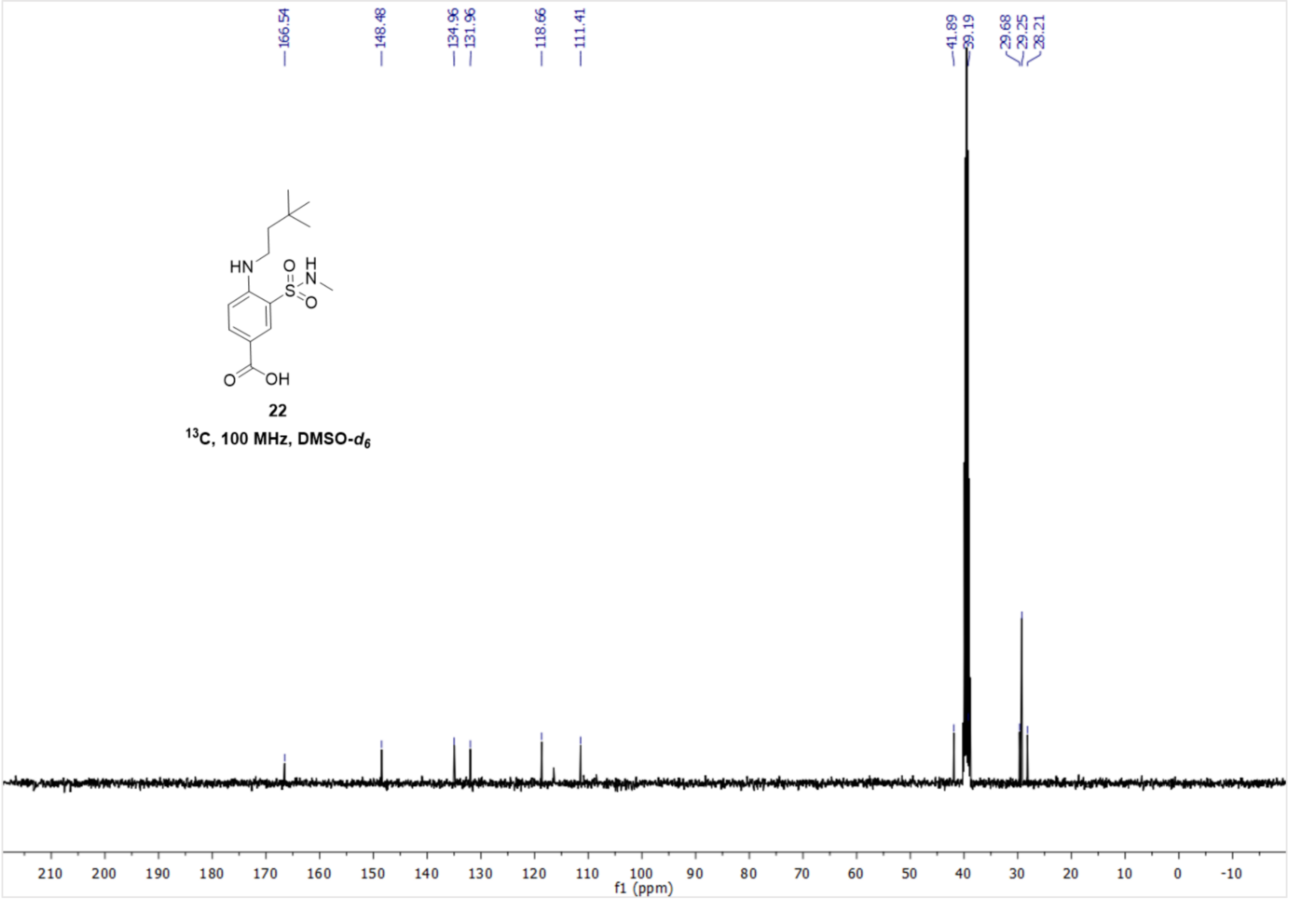
166.54
148.48
134.96
131.96
118.66
111.41
41.89
39.19
29.68
29.25
28.21
HN
O
H
S
N
O
O
OH
22
13C, 100 MHz, DMSO-d6
f1 (ppm)

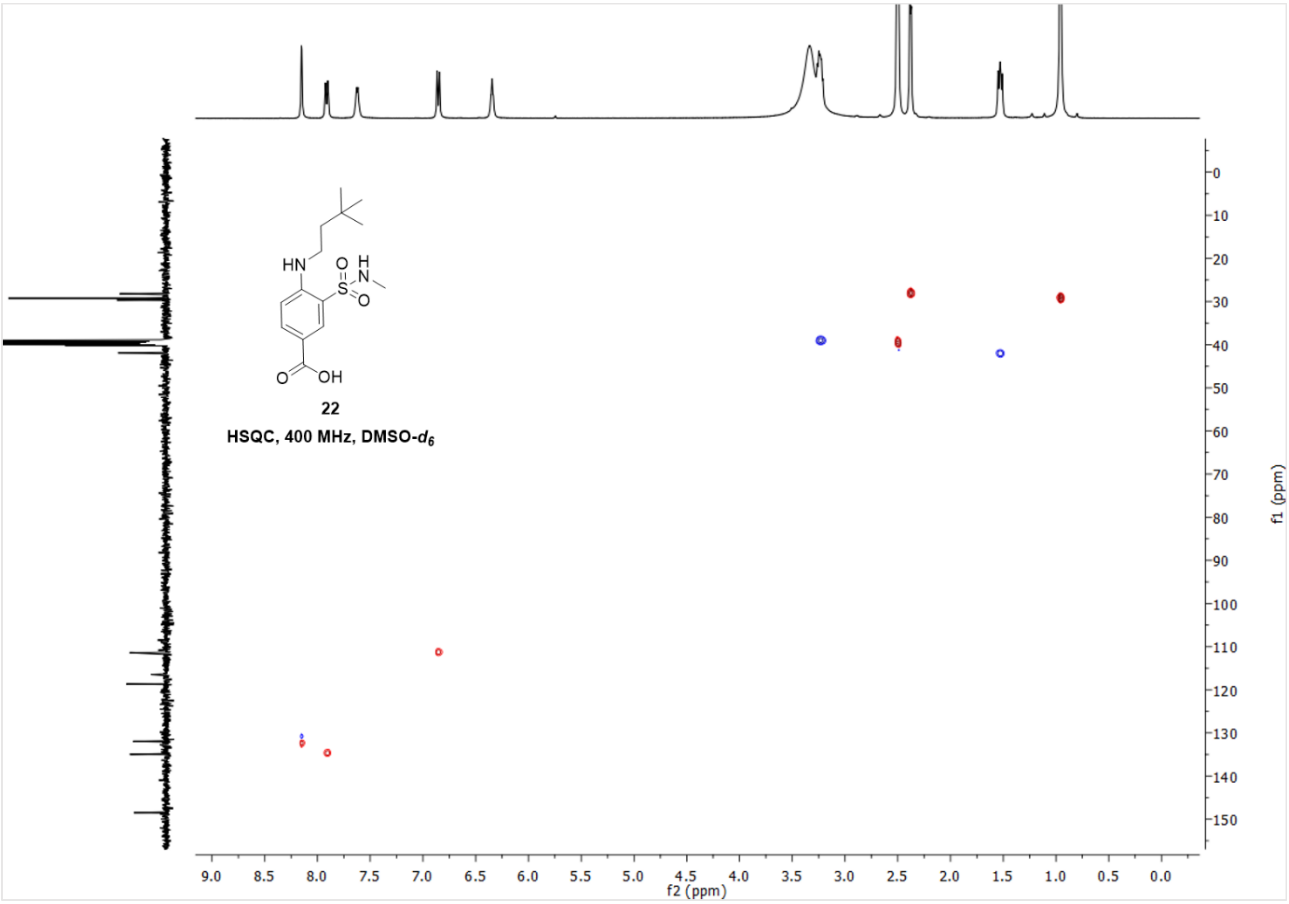
HN
O
H
S
N
O
O
OH
22
HSQC, 400 MHz, DMSO-d6
f2 (ppm)
f1 (ppm)

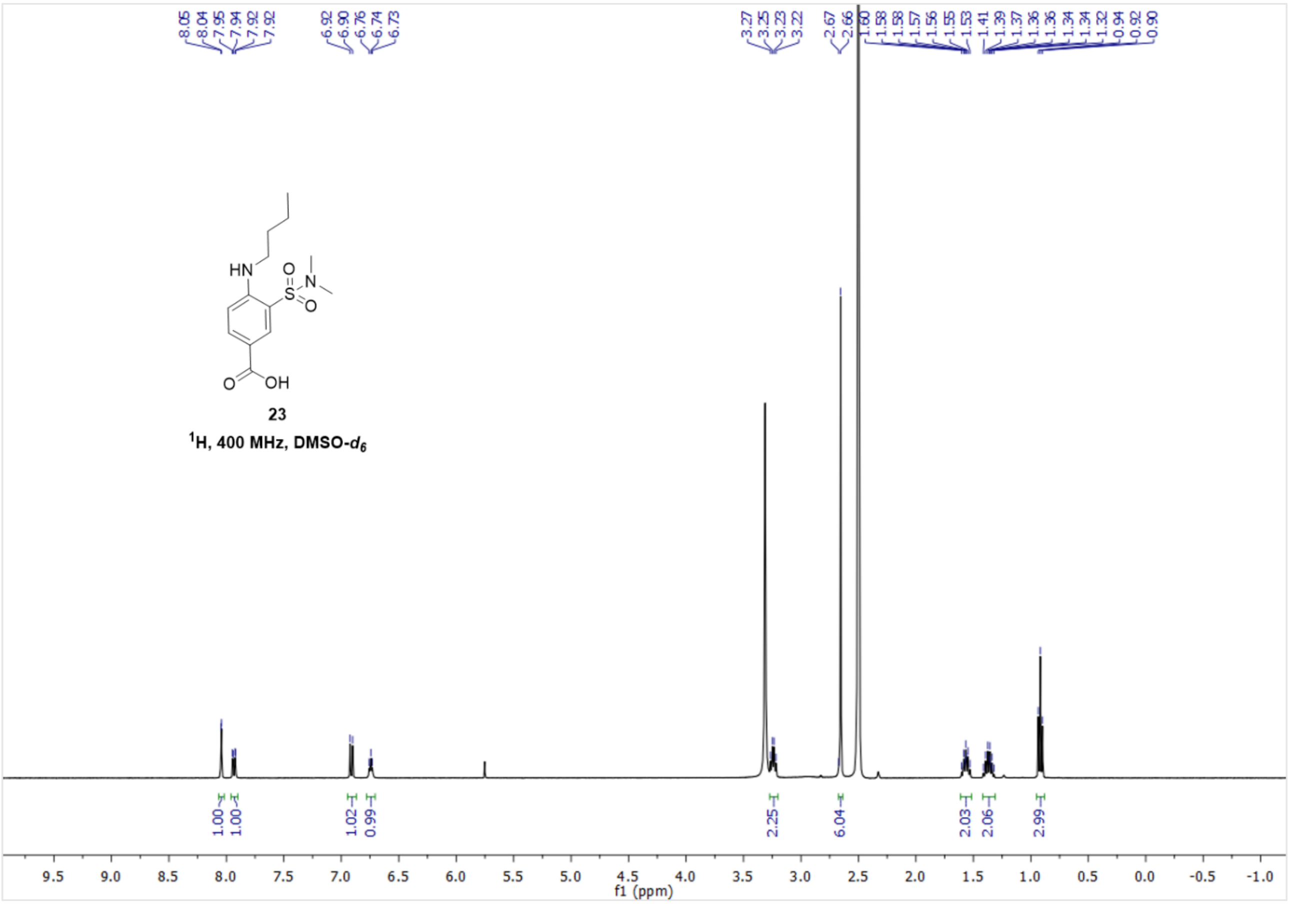
HN
O
S
N
O
O
OH
23
1H, 400 MHz, DMSO-d6
f1 (ppm)

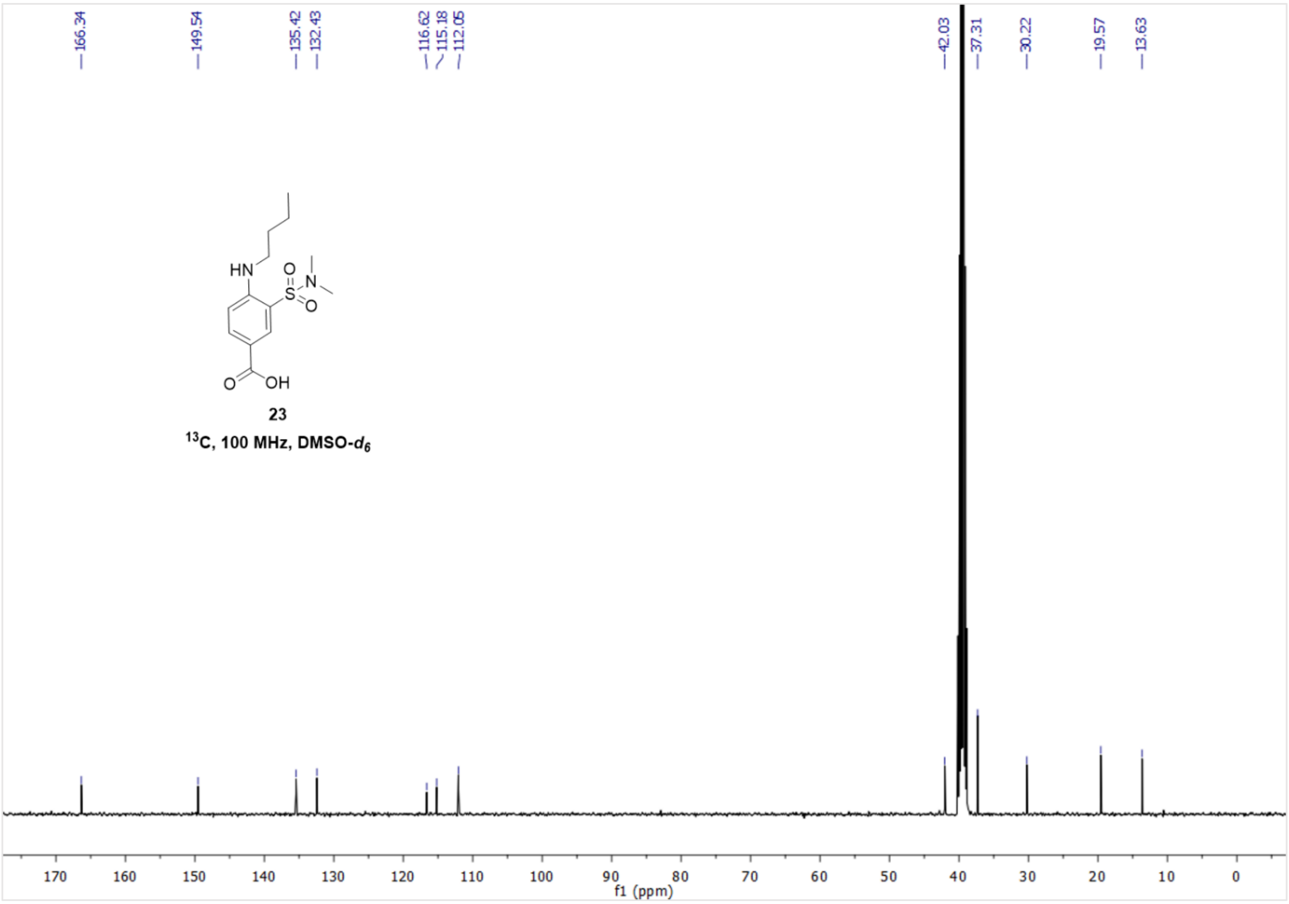
HN
O
S
N
O
O
OH
23
13C, 100 MHz, DMSO-d6
f1 (ppm)

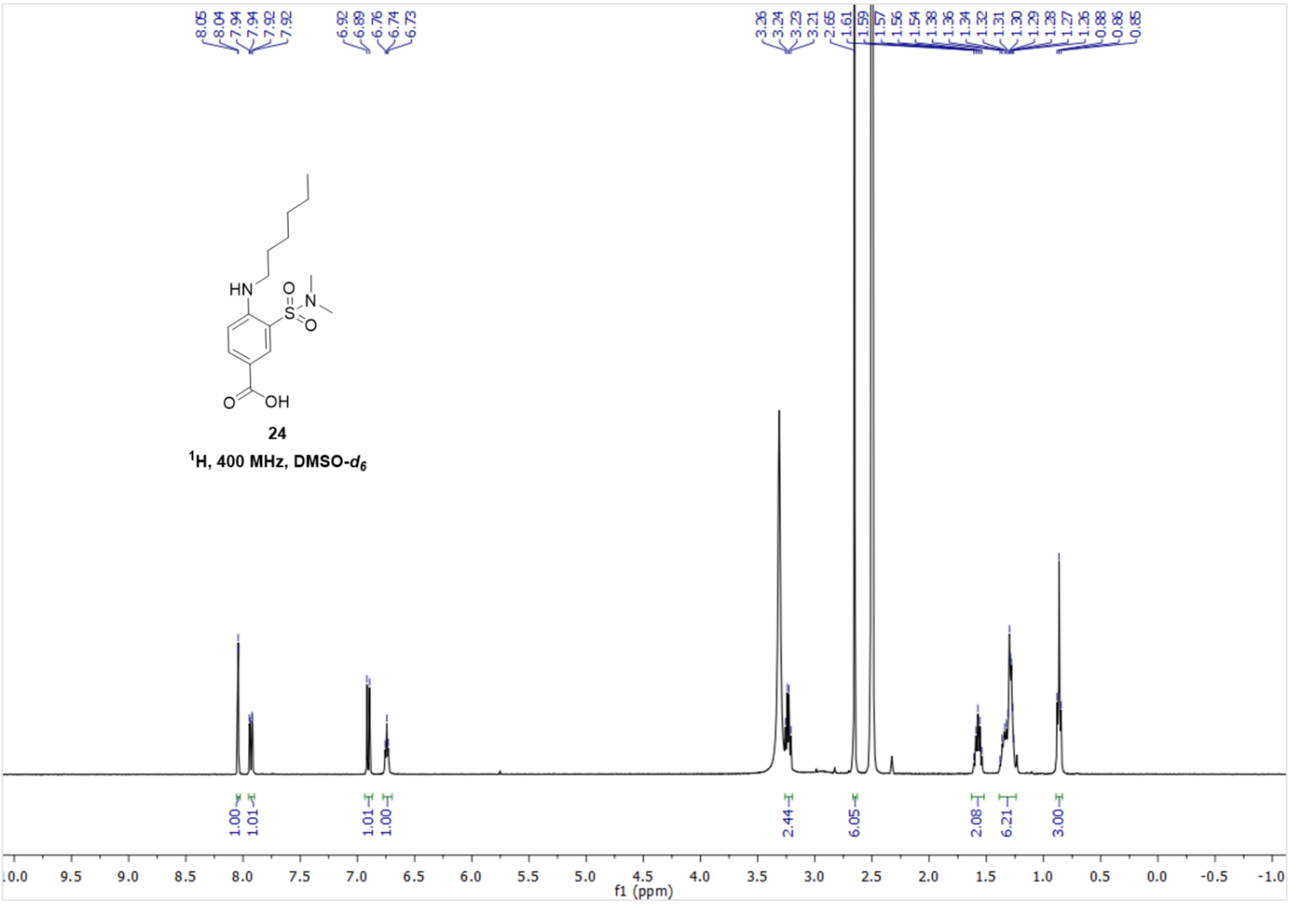
8.05 8.04 7.94 7.94 7.92 7.92 6.92 6.89 6.76 6.74 6.73
3.26 3.24 3.23 3.21 2.65 1.61 1.59 1.57 1.56 1.54 1.38 1.36 1.34 1.32 1.31 1.30 1.29 1.28 1.27 1.26 0.88 0.86 0.85
24
^{1}H, 400 MHz, DMSO-d_6
1.00 1.01 1.01 1.00 2.44 6.05 2.08 6.21 3.00
f1 (ppm)

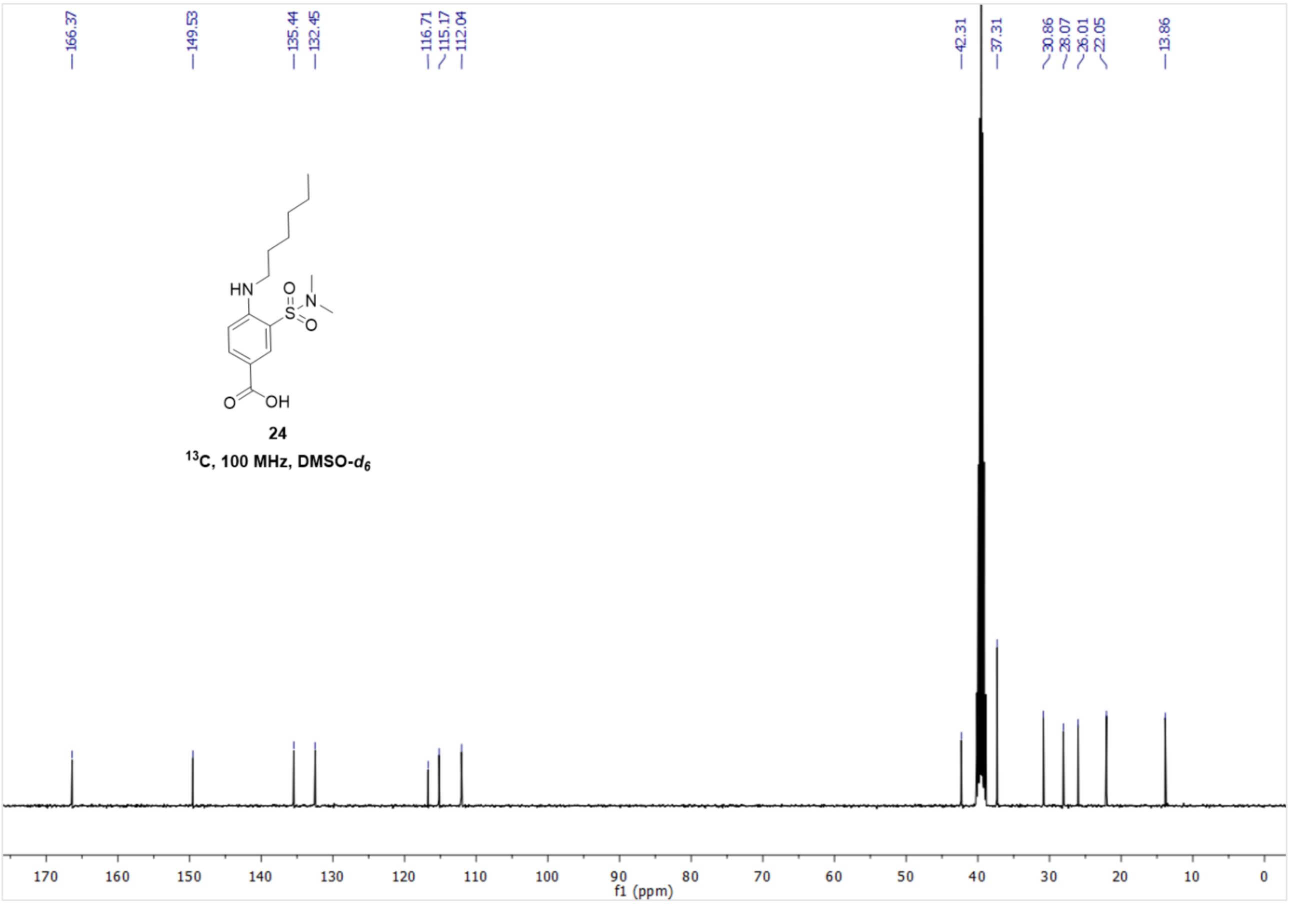
166.37 149.53 135.44 132.45 116.71 115.17 112.04 42.31 37.31 30.86 28.07 26.01 22.05 13.86
24
^{13}C, 100 MHz, DMSO-d_6
f1 (ppm)

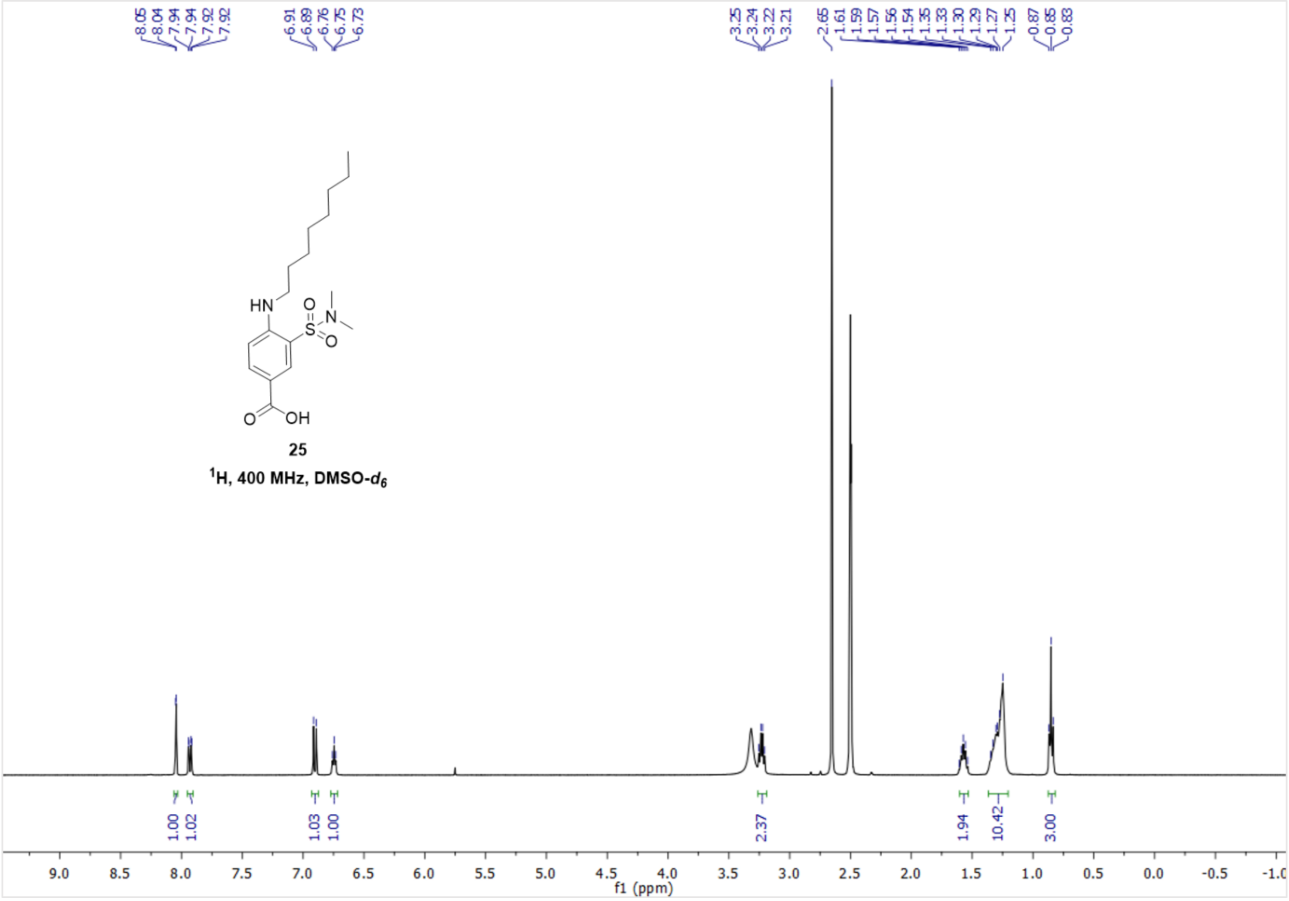
25
^{1}H, 400 MHz, DMSO-d_6
f1 (ppm)

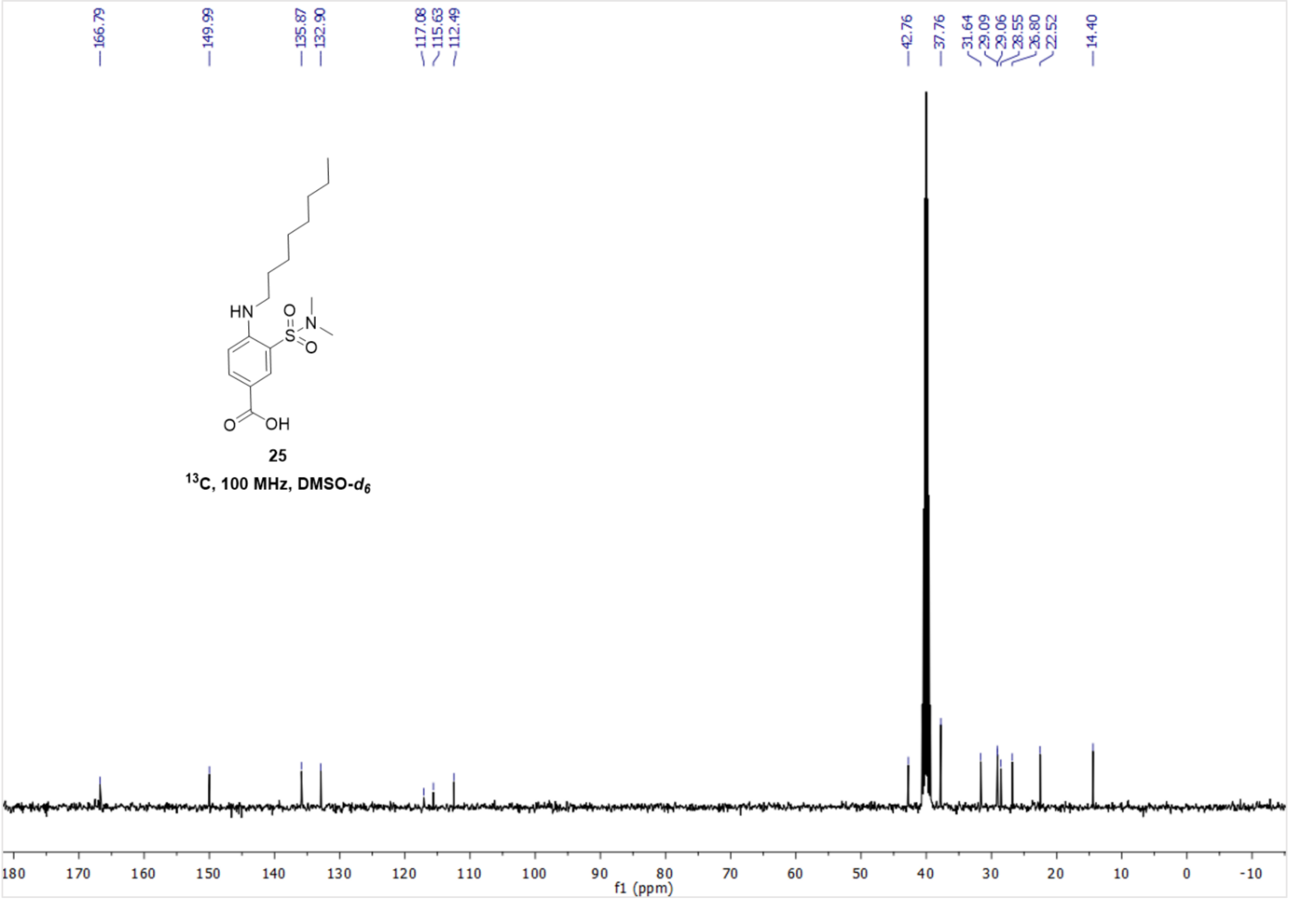
25
^{13}C, 100 MHz, DMSO-d_6
f1 (ppm)

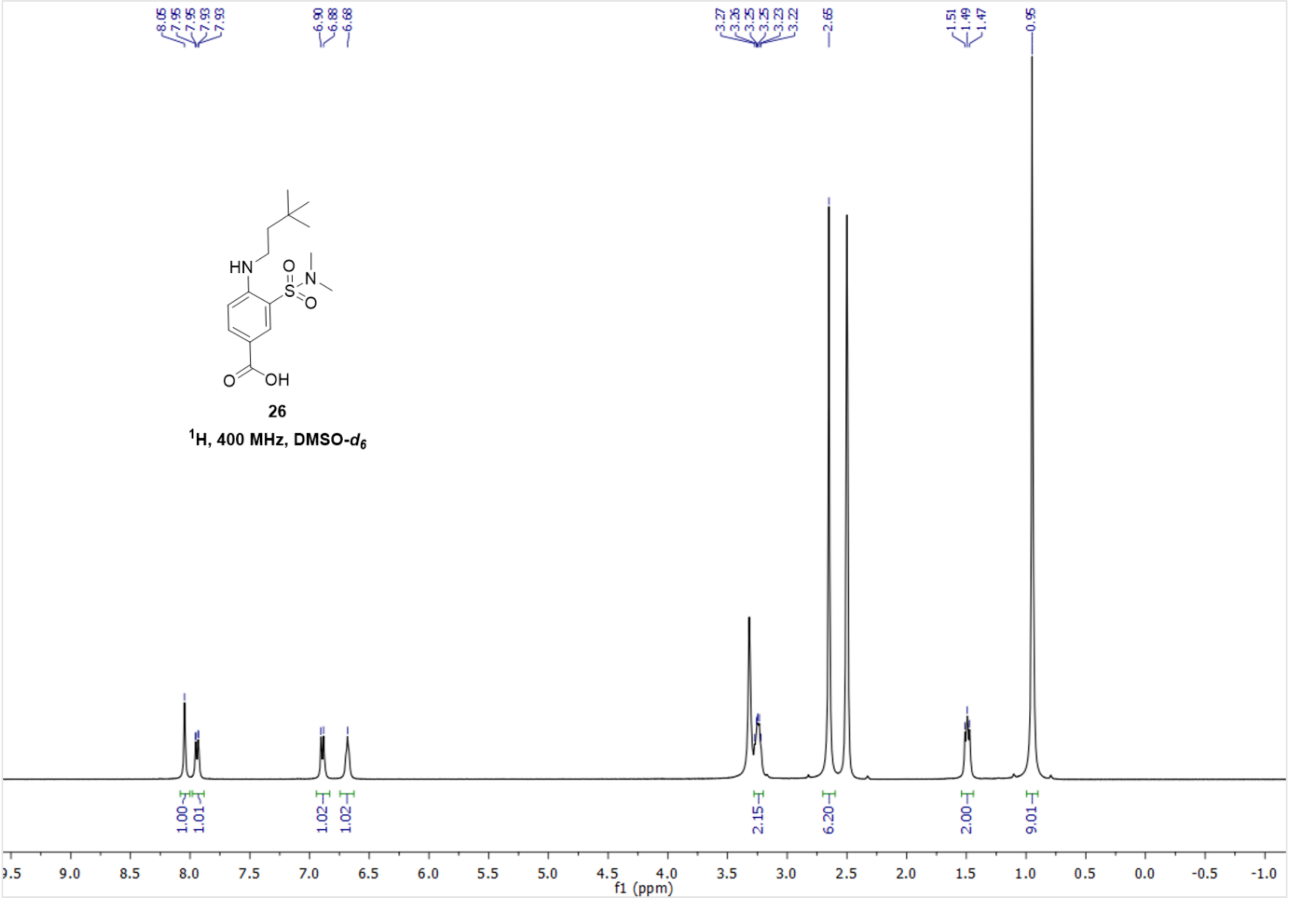
8.05
7.95
7.95
7.93
7.93
6.90
6.88
6.68
3.27
3.26
3.25
3.25
3.23
3.22
2.65
1.51
1.49
1.47
0.95
HN
O
S
N
O
OH
O
26
1H, 400 MHz, DMSO-d6
1.00
1.01
1.02
1.02
2.15
6.20
2.00
9.01
9.5
9.0
8.5
8.0
7.5
7.0
6.5
6.0
5.5
5.0
4.5
4.0
3.5
3.0
2.5
2.0
1.5
1.0
0.5
0.0
-0.5
-1.0
f1 (ppm)

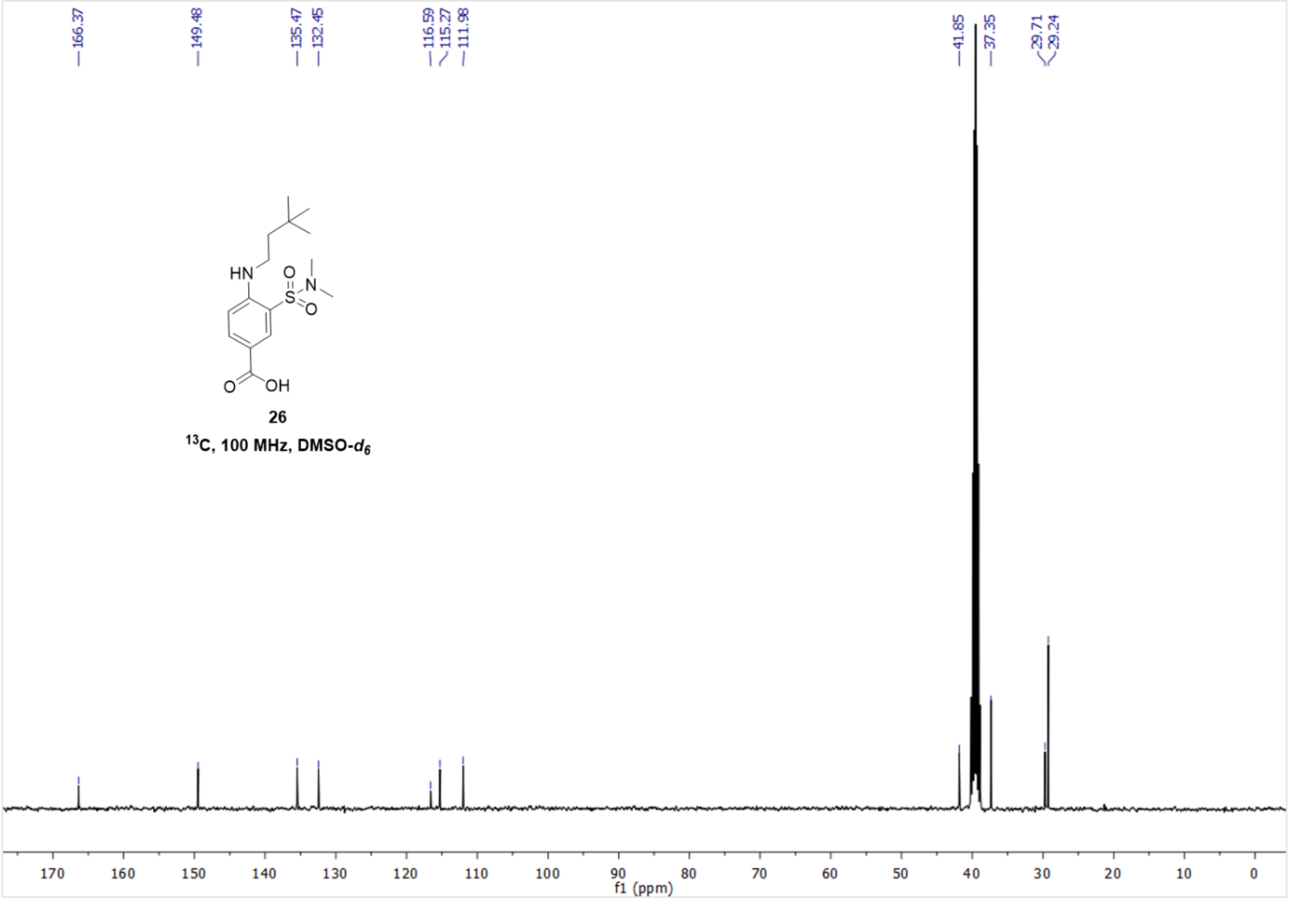
166.37
149.48
135.47
132.45
116.59
115.27
111.98
41.85
37.35
29.71
29.24
HN
O
S
N
O
OH
O
26
13C, 100 MHz, DMSO-d6
170
160
150
140
130
120
110
100
90
80
70
60
50
40
30
20
10
0
f1 (ppm)

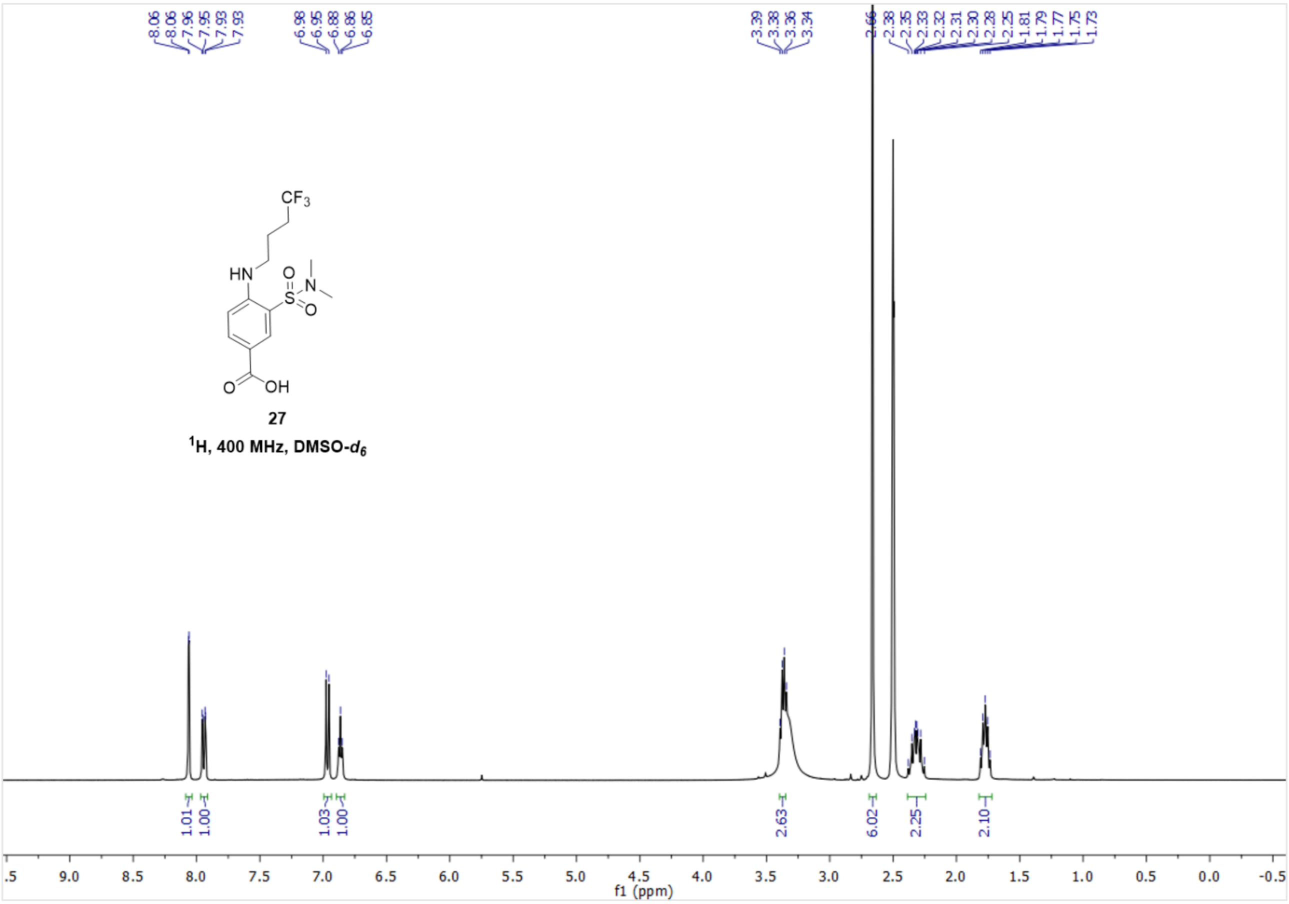
8.06
8.06
7.96
7.95
7.93
7.93
6.98
6.95
6.88
6.86
6.85
3.39
3.38
3.36
3.34
2.66
2.38
2.35
2.33
2.32
2.31
2.30
2.28
2.25
1.81
1.79
1.77
1.75
1.73
CF3
HN
O
S
N
O
O
OH
27
1H, 400 MHz, DMSO-d6
1.01
1.00
1.03
1.00
2.63
6.02
2.25
2.10
f1 (ppm)

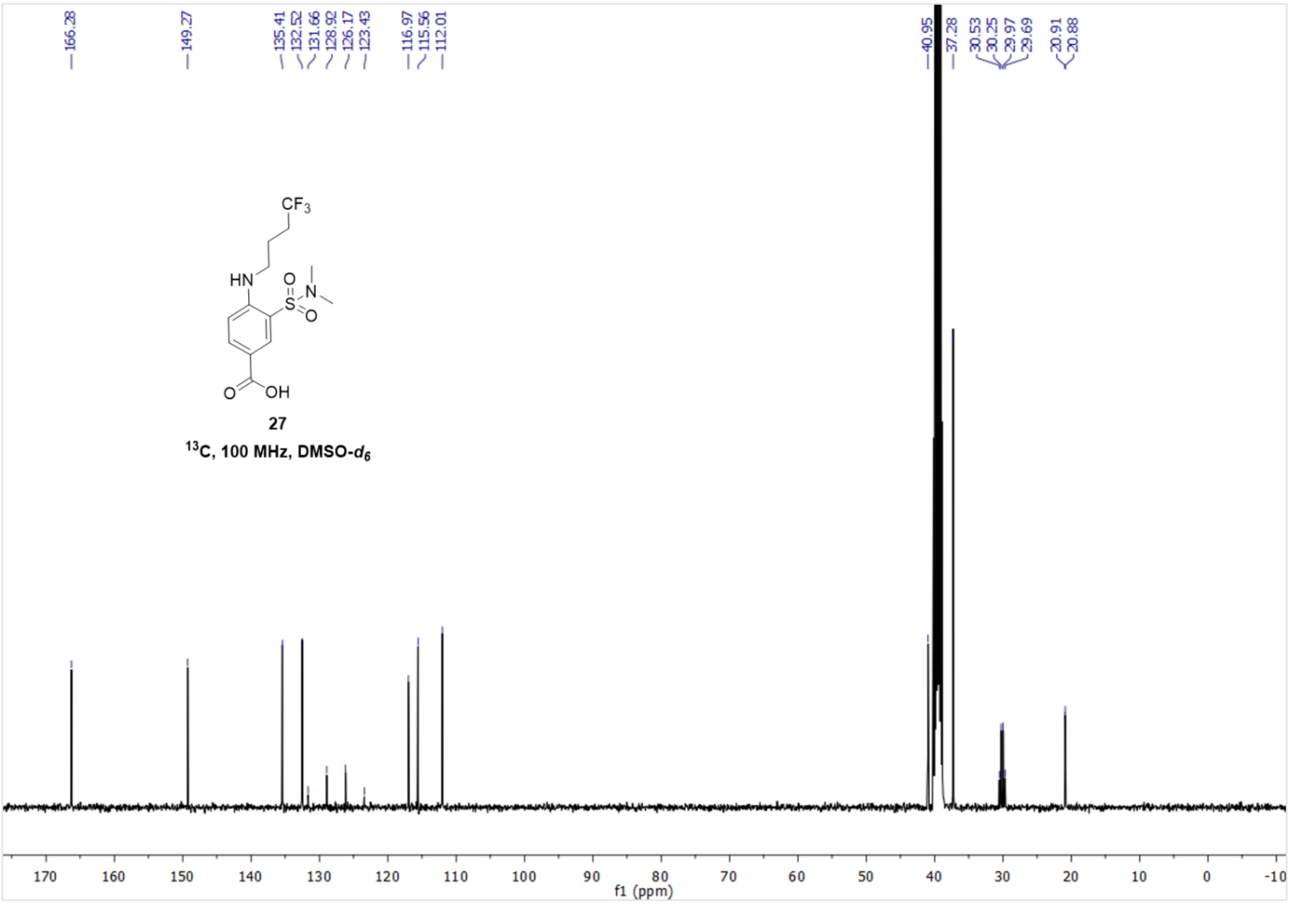
166.28
149.27
135.41
132.52
131.66
128.92
126.17
123.43
116.97
115.56
112.01
40.95
37.28
30.53
30.25
29.97
29.69
20.91
20.88
CF3
HN
O
S
N
O
O
OH
27
13C, 100 MHz, DMSO-d6
f1 (ppm)

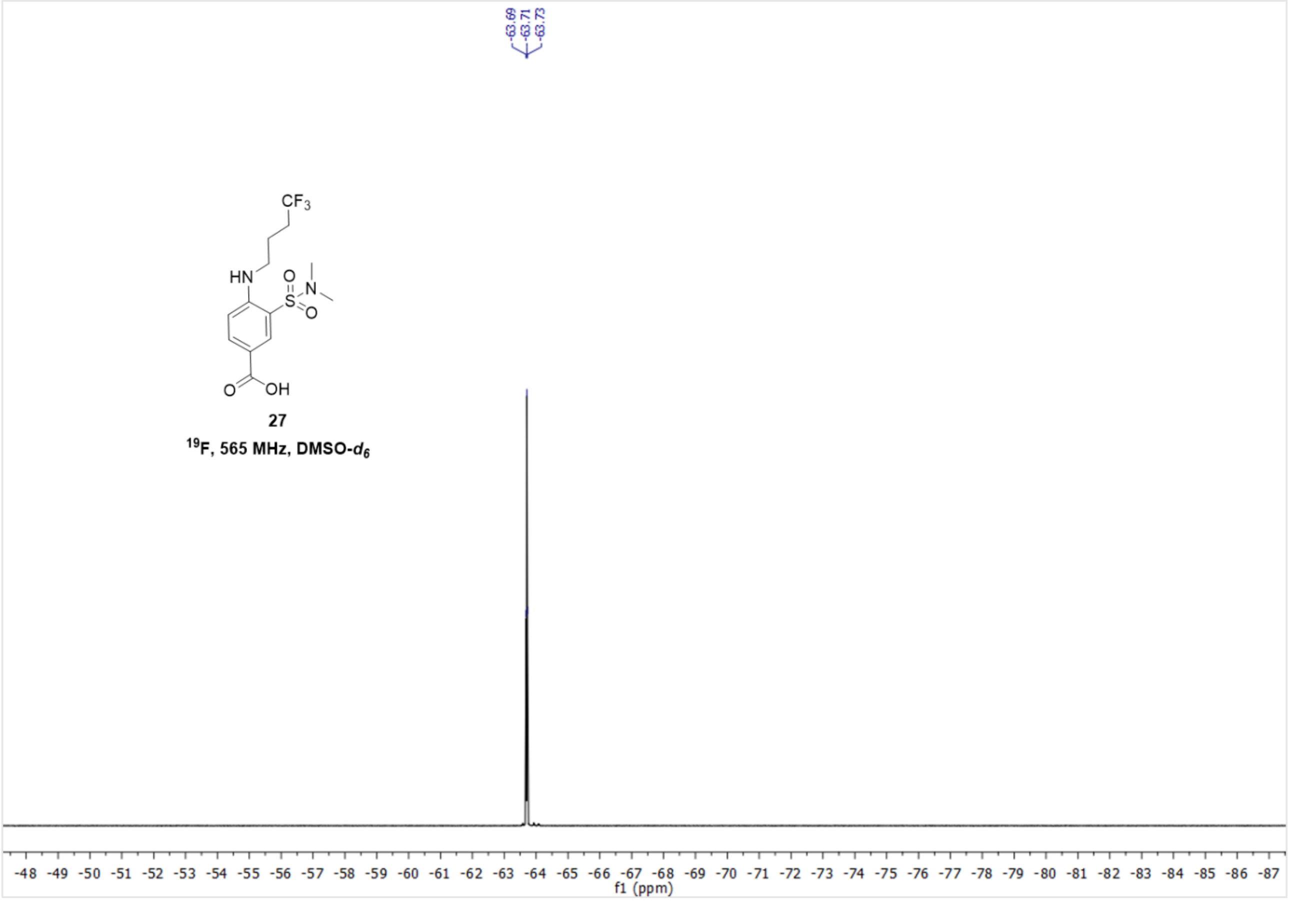

-63.69
-63.71
-63.73
CF3
HN
O
S
N
O
O
OH
27
19F, 565 MHz, DMSO-d6
f1 (ppm)

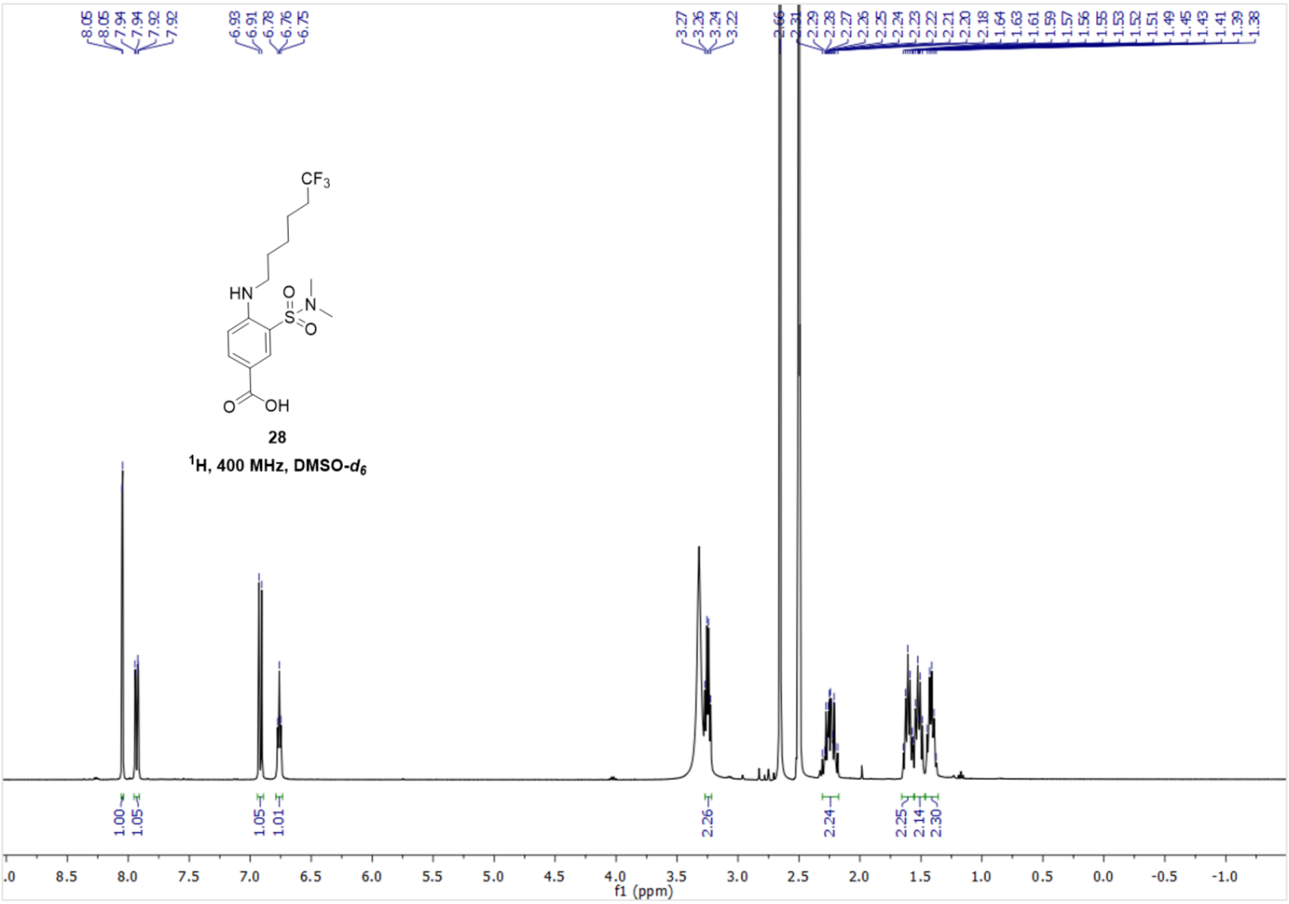

CF3
HN
O
S
N
O
O
OH
28
1H, 400 MHz, DMSO-d6
1.00
1.05
1.05
1.01
2.26
2.24
2.25
2.14
2.30
f1 (ppm)

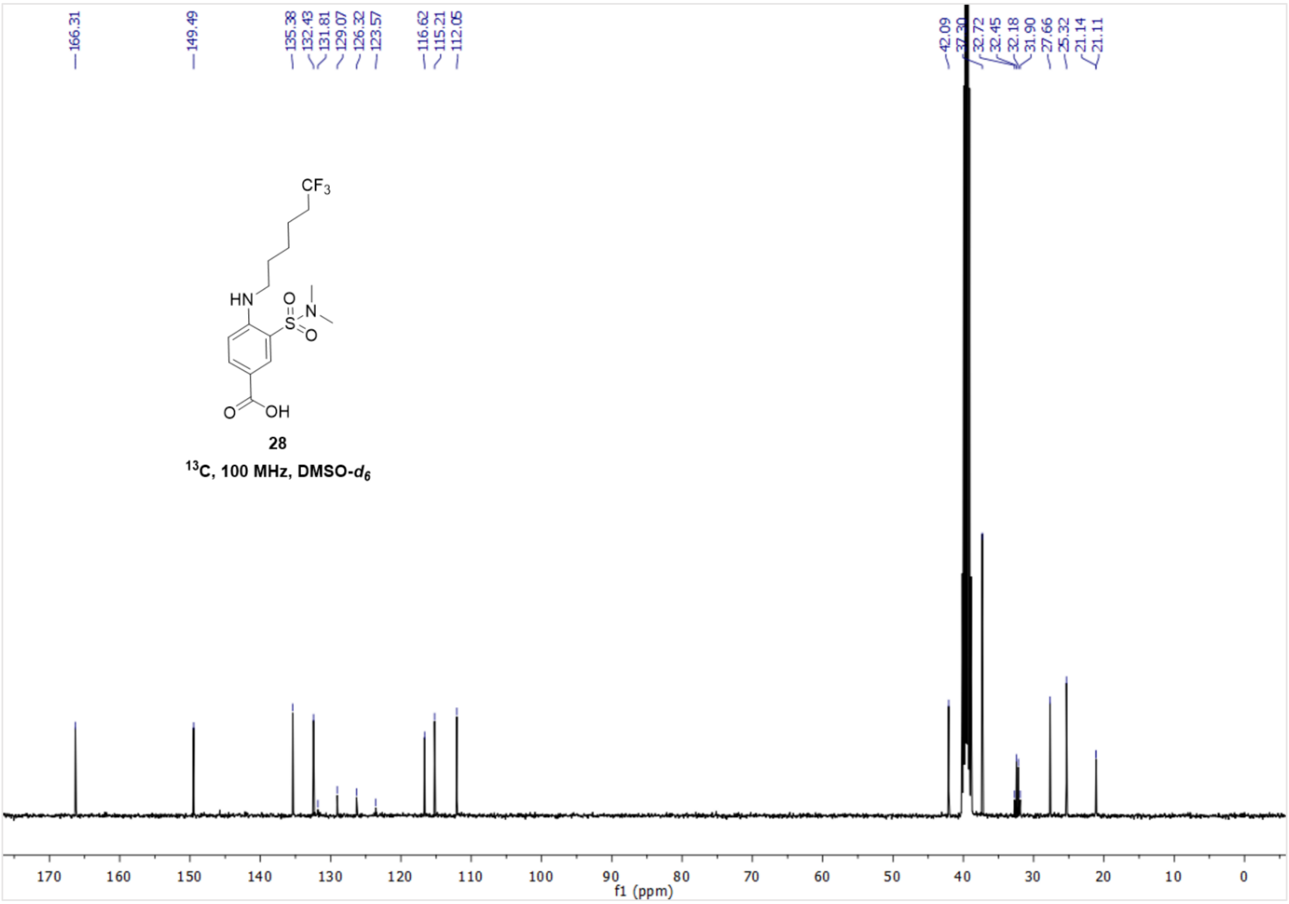
166.31
149.49
135.38
132.43
131.81
129.07
126.32
123.57
116.62
115.21
112.05
42.09
37.30
32.72
32.45
32.18
31.90
27.66
25.32
21.14
21.11
CF3
HN
O
S
N
O
O
OH
28
13C, 100 MHz, DMSO-d6
170 160 150 140 130 120 110 100 90 80 70 60 50 40 30 20 10 0
f1 (ppm)

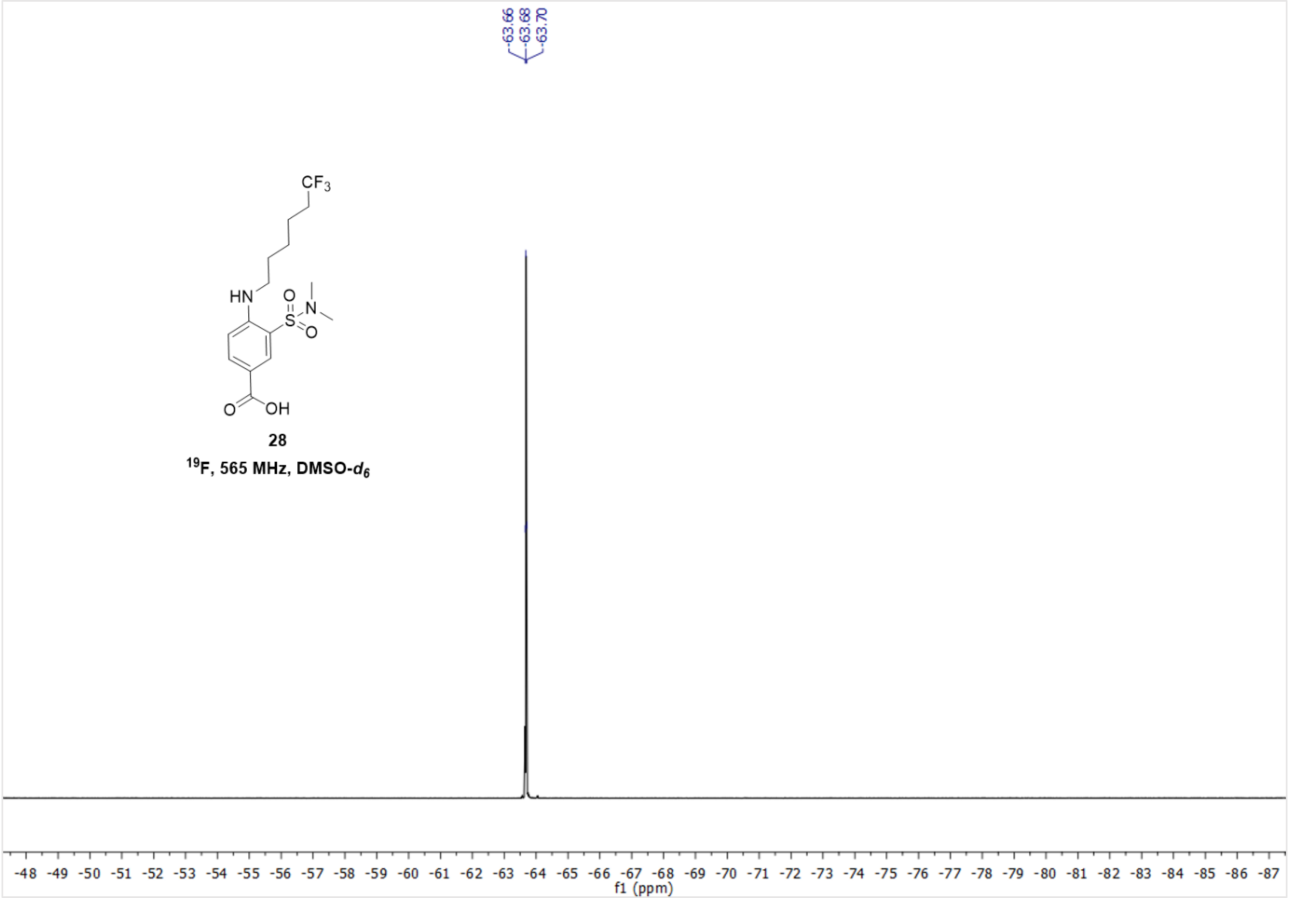
-63.66
-63.68
-63.70
CF3
HN
O
S
N
O
O
OH
28
19F, 565 MHz, DMSO-d6
-48 -49 -50 -51 -52 -53 -54 -55 -56 -57 -58 -59 -60 -61 -62 -63 -64 -65 -66 -67 -68 -69 -70 -71 -72 -73 -74 -75 -76 -77 -78 -79 -80 -81 -82 -83 -84 -85 -86 -87
f1 (ppm)

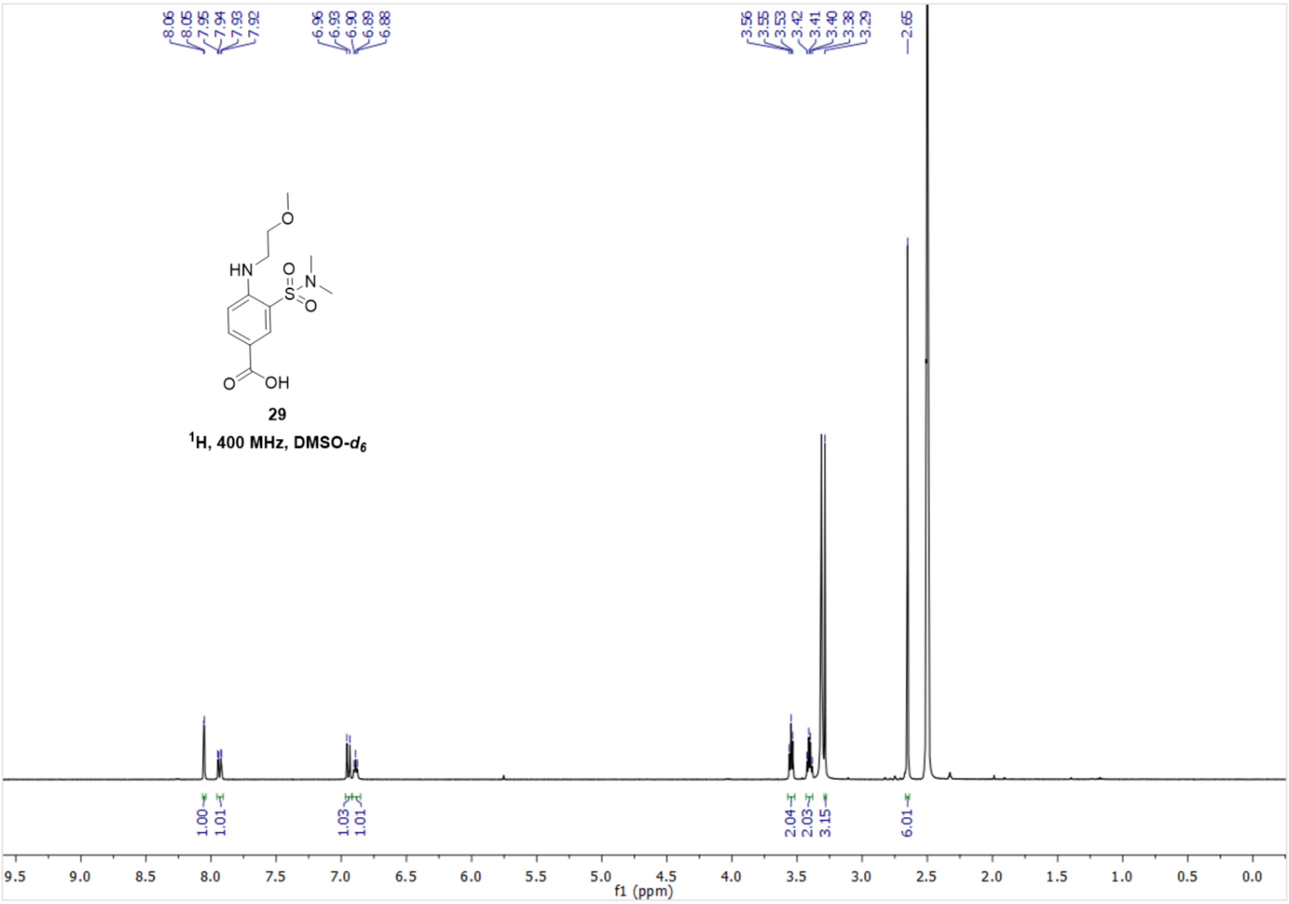
8.06
8.05
7.95
7.94
7.93
7.92
6.96
6.93
6.90
6.89
6.88
3.56
3.55
3.53
3.42
3.41
3.40
3.38
3.29
2.65
HN
O
S
N
O
O
OH
29
1H, 400 MHz, DMSO-d6
1.00
1.01
1.03
1.01
2.04
2.03
3.15
6.01
f1 (ppm)

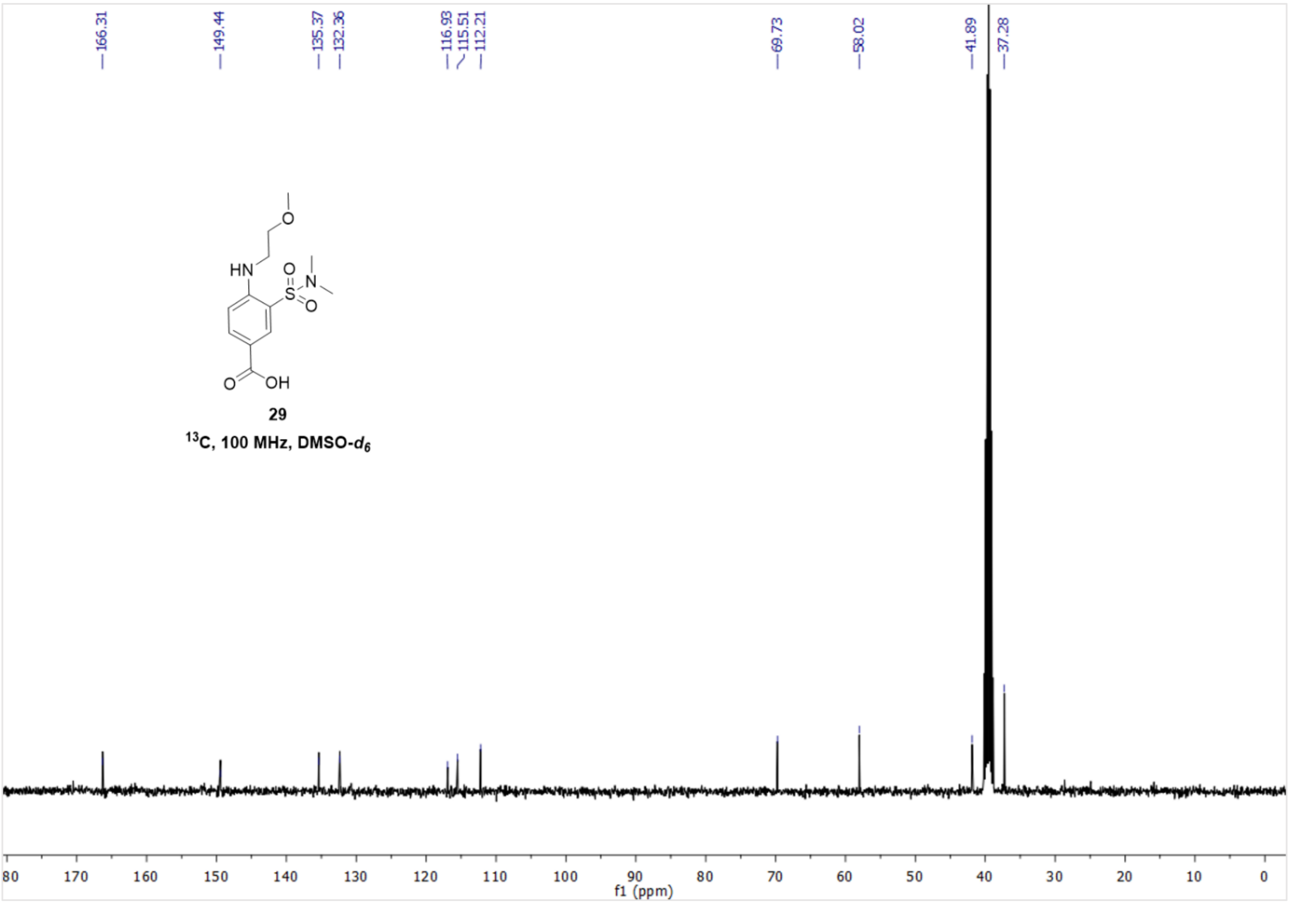
166.31
149.44
135.37
132.36
116.93
115.51
112.21
69.73
58.02
41.89
37.28
HN
O
S
N
O
O
OH
29
13C, 100 MHz, DMSO-d6
f1 (ppm)

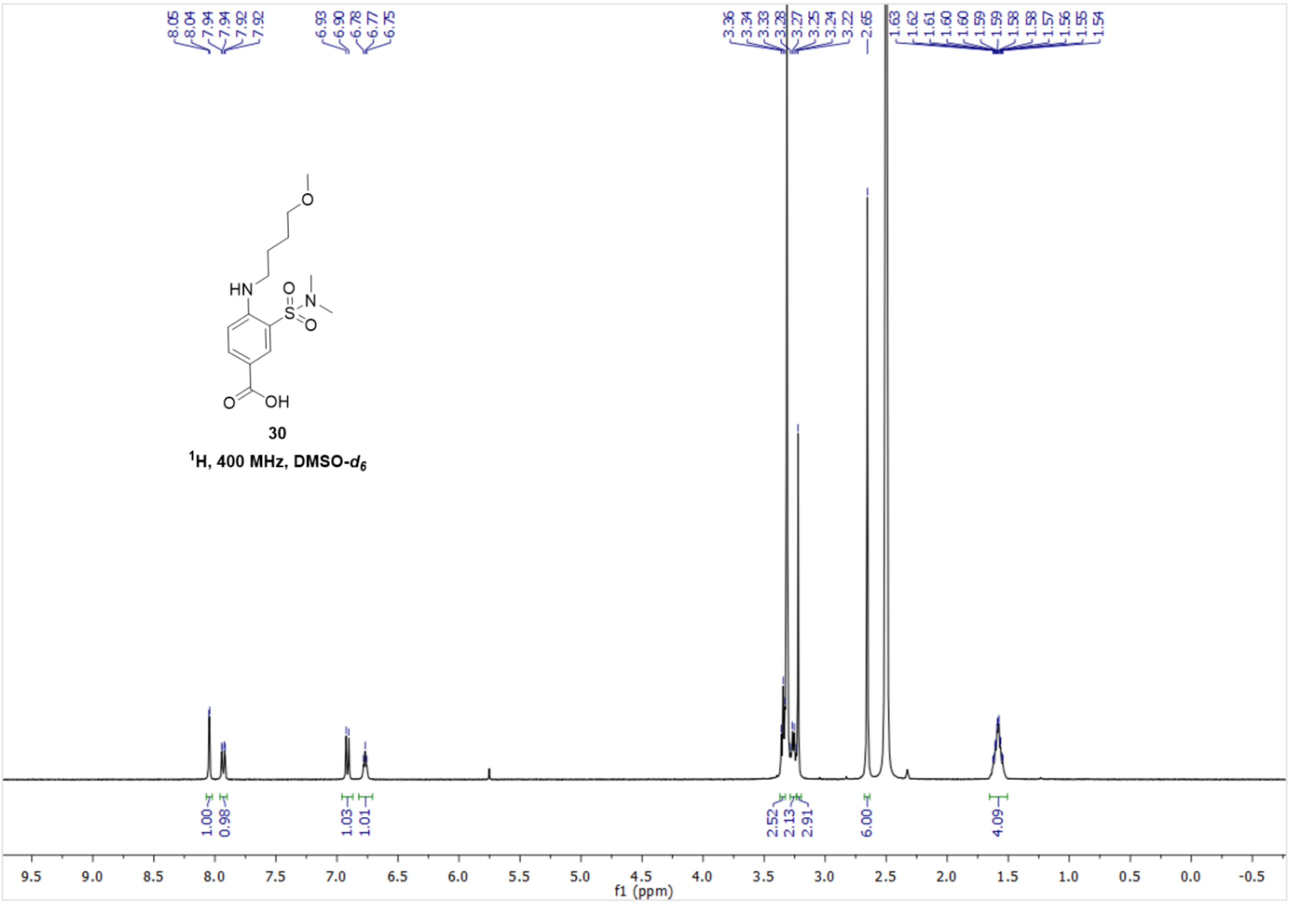
8.05 8.04 7.94 7.94 7.92 7.92
6.93 6.90 6.78 6.77 6.75
3.36 3.34 3.33 3.28 3.27 3.25 3.24 3.22
2.65
1.63 1.62 1.61 1.60 1.60 1.59 1.59 1.58 1.58 1.57 1.56 1.55 1.54
HN
O
S
N
OH
30
¹H, 400 MHz, DMSO-d6
1.00 0.98 1.03 1.01 2.52 2.13 2.91 6.00 4.09
f1 (ppm)

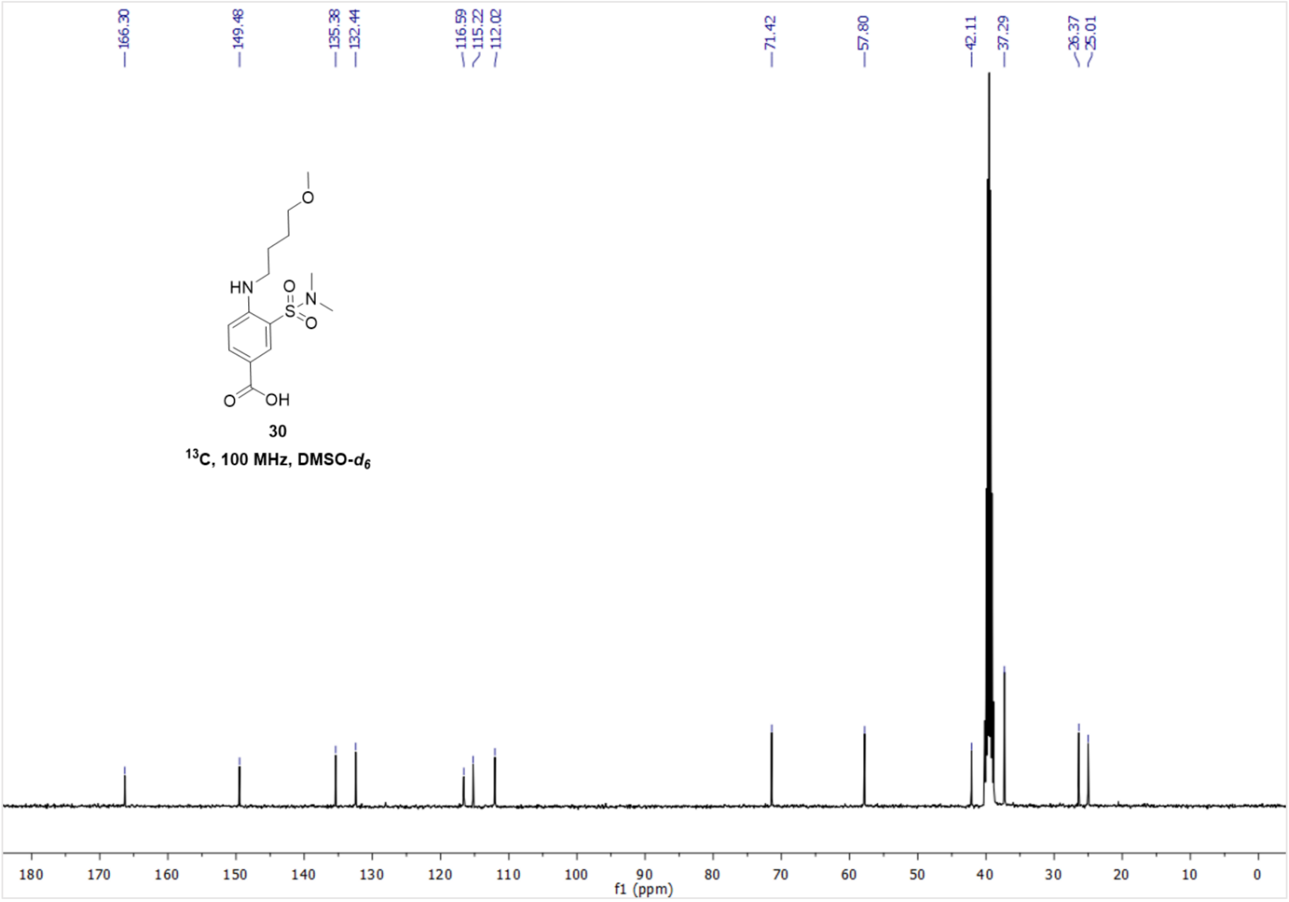
166.30
149.48
135.38 132.44
116.59 115.22 112.02
71.42
57.80
42.11 37.29
26.37 25.01
HN
O
S
N
OH
30
¹³C, 100 MHz, DMSO-d6
f1 (ppm)

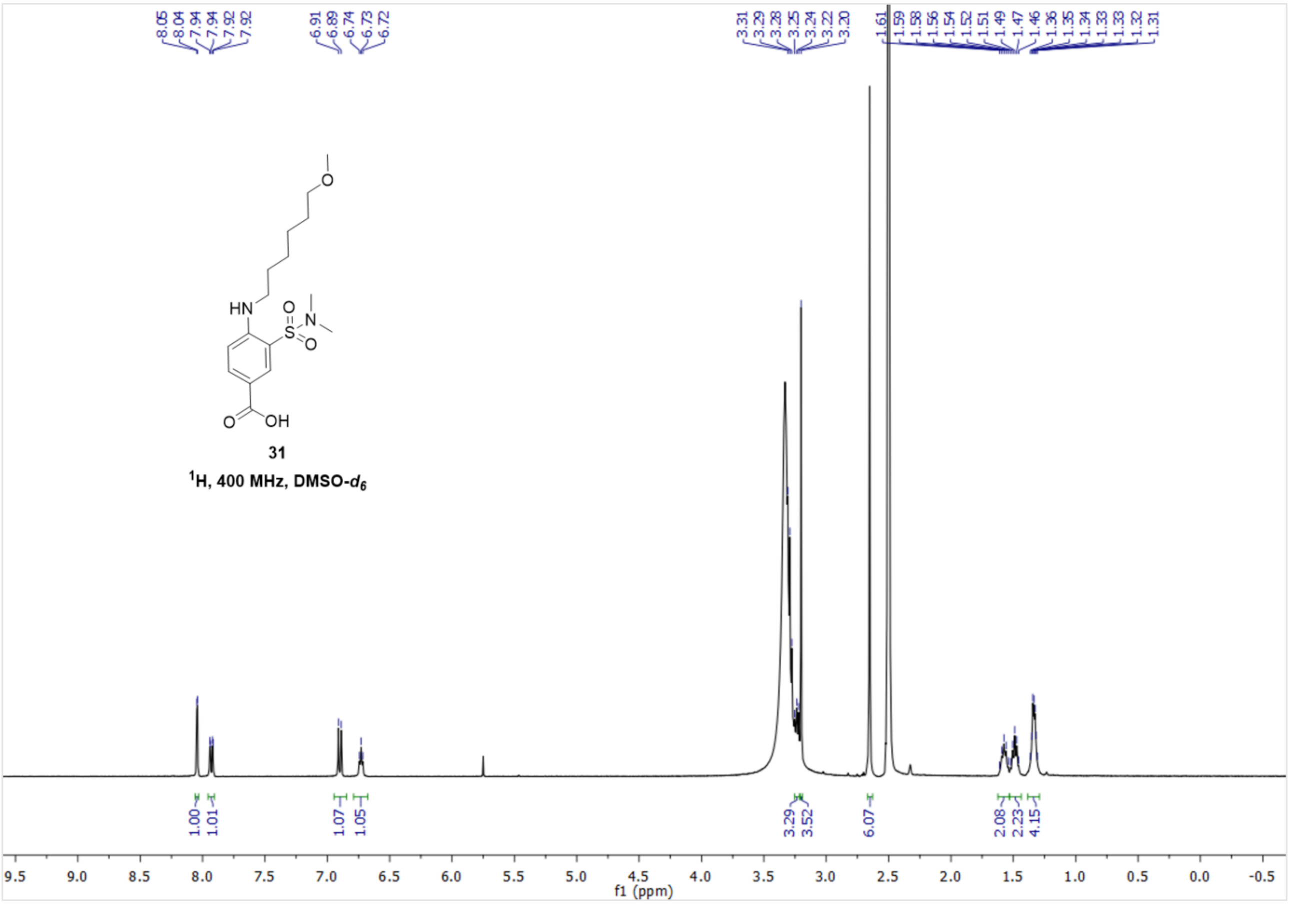
8.05
8.04
7.94
7.94
7.92
7.92
6.91
6.89
6.74
6.73
6.72
3.31
3.29
3.28
3.25
3.24
3.22
3.20
1.61
1.59
1.58
1.56
1.54
1.52
1.51
1.49
1.47
1.46
1.36
1.35
1.34
1.33
1.33
1.32
1.31
HN
O
S
N
O
O
OH
O
31
1H, 400 MHz, DMSO-d6
1.00
1.01
1.07
1.05
3.29
3.52
6.07
2.08
2.23
4.15
f1 (ppm)

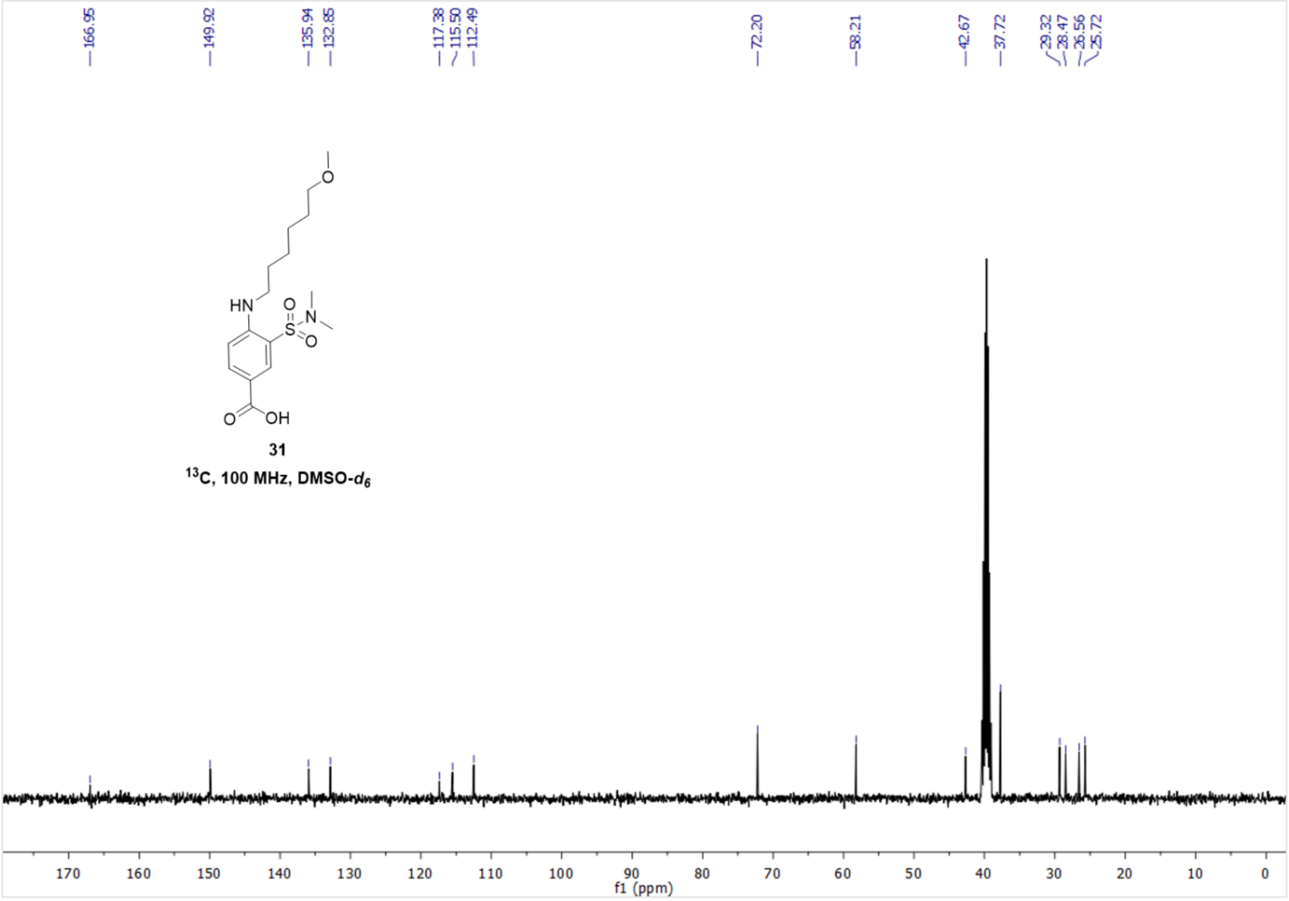
166.95
149.92
135.94
132.85
117.38
115.50
112.49
72.20
58.21
42.67
37.72
29.32
28.47
26.56
25.72
HN
O
S
N
O
O
OH
O
31
13C, 100 MHz, DMSO-d6
f1 (ppm)

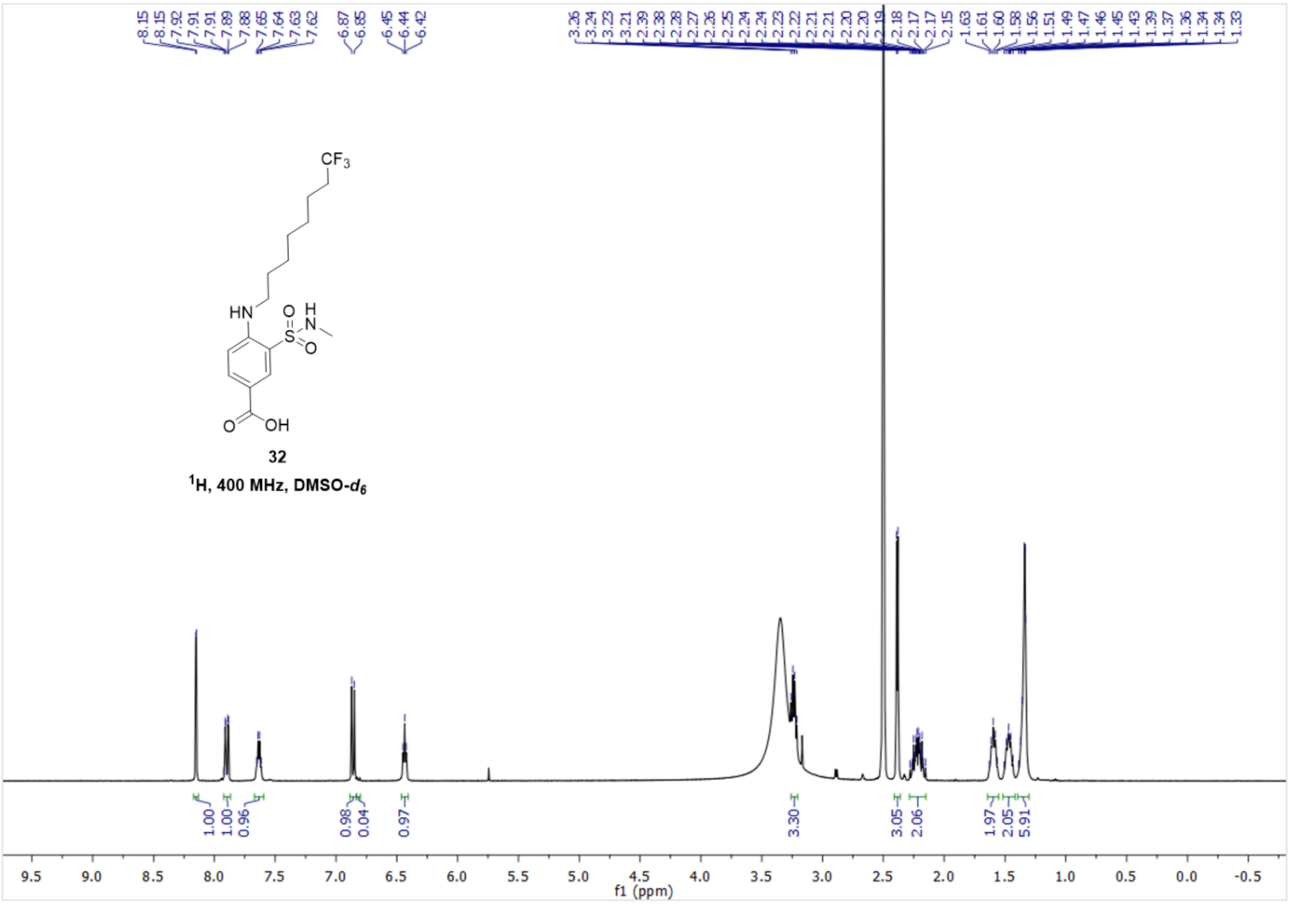

32
^{1}H, 400 MHz, DMSO-d_6
f1 (ppm)

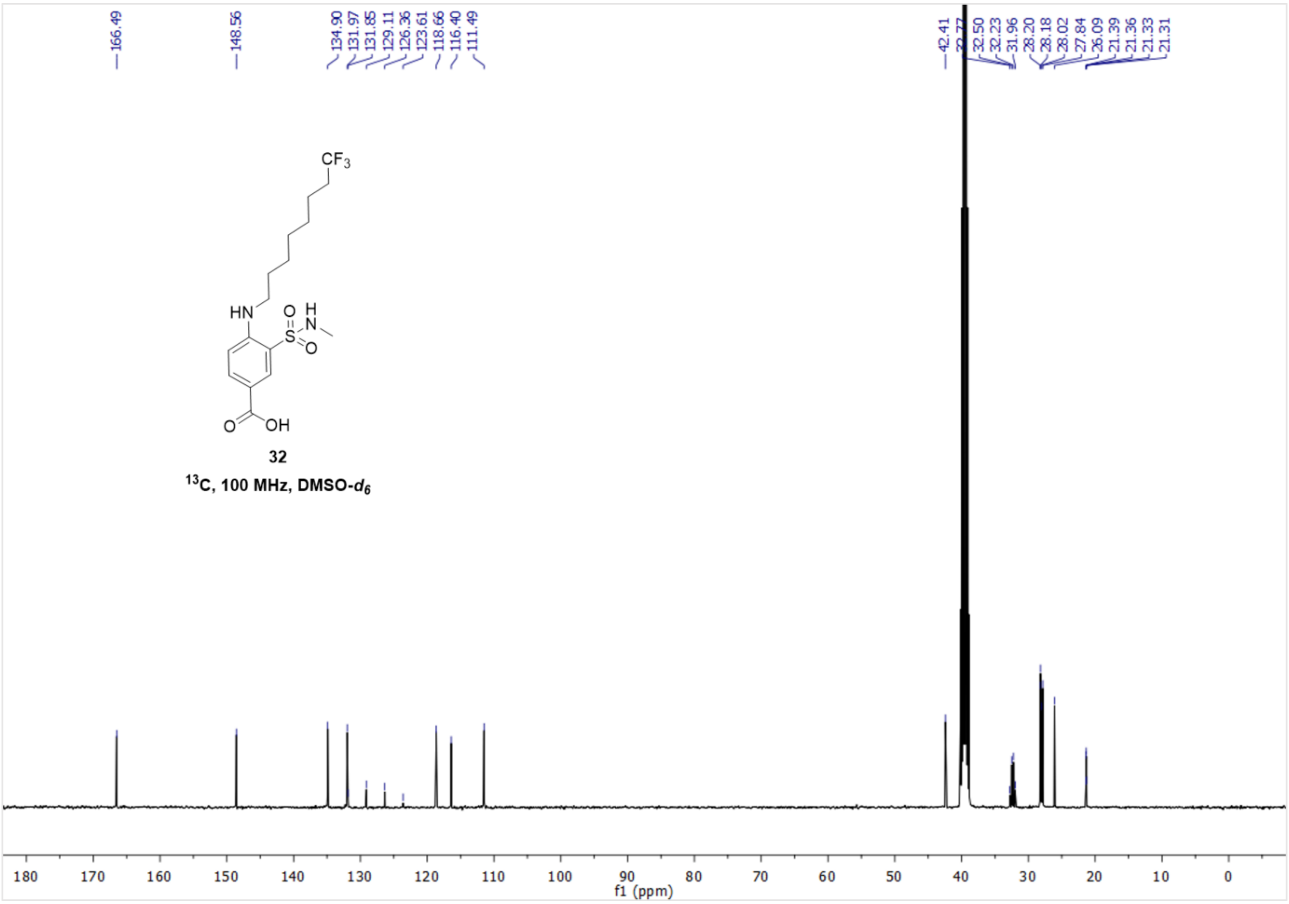

32
^{13}C, 100 MHz, DMSO-d_6
f1 (ppm)

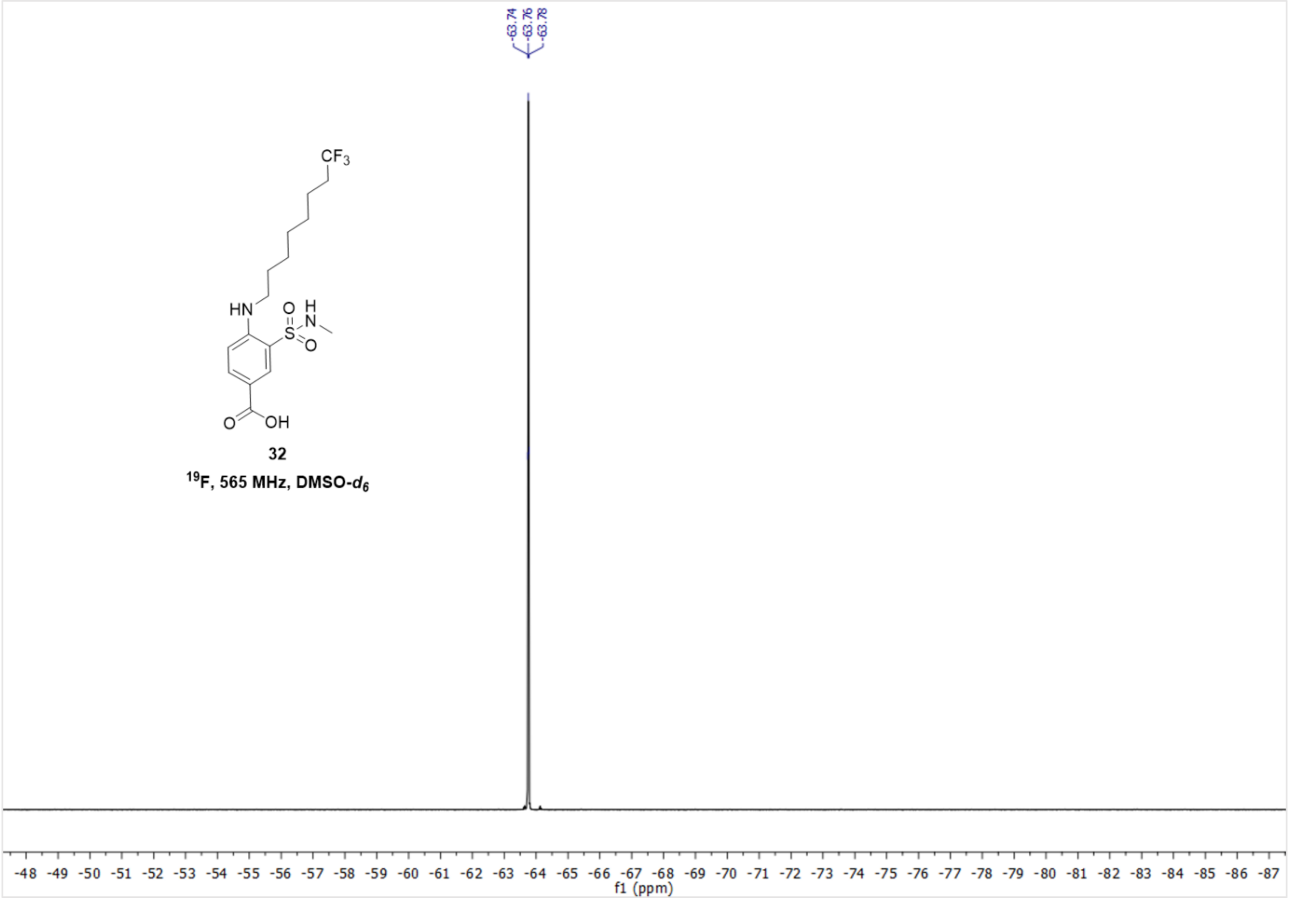

32
^{19}F, 565 MHz, DMSO-d_6
f1 (ppm)

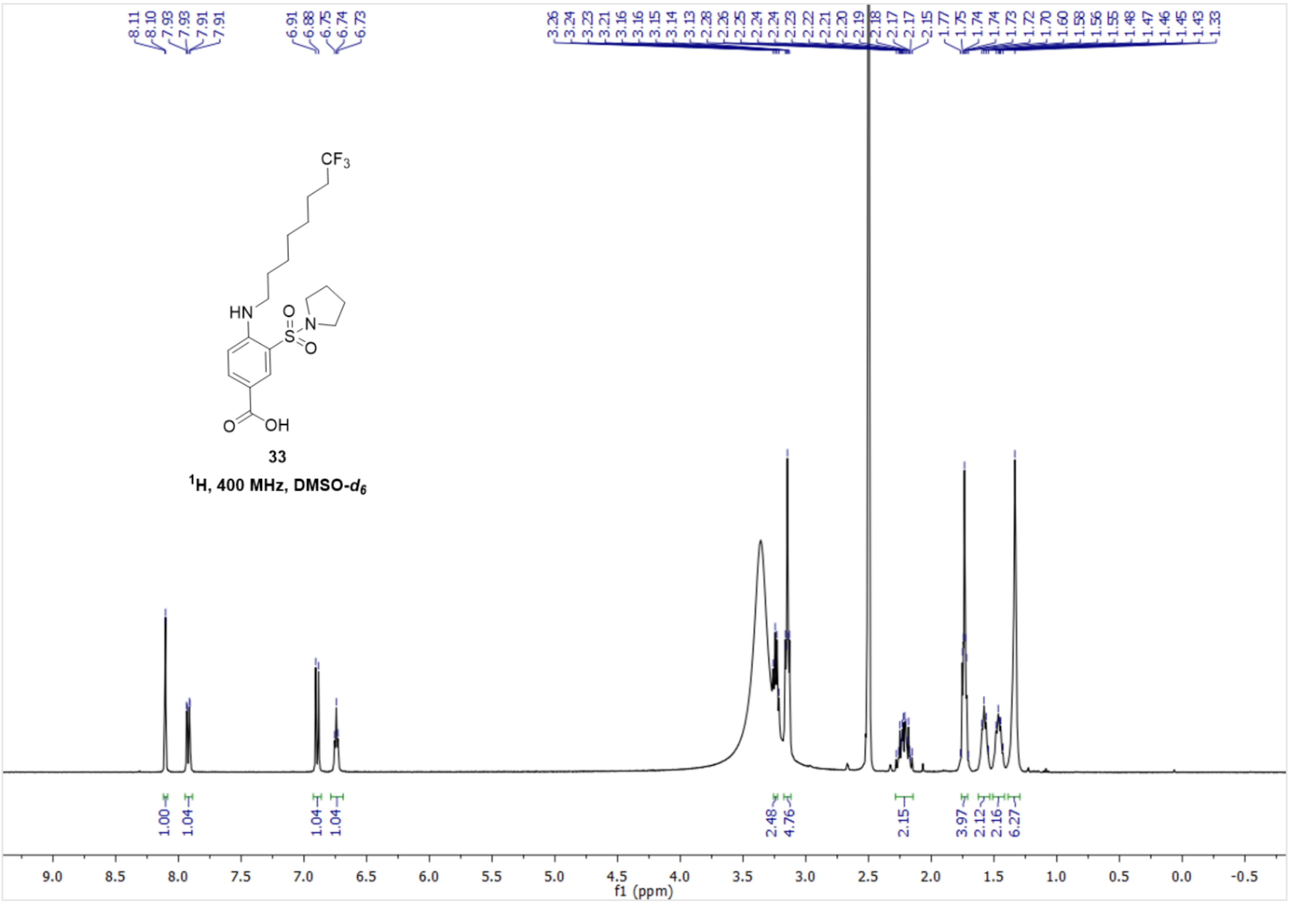

33
^{1}H, 400 MHz, DMSO-d_6
f1 (ppm)

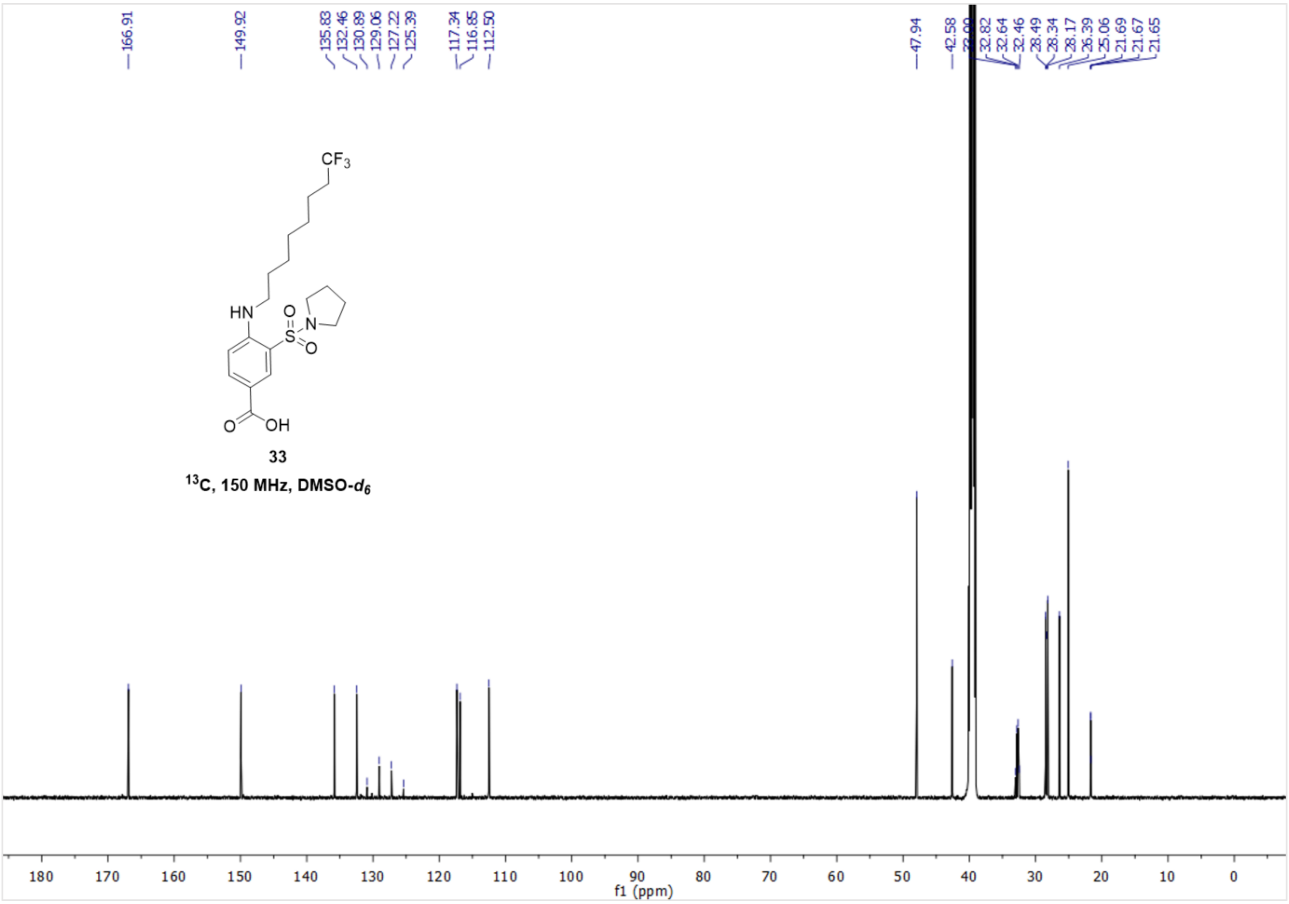

166.91
149.92
135.83
132.46
130.89
129.06
127.22
125.39
117.34
116.85
112.50
47.94
42.58
32.82
32.64
32.46
28.49
28.34
28.17
26.39
25.06
21.69
21.67
21.65
CF3
HN
O
S
N
O
O
OH
33
13C, 150 MHz, DMSO-d6
f1 (ppm)

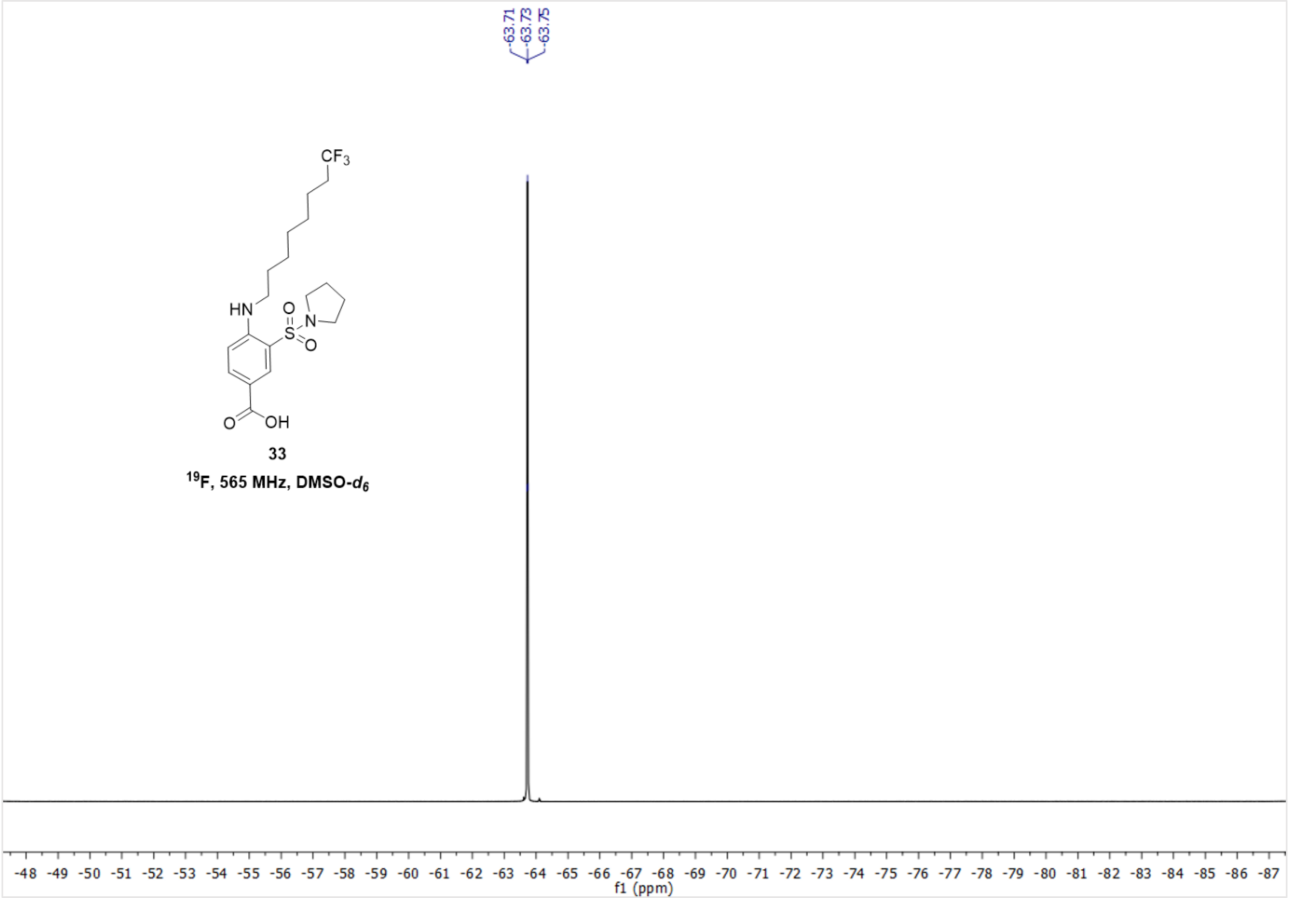

-63.71
-63.73
-63.75
CF3
HN
O
S
N
O
O
OH
33
19F, 565 MHz, DMSO-d6
f1 (ppm)

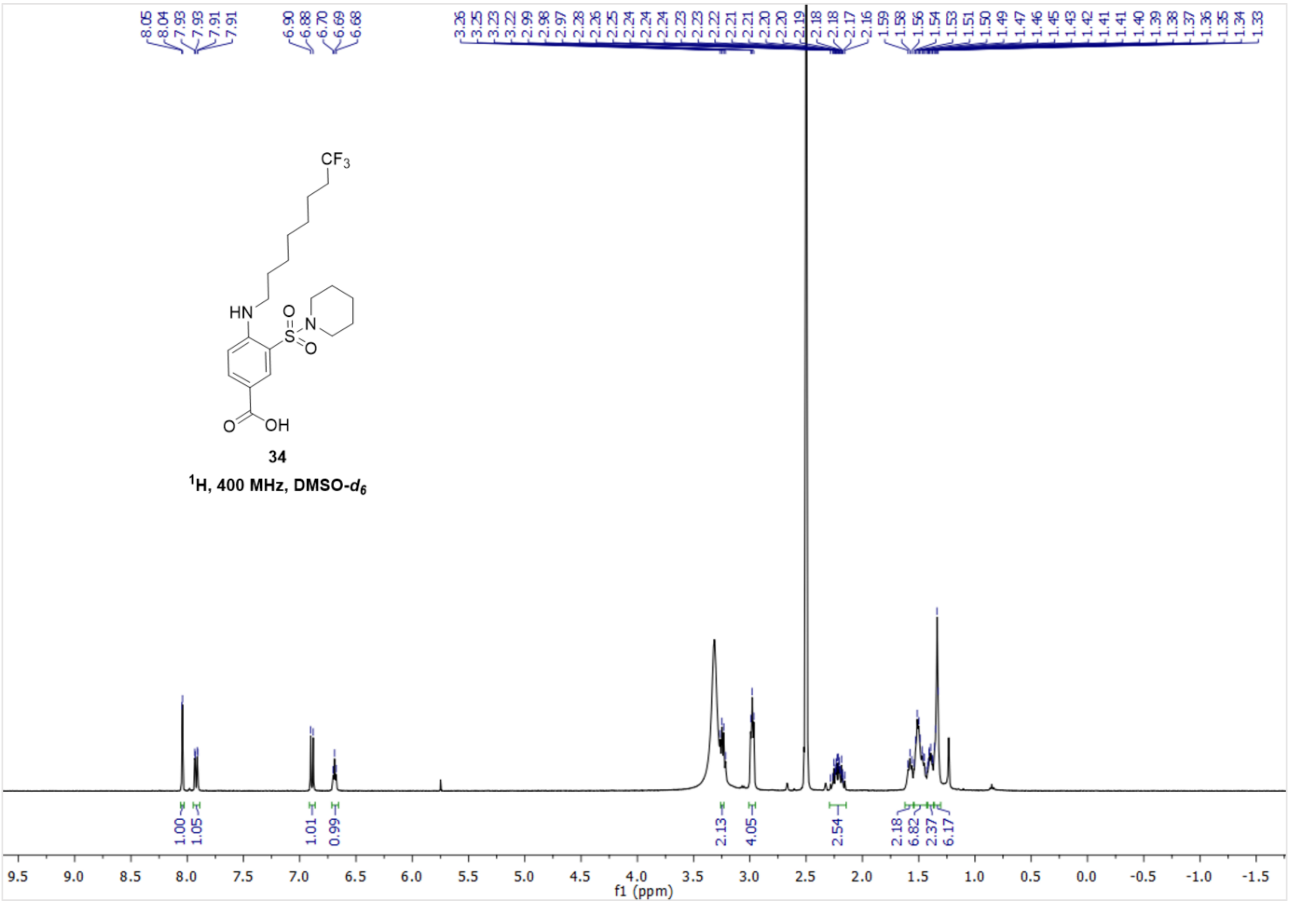
34
^{1}H, 400 MHz, DMSO-d_6
f1 (ppm)

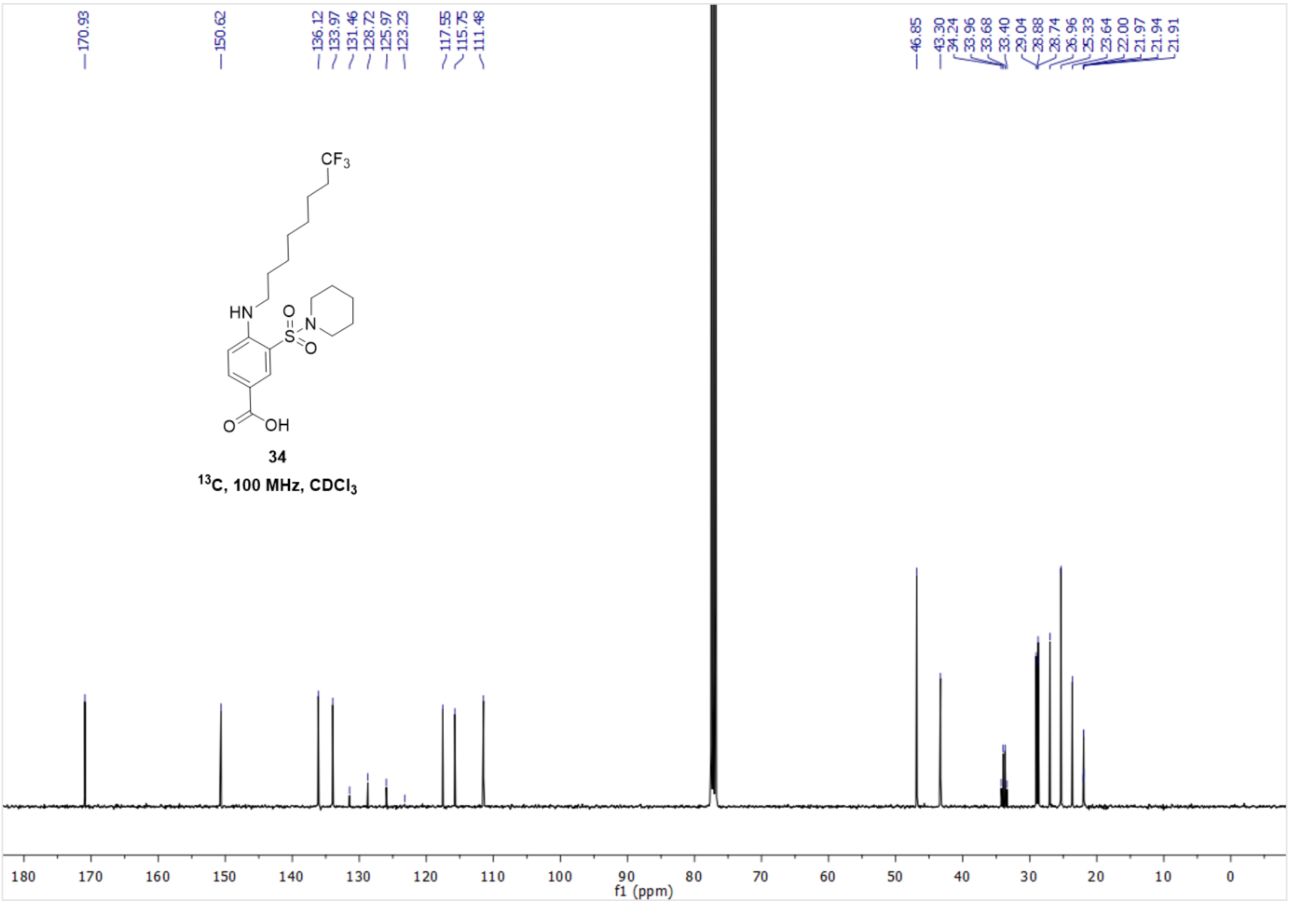
34
^{13}C, 100 MHz, CDCl$_3$
f1 (ppm)

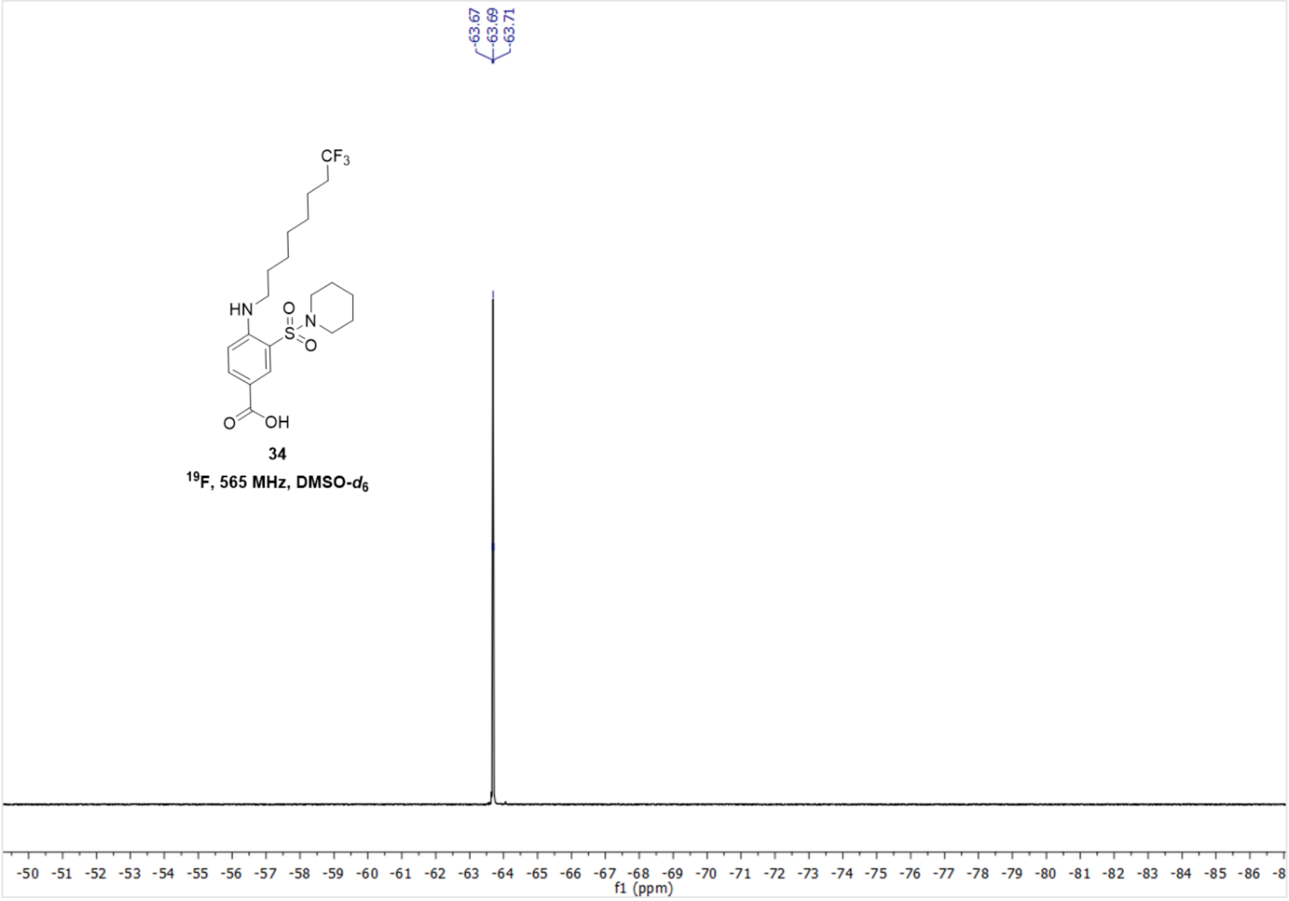
-63.67
-63.69
-63.71
CF3
HN
O
S
N
O
O
OH
34
19F, 565 MHz, DMSO-d6
f1 (ppm)

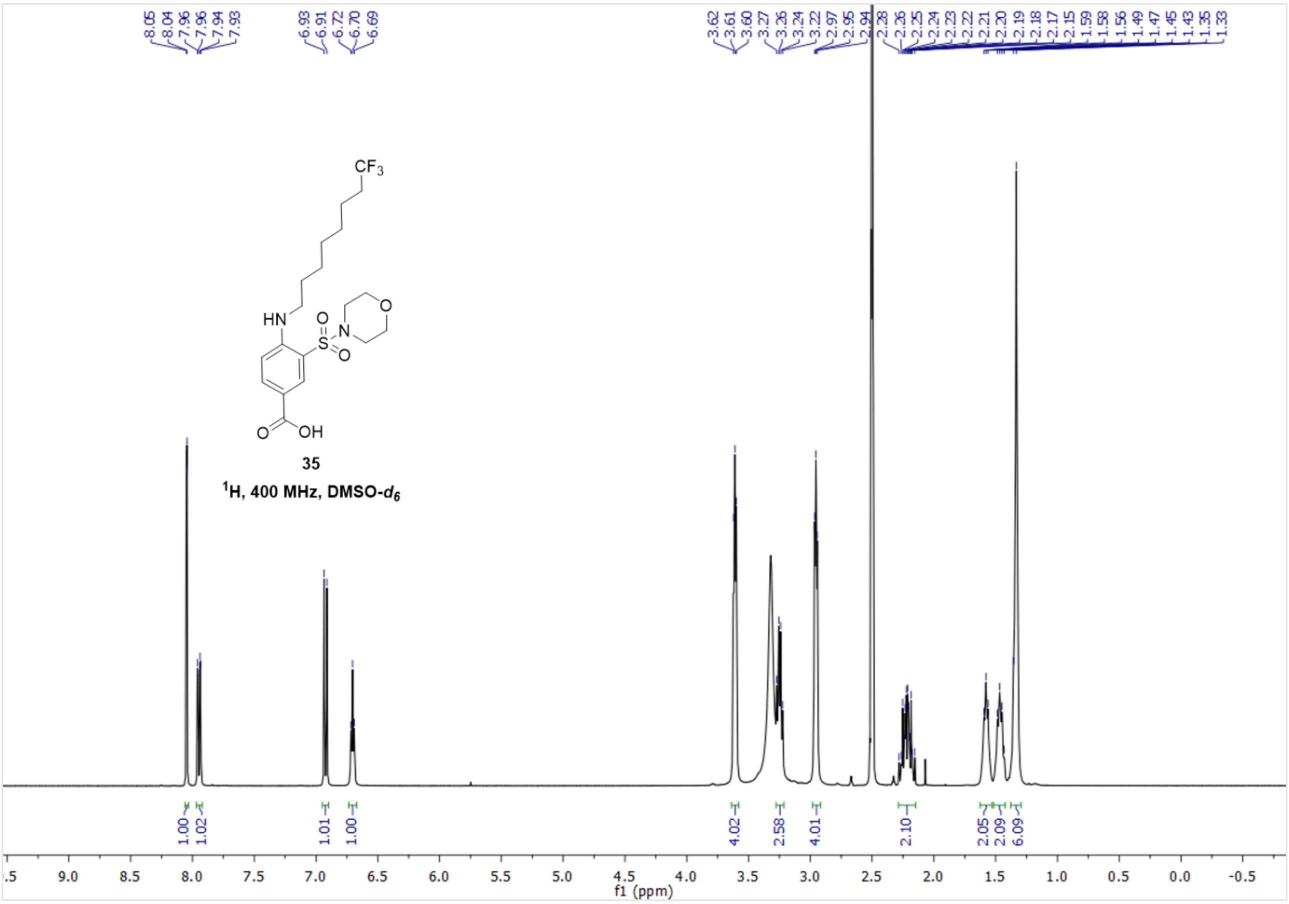
8.05
8.04
7.96
7.96
7.94
7.93
6.93
6.91
6.72
6.70
6.69
3.62
3.61
3.60
3.27
3.26
3.24
3.22
2.97
2.95
2.94
2.28
2.26
2.25
2.24
2.23
2.22
2.21
2.20
2.19
2.18
2.17
2.15
1.59
1.58
1.56
1.49
1.47
1.45
1.43
1.35
1.33
CF3
HN
O
S
N
O
O
O
OH
35
1H, 400 MHz, DMSO-d6
1.00
1.02
1.01
1.00
4.02
2.58
4.01
2.10
2.05
2.09
6.09
f1 (ppm)

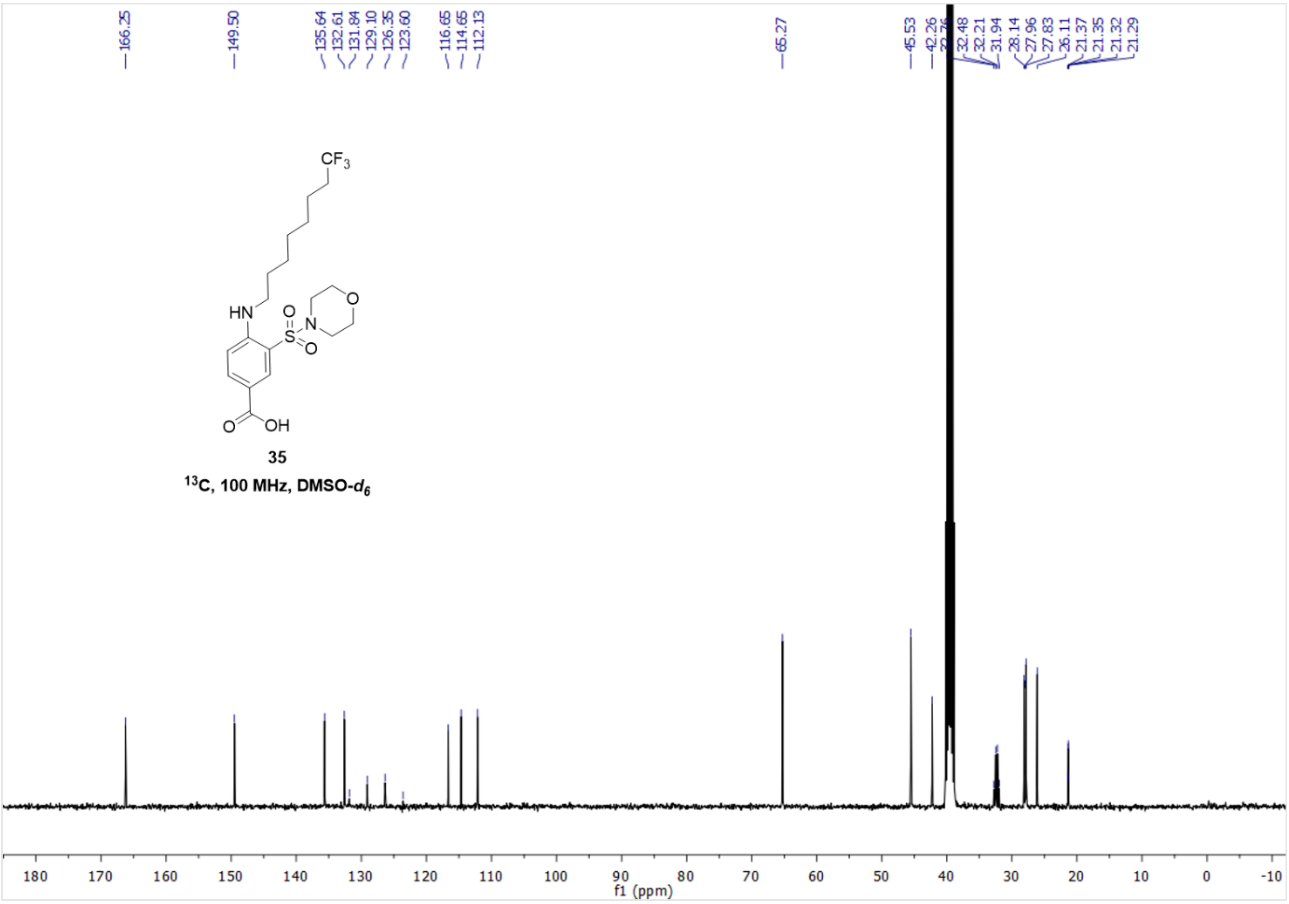

35
^{13}C, 100 MHz, DMSO-d_6

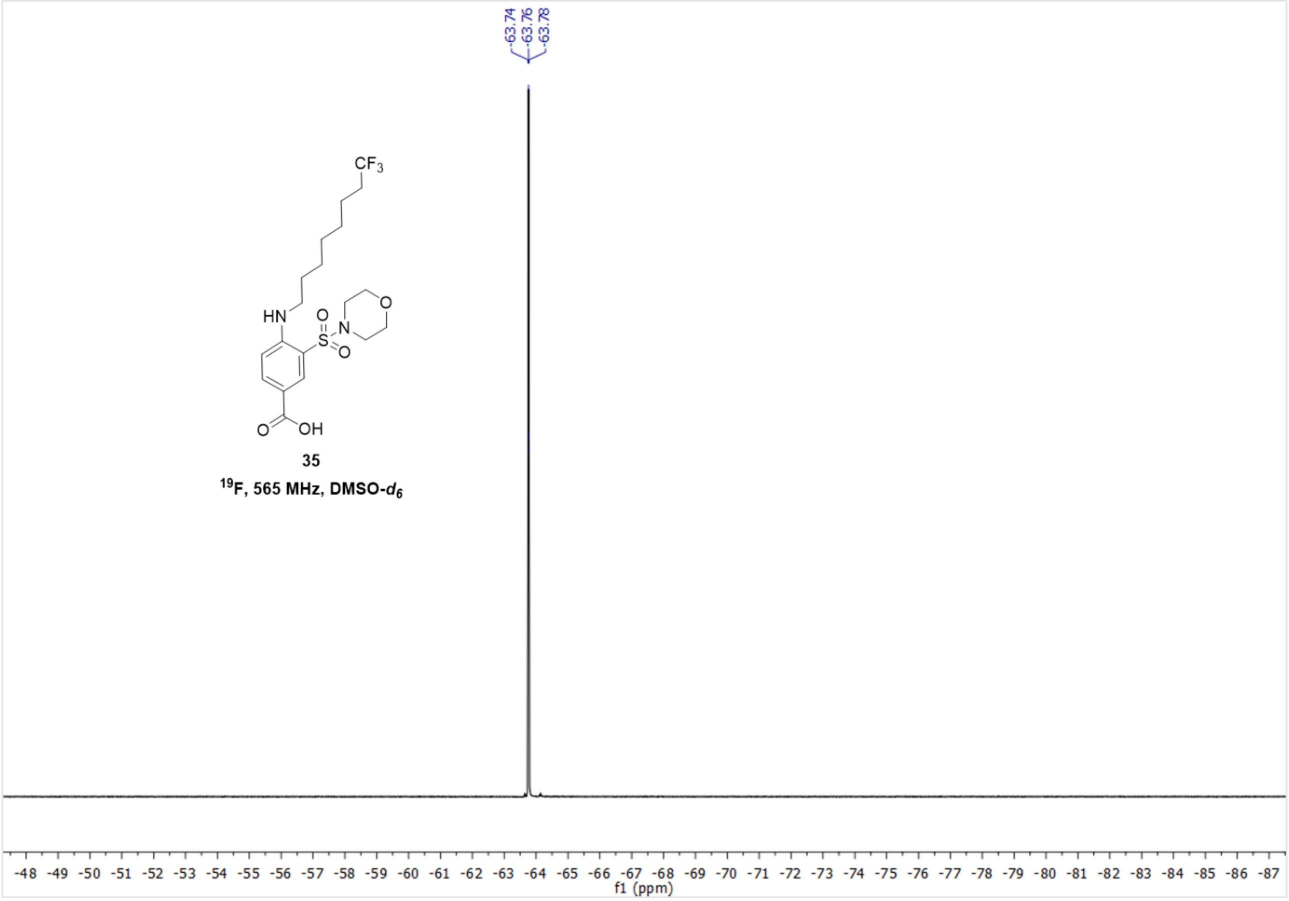

35
^{19}F, 565 MHz, DMSO-d_6

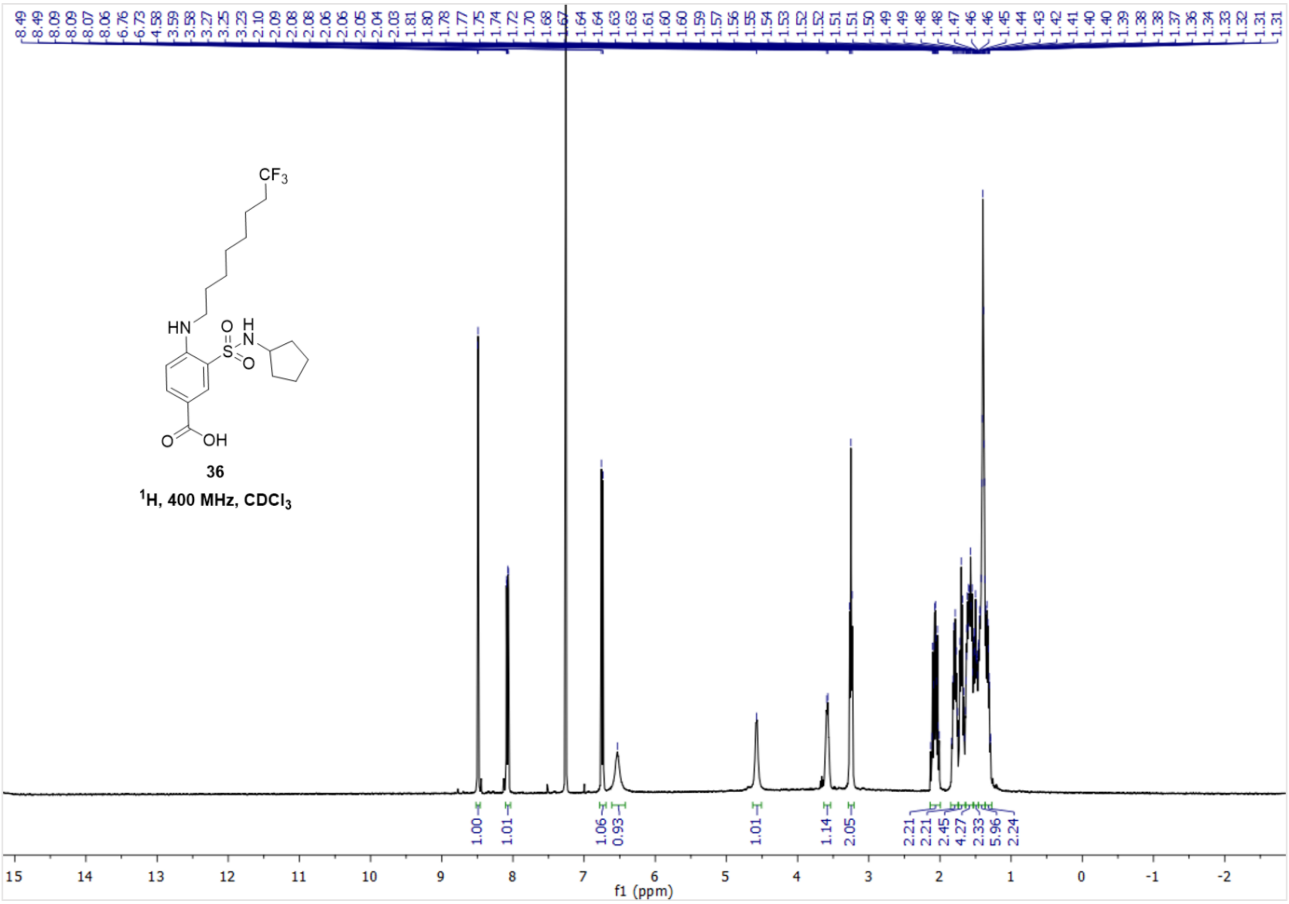

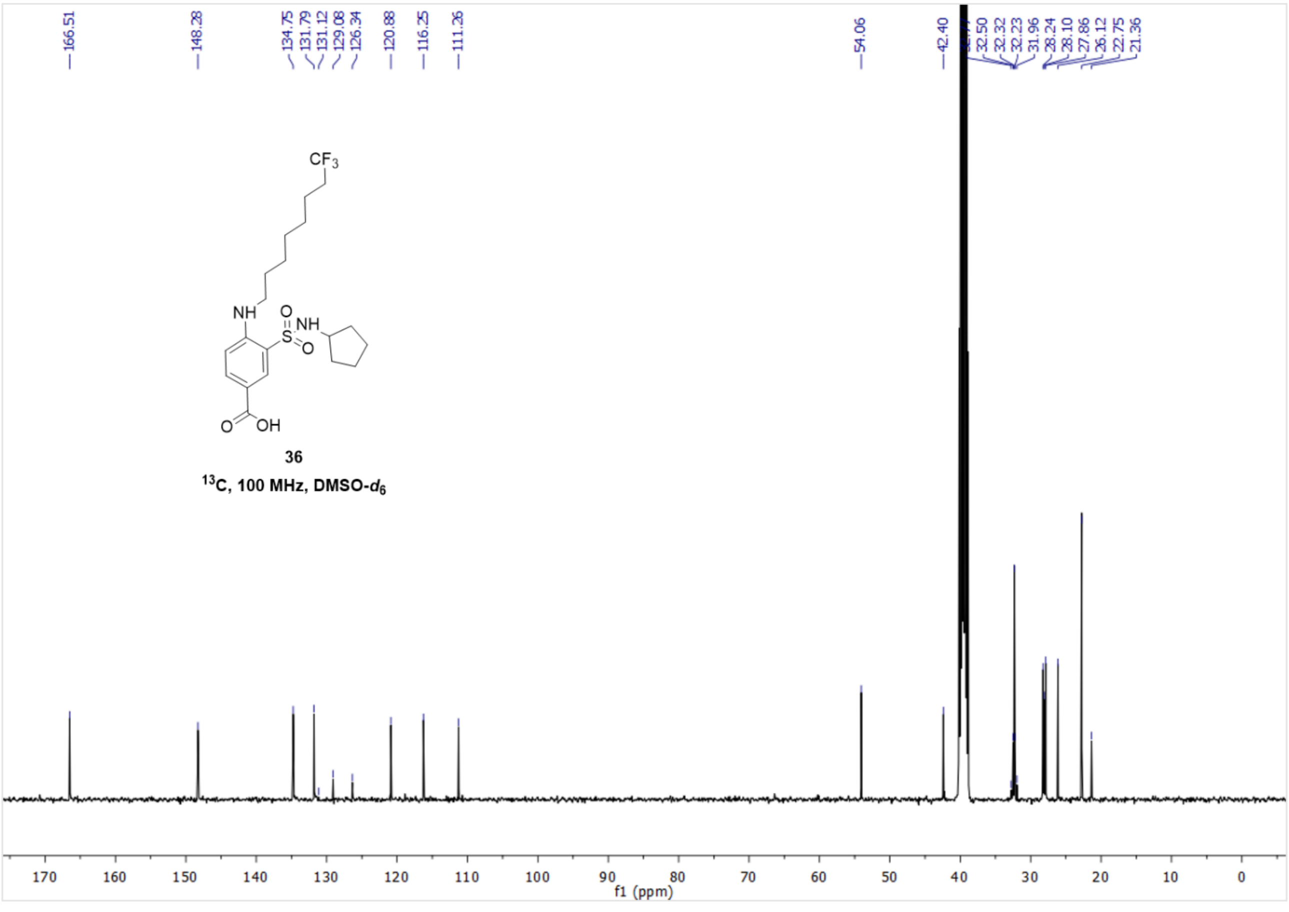

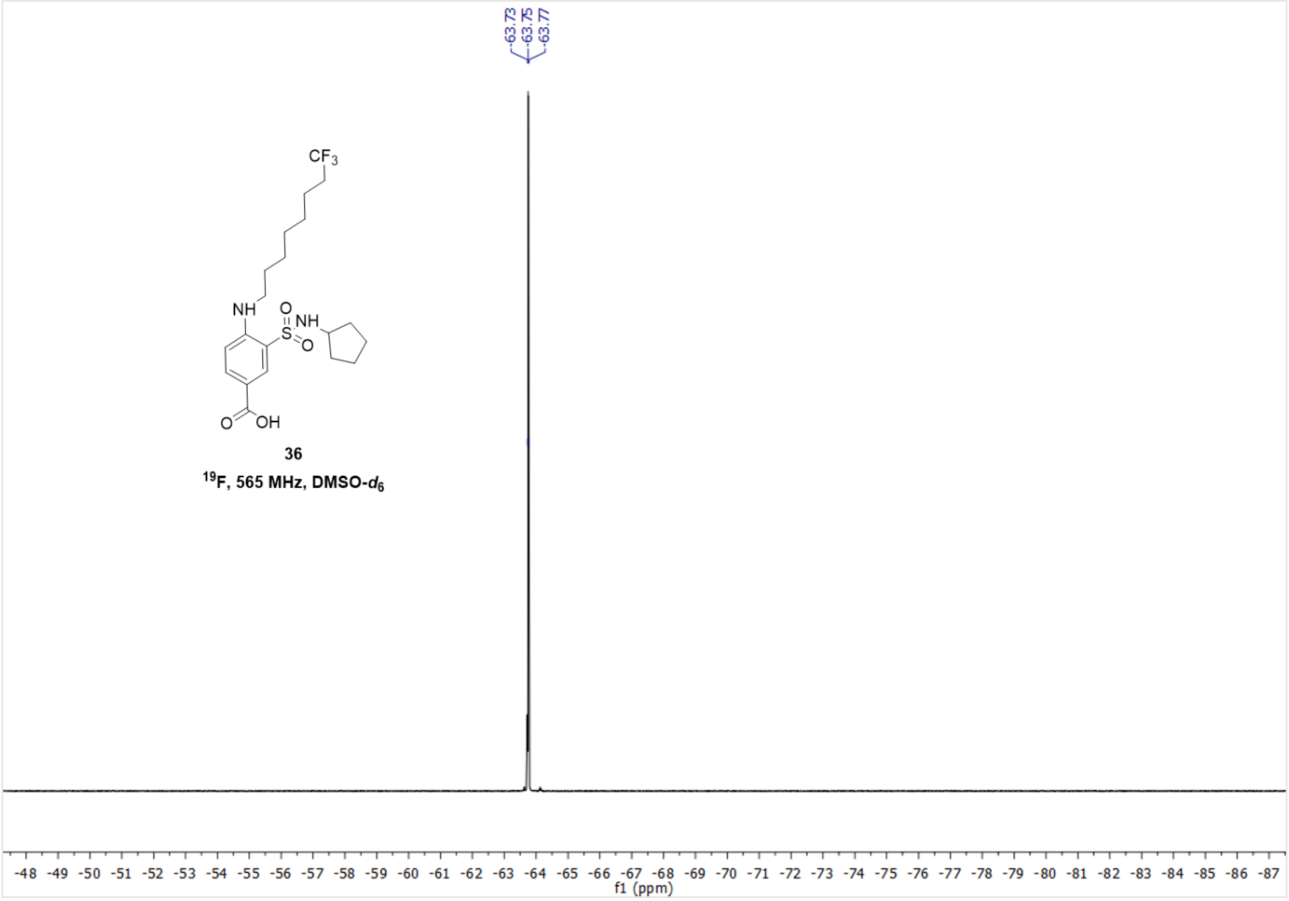
-63.73
-63.75
-63.77
CF3
NH
O
S
NH
O
O
OH
36
19F, 565 MHz, DMSO-d6
-48 -49 -50 -51 -52 -53 -54 -55 -56 -57 -58 -59 -60 -61 -62 -63 -64 -65 -66 -67 -68 -69 -70 -71 -72 -73 -74 -75 -76 -77 -78 -79 -80 -81 -82 -83 -84 -85 -86 -87
f1 (ppm)

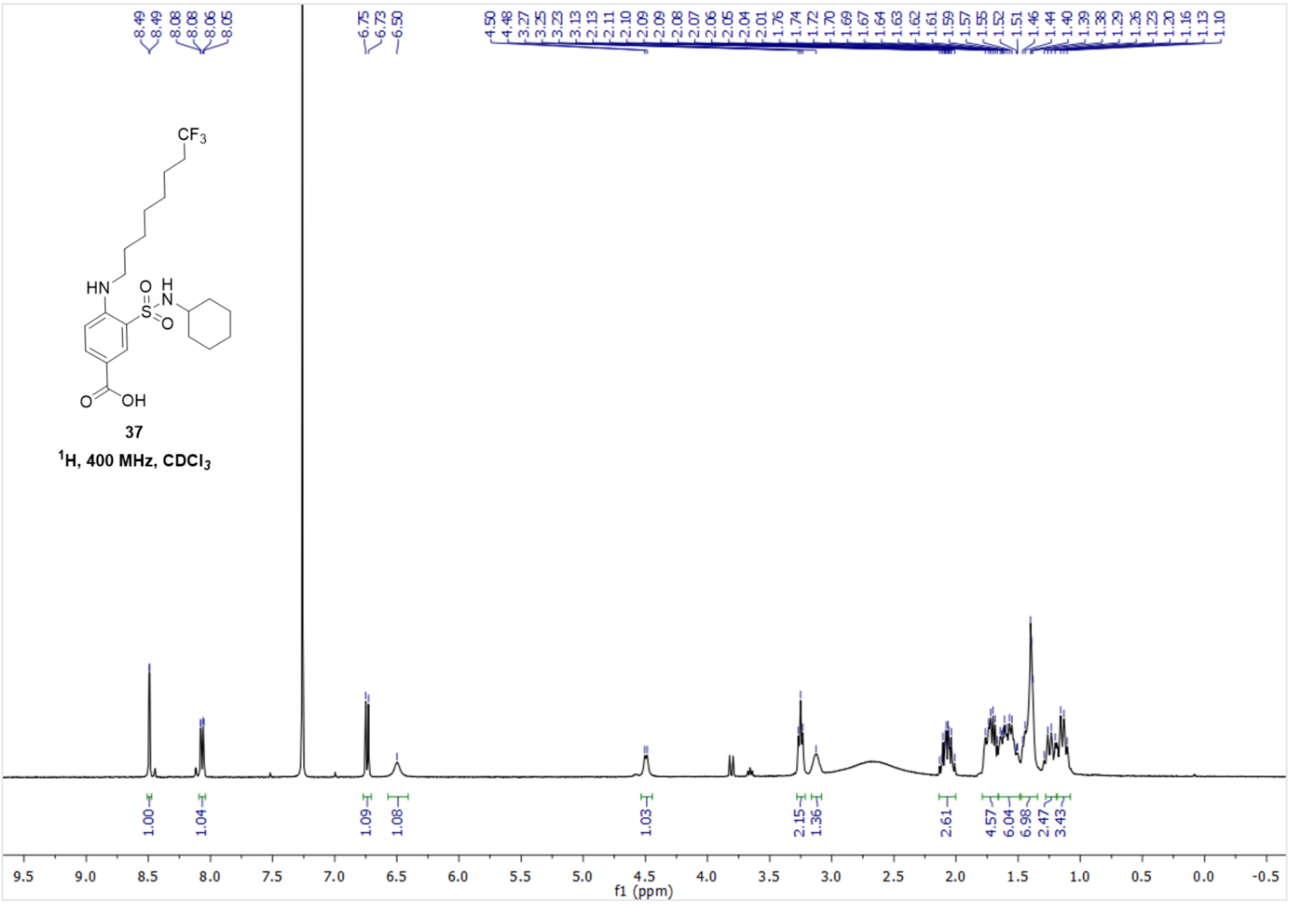
8.49
8.49
8.08
8.08
8.06
8.05
6.75
6.73
6.50
4.50
4.48
3.27
3.25
3.23
3.13
2.13
2.11
2.10
2.09
2.09
2.08
2.07
2.06
2.05
2.04
2.01
1.76
1.74
1.72
1.70
1.69
1.67
1.64
1.63
1.62
1.61
1.59
1.57
1.55
1.52
1.51
1.46
1.44
1.40
1.39
1.38
1.29
1.26
1.23
1.20
1.16
1.13
1.10
CF3
HN
O
S
H
N
O
O
OH
37
1H, 400 MHz, CDCl3
1.00
1.04
1.09
1.08
1.03
2.15
1.36
2.61
4.57
6.04
6.98
2.47
3.43
9.5 9.0 8.5 8.0 7.5 7.0 6.5 6.0 5.5 5.0 4.5 4.0 3.5 3.0 2.5 2.0 1.5 1.0 0.5 0.0 -0.5
f1 (ppm)

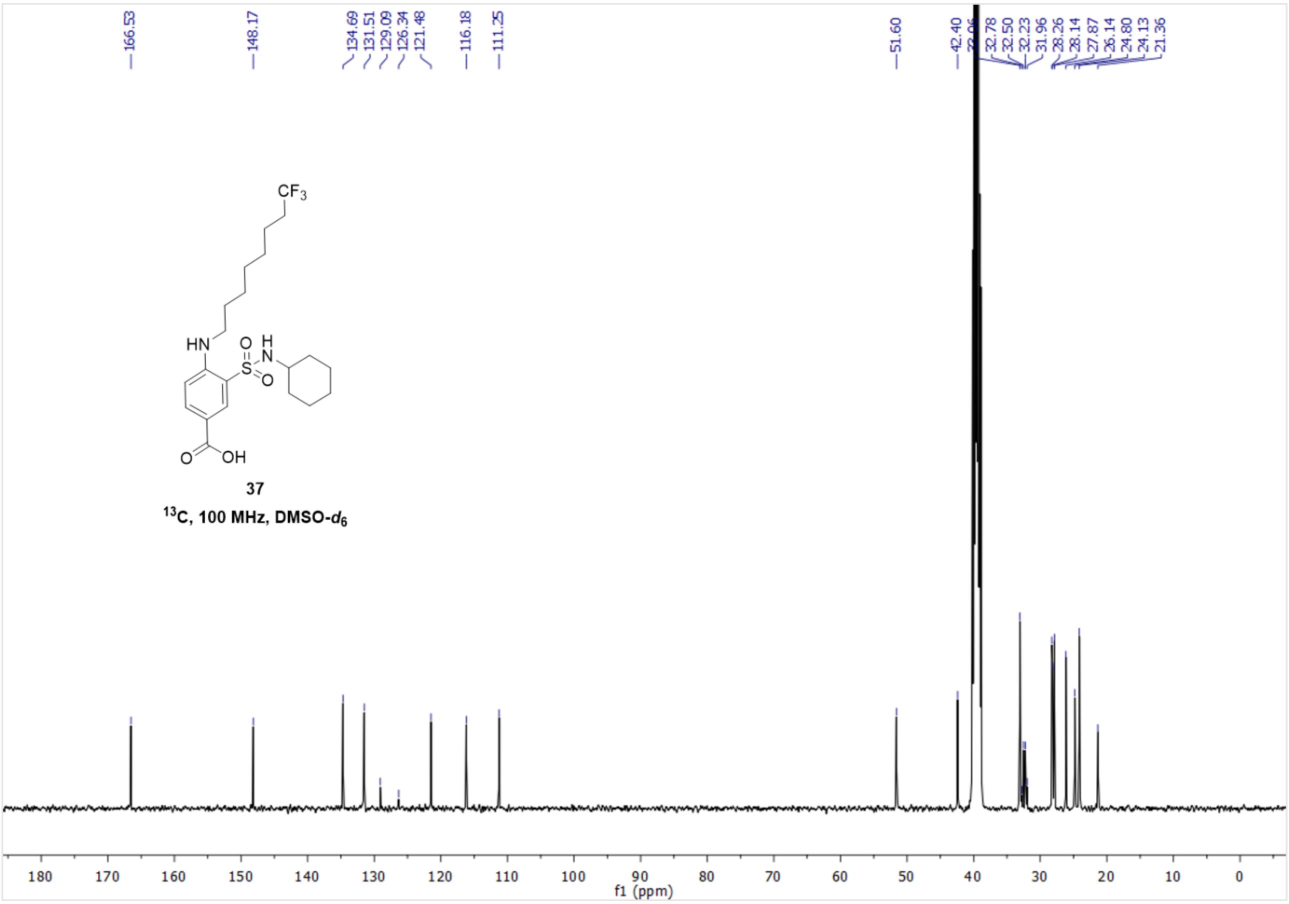

166.53
148.17
134.69
131.51
129.09
126.34
121.48
116.18
111.25
51.60
42.40
32.78
32.50
32.23
31.96
28.26
28.14
27.87
26.14
24.80
24.13
21.36
CF3
HN
O
H
S
N
O
O
OH
37
13C, 100 MHz, DMSO-d6
180
170
160
150
140
130
120
110
100
90
80
70
60
50
40
30
20
10
0
f1 (ppm)

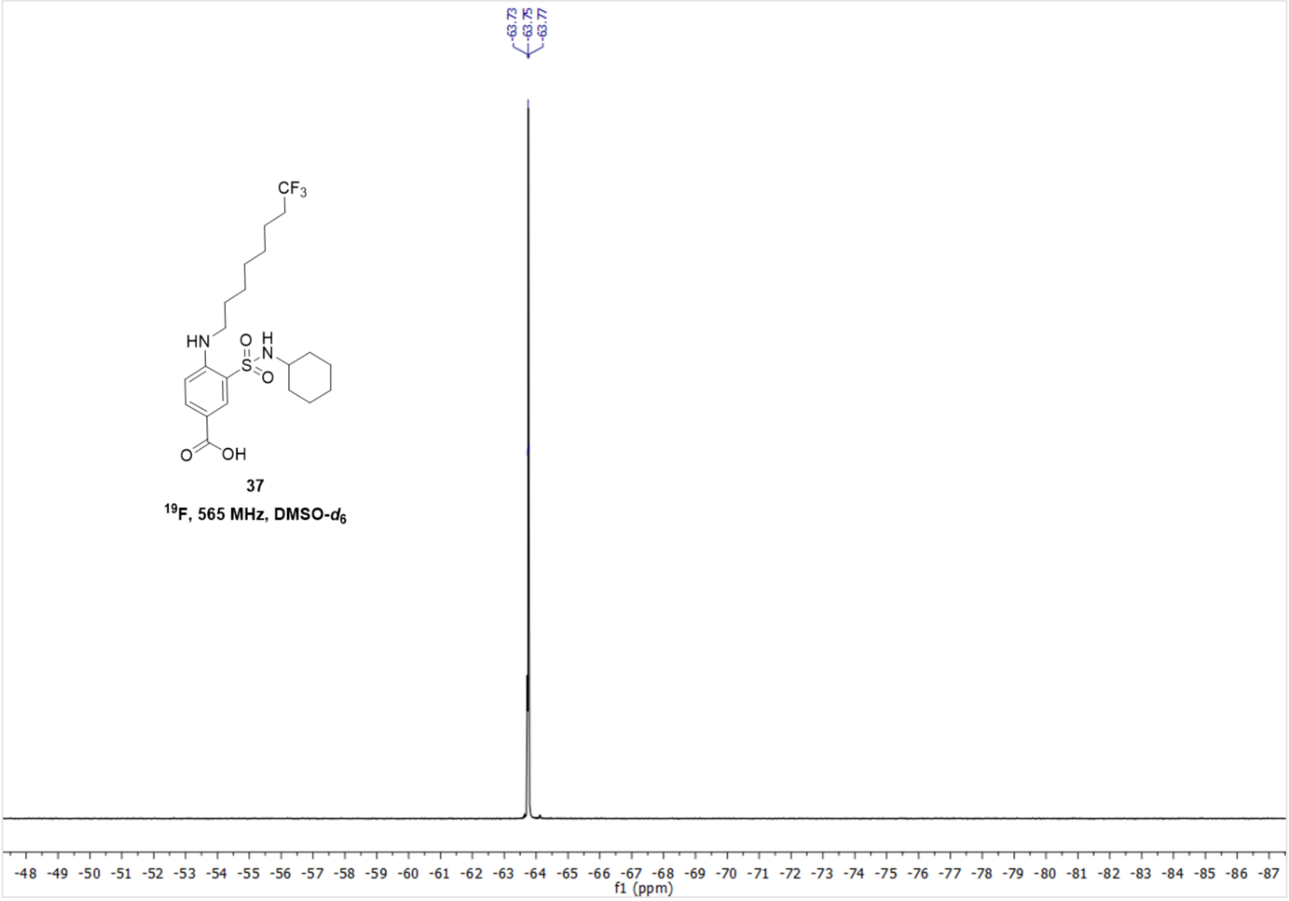

-63.73
-63.75
-63.77
CF3
HN
O
H
S
N
O
O
OH
37
19F, 565 MHz, DMSO-d6
-48 -49 -50 -51 -52 -53 -54 -55 -56 -57 -58 -59 -60 -61 -62 -63 -64 -65 -66 -67 -68 -69 -70 -71 -72 -73 -74 -75 -76 -77 -78 -79 -80 -81 -82 -83 -84 -85 -86 -87
f1 (ppm)

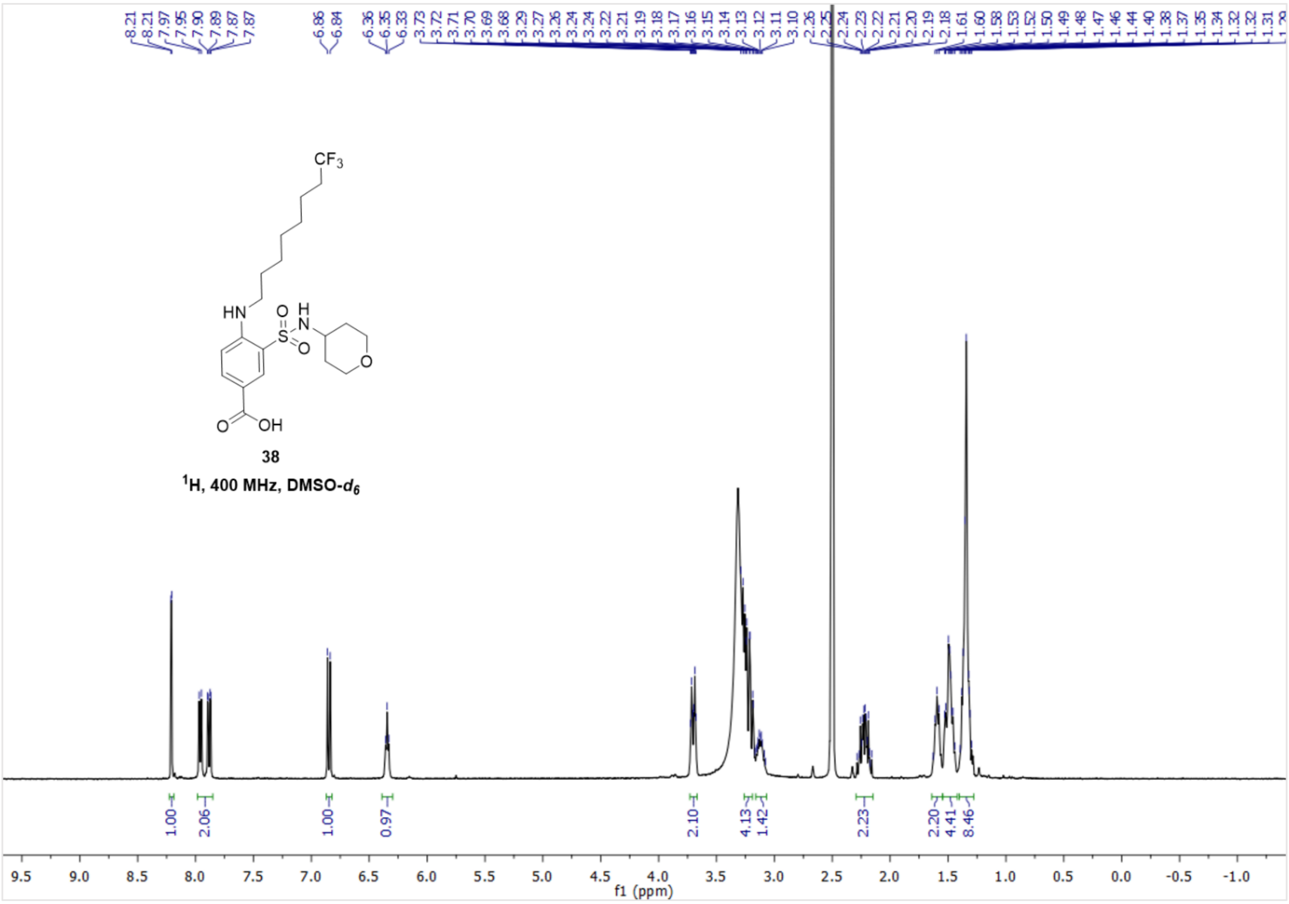
CF3
HN
O
S
O
H
N
O
O
OH
38
1H, 400 MHz, DMSO-d6
f1 (ppm)

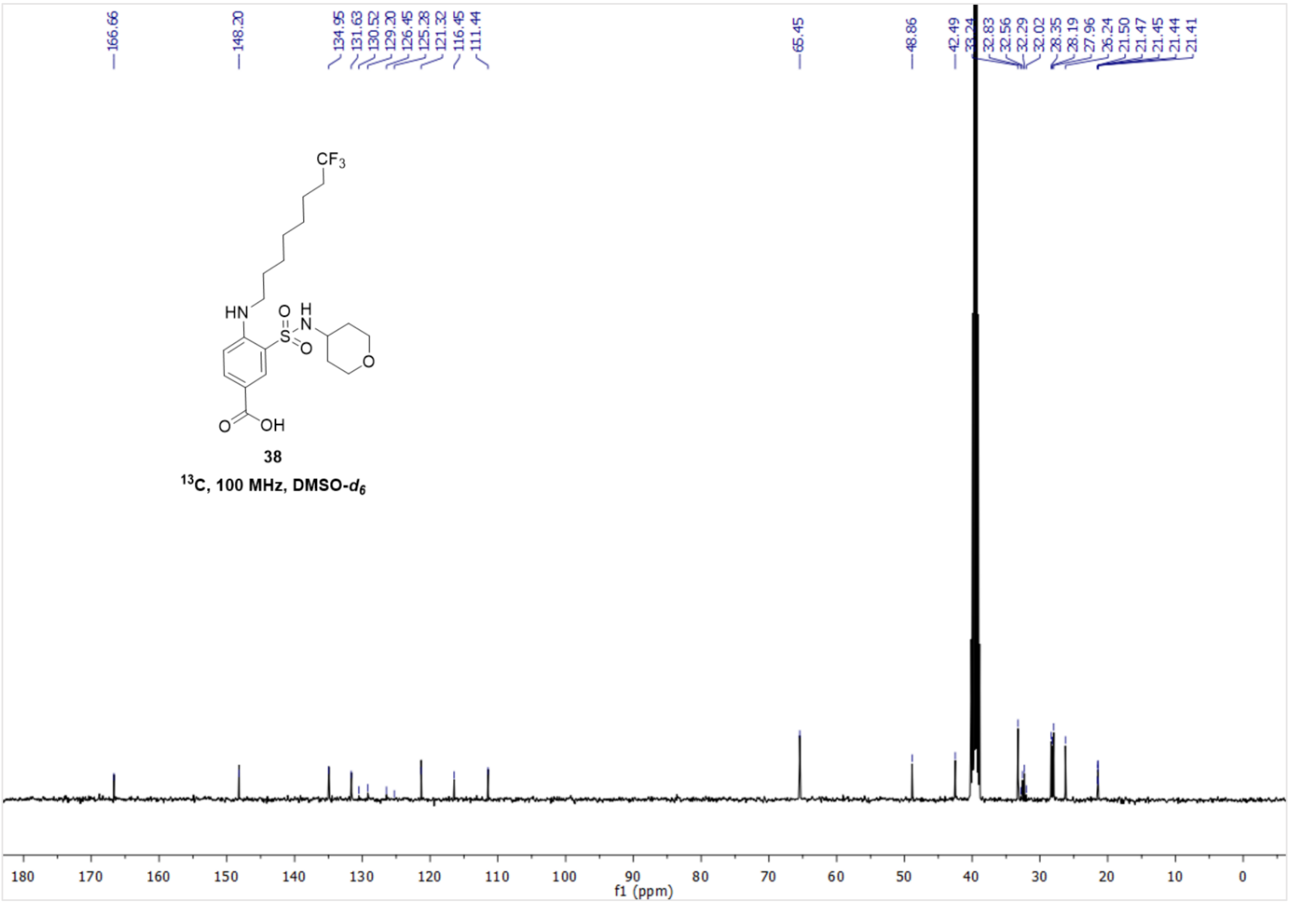
166.66
148.20
134.95
131.63
130.52
129.20
126.45
125.28
121.32
116.45
111.44
65.45
48.86
42.49
33.24
32.83
32.56
32.29
32.02
28.35
28.19
27.96
26.24
21.50
21.47
21.45
21.44
21.41
CF3
HN
O
S
O
H
N
O
O
OH
38
13C, 100 MHz, DMSO-d6
f1 (ppm)

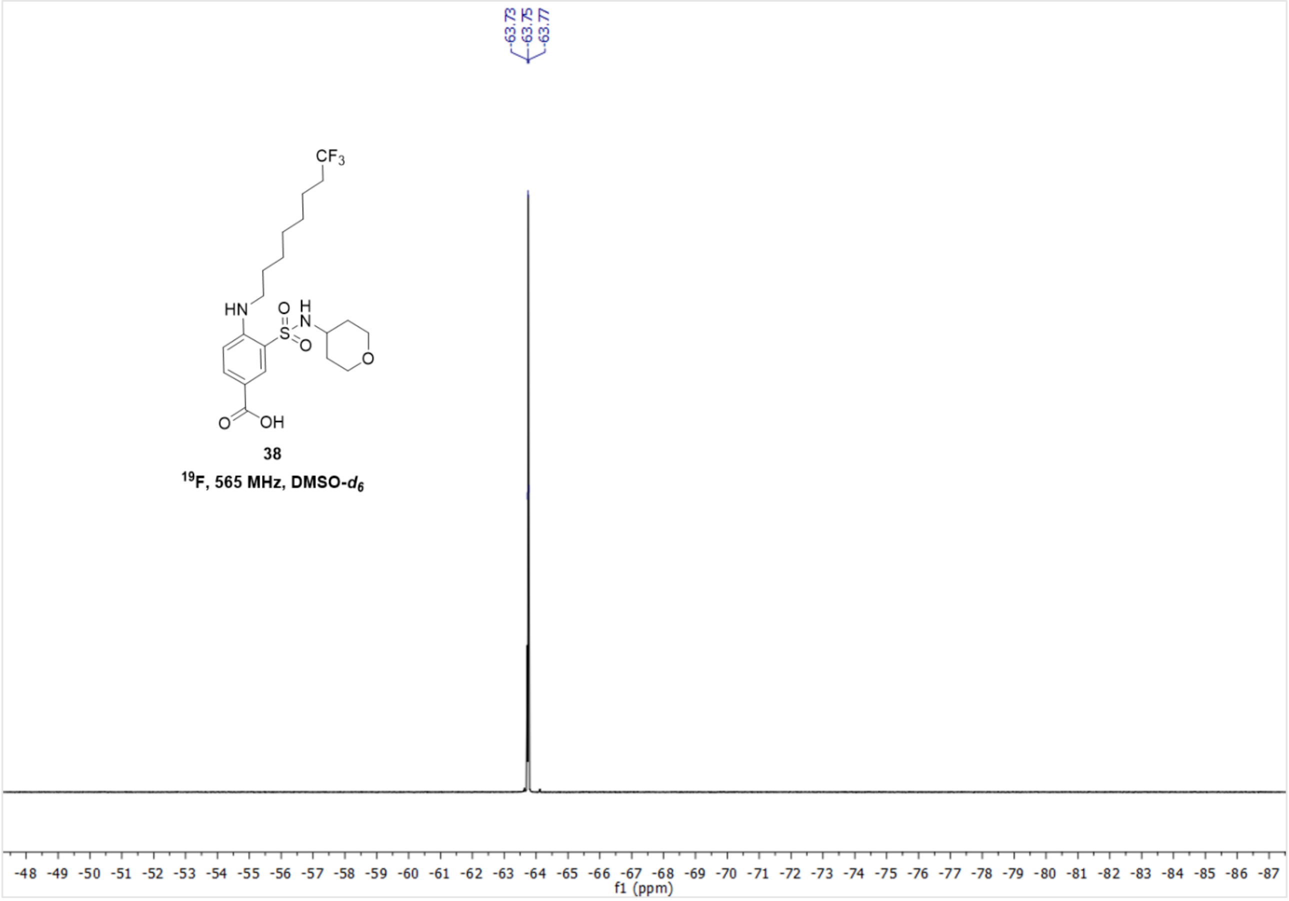
-63.73
-63.75
-63.77
CF3
HN
O
S
O
H
N
O
O
OH
38
19F, 565 MHz, DMSO-d6
f1 (ppm)

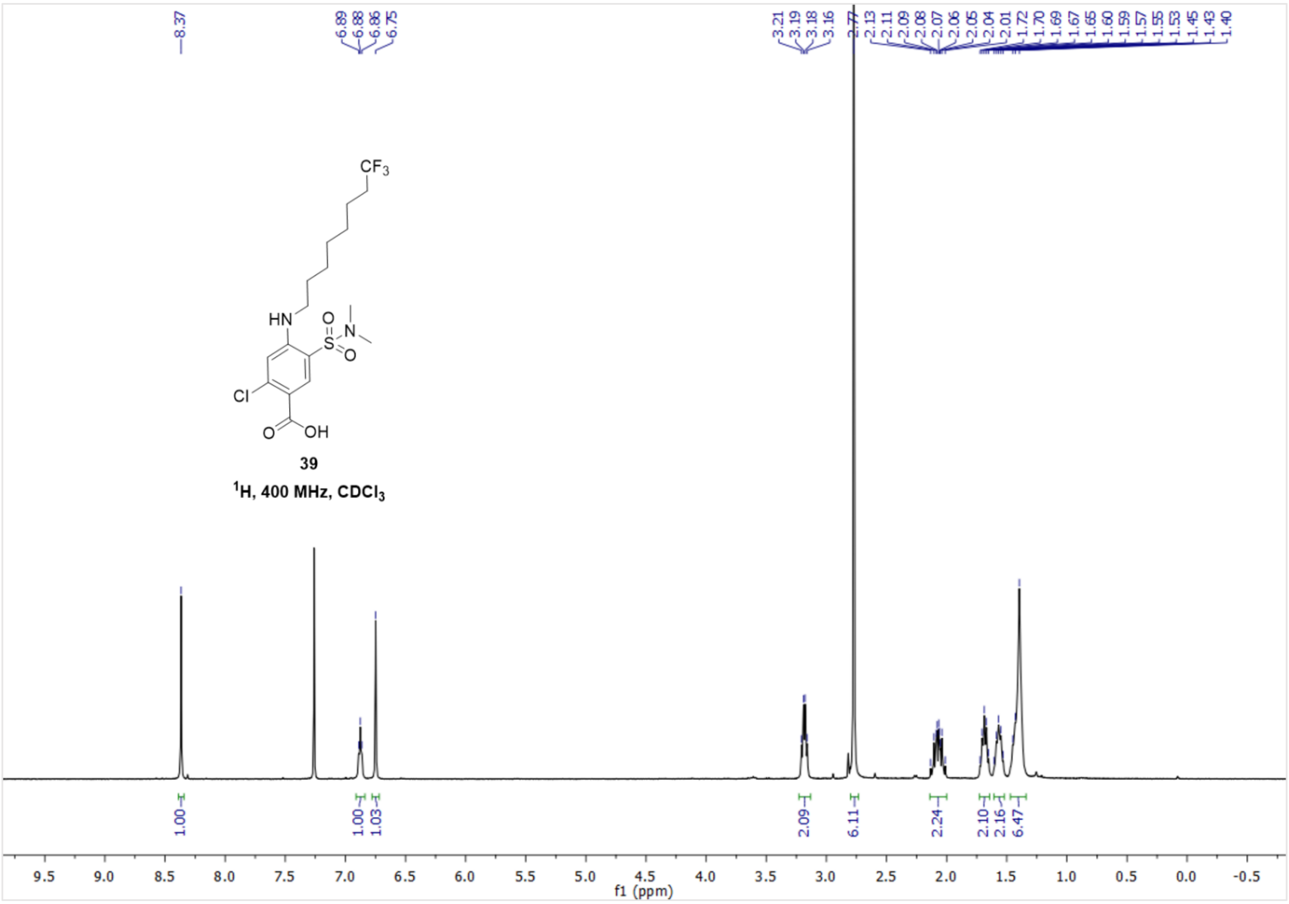
8.37
6.89
6.88
6.86
6.75
3.21
3.19
3.18
3.16
2.77
2.13
2.11
2.09
2.08
2.07
2.06
2.05
2.04
2.01
1.72
1.70
1.69
1.67
1.65
1.60
1.59
1.57
1.55
1.53
1.45
1.43
1.40
CF3
HN
O
S
O
N
Cl
O
OH
39
1H, 400 MHz, CDCl3
1.00
1.00
1.03
2.09
6.11
2.24
2.10
2.16
6.47
f1 (ppm)

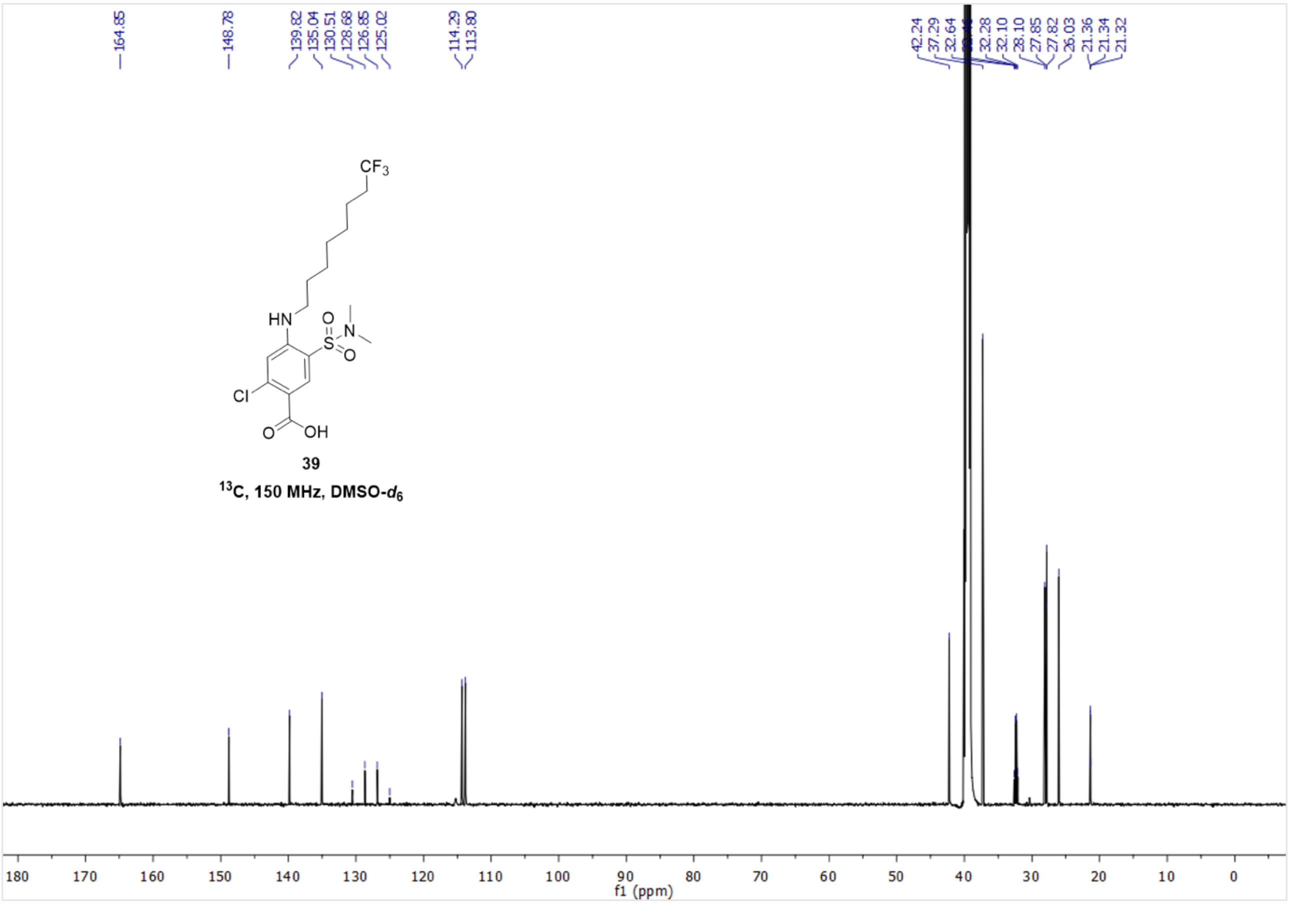
164.85
148.78
139.82
135.04
130.51
128.68
126.85
125.02
114.29
113.80
42.24
37.29
32.64
32.28
32.10
28.10
27.85
27.82
26.03
21.36
21.34
21.32
CF3
HN
O
S
N
O
Cl
O
OH
39
13C, 150 MHz, DMSO-d6
f1 (ppm)

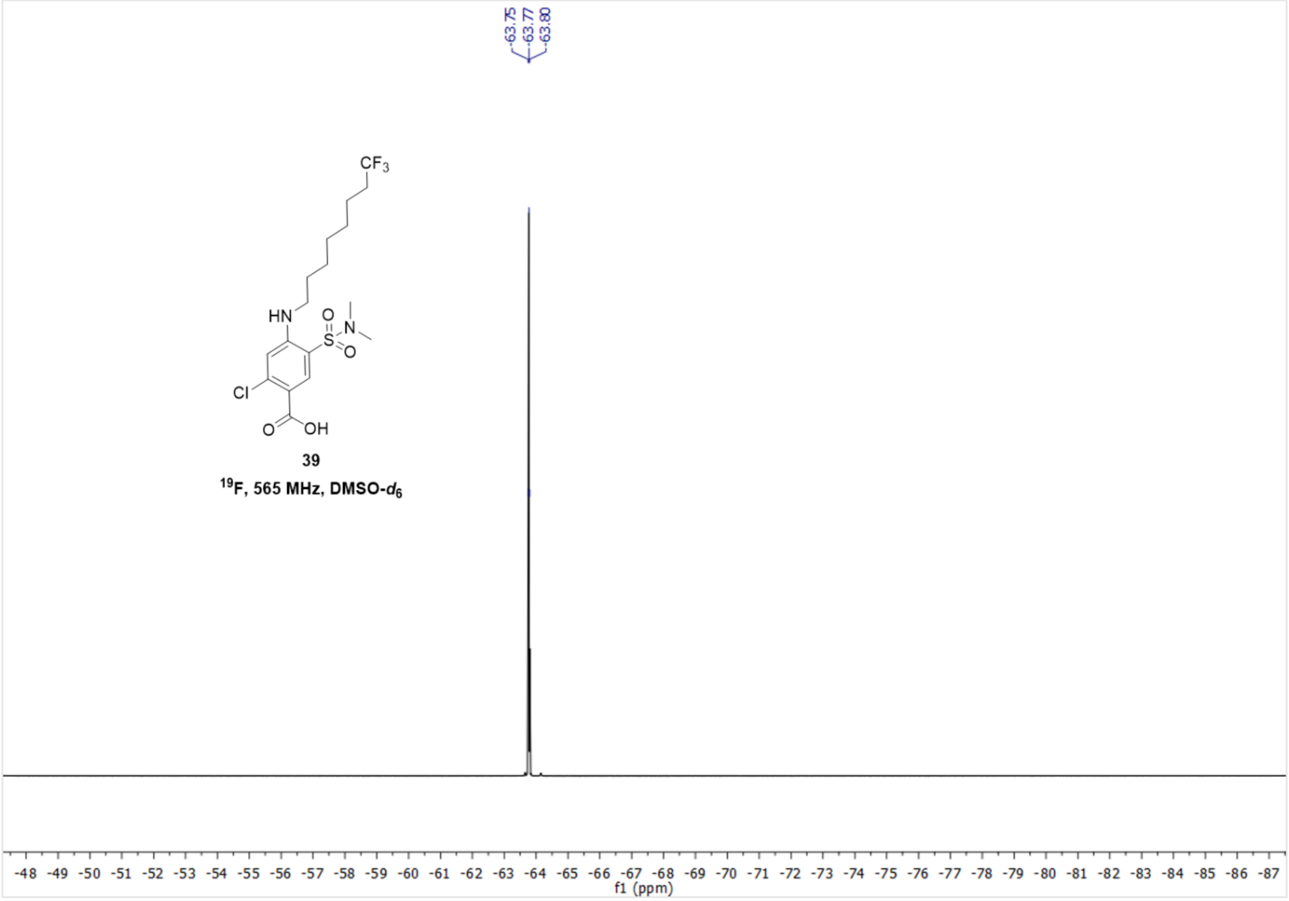
-63.75
-63.77
-63.80
CF3
HN
O
S
N
O
Cl
O
OH
39
19F, 565 MHz, DMSO-d6
f1 (ppm)

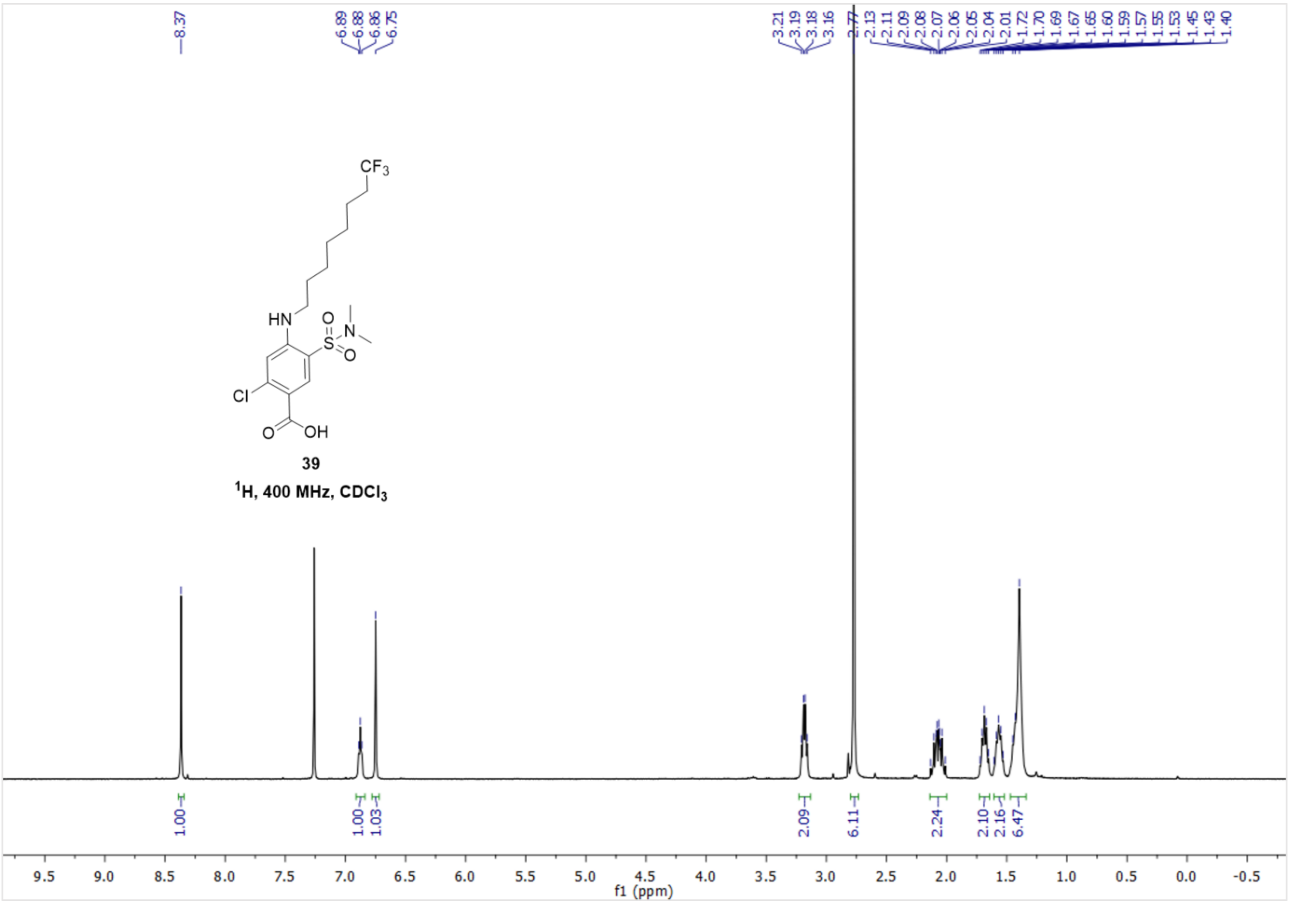
CF3
HN
O
S
N
O
Cl
O
OH
39
1H, 400 MHz, CDCl3
f1 (ppm)

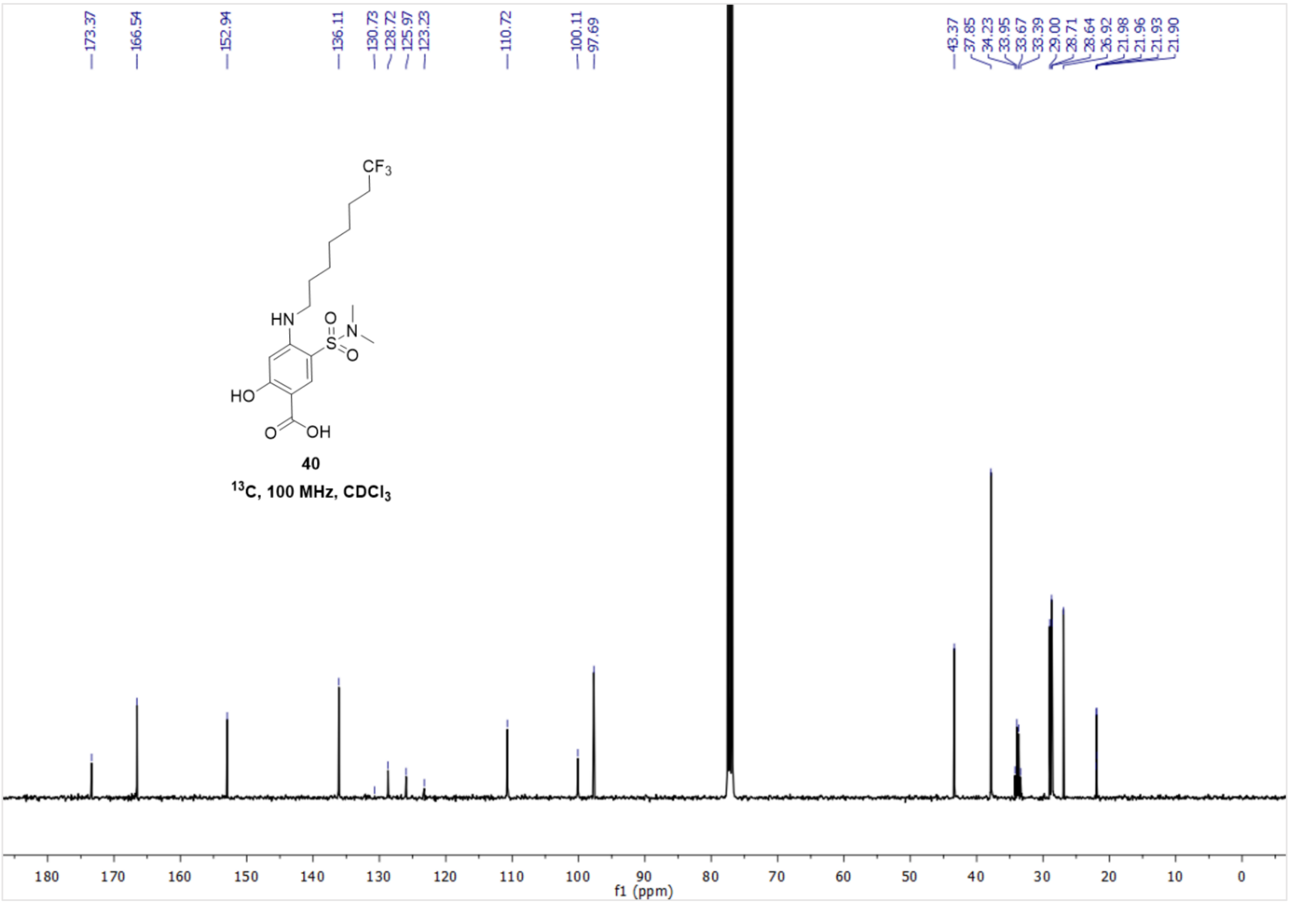
CF3
HN
O
S
N
O
HO
O
OH
40
13C, 100 MHz, CDCl3
f1 (ppm)

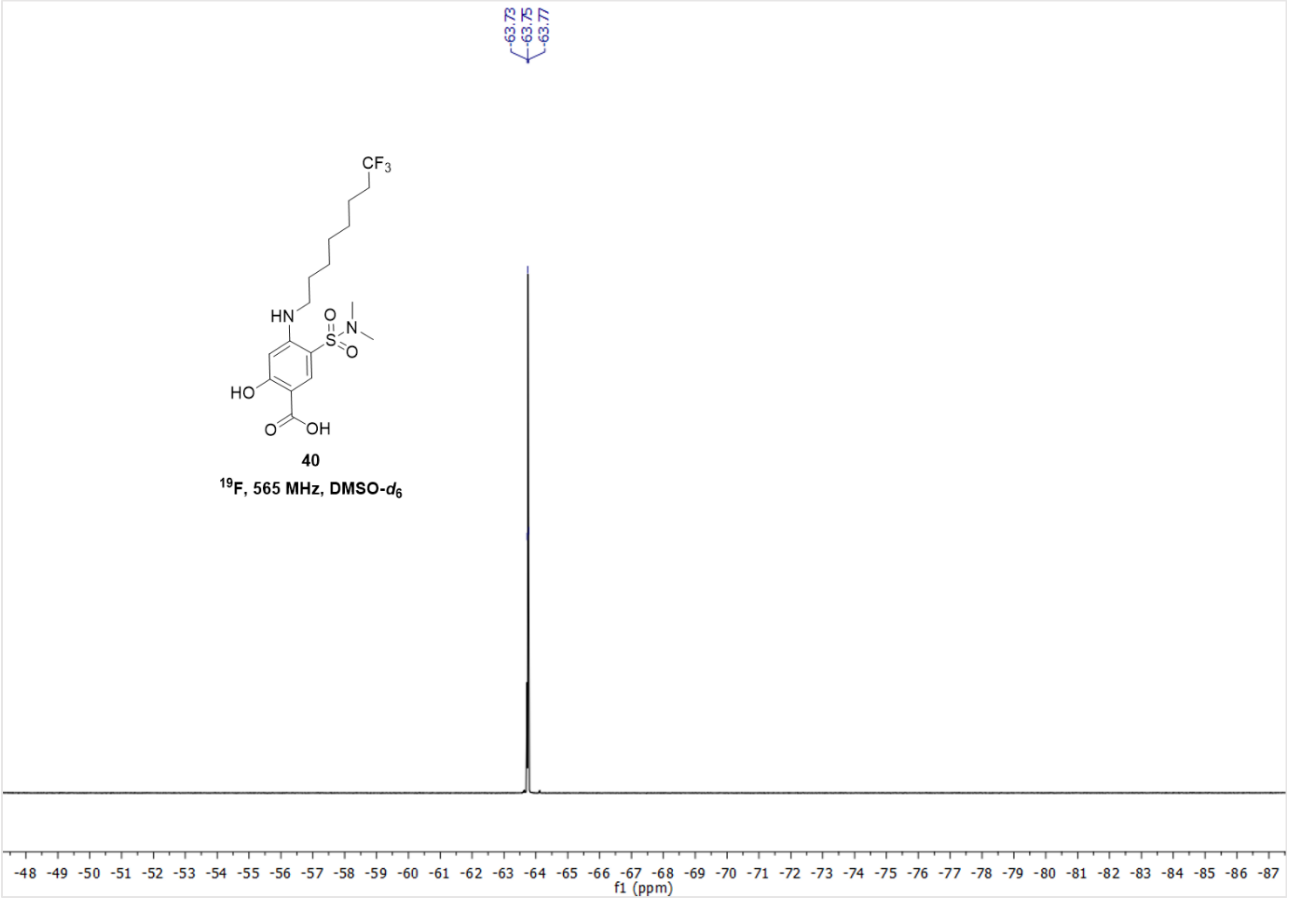
-63.73
-63.75
-63.77
CF3
HN
O
S
N
O
HO
O
OH
40
19F, 565 MHz, DMSO-d6
f1 (ppm)

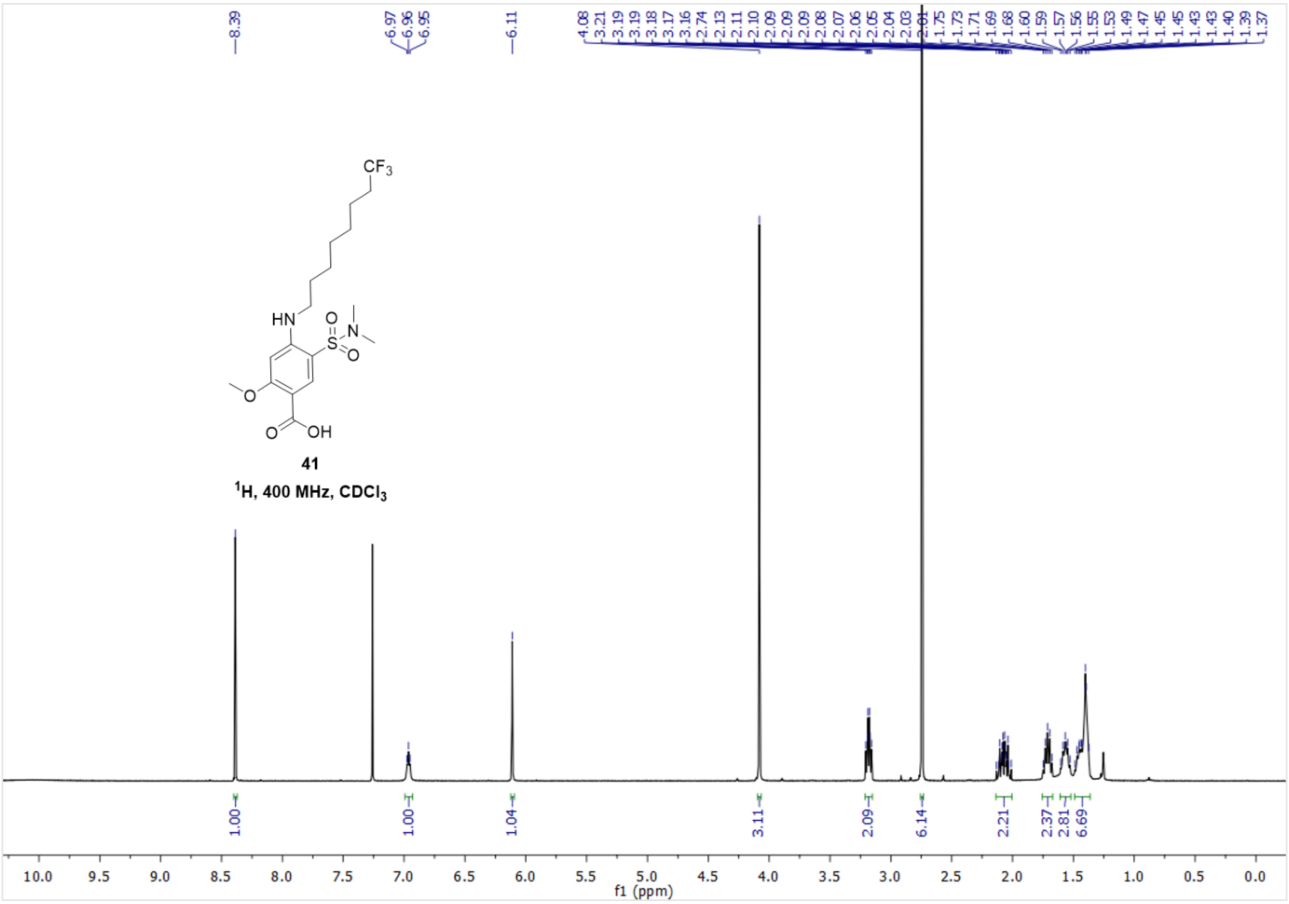
8.39
6.97
6.96
6.95
6.11
4.08
CF3
HN
O
S
N
O
O
O
OH
41
1H, 400 MHz, CDCl3
1.00
1.00
1.04
3.11
2.09
6.14
2.21
2.37
2.81
6.69
f1 (ppm)

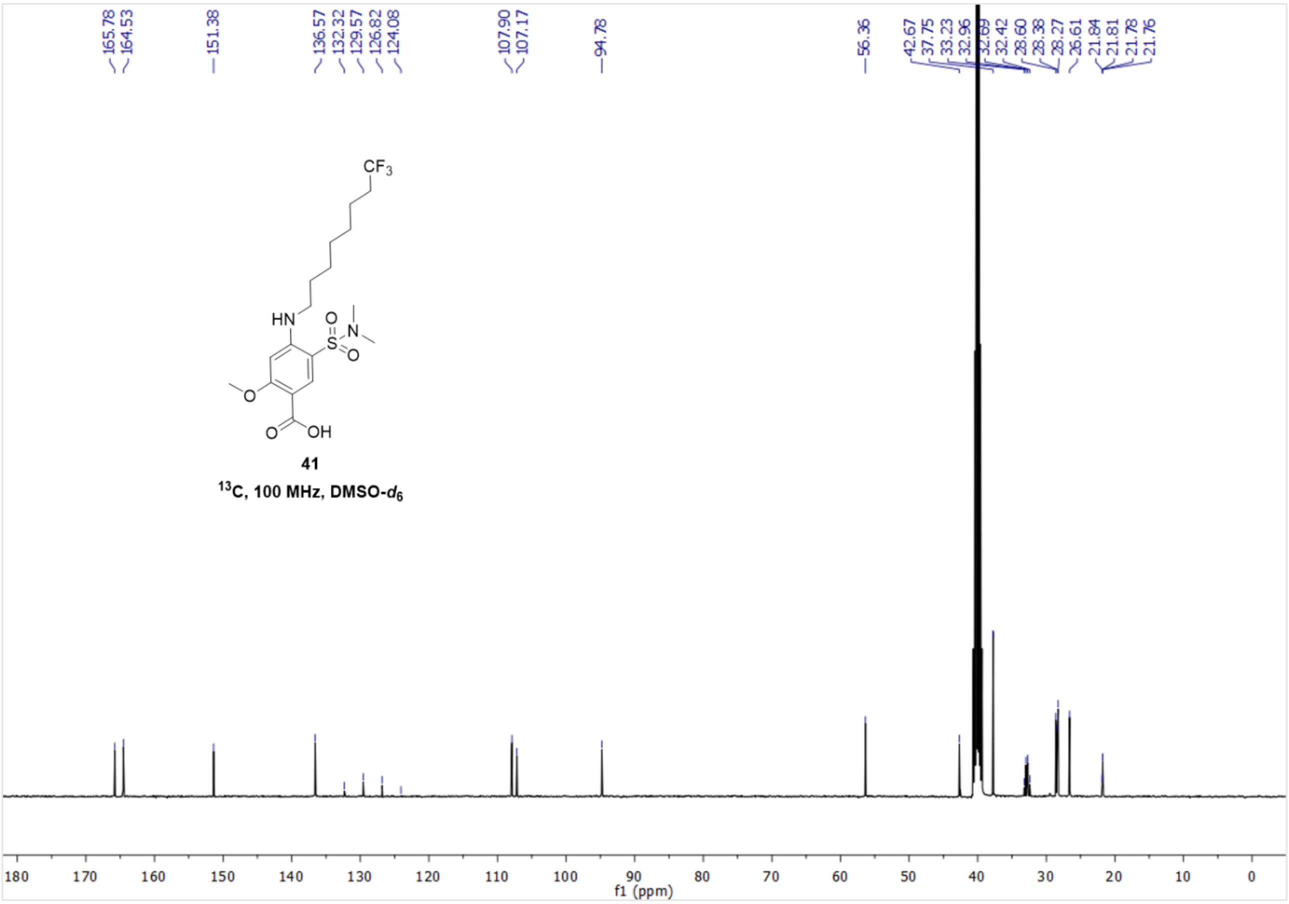

165.78
164.53
151.38
136.57
132.32
129.57
126.82
124.08
107.90
107.17
94.78
56.36
42.67
37.75
33.23
32.96
32.69
32.42
28.60
28.38
28.27
26.61
21.84
21.81
21.78
21.76
CF3
HN
O
S
N
O
O
O
OH
41
13C, 100 MHz, DMSO-d6
f1 (ppm)

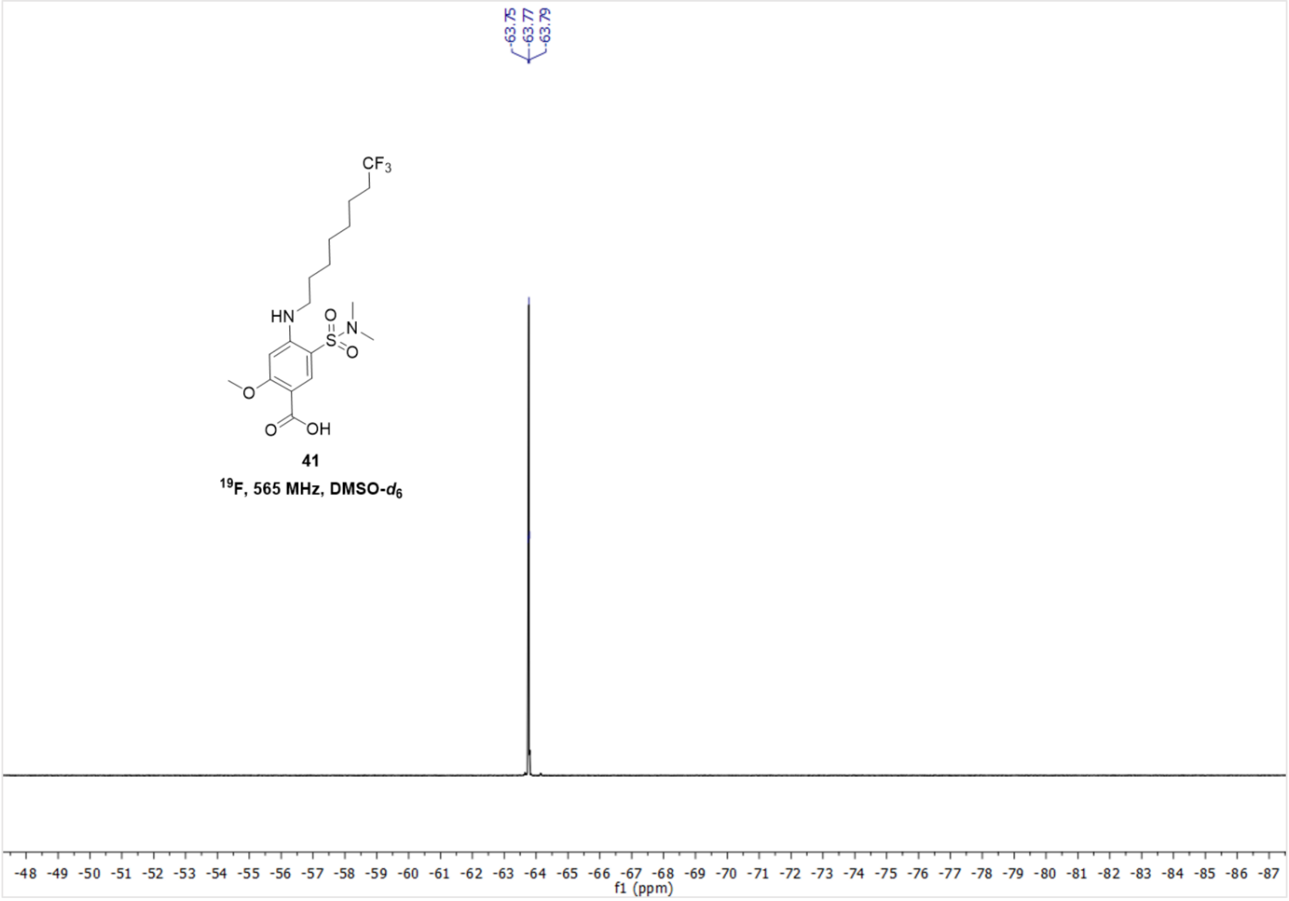

-63.75
-63.77
-63.79
CF3
HN
O
S
N
O
O
O
OH
41
19F, 565 MHz, DMSO-d6
f1 (ppm)

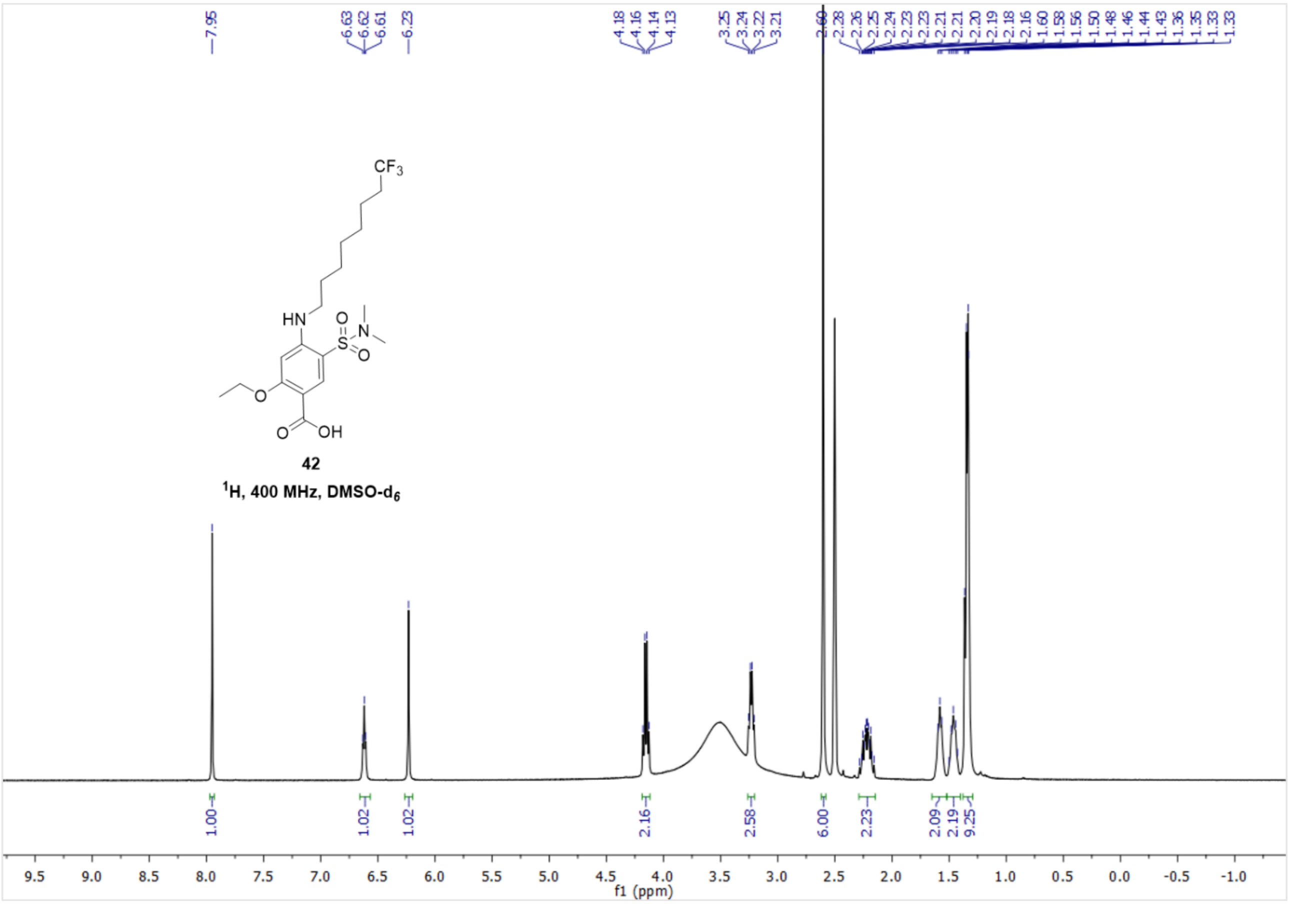

42
^{1}H, 400 MHz, DMSO-d$_6$
f1 (ppm)

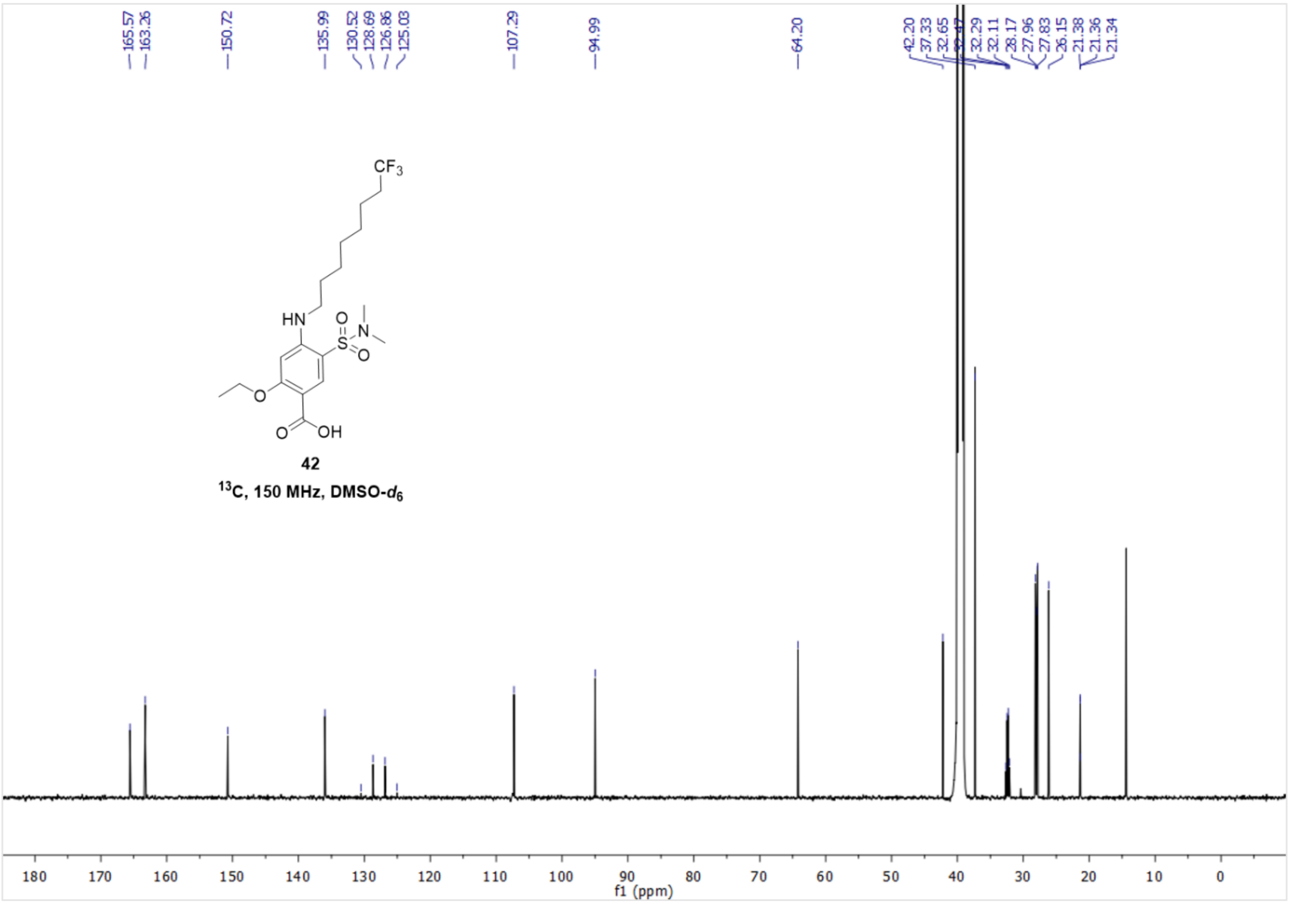

42
^{13}C, 150 MHz, DMSO-d_6
f1 (ppm)

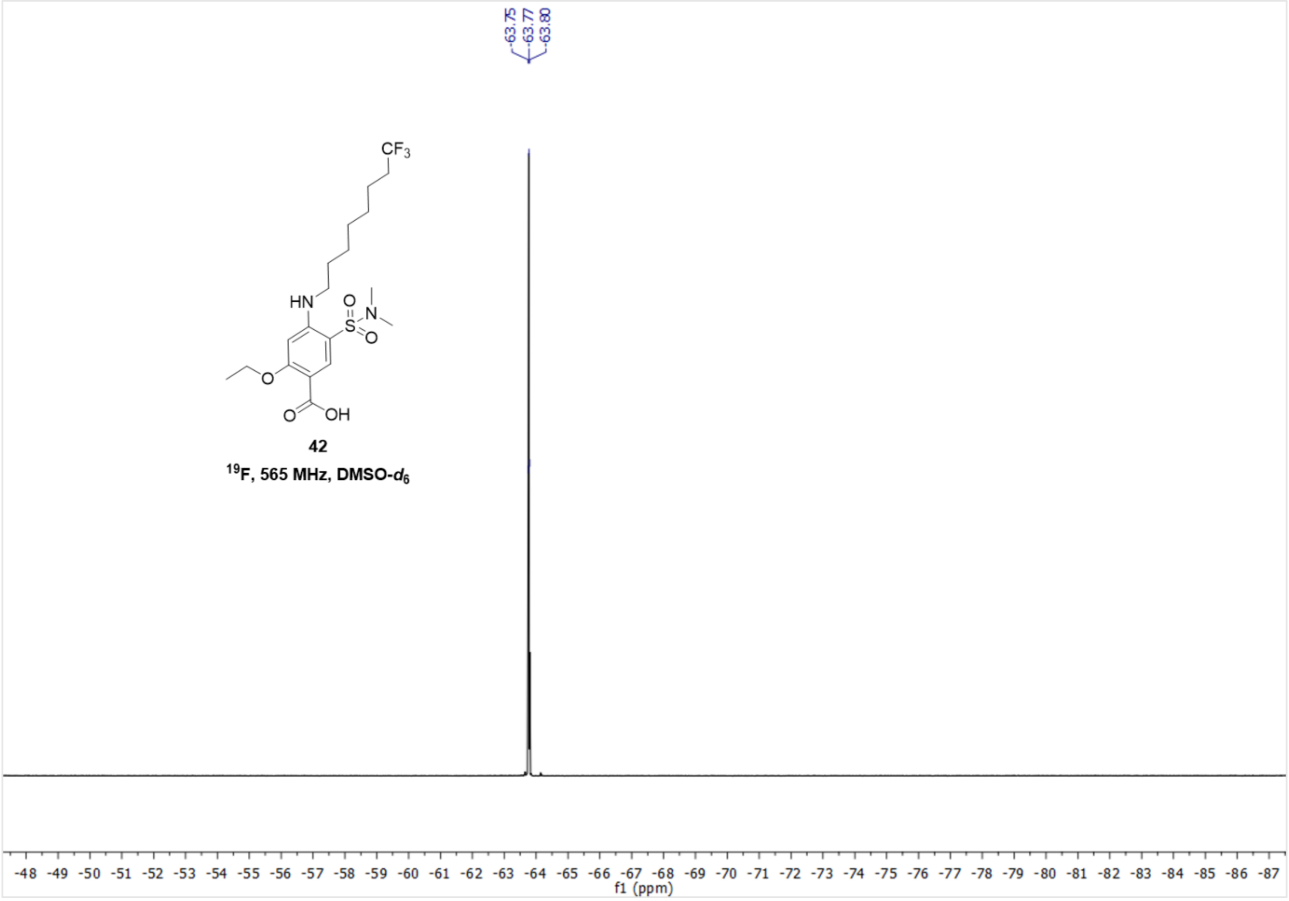
-63.75
-63.77
-63.80
CF3
HN
O
S
N
O
O
O
OH
42
19F, 565 MHz, DMSO-d6
-48 -49 -50 -51 -52 -53 -54 -55 -56 -57 -58 -59 -60 -61 -62 -63 -64 -65 -66 -67 -68 -69 -70 -71 -72 -73 -74 -75 -76 -77 -78 -79 -80 -81 -82 -83 -84 -85 -86 -87
f1 (ppm)

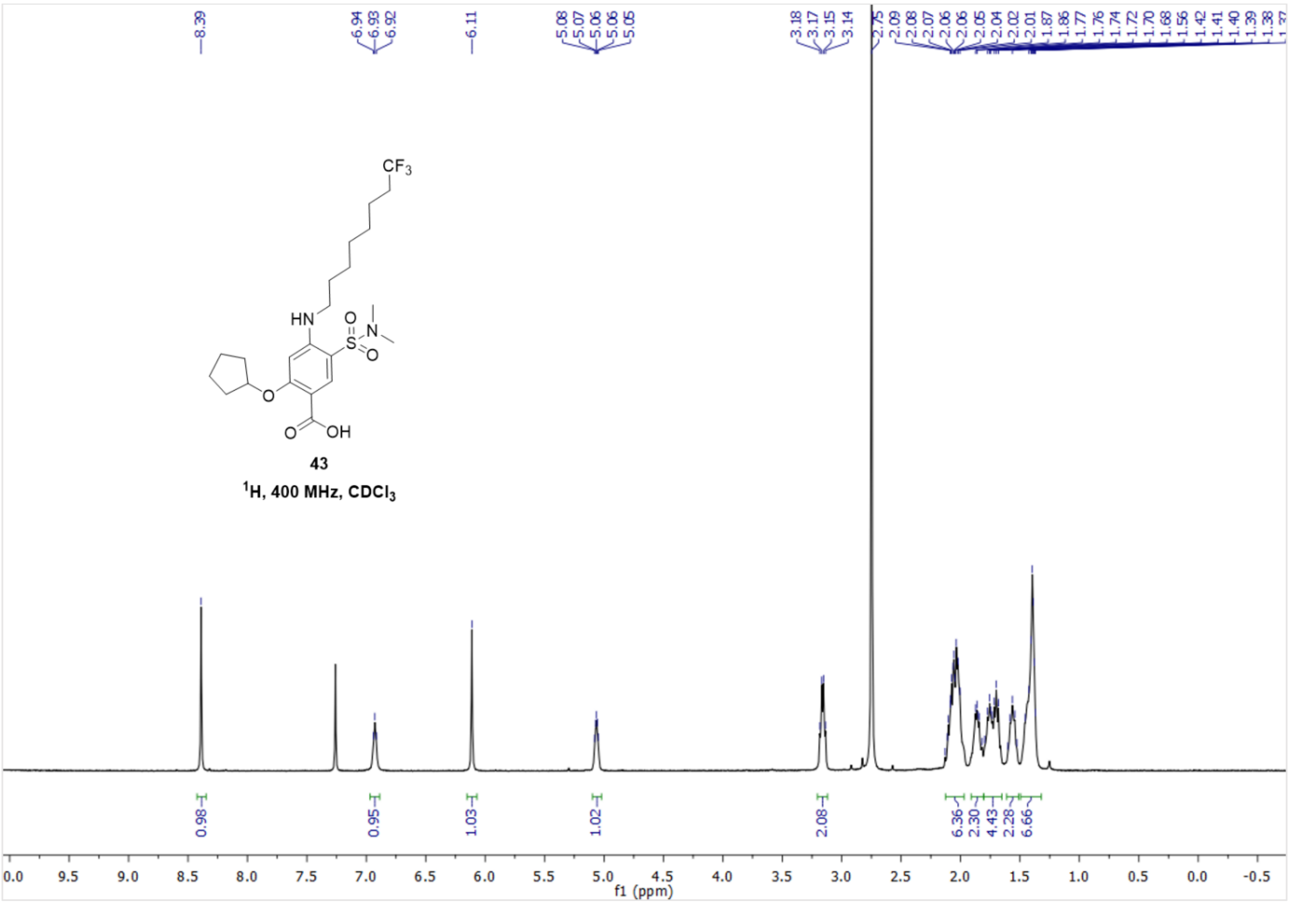
8.39
6.94
6.93
6.92
6.11
5.08
5.07
5.06
5.06
5.05
3.18
3.17
3.15
3.14
2.75
2.09
2.08
2.07
2.06
2.06
2.05
2.04
2.02
2.01
1.87
1.86
1.77
1.76
1.74
1.72
1.70
1.68
1.56
1.42
1.41
1.40
1.39
1.38
CF3
HN
O
S
N
O
O
O
OH
43
1H, 400 MHz, CDCl3
0.98
0.95
1.03
1.02
2.08
6.36
2.30
4.43
2.28
6.66
0.0 9.5 9.0 8.5 8.0 7.5 7.0 6.5 6.0 5.5 5.0 4.5 4.0 3.5 3.0 2.5 2.0 1.5 1.0 0.5 0.0 -0.5
f1 (ppm)

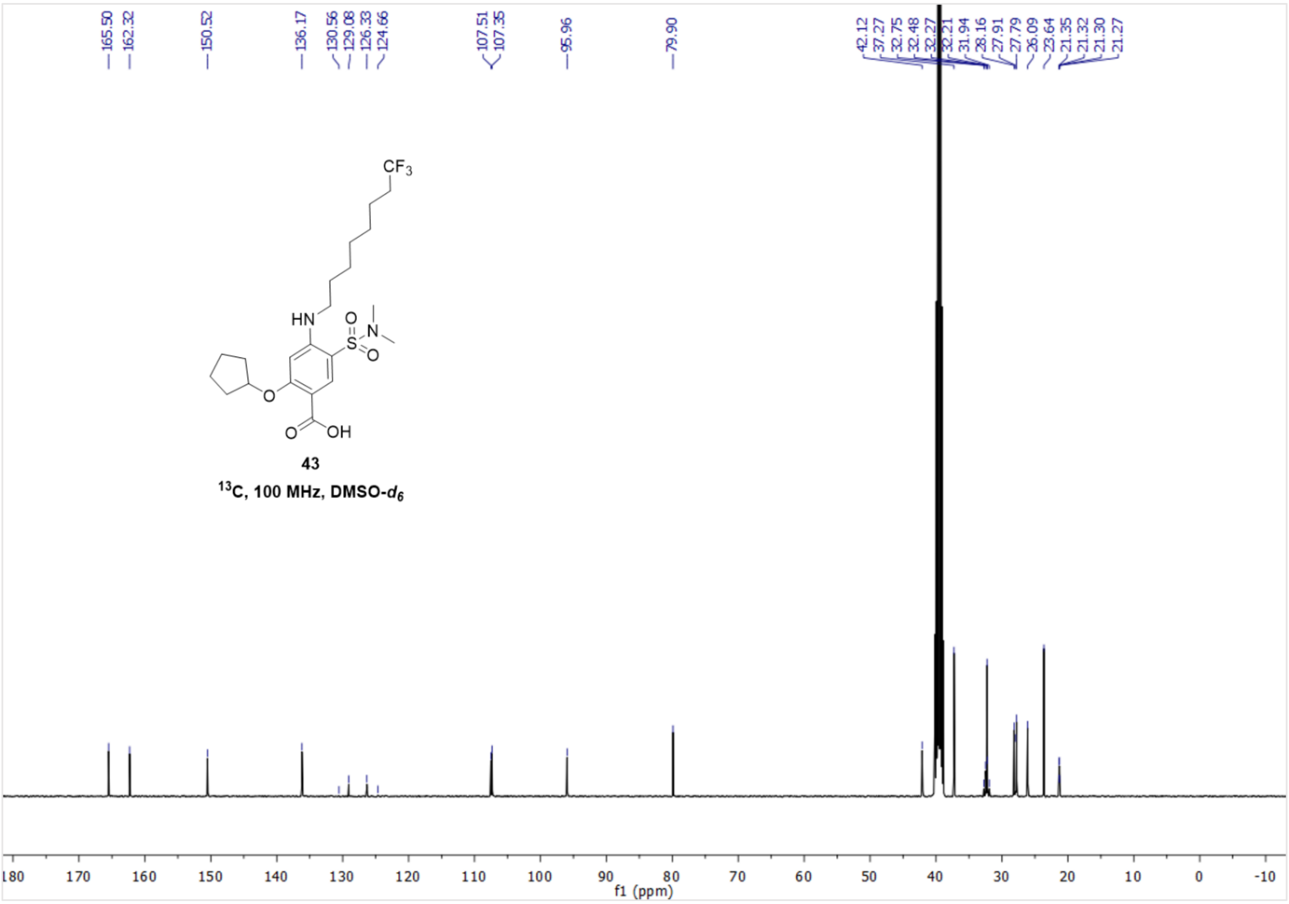
165.50
162.32
150.52
136.17
130.56
129.08
126.33
124.66
107.51
107.35
95.96
79.90
42.12
37.27
32.75
32.48
32.27
32.21
31.94
28.16
27.91
27.79
26.09
23.64
21.35
21.32
21.30
21.27
CF3
HN
O
S
N
O
O
O
OH
43
13C, 100 MHz, DMSO-d6
f1 (ppm)

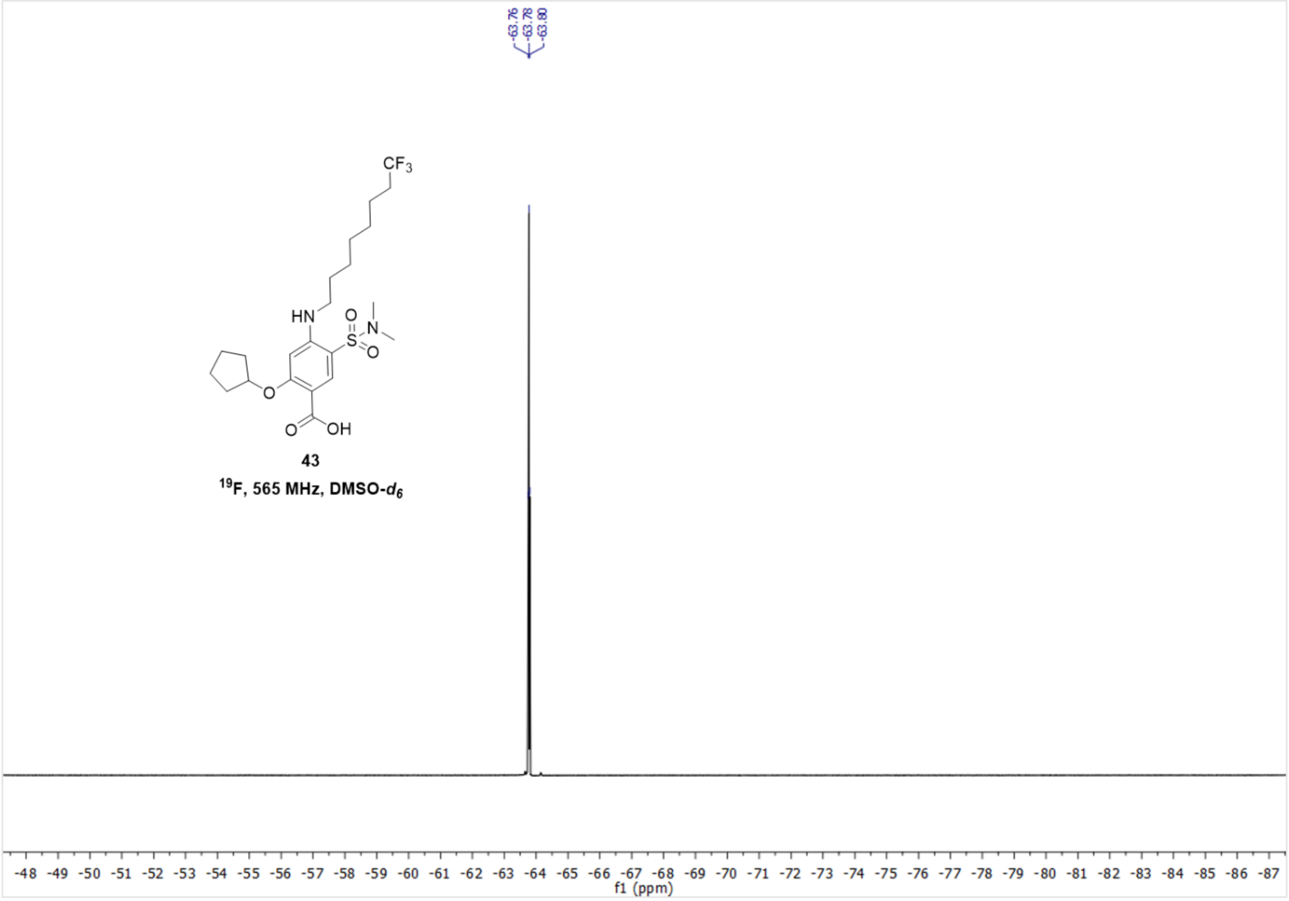
-63.76
-63.78
-63.80
CF3
HN
O
S
N
O
O
O
OH
43
19F, 565 MHz, DMSO-d6
f1 (ppm)

9. Chromatographic analysis of key compounds.

Performed on a Waters ACQUITY UPLC/MS system consisting of a SQD (Single Quadrupole Detector) Mass Spectrometer equipped with an Electrospray Ionization interface and a Photodiode Array Detector. The PDA range was 210-400nm. Analyses were performed on an ACQUITY UPLC BEH C_{18} column (100x2.1mmID, particle size 1.7µm) with a VanGuard BEH C_{18} pre-column (5x2.1mmID, particle size 1.7µm). Mobile phase was 10mM NH_4OAc in H_2O at pH 5 adjusted with AcOH (A) and 10mM NH_4OAc in MeCN-H_2O (95:5) at pH 5 (B). Linear Gradient: start at 10% B, hold for 0.20min, then 10-90%B in 6min, 90-100%B in 0.10min and 100% B hold for 0.70min. Total run time: 7min. Electrospray ionization in positive and negative mode. All final compounds displayed ≥ 95% purity by UPLC/MS analysis with the exception of compounds **15** (92%) and **16** (90%).

Compound **1**

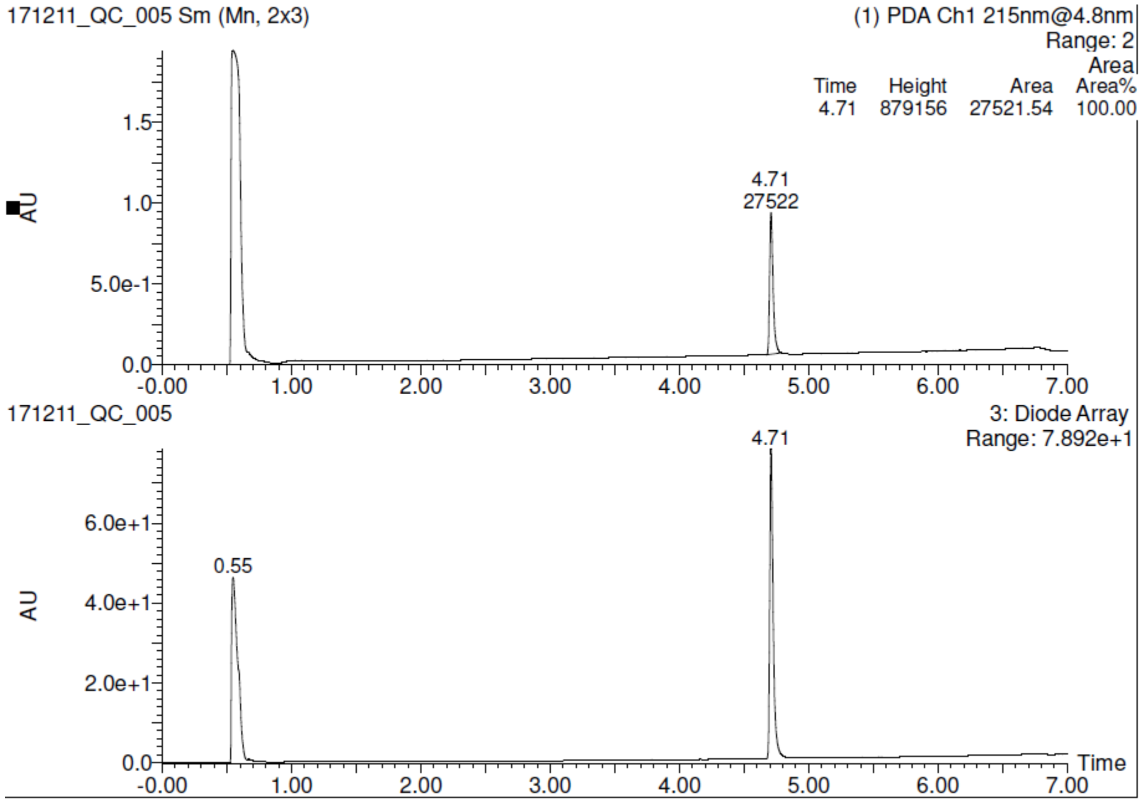

Compound **3**

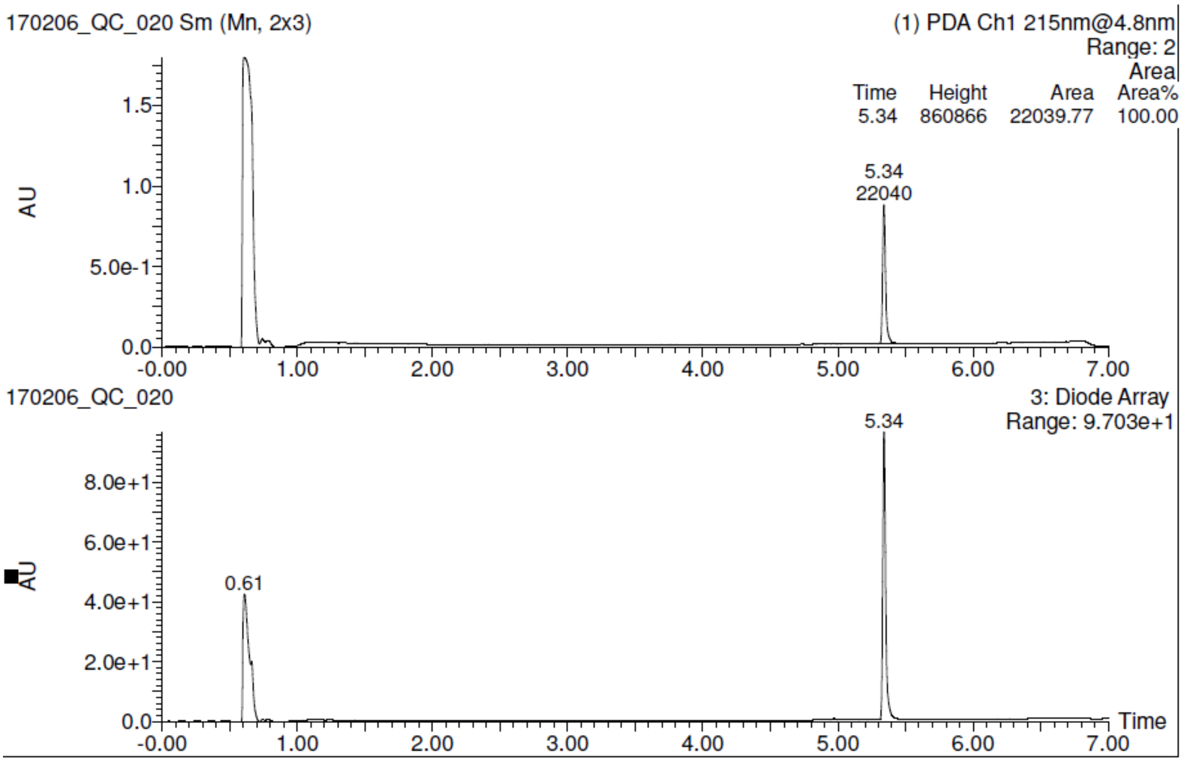

Compound **4**

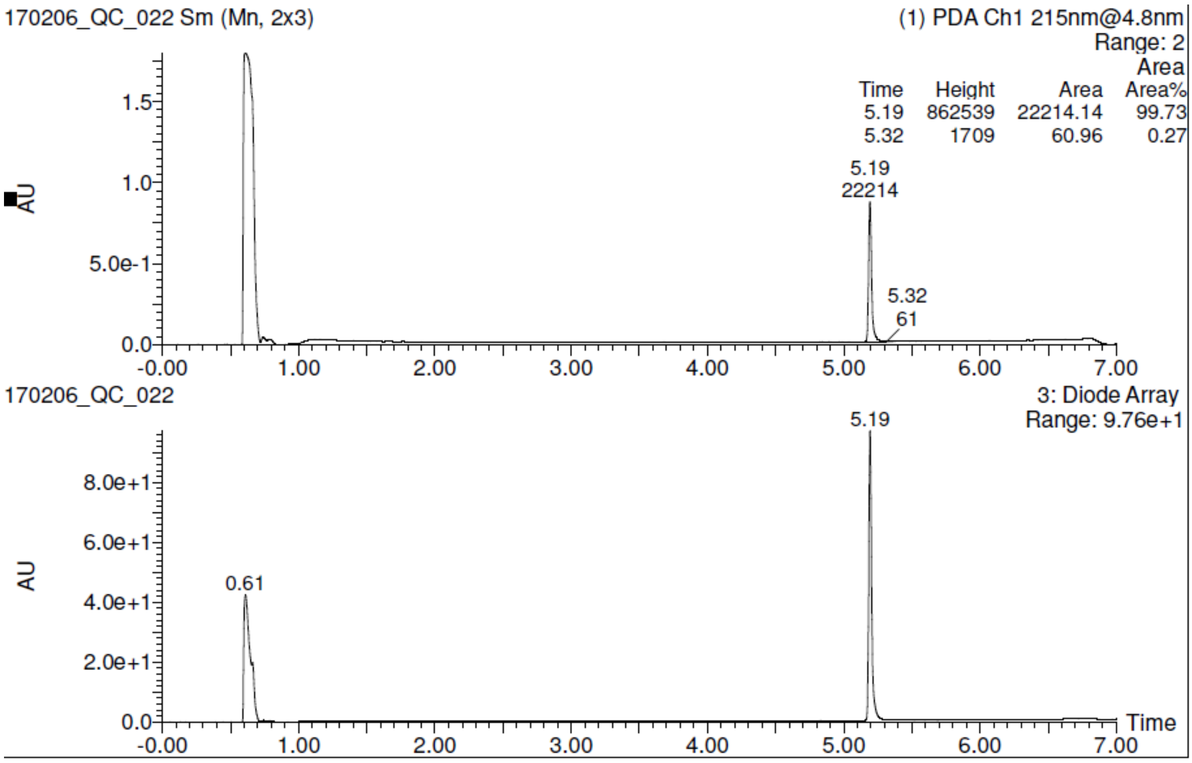

Compound **5**

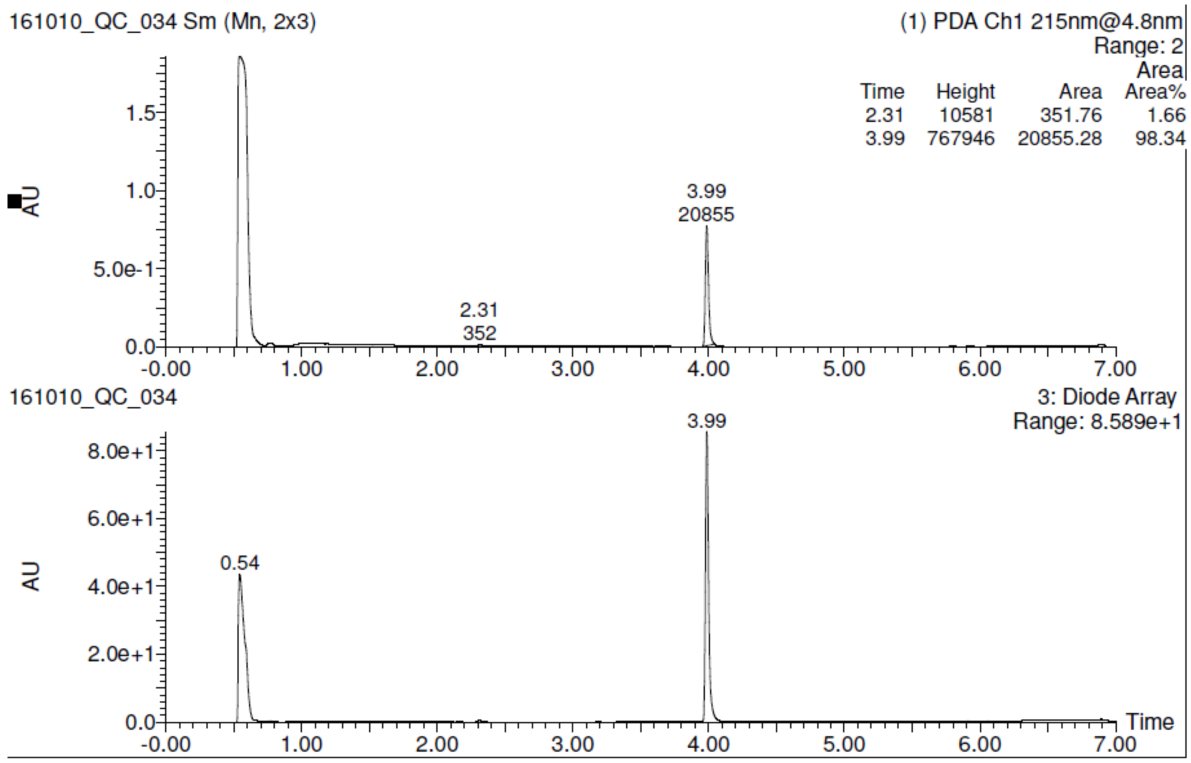

Compound **6**

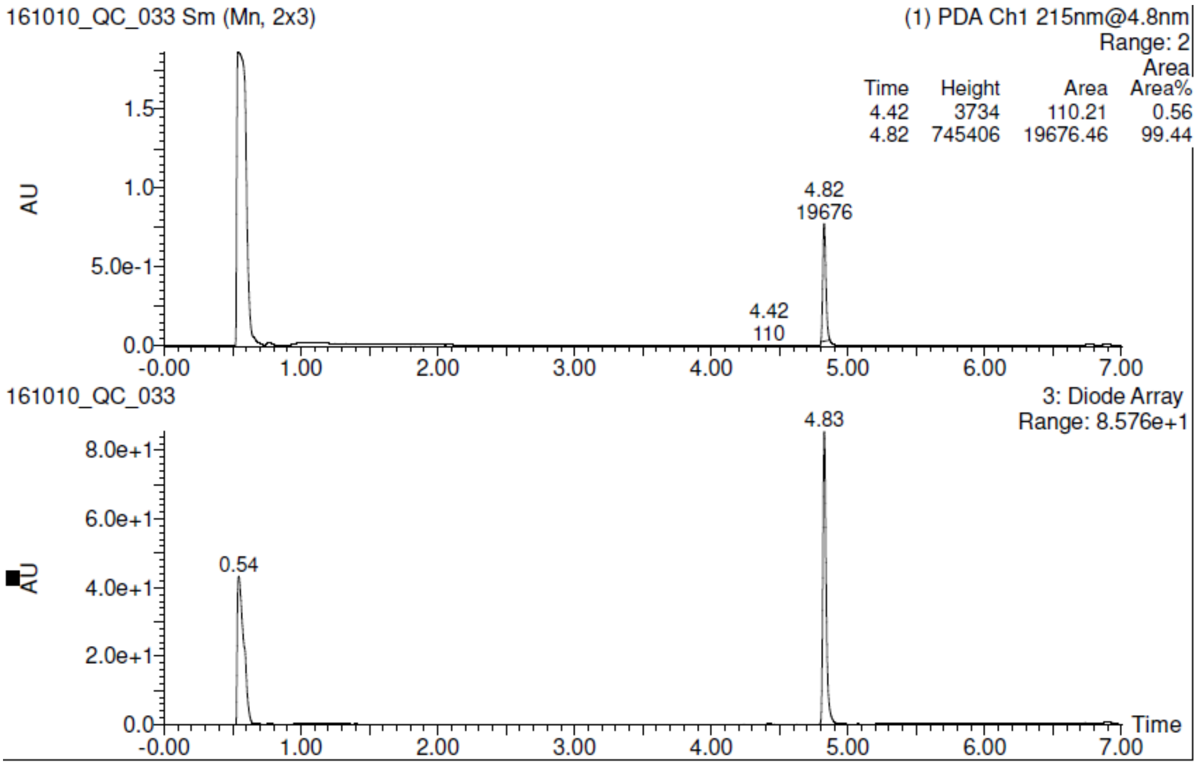

Compound **7**

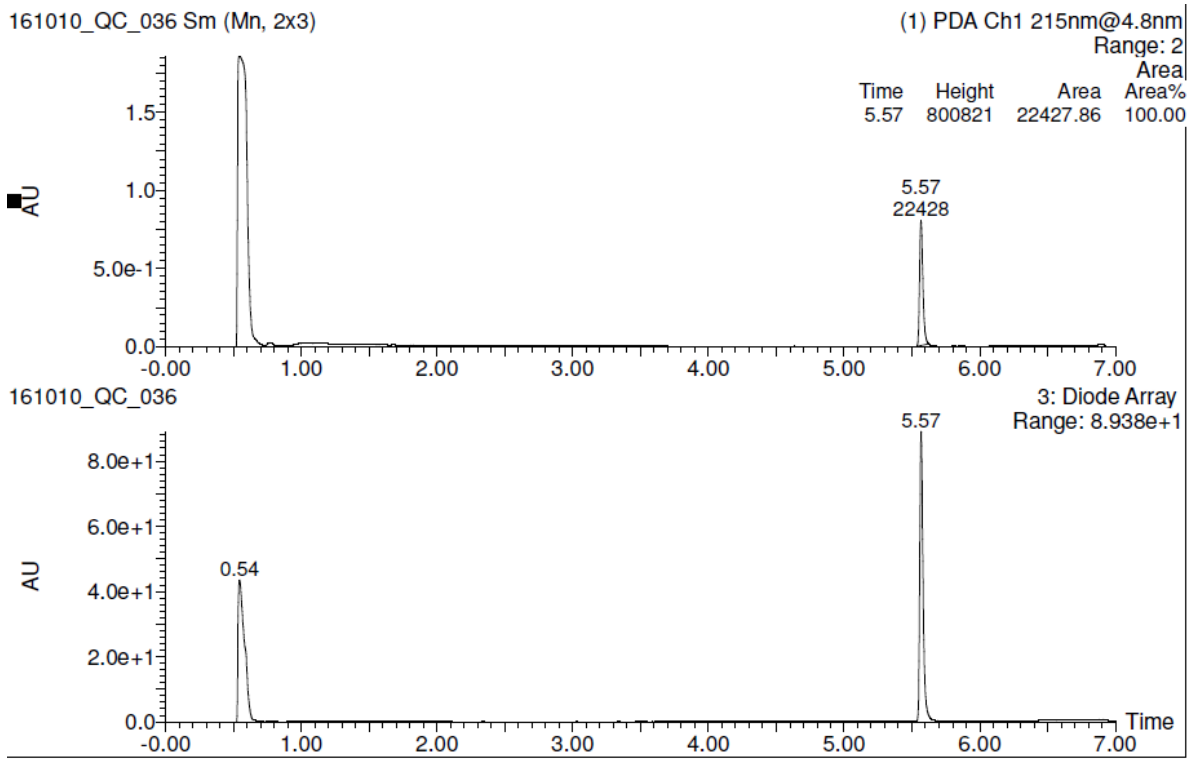

Compound **8**

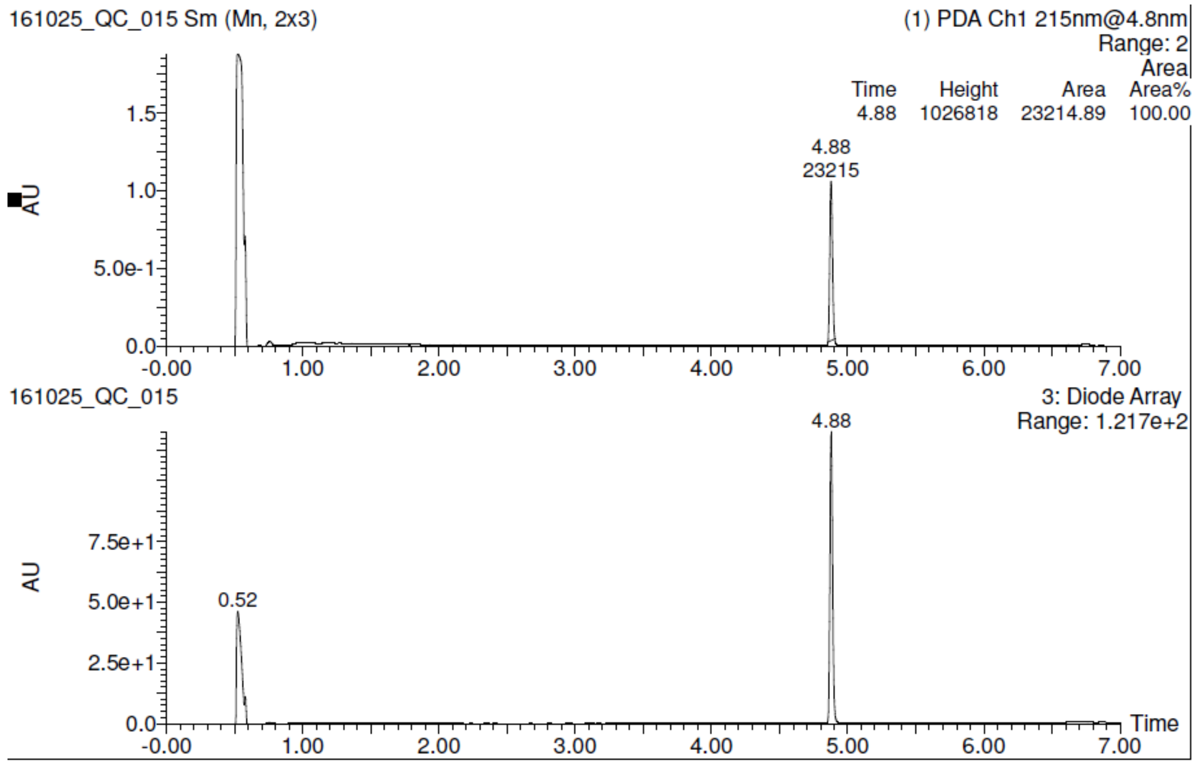

Compound **9**

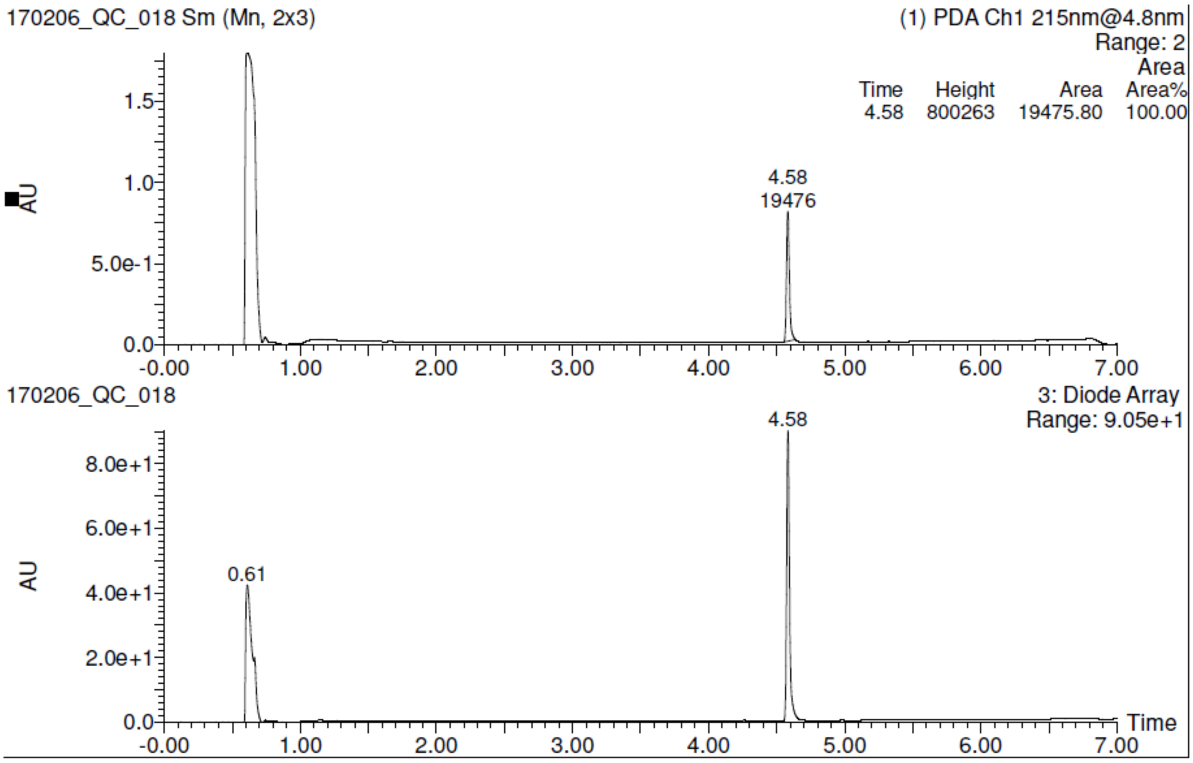

Compound **10**

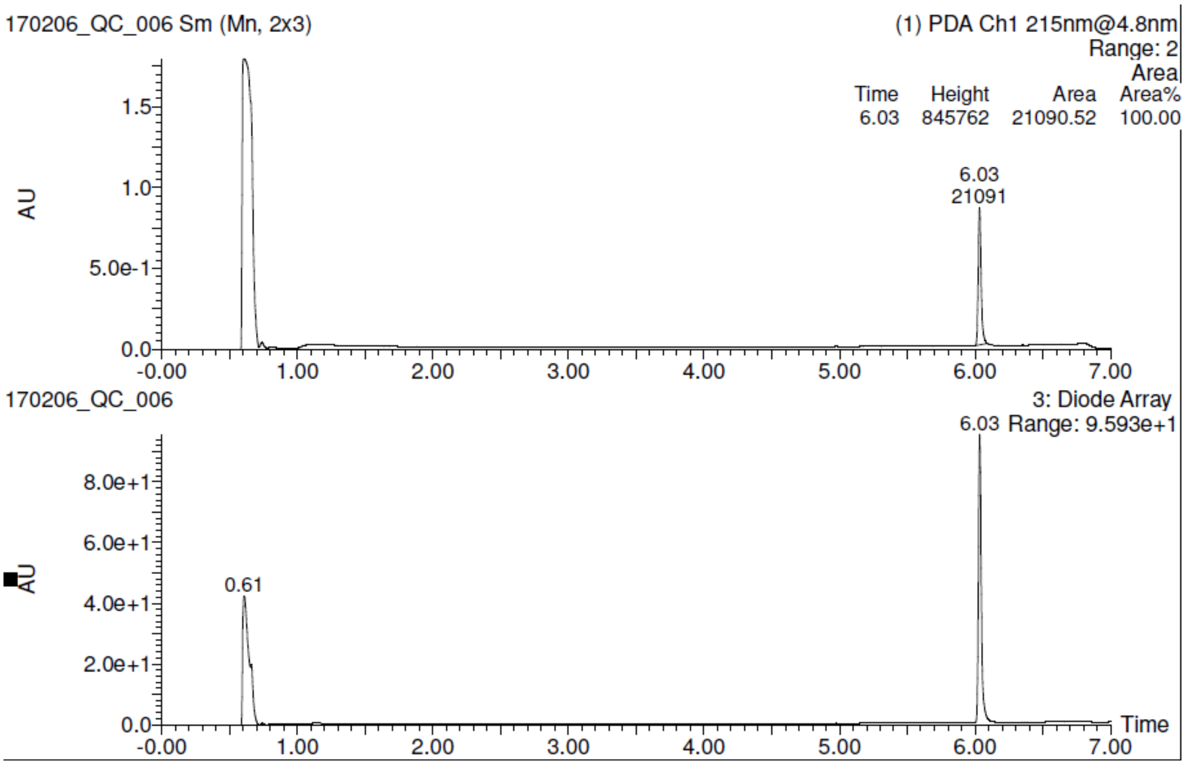

Compound **11**

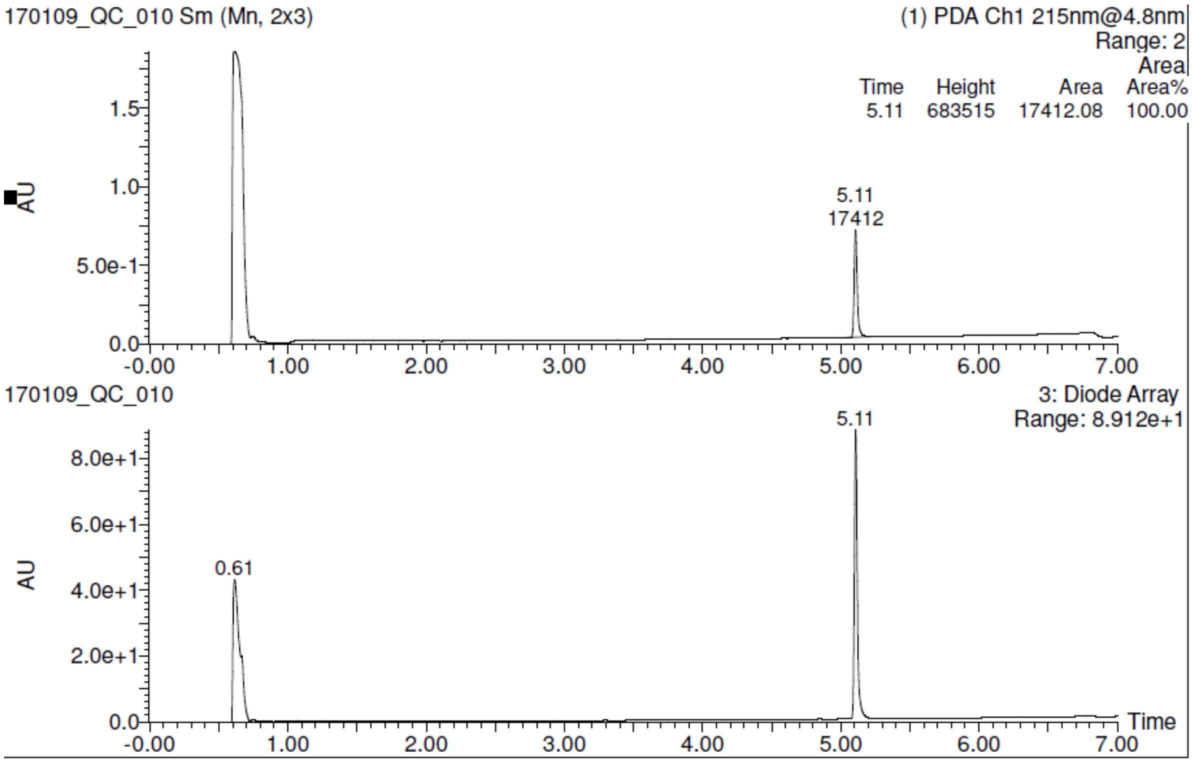

Compound **12**

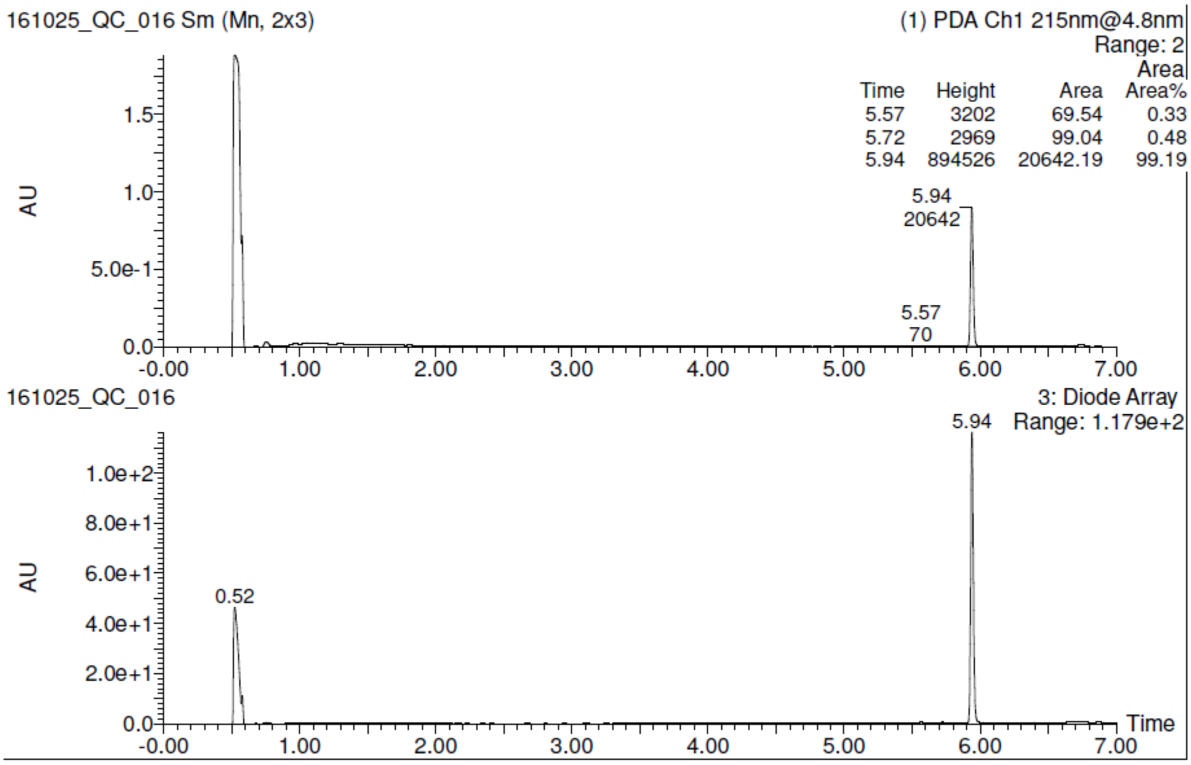

Compound **13**

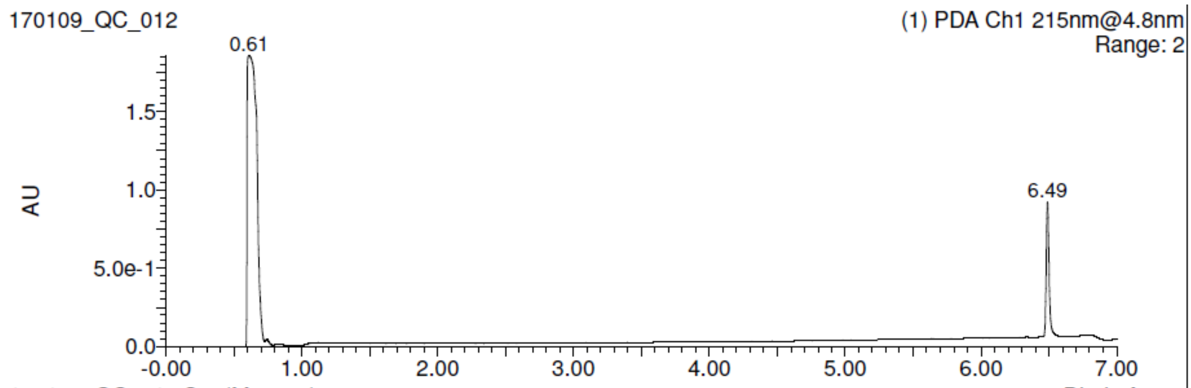

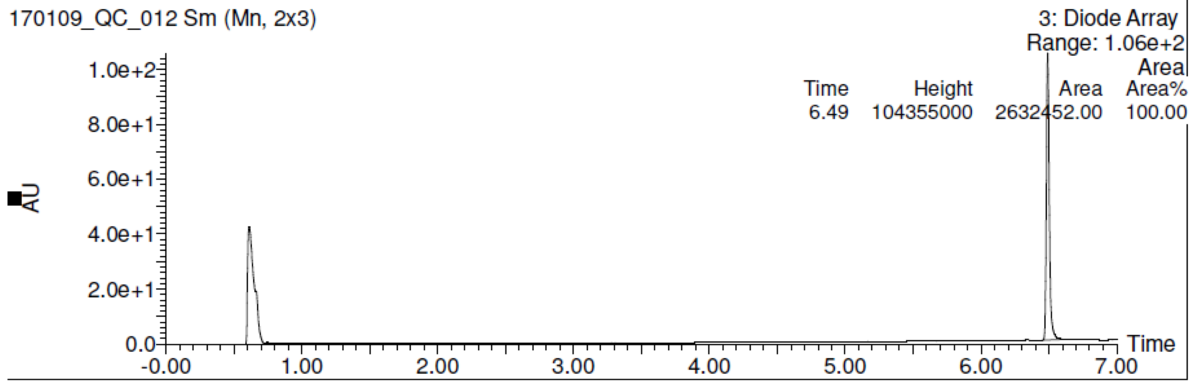

Compound **14**

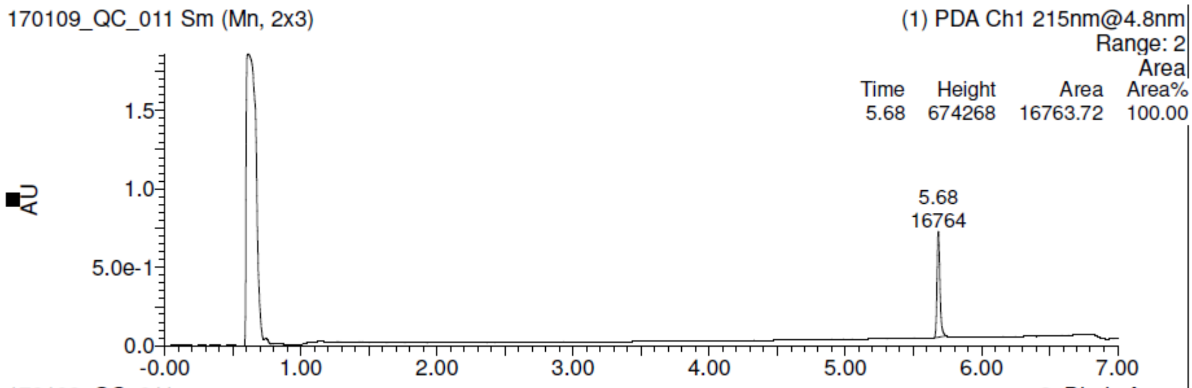

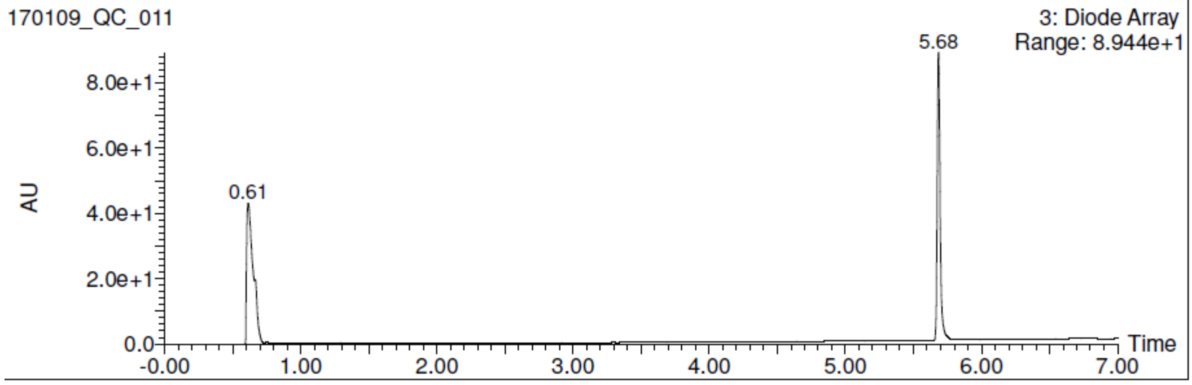

Compound **15**

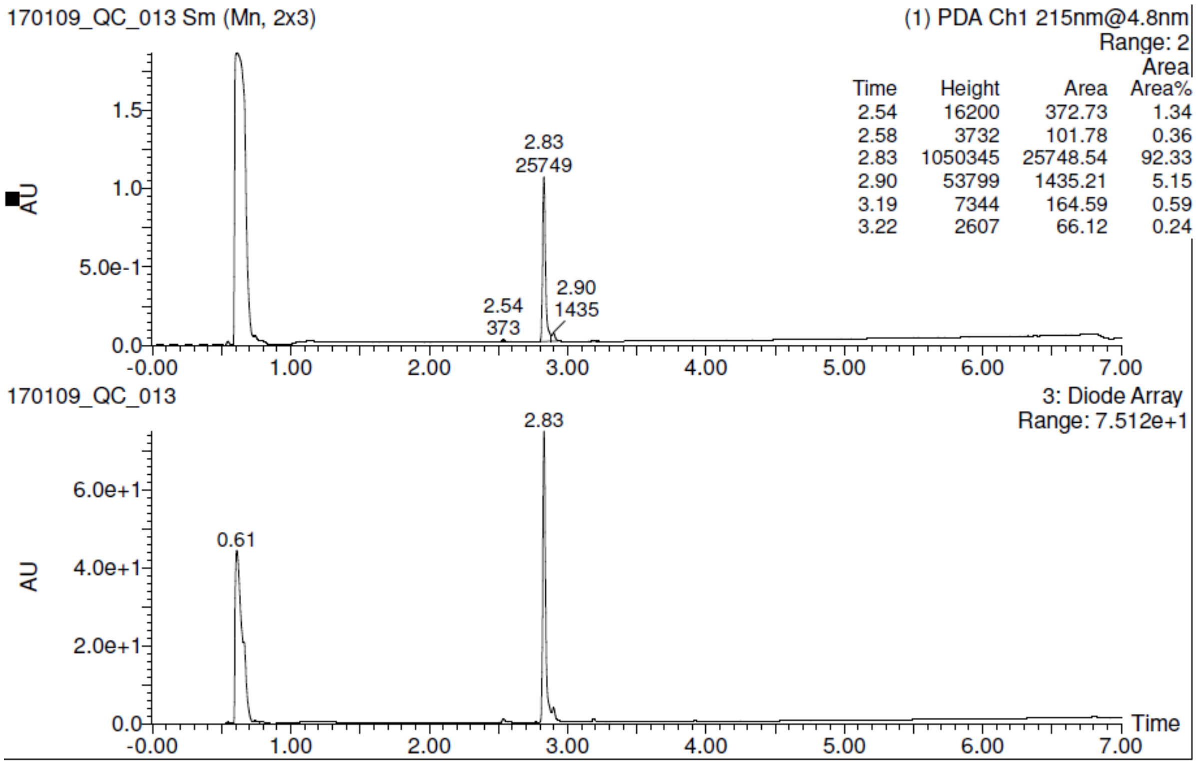

Time	Height	Area	Area Area%
2.54	16200	372.73	1.34
2.58	3732	101.78	0.36
2.83	1050345	25748.54	92.33
2.90	53799	1435.21	5.15
3.19	7344	164.59	0.59
3.22	2607	66.12	0.24

Compound **16**

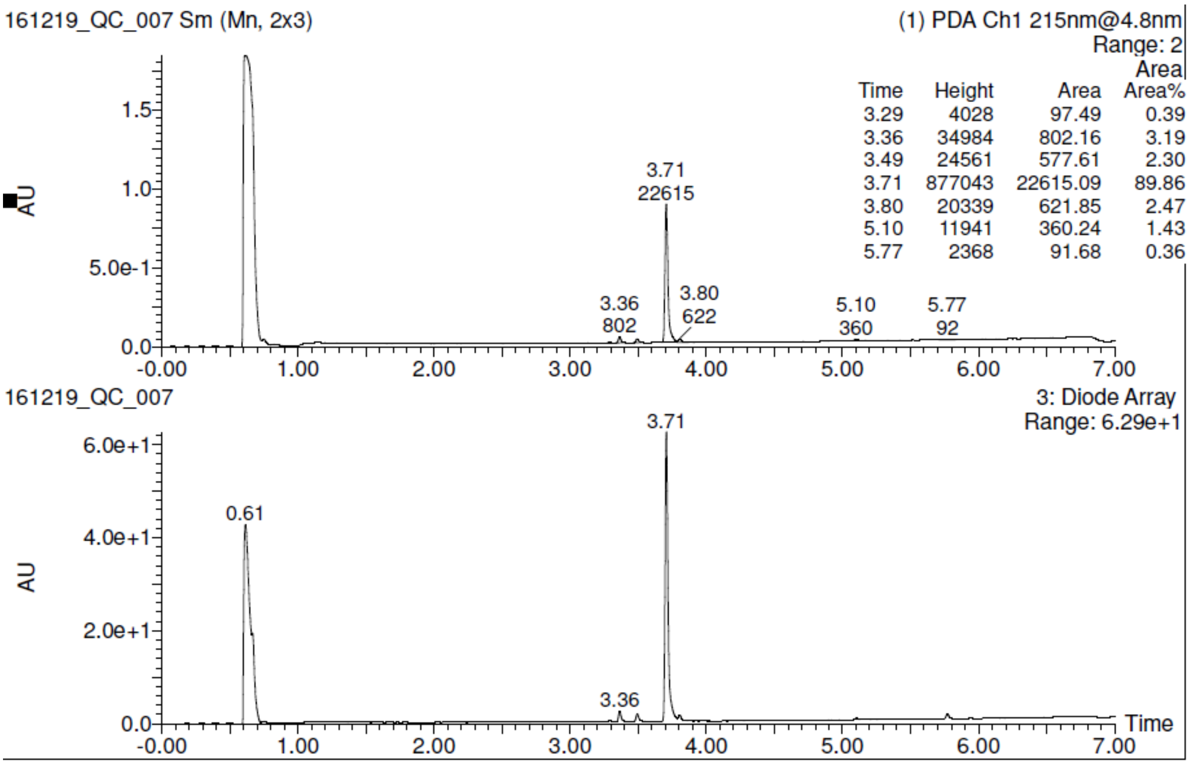

Time	Height	Area	Area Area%
3.29	4028	97.49	0.39
3.36	34984	802.16	3.19
3.49	24561	577.61	2.30
3.71	877043	22615.09	89.86
3.80	20339	621.85	2.47
5.10	11941	360.24	1.43
5.77	2368	91.68	0.36

Compound **17**

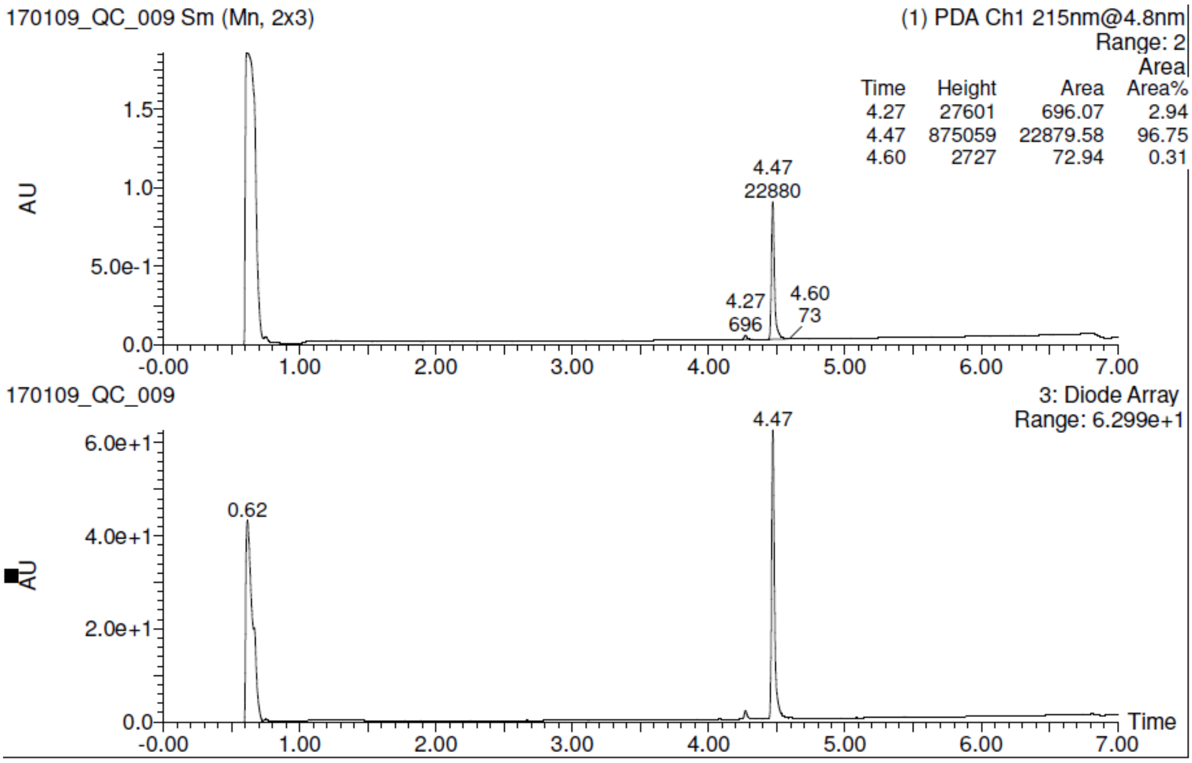

Compound **18**

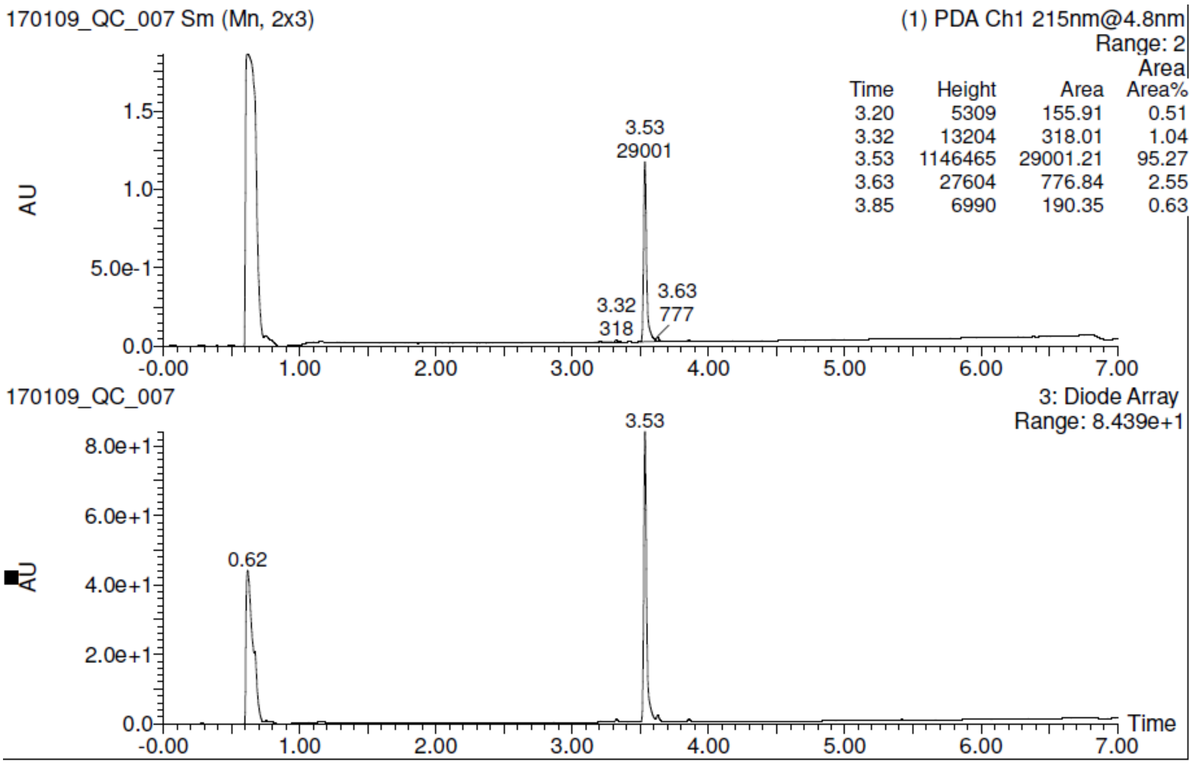

Compound **19**

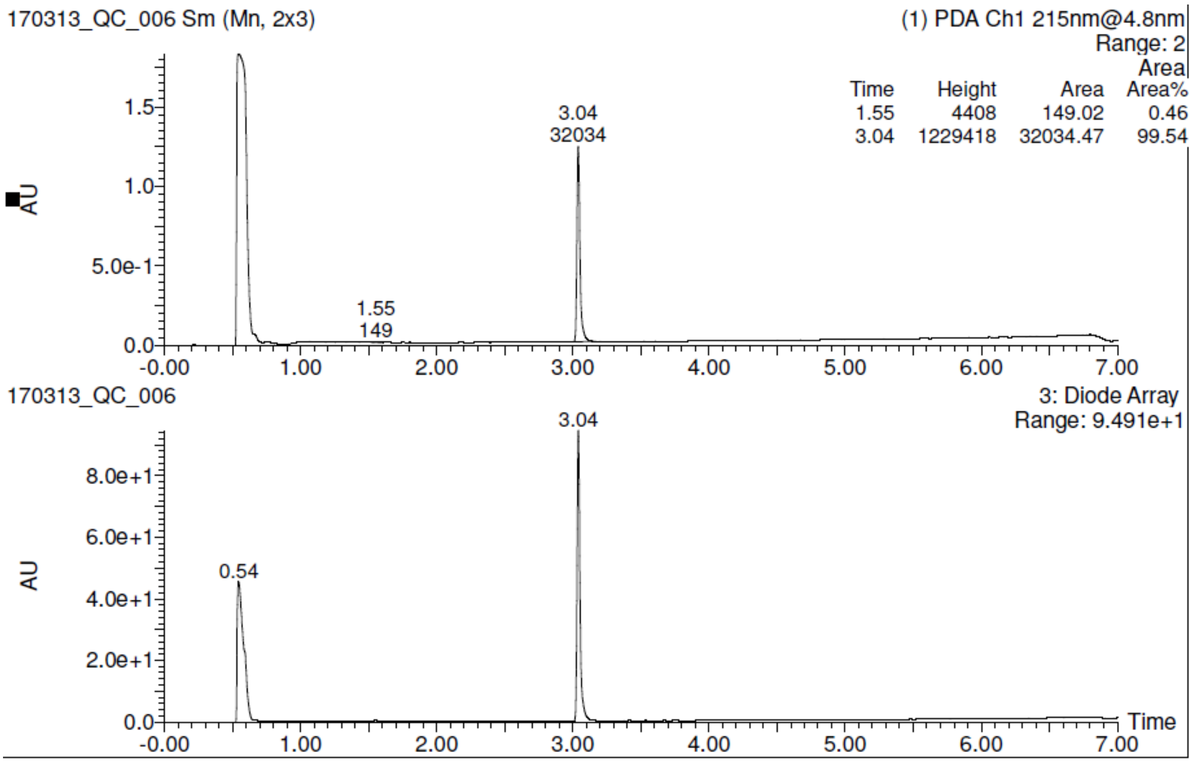

Compound **20**

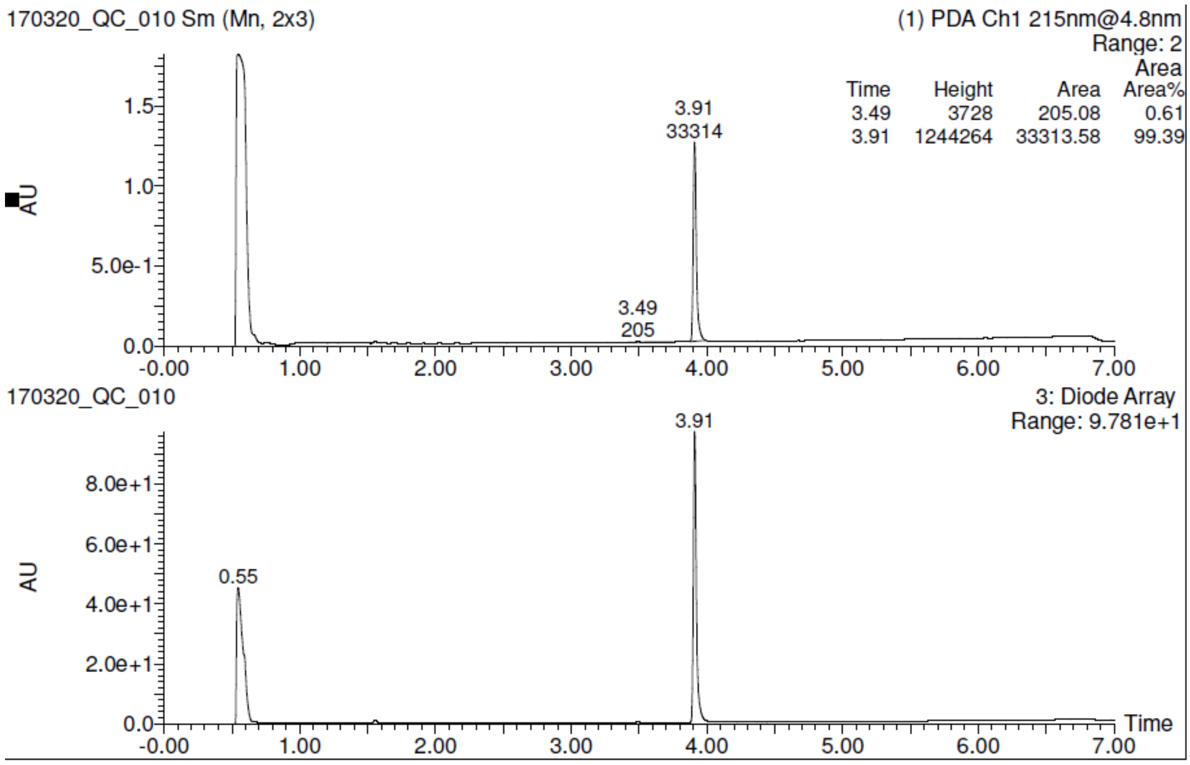

Compound **21**

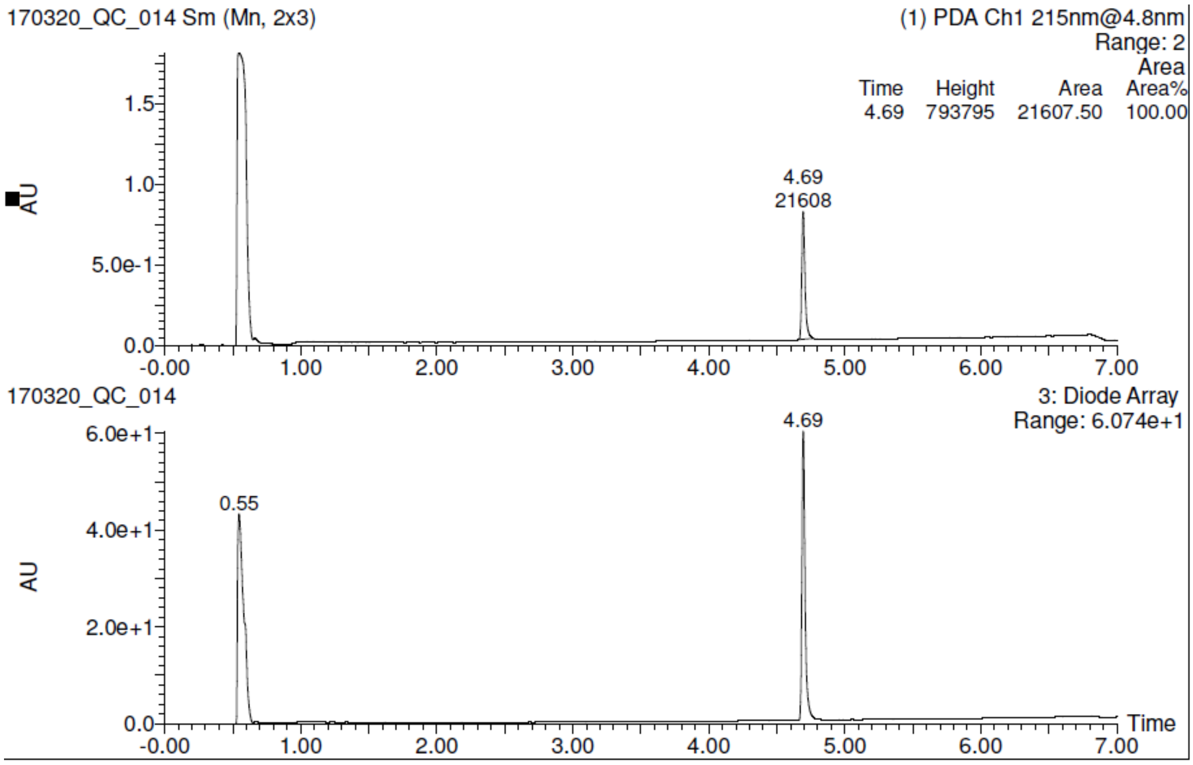

Compound **22**

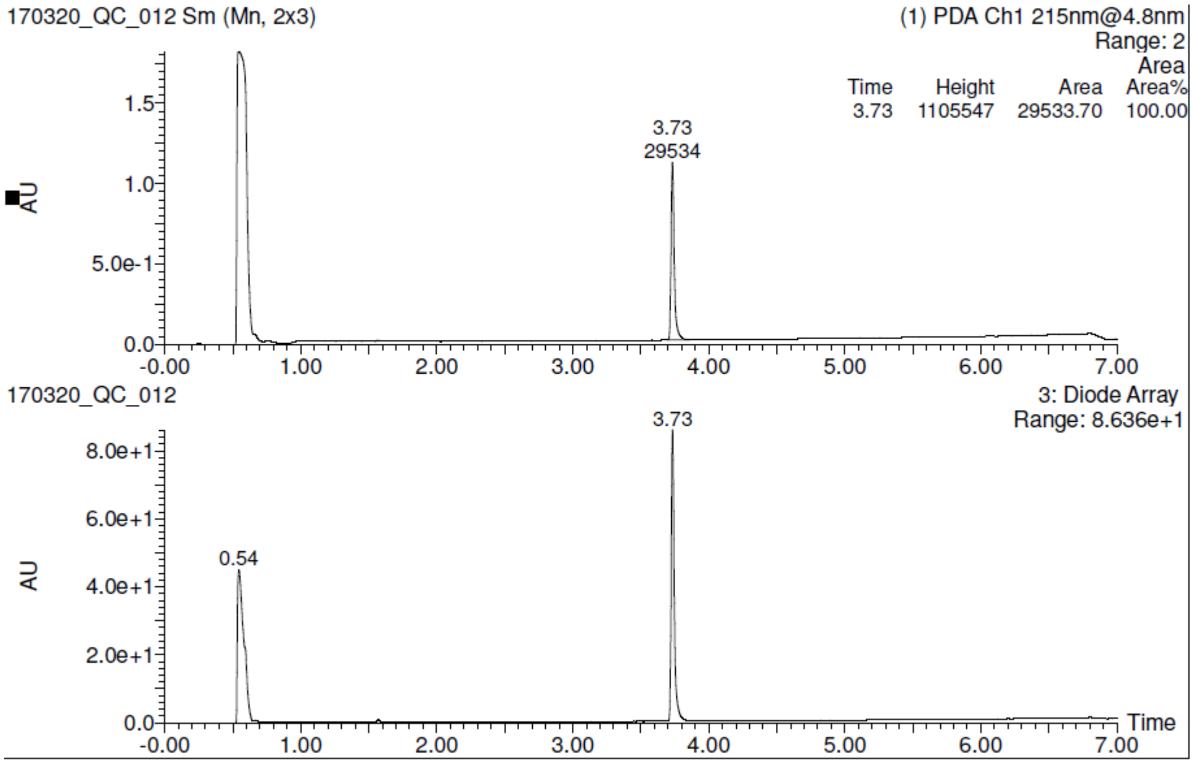

Compound **23**

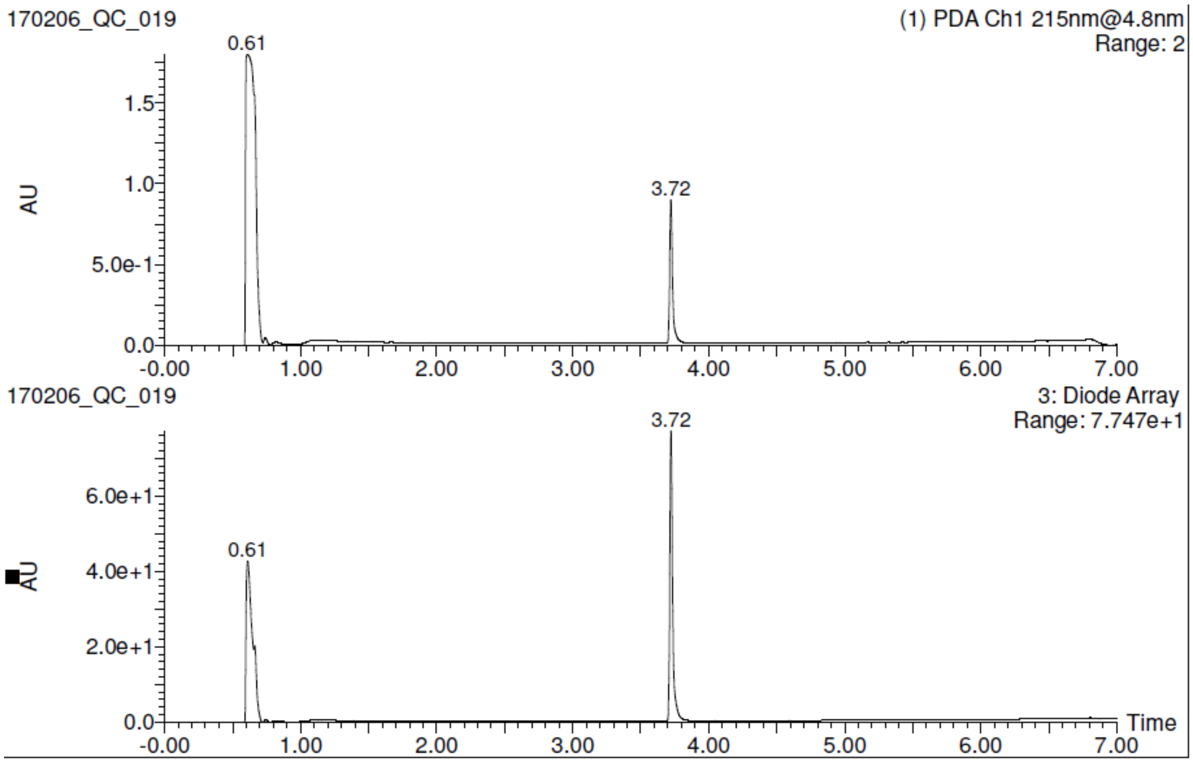

Compound **24**

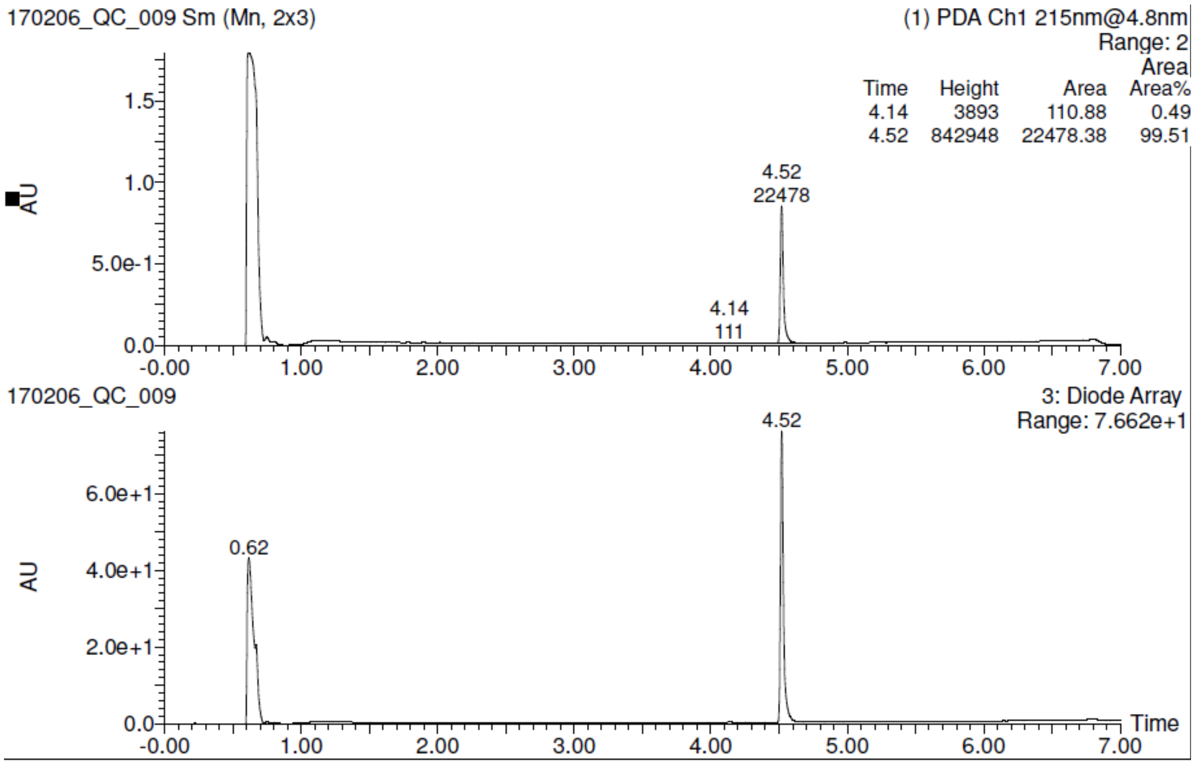

Time	Height	Area	Area%
4.14	3893	110.88	0.49
4.52	842948	22478.38	99.51

Compound **25**

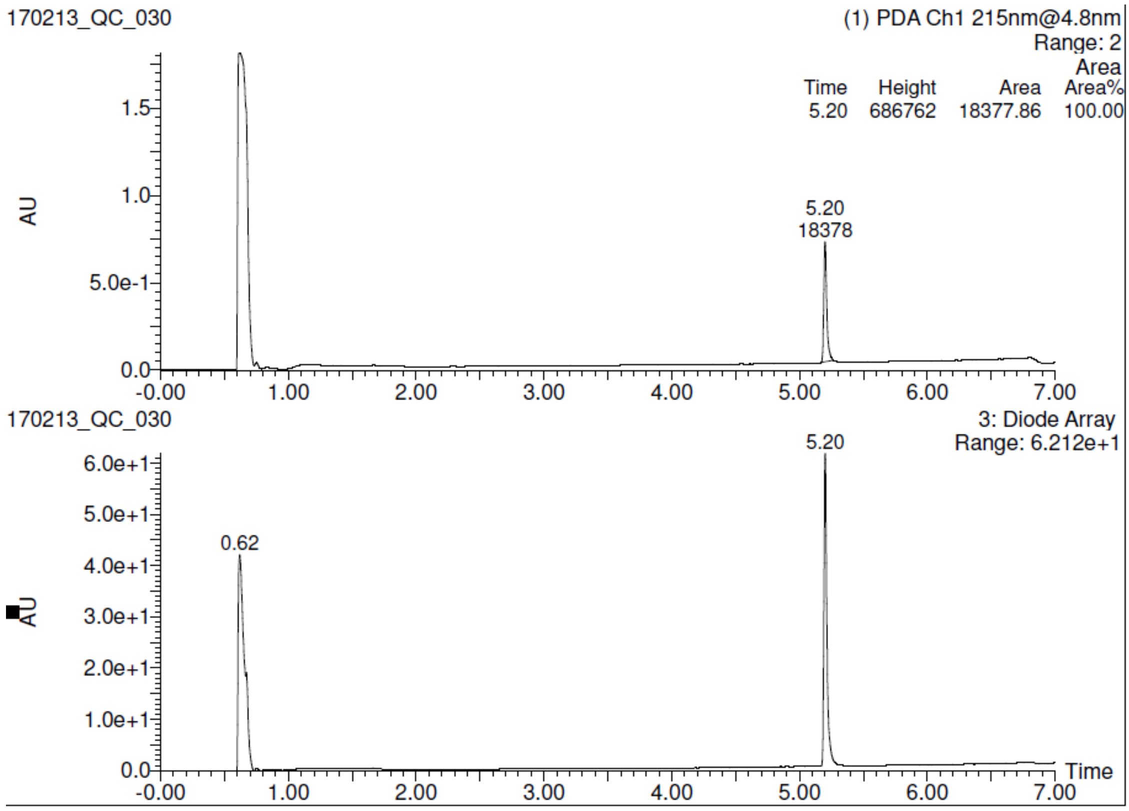

Compound **26**

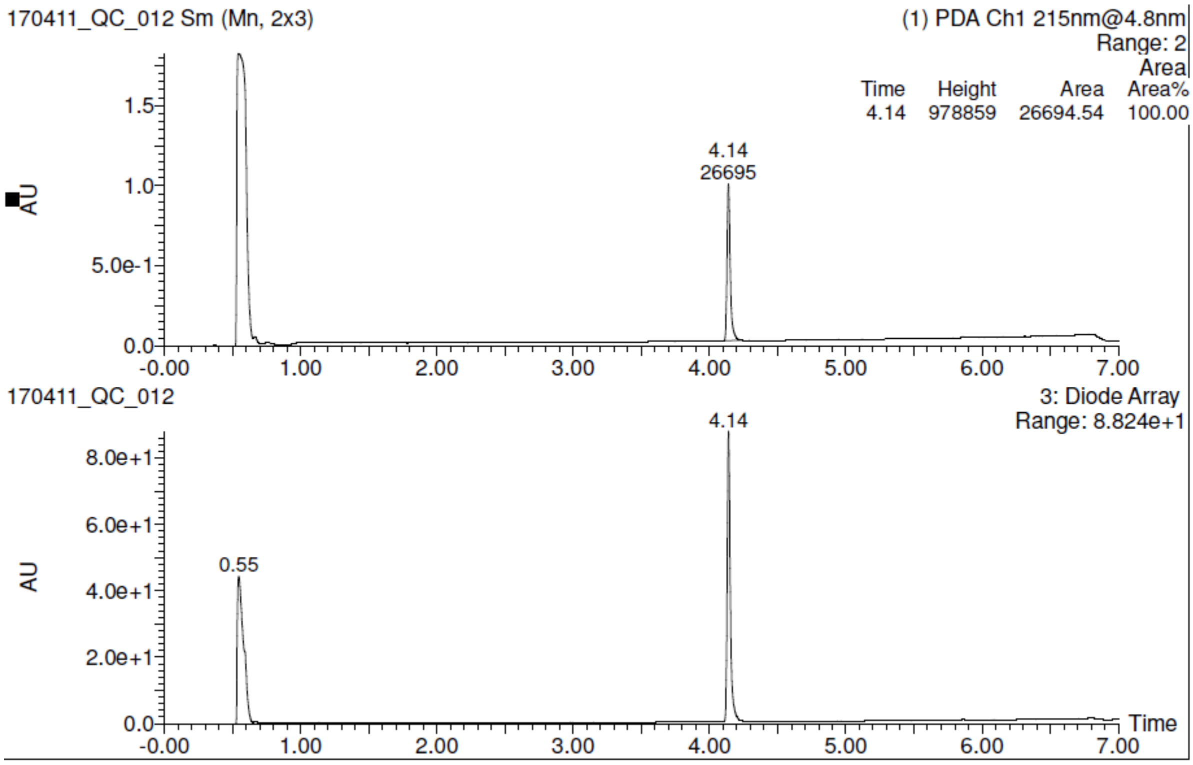

Compound **27**

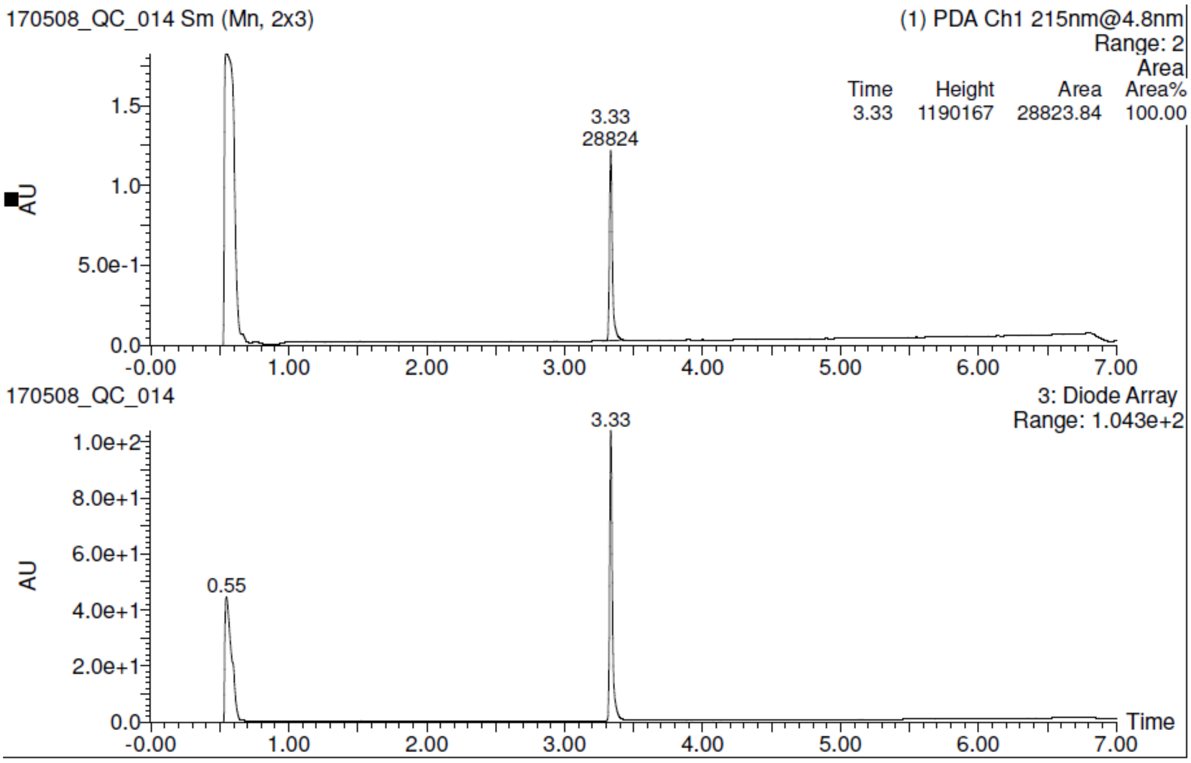

Compound **28**

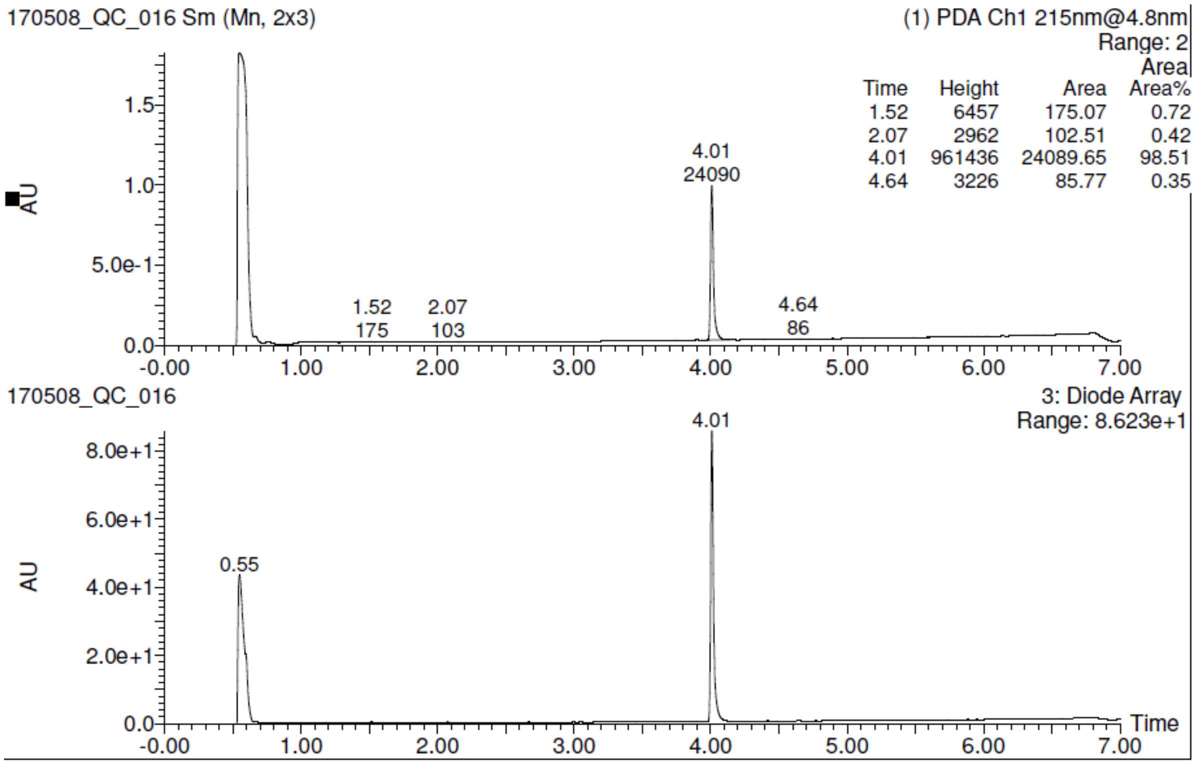

Compound **29**

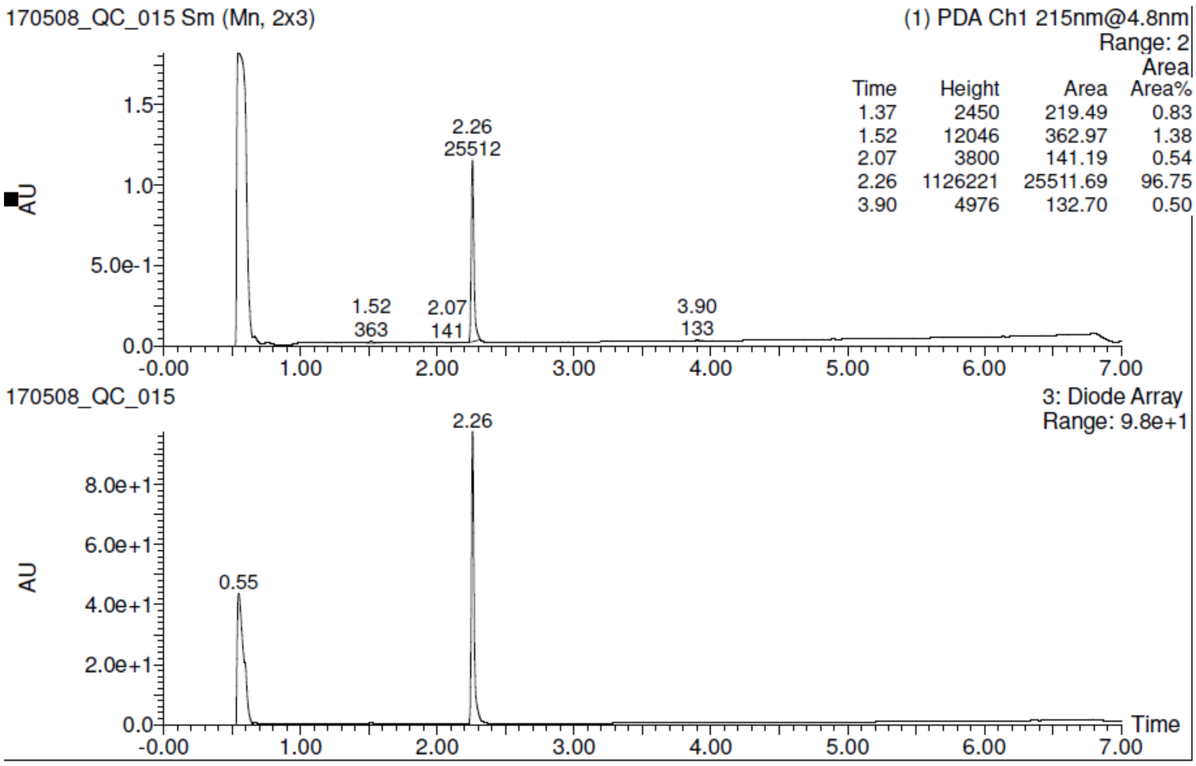

Compound **30**

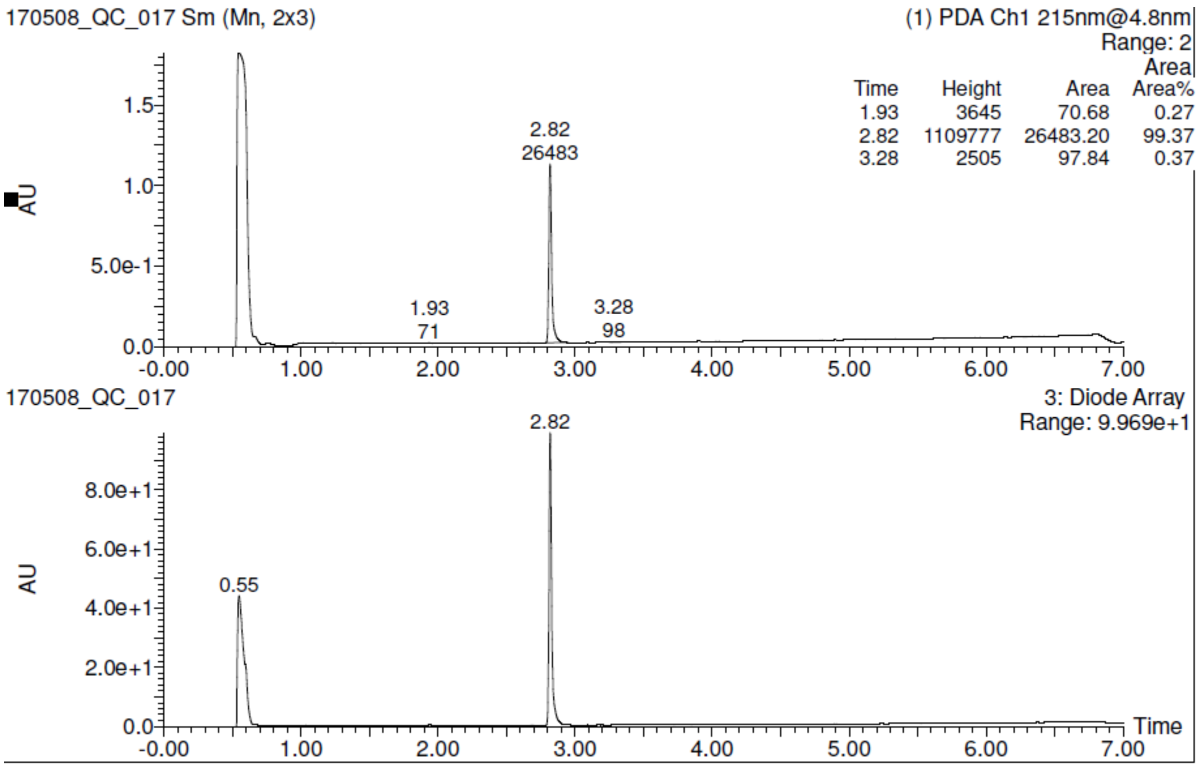

Compound **31**

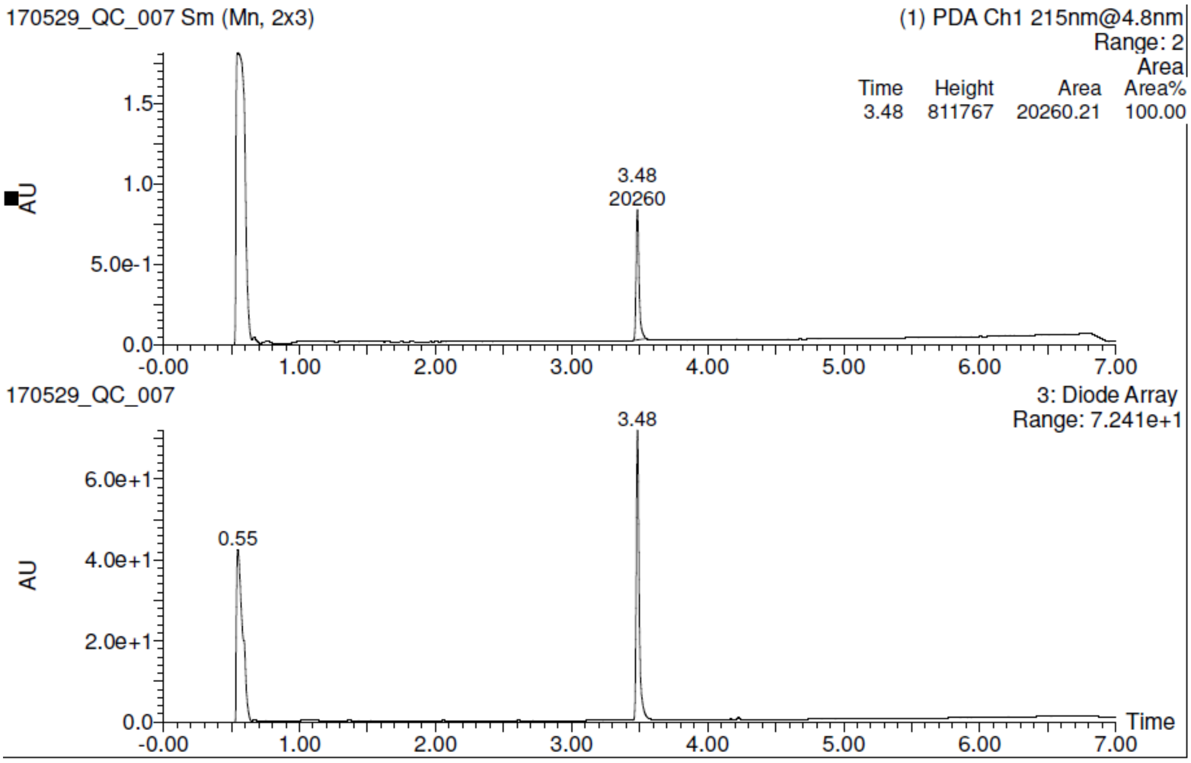

Compound **32**

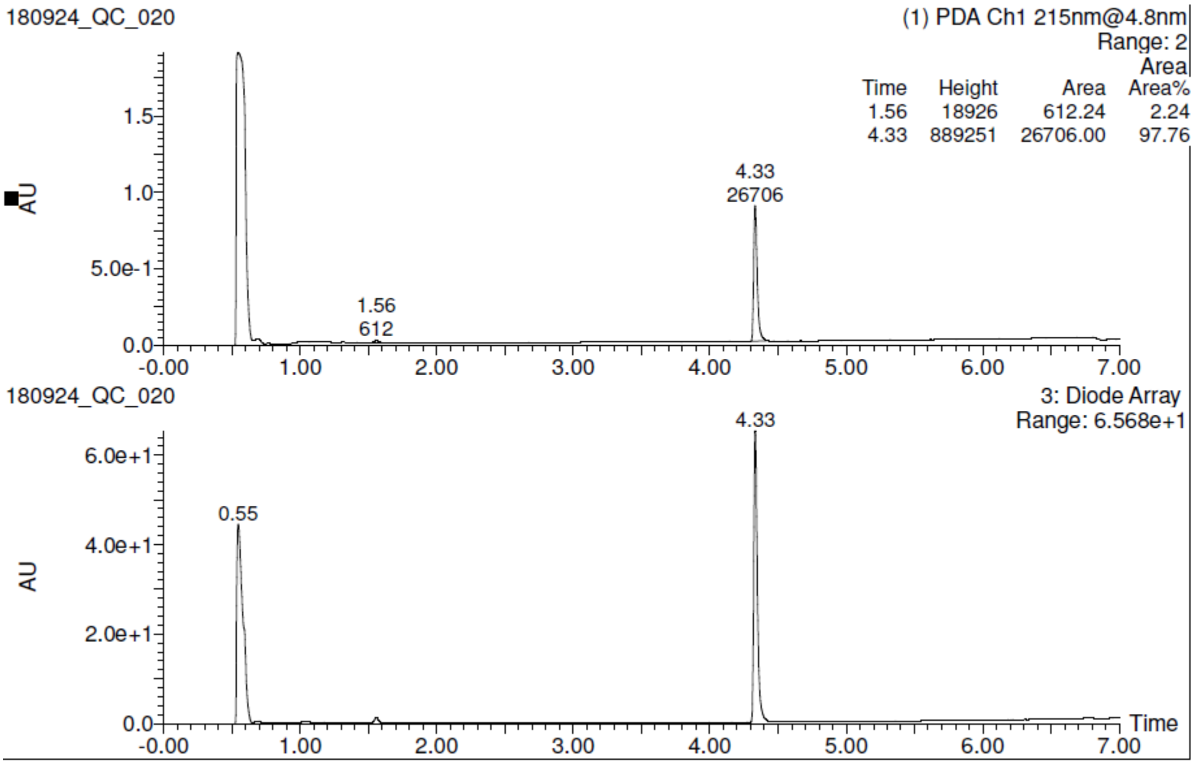

Compound **33**

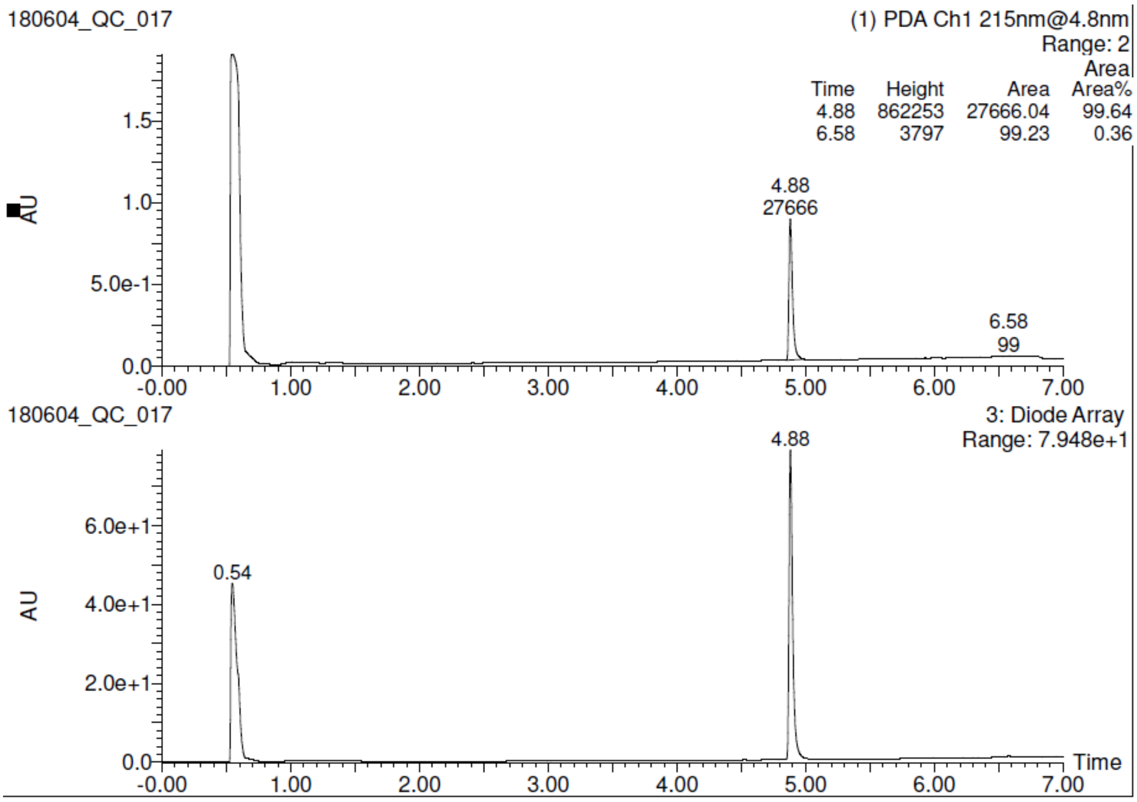

Compound **34**

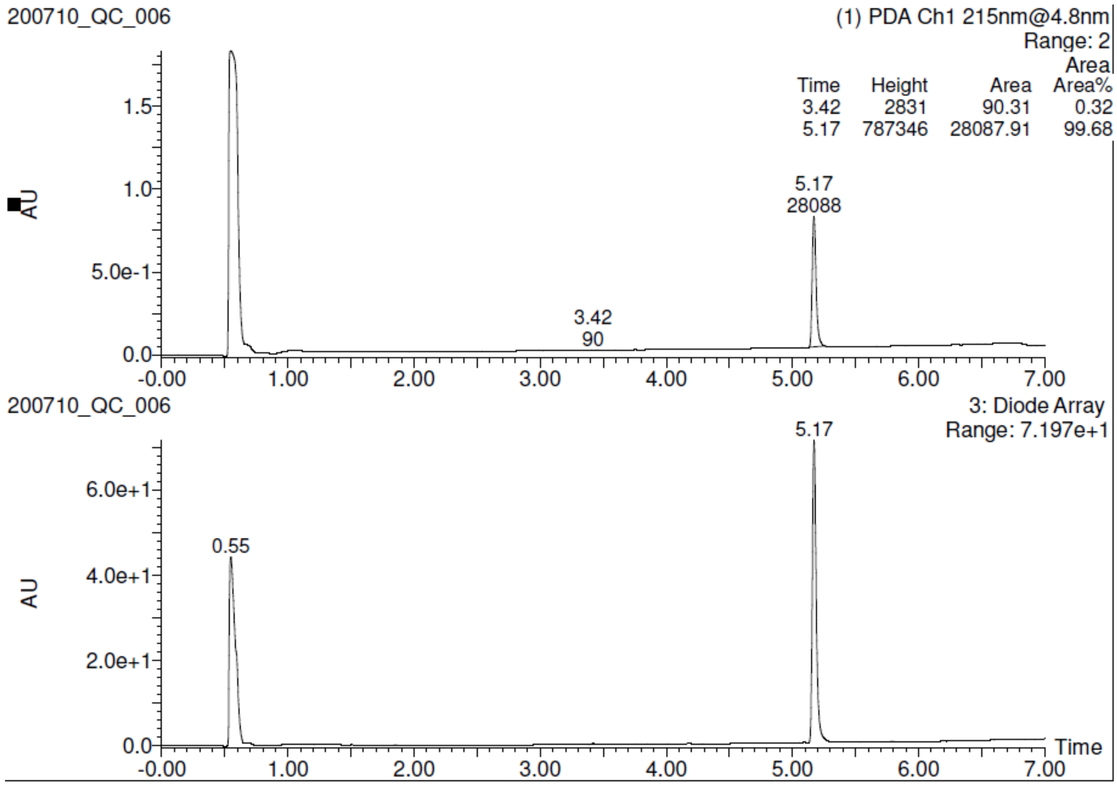

Compound **35**

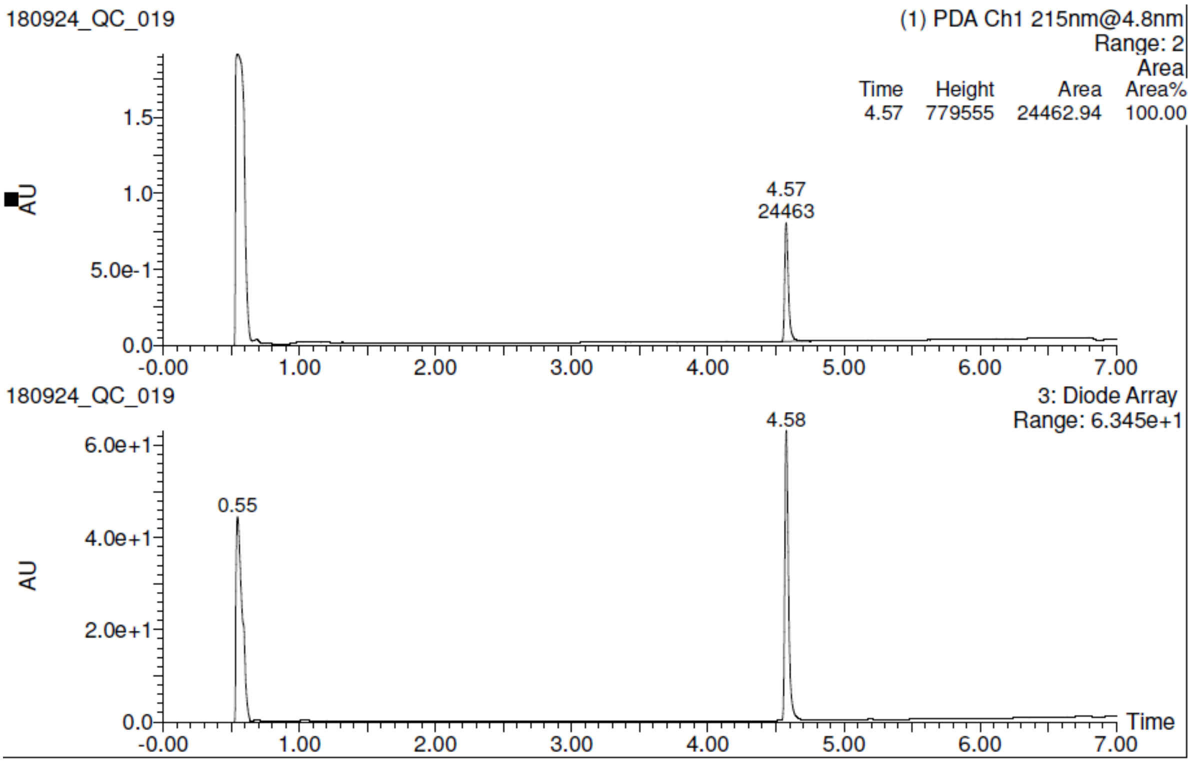

Compound **36**

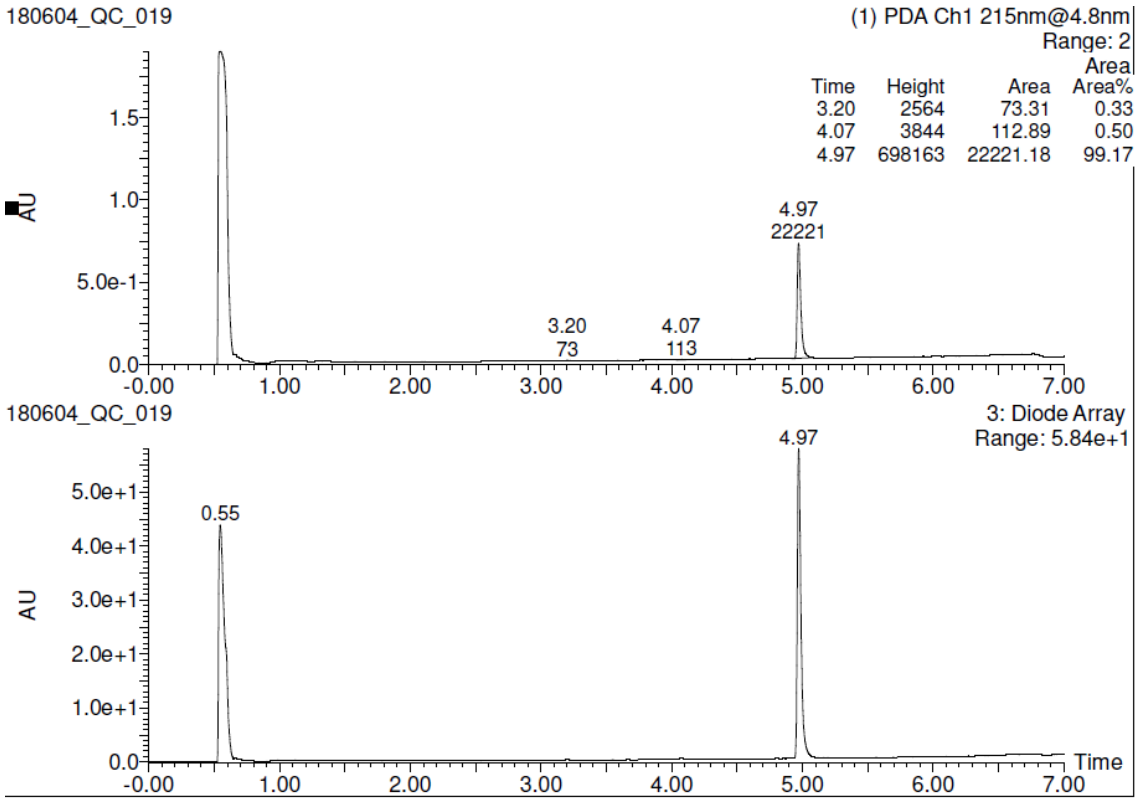

Compound **37**

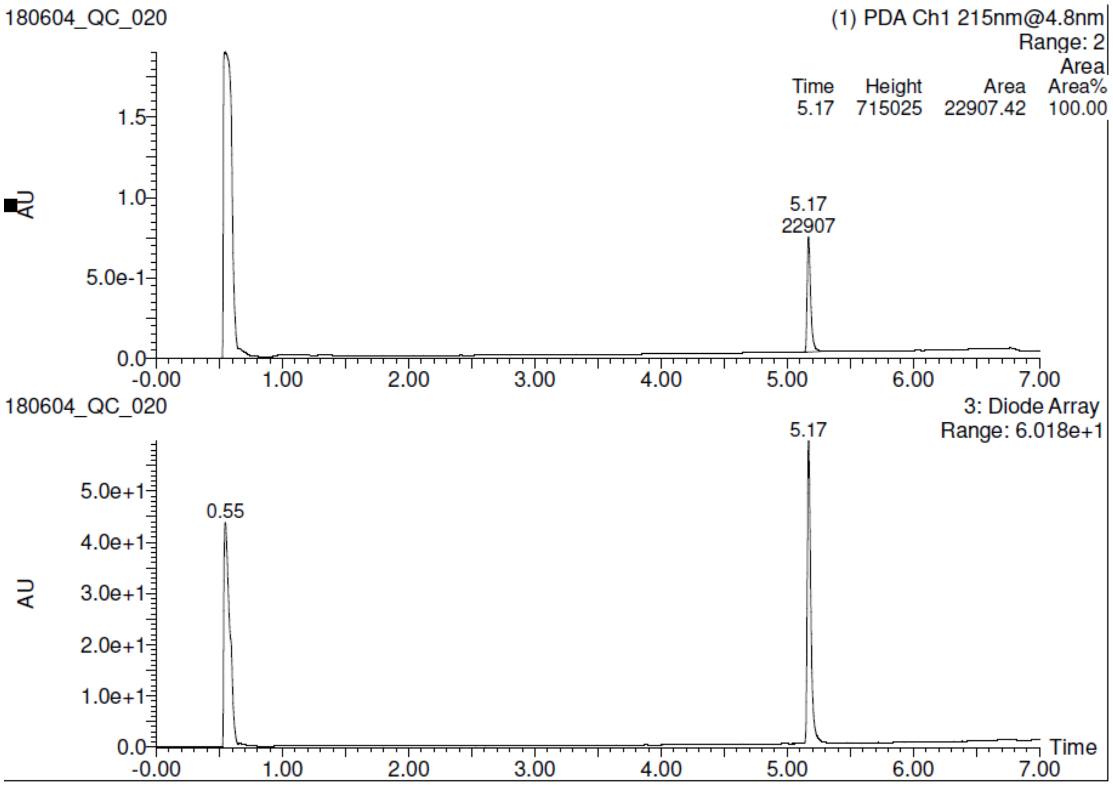

Compound **38**

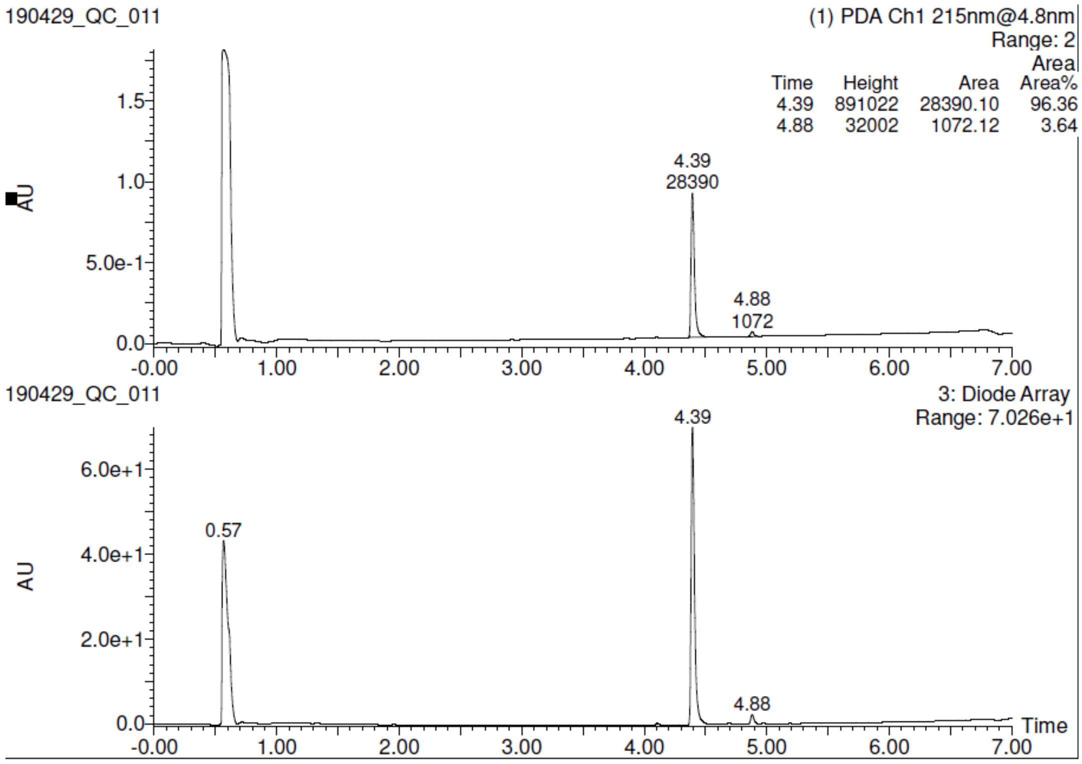

Compound **39**

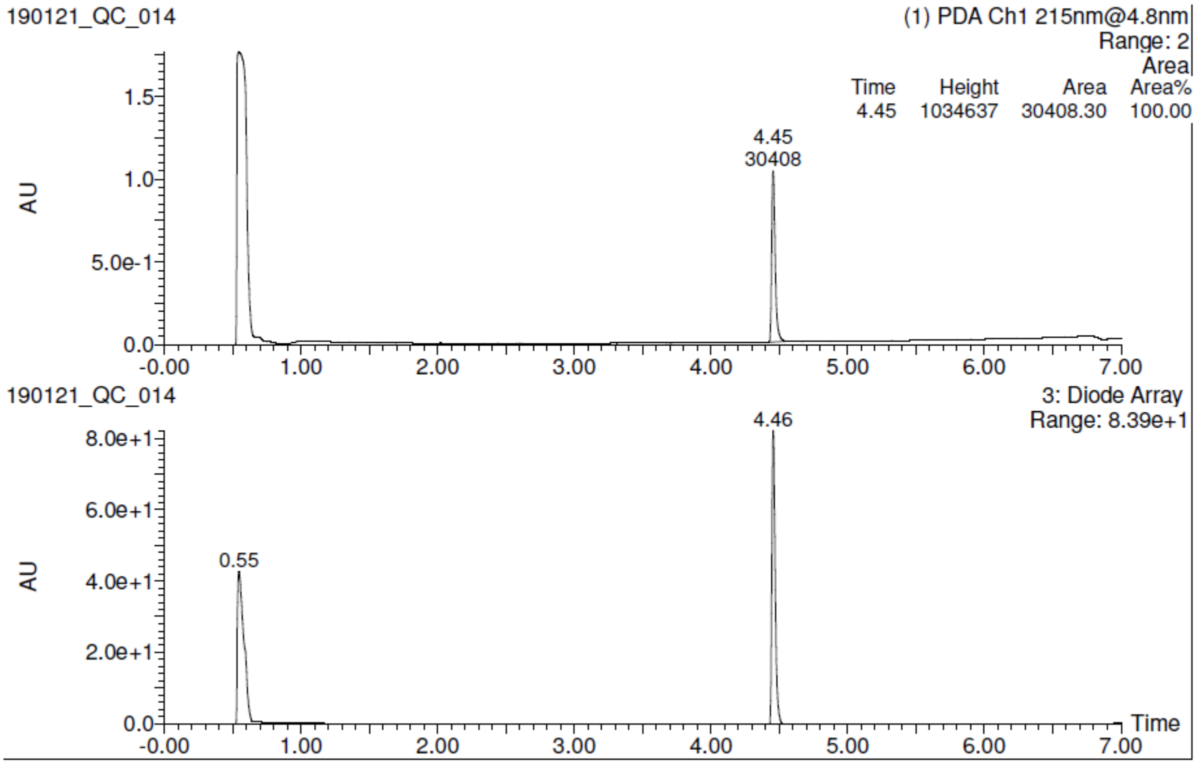

Compound **40**

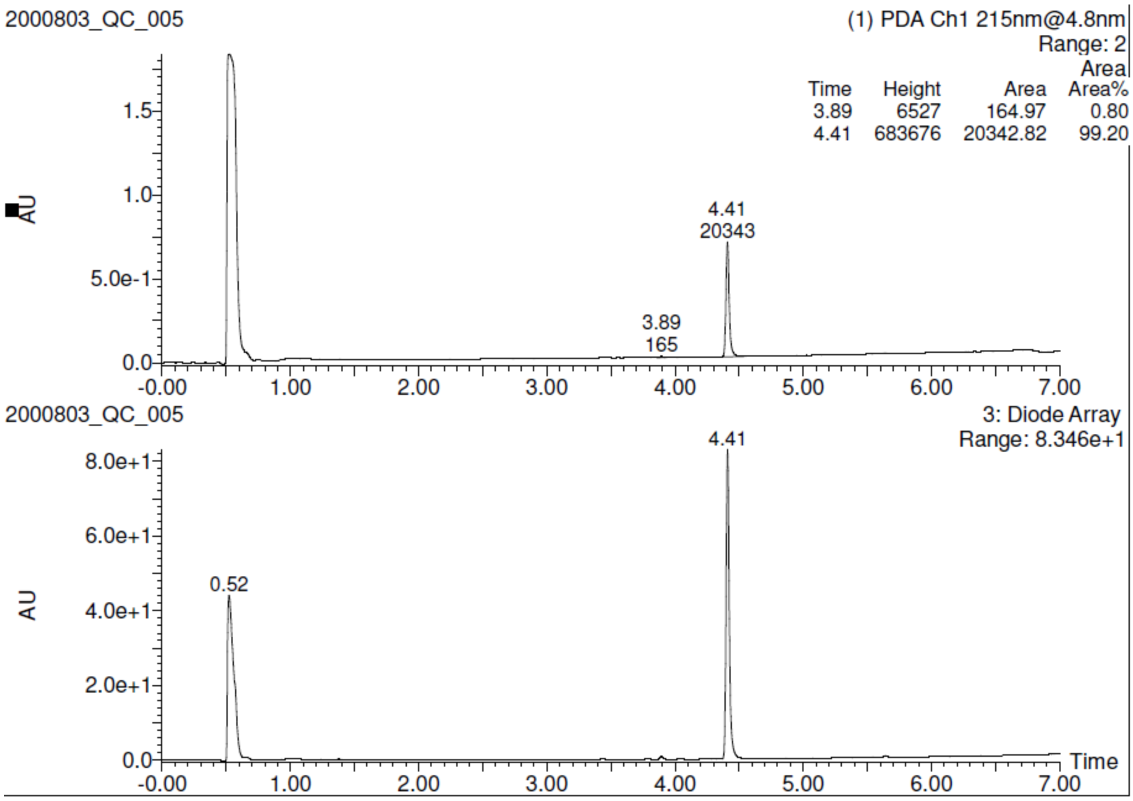

Compound **41**

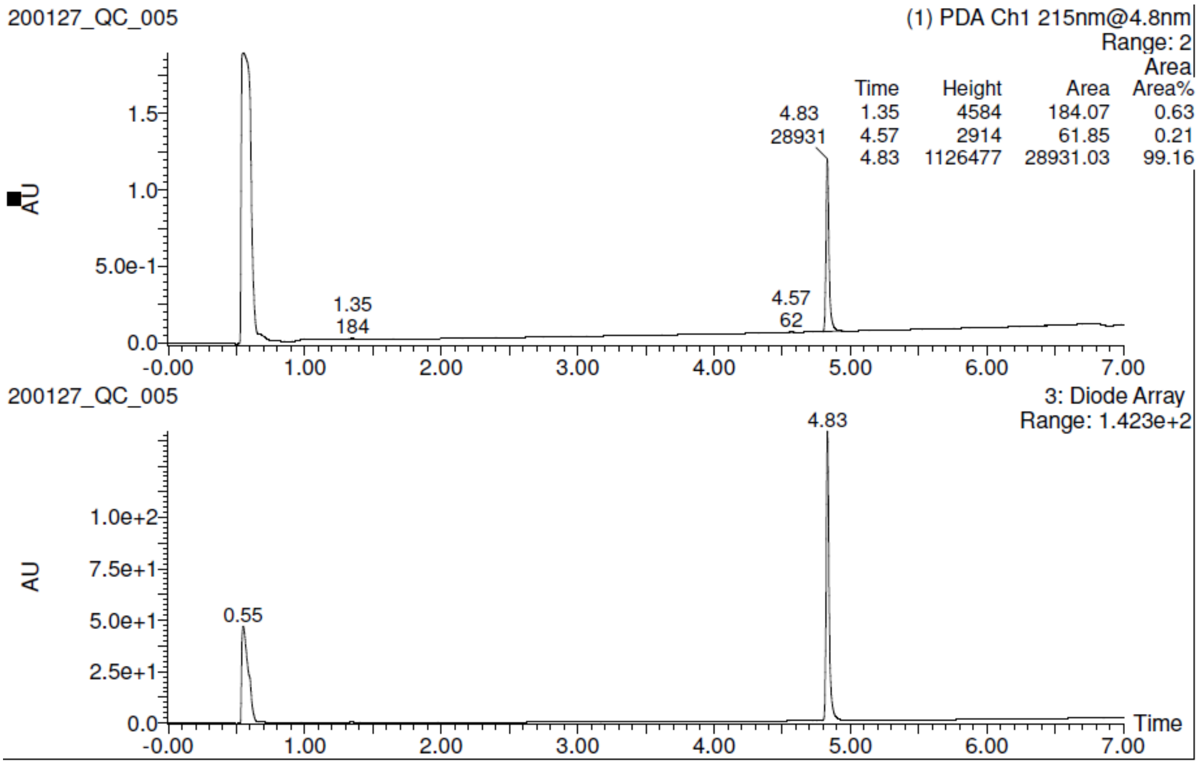

Compound **42**

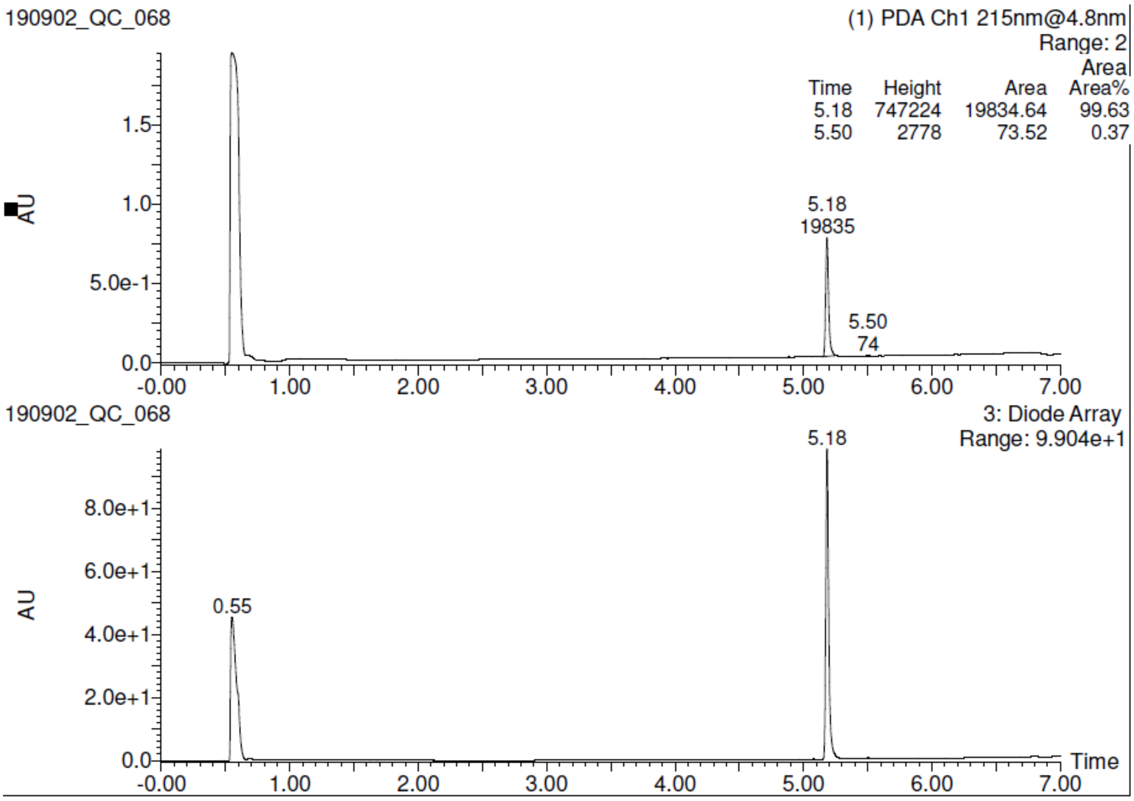

Compound **43**

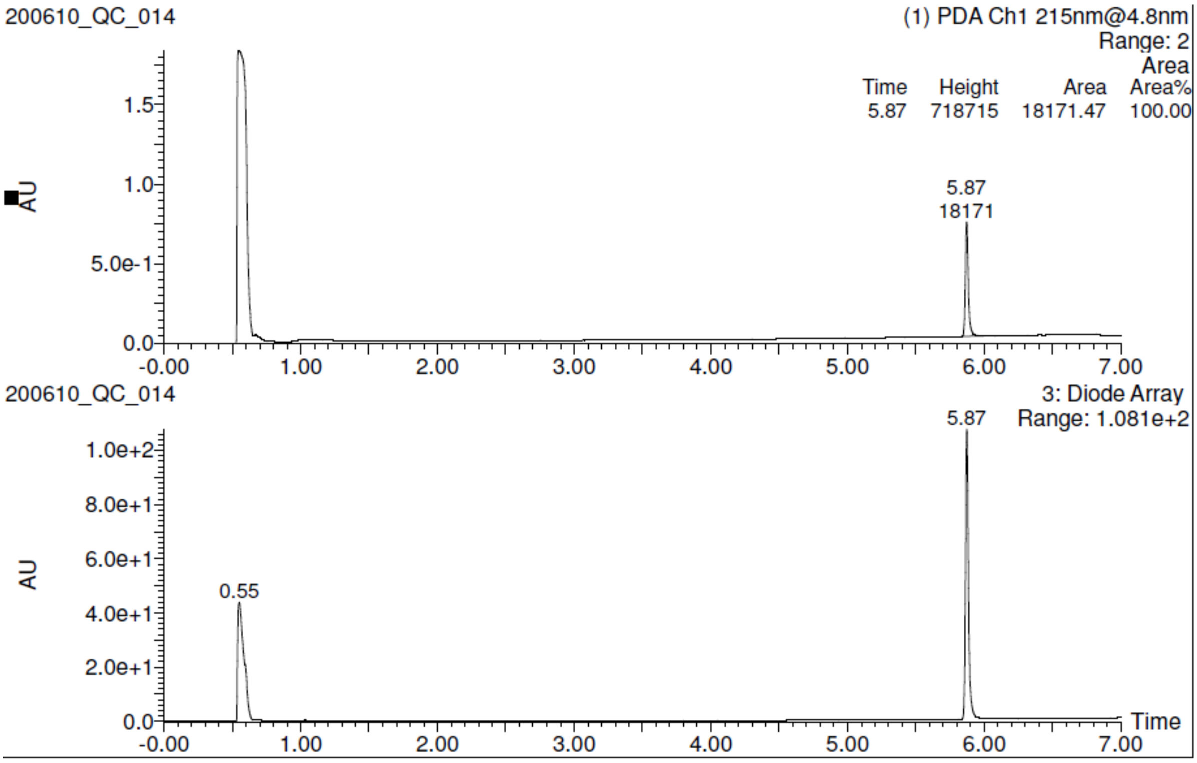

200610_QC_014
(1) PDA Ch1 215nm@4.8nm
Range: 2
Time Height Area Area%
5.87 718715 18171.47 100.00
5.87
18171
AU
1.5
1.0
5.0e-1
0.0
-0.00
1.00
2.00
3.00
4.00
5.00
6.00
7.00
200610_QC_014
3: Diode Array
Range: 1.081e+2
5.87
0.55
AU
1.0e+2
8.0e+1
6.0e+1
4.0e+1
2.0e+1
0.0
Time
